# Supplementary material for: Synthesis and Biological Evaluation of New Isoxazolyl Steroids as Anti-Prostate Cancer Agents
Source: Int J Mol Sci. 2022 Nov 4;23(21):13534. doi: 10.3390/ijms232113534 (PMC9656436; doi:10.3390/ijms232113534)

Supporting information to:

Rudovich, A. S.; Peřina, M.; Krech, A. V.; Novozhilova, M. Y.; Tumilovich, A. M.; Shkel, T. V.; Grabovec, I. P.; Kvasnica, M.; Mada, L.; Zavialova, M. G.; Mekhtiev, A. R.; Jorda, R.; Zhabinskii, V. N.; Khripach, V. A.

Synthesis and Biological Evaluation of New Isoxazolyl Steroids as Anti-Prostate Cancer Agents

Table S1. AR transcriptional and antiproliferative activities of novel derivatives.

Cmp.	AR transcriptional activity (%) ANTAGONIST MODE[a]			AR transcriptional activity (%) AGONIST MODE[b]			Viability after 72 h (GI_{50})[c]		
	50 μM	10 μM	2 μM	50 μM	10 μM	2 μM	LNCaP	LAPC-4	DU145
**24d**	103.6 ± 42.6	112.8 ± 21.1	109.0 ± 12.1	18.6 ± 3.6	21.1 ± 1.6	17.8 ± 0.1	> 50	> 50	> 50
**24e**	125.3 ± 27.4	101.1 ± 13.6	101.3 ± 9.5	12.8 ± 0.2	14.2 ± 1.4	14.7 ± 0.5	> 50	> 50	> 50
**24g**	111.6 ± 19.9	112.5 ± 14.5	112.9 ± 10.9	19.7 ± 1.6	17.5 ± 0.5	15.6 ± 0.6	> 50	> 50	> 50
**24j**	21.6 ± 7.8	84.8 ± 17.4	100.2 ± 13.5	6.1 ± 1.5	12.6 ± 0.1	14.4 ± 2.8	25.8 ± 0.6	18.2 ± 1.2	> 50
**27**	63.5 ± 8.0	62.0 ± 14.8	89.7 ± 9.9	20.4 ± 4.2	20.9 ± 2.9	17.4 ± 0.5	> 50	> 50	> 50
**32**	31.1 ± 13.3	87.8 ± 20.9	99.7 ± 17.4	14.6 ± 0.5	12.1 ± 2.7	14.0 ± 1.1	19.5 ± 0.1	18.9 ± 7.2	> 50
**36**	67.4 ± 21.9	72.5 ± 15.7	101.1 ± 18.1	16.3 ± 1.7	12.0 ± 0.5	14.7 ± 0.2	> 50	19.0 ± 7.8	> 50
**38**	78.2 ± 24.9	92.6 ± 20.5	96.1 ± 11.2	7.3 ± 1.1	13.0 ± 0.4	14.6 ± 1.0	> 50	> 50	> 50
**41a**	50.8 ± 18.8	73.7 ± 14.9	98.2 ± 17.5	12.1 ± 1.3	14.7 ± 0.1	14.5 ± 1.5	> 50	> 50	> 50
**Gal**	3.0 ± 1.8	35.1 ± 3.3	65.2 ± 6.1	2.4 ± 1.1	10.8 ± 3.1	15.4 ± 0.4	46.8 ± 0.1	28.6 ± 0.6	47.6 ± 0.2

[a] measured in the presence of compound and 1 nM R1881 and normalized to signal of 1 nM R1881 = 100%, determined in duplicate and repeated twice, mean ± SD is presented.
[b] measured in the presence of compound only, normalized to signal of 1 nM R1881 = 100%, measured in duplicate and repeated twice, mean ± SD is presented.
[c] measured at least in duplicate, mean ± SD is presented.

Table S2. Raw data for **Figure 2. (A)** Transcriptional activity of AR measured in reporter cell line in both antagonist (competition with 1 nM R1881) and agonist (presence of compound alone) mode upon treatment with different concentration of **24j**.

Concentration (μM)	AR transcriptional activity (%) after treatment with **24 j** (antagonist mode)				AR transcriptional activity (%) after treatment with **24 j** (agonist mode)			
60.00	20.55	20.45	15.39	15.98	6.48	8.33	7.15	5.97
20.00	55.89	57.74	58.97	51.32	14.35	16.25	14.56	15.38
6.67	78.82	77.07	74.29	83.26	16.97	15.48	17.44	20.21
2.22	88.25	93.78	91.50	88.12	15.89	17.90	21.24	19.34
0.74	95.70	97.95	95.17	95.70	17.18	19.44	20.73	22.58
0.25	98.38	91.23	93.41	98.15	18.16	19.80	20.78	20.68

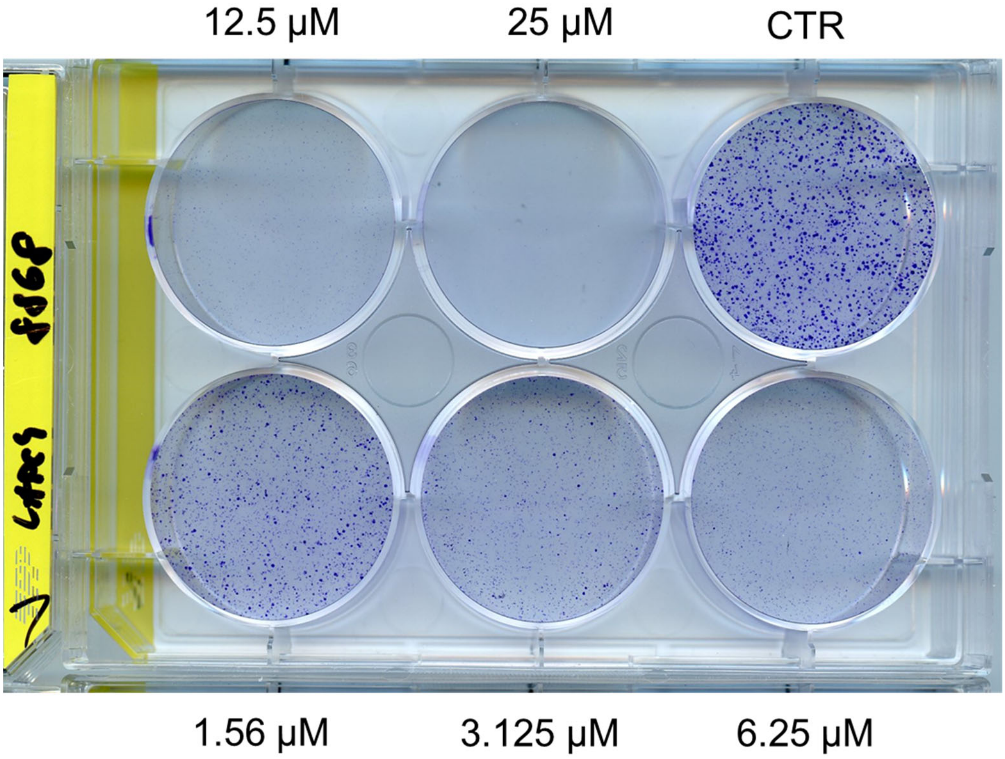

Figure S1. Raw picture for **Figure 2. (B)** Colony formation assay of PCa cells LAPC-4 after treatment with **24j** for 10 days.

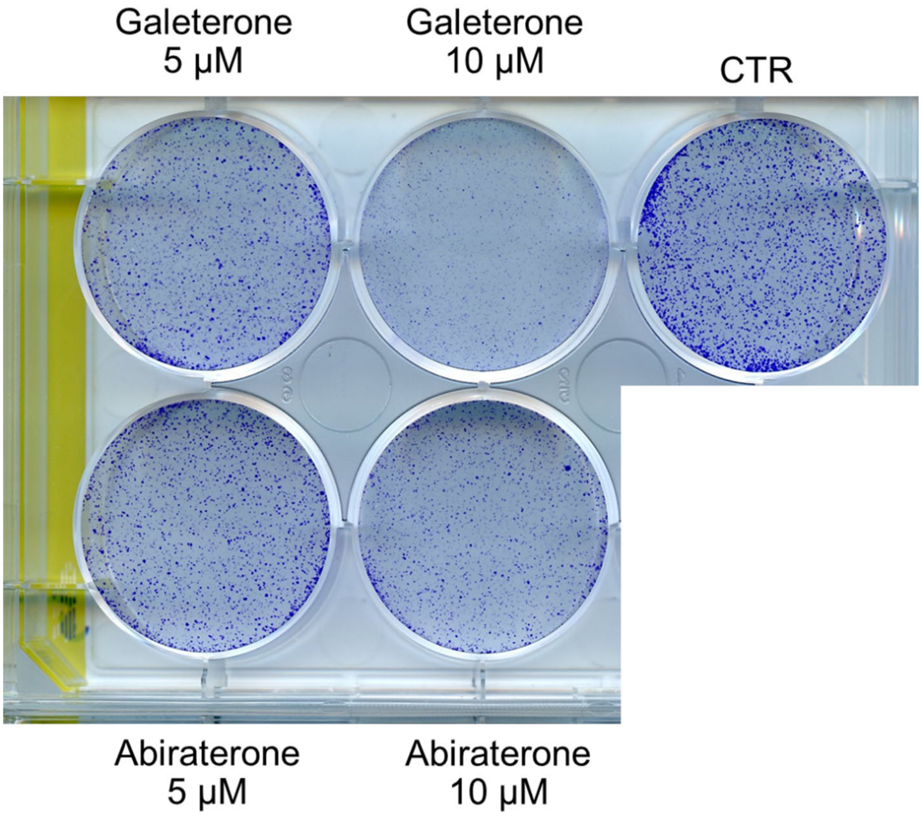

Figure S2. Colony formation assay of PCa cells LAPC-4 after treatment with standards for 10 days.

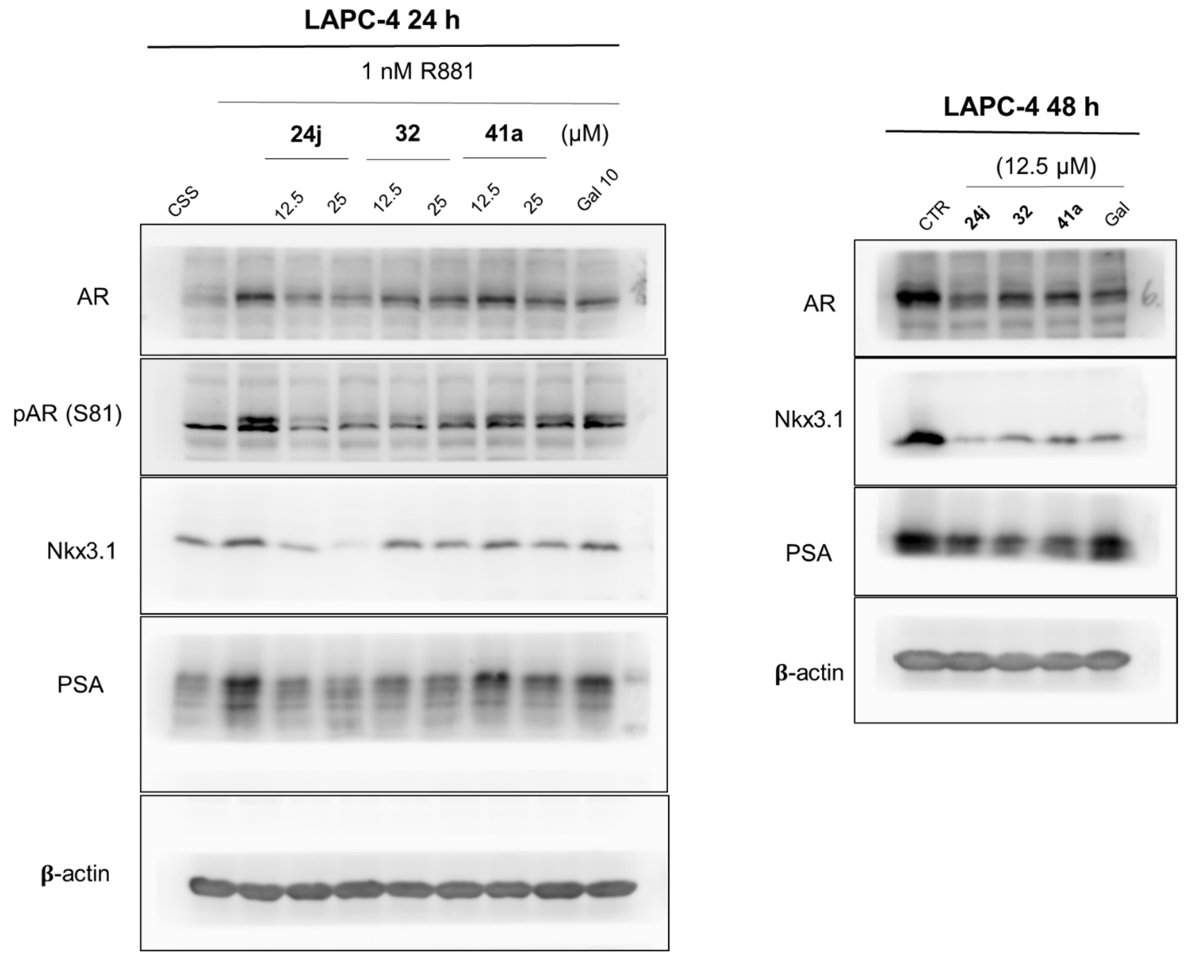

Figure S3. Raw picture for **Figure 3**. Western blotting analysis of AR and AR-regulated proteins in treated LAPC-4 cells.

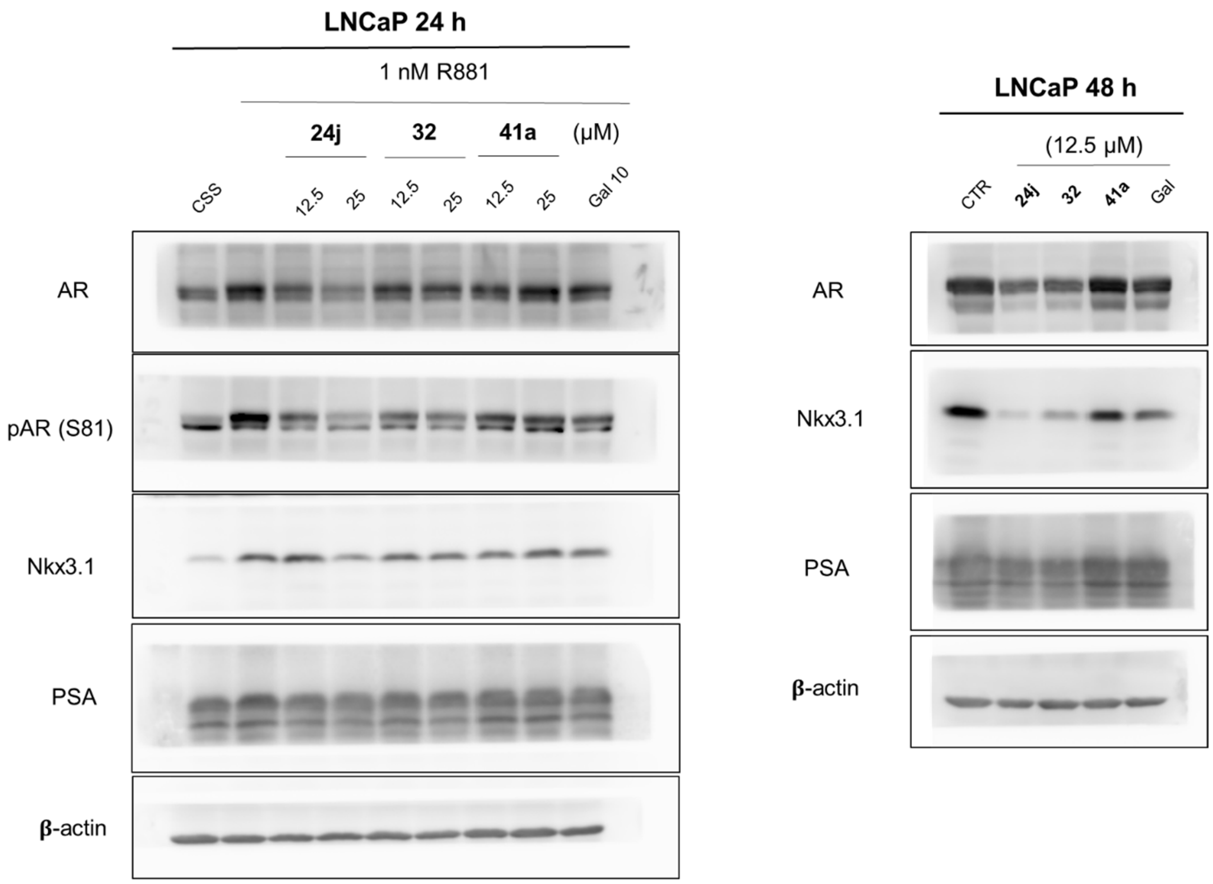

Figure S4. Raw picture for **Figure 3**. Western blotting analysis of AR and AR-regulated proteins in treated LAPC-4 cells.

1H and 13C NMR spectra

Methyl 2-(3β-((*tert*-butyldimethylsilyl)oxy)-androst-5-en-17-yl)acetate (**12**)

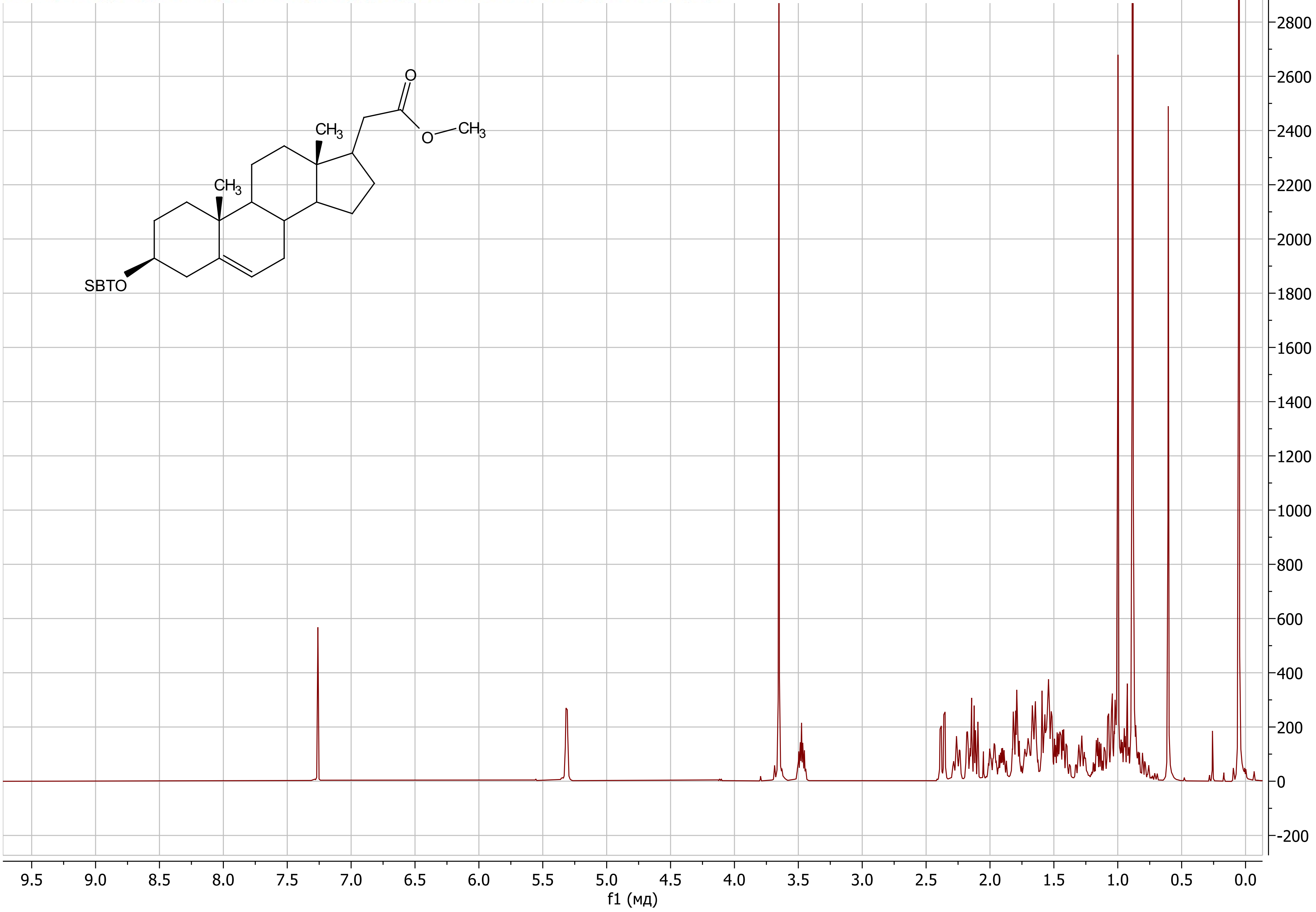

Methyl 2-(3β-((*tert*-butyldimethylsilyl)oxy)-androst-5-en-17-yl)acetate (**12**)

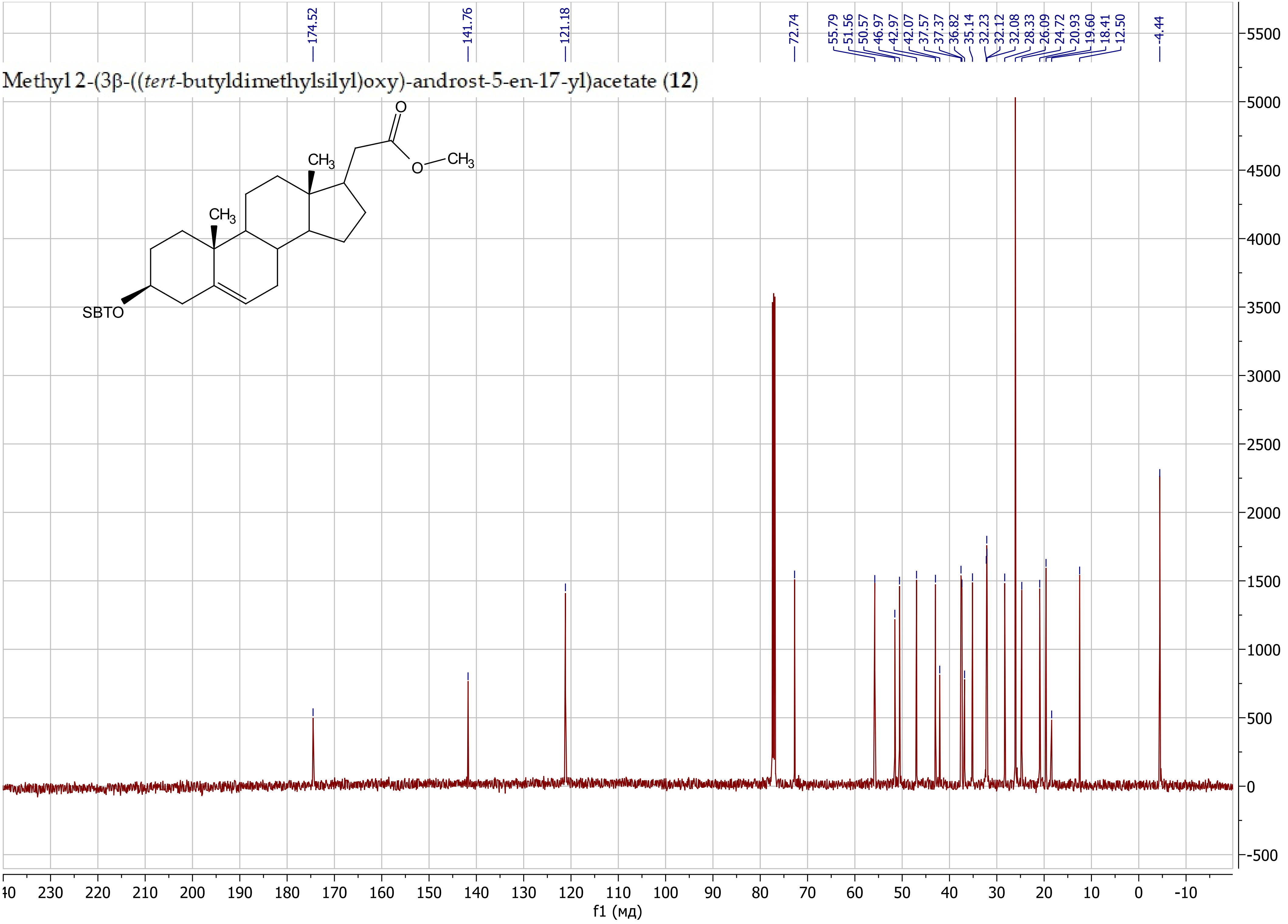

2-(3β-((*tert*-Butyldimethylsilyl)oxy)-androst-5-en-17-yl)-*N*-methoxy-*N*-methylacetamide (**14**)

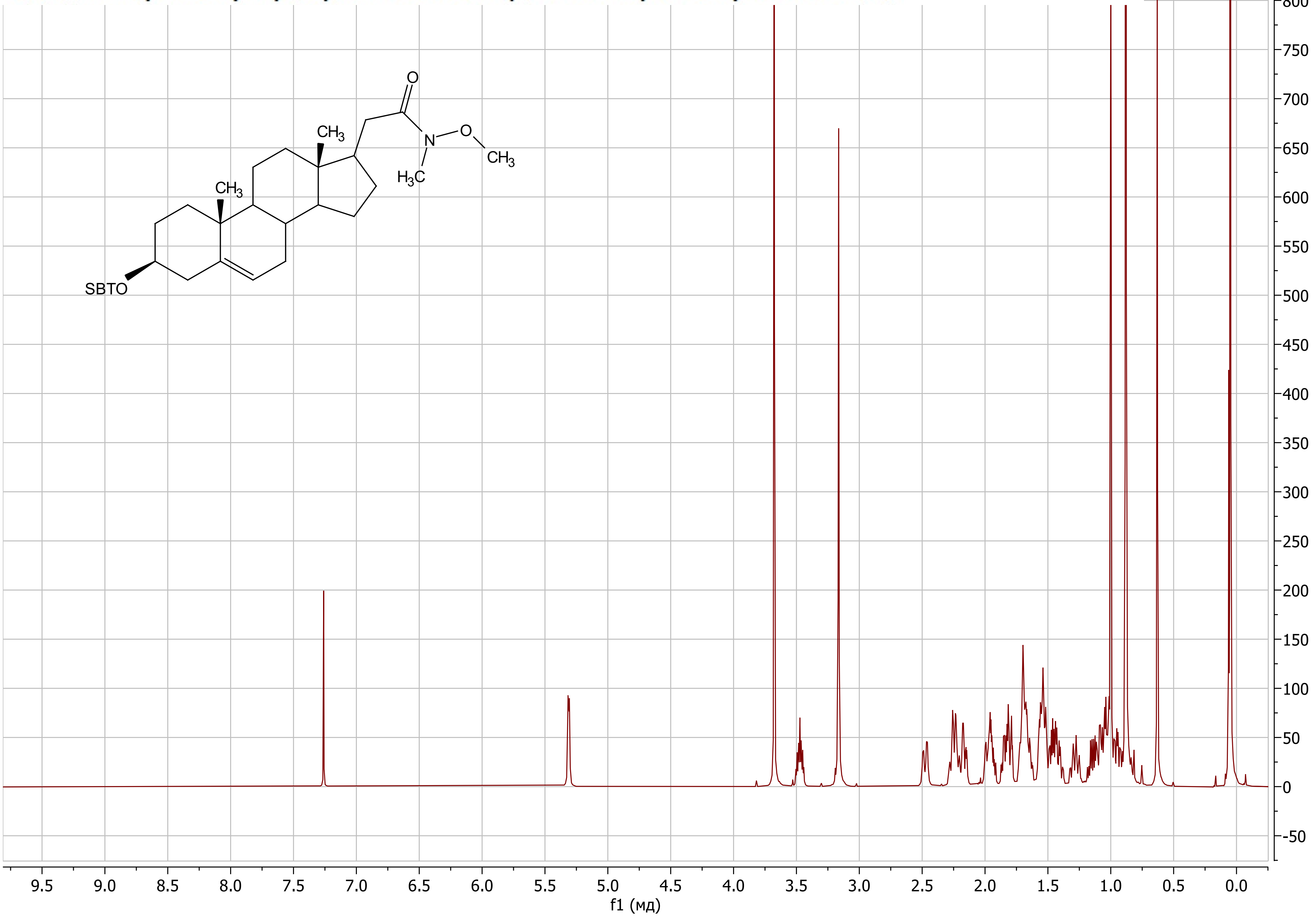

2-(3β-((*tert*-Butyldimethylsilyl)oxy)-androst-5-en-17-yl)-*N*-methoxy-*N*-methylacetamide (**14**)

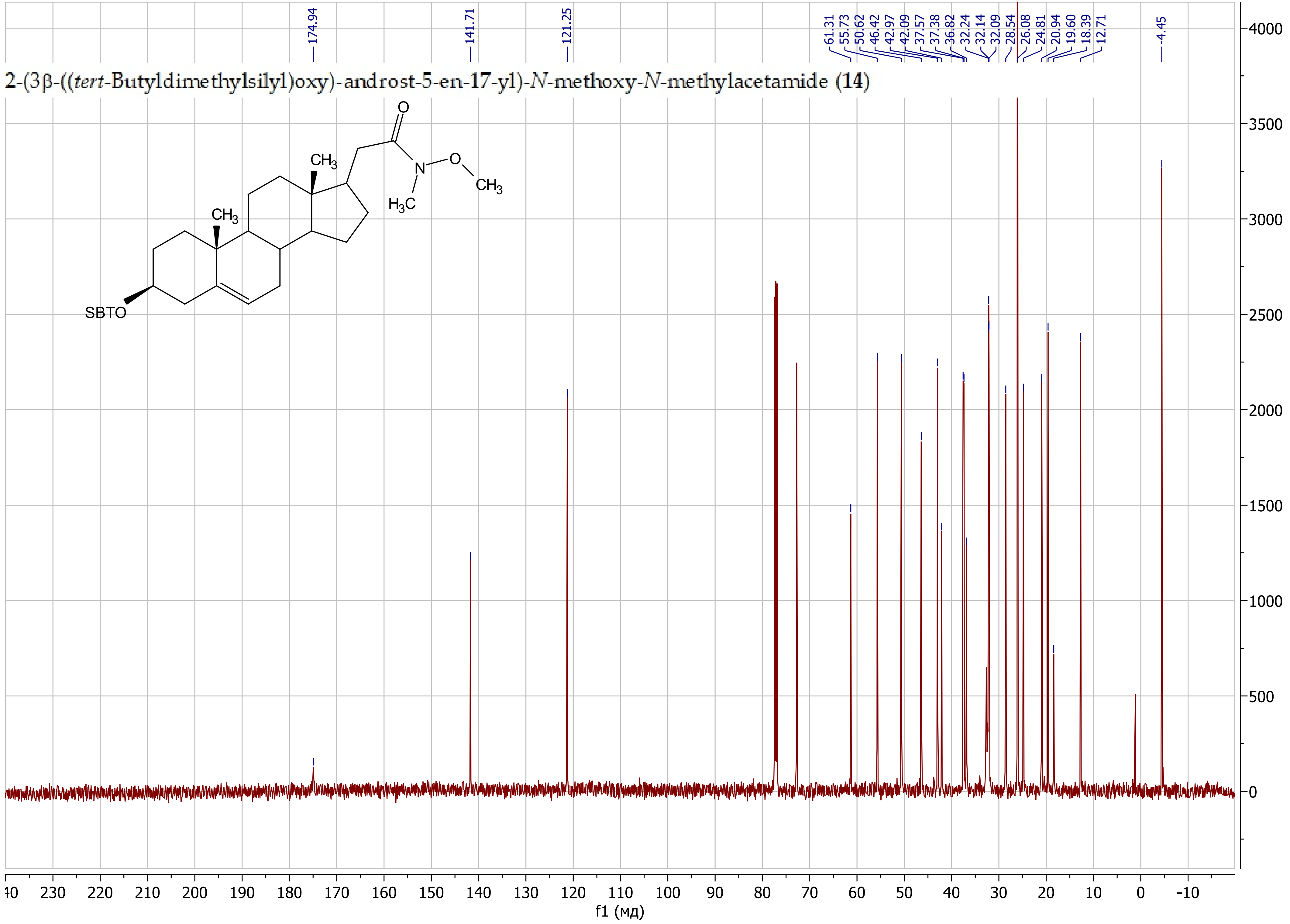

3β-((*tert*-Butyldimethylsilyl)oxy)-pregn-5-en-21-ol (**15**)

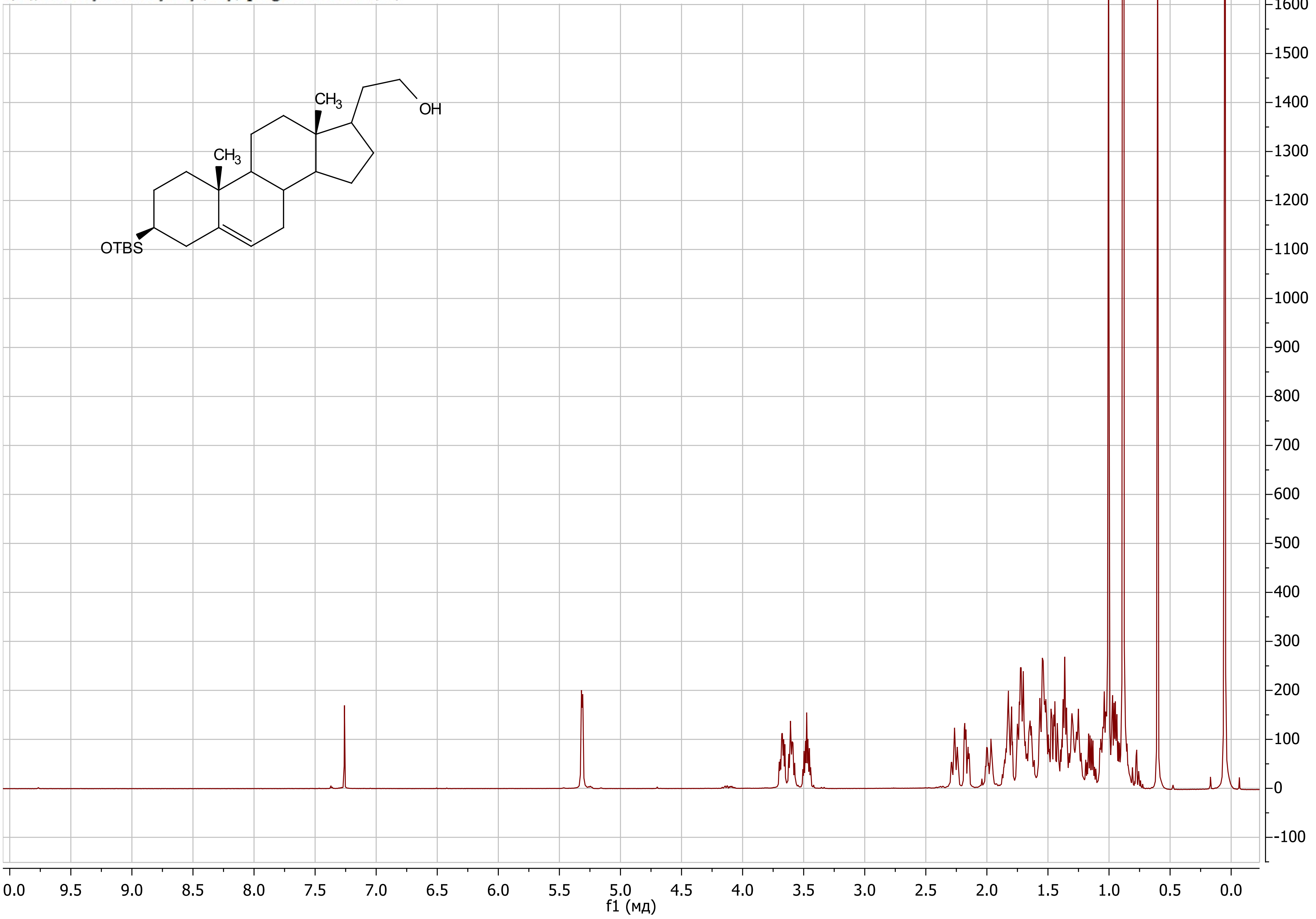

3β-((*tert*-Butyldimethylsilyl)oxy)-pregn-5-en-21-ol (**15**)

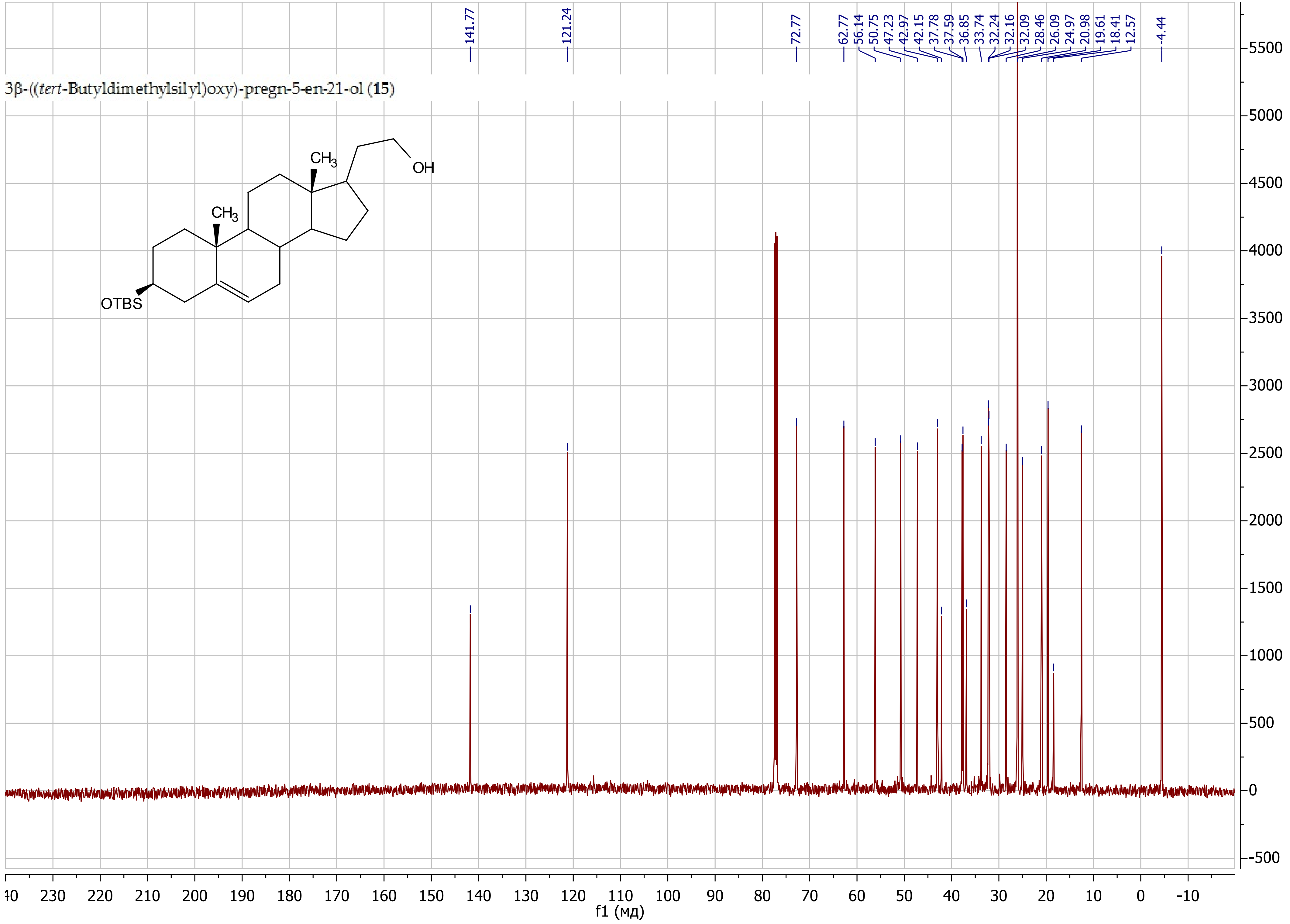

5-((*tert*-Butyldimethylsilyl)oxy)-1-((17*R*)-3β-((*tert*-butyldimethylsilyl)oxy)-androst-5-en-17-yl)pent-3-yn-2-one (**18**)

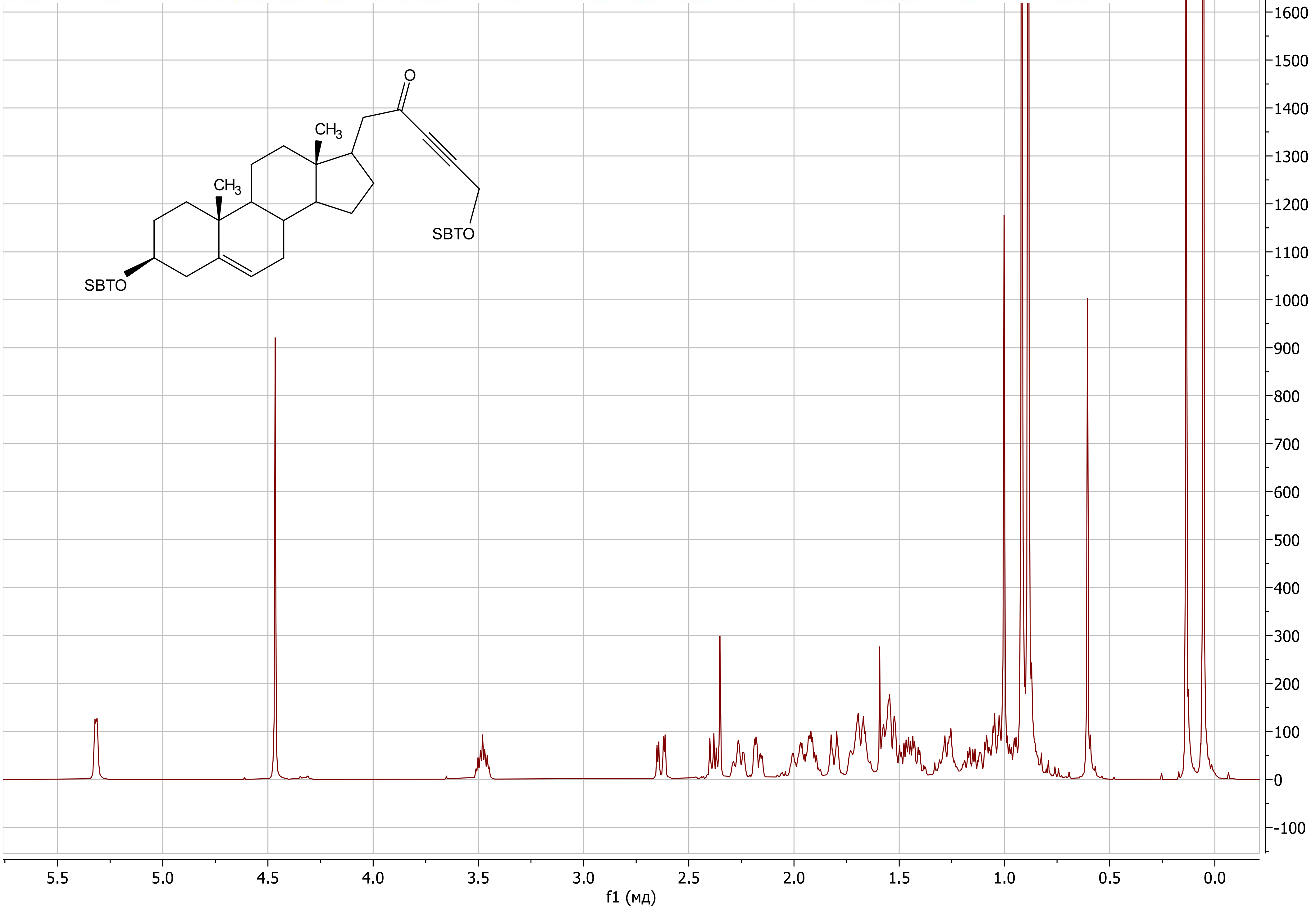

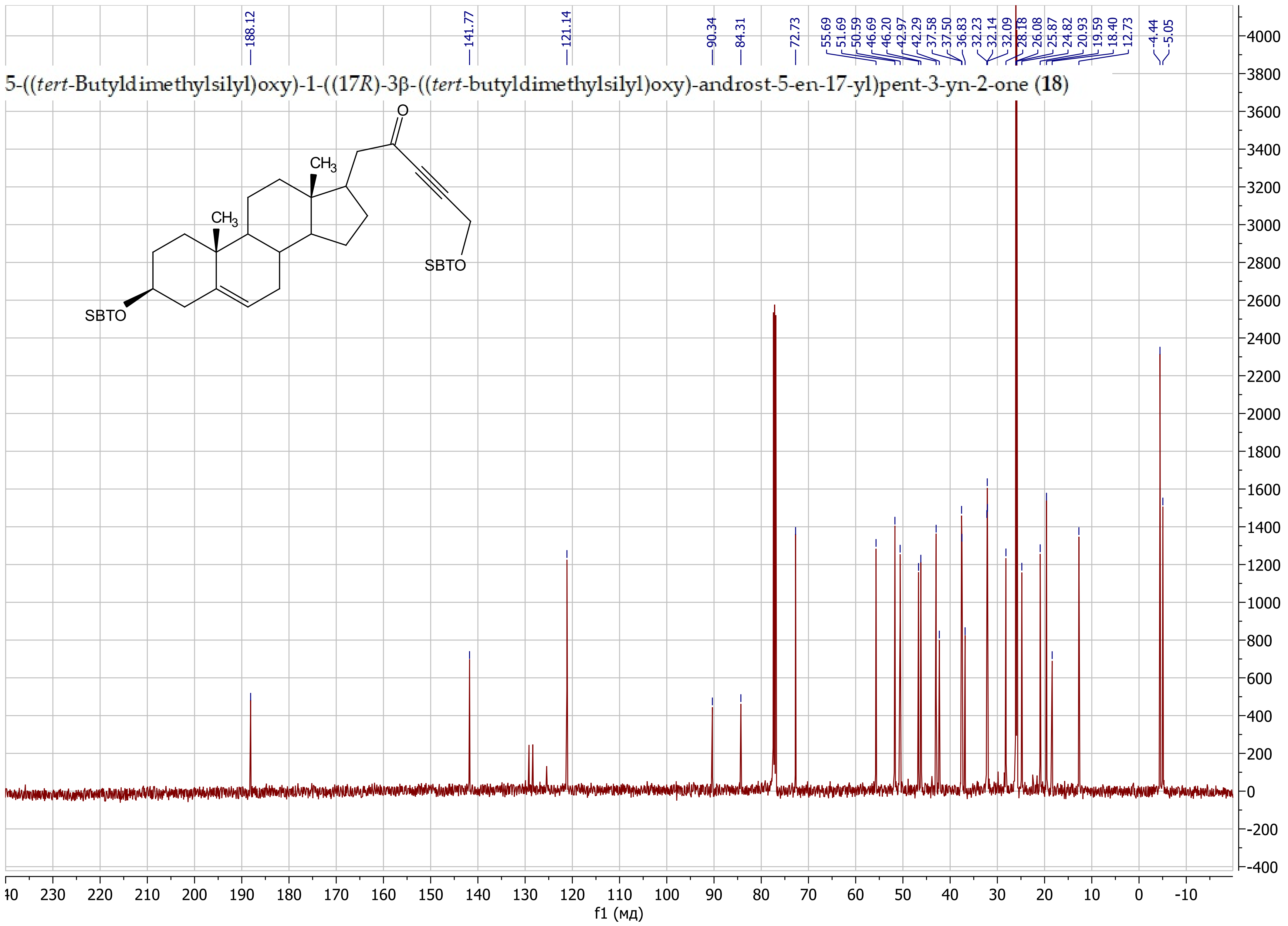
5-((tert-Butyldimethylsilyl)oxy)-1-((17R)-3β-((tert-butyldimethylsilyl)oxy)-androst-5-en-17-yl)pent-3-yn-2-one (18)
188.12
141.77
121.14
90.34
84.31
72.73
55.69
51.69
50.59
46.69
46.20
42.97
42.29
37.58
37.50
36.83
32.23
32.14
32.09
28.18
26.08
25.87
24.82
20.93
19.59
18.40
12.73
-4.44
-5.05
O
CH3
CH3
SBTO
SBTO
f1 (мд)

1-(((17*R*)-3β-((*tert*-Butyldimethylsilyl)oxy)-androst-5-en-17-yl)but-3-yn-2-one (**20a**)

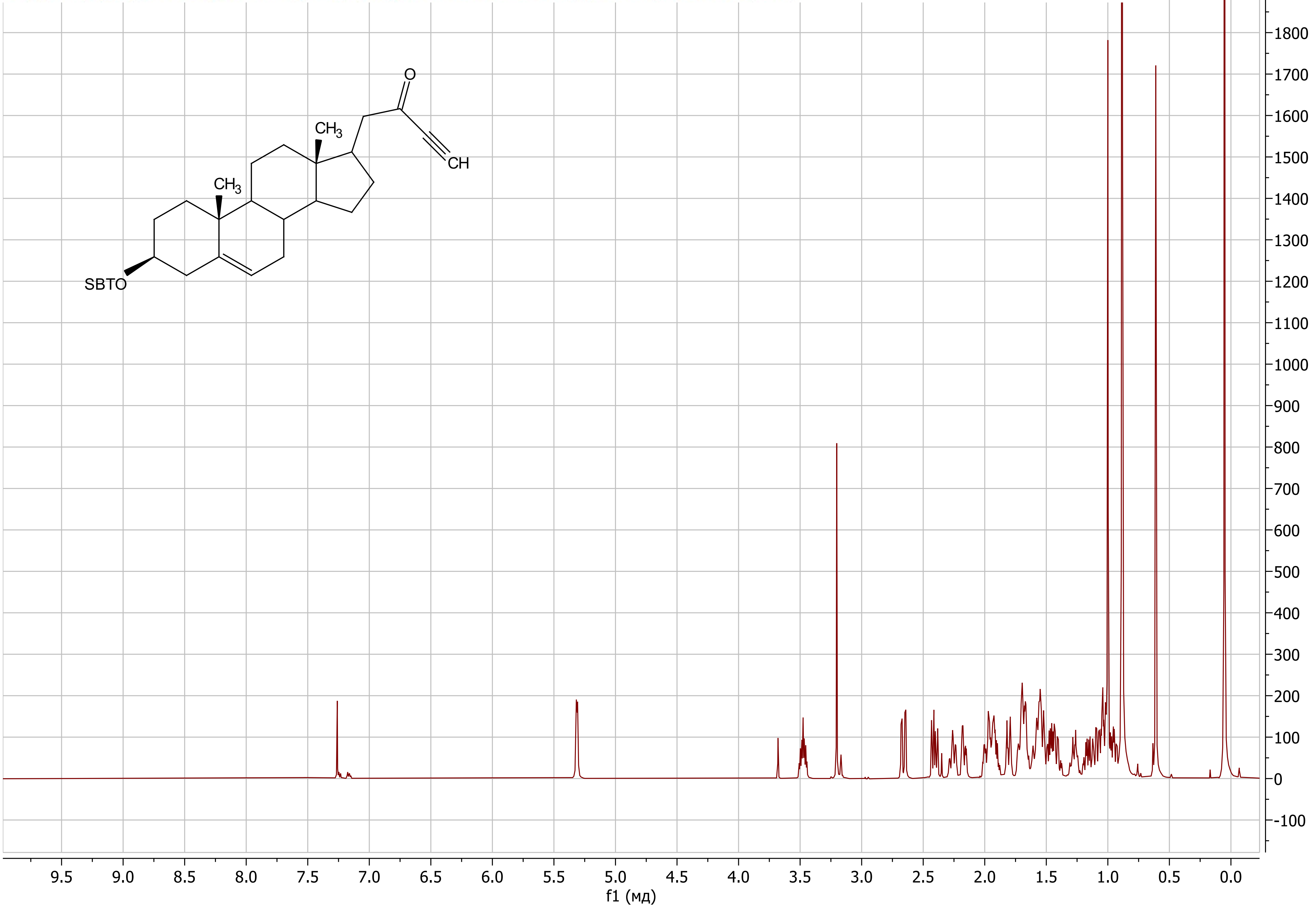

1-(((17*R*)-3β-((*tert*-Butyldimethylsilyl)oxy)-androst-5-en-17-yl)but-3-yn-2-one (**20a**)

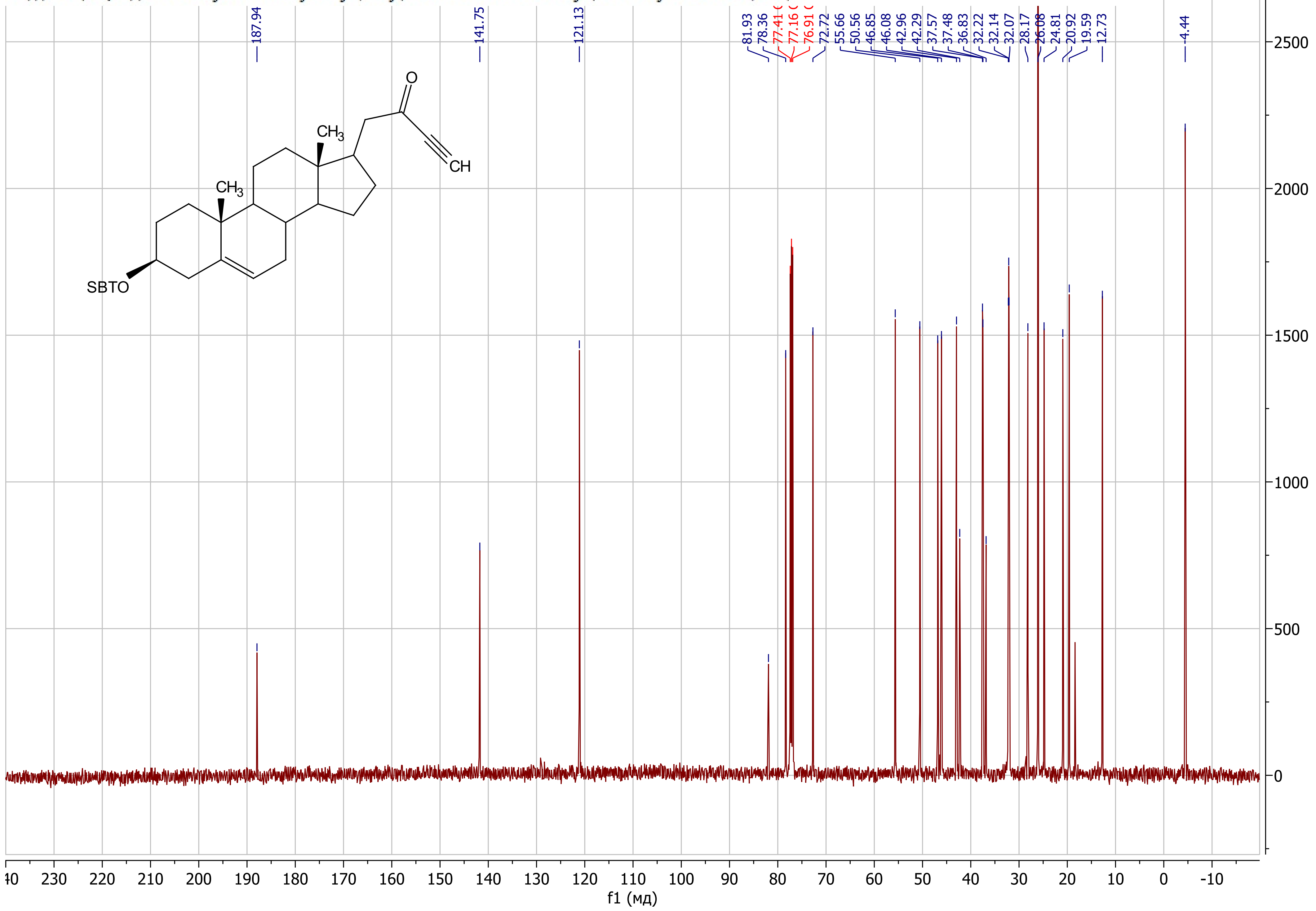

1-((17*R*)-3β-((*tert*-Butyldimethylsilyl)oxy)-androst-5-en-17-yl)-5-methylhex-3-yn-2-one (**20b**)

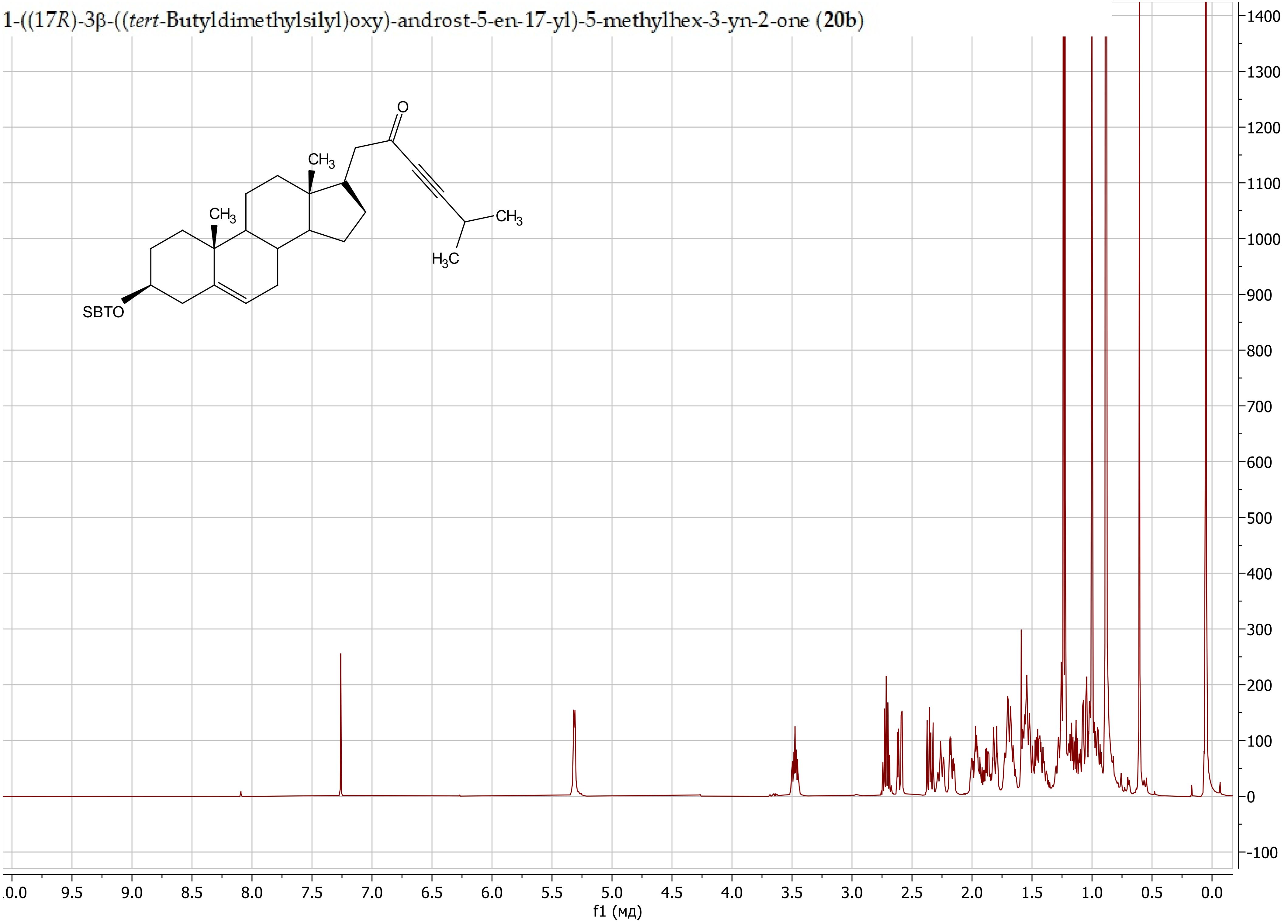

1-((17*R*)-3β-((*tert*-Butyldimethylsilyl)oxy)-androst-5-en-17-yl)-5-methylhex-3-yn-2-one (**20b**)

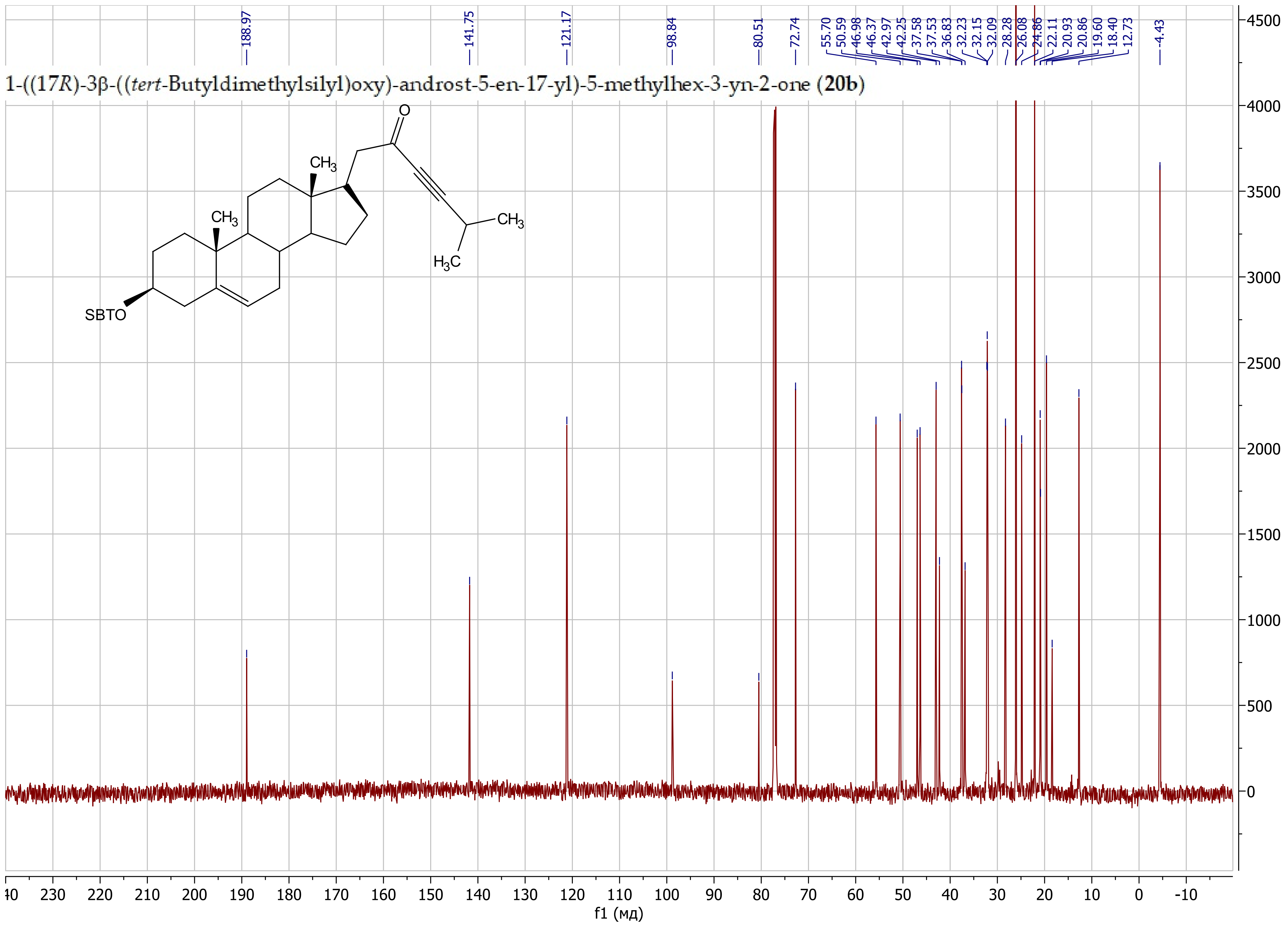

1-((17*R*)-3β-((*tert*-Butyldimethylsilyl)oxy)-androst-5-en-17-yl)-4-cyclopropylbut-3-yn-2-one (**20c**)

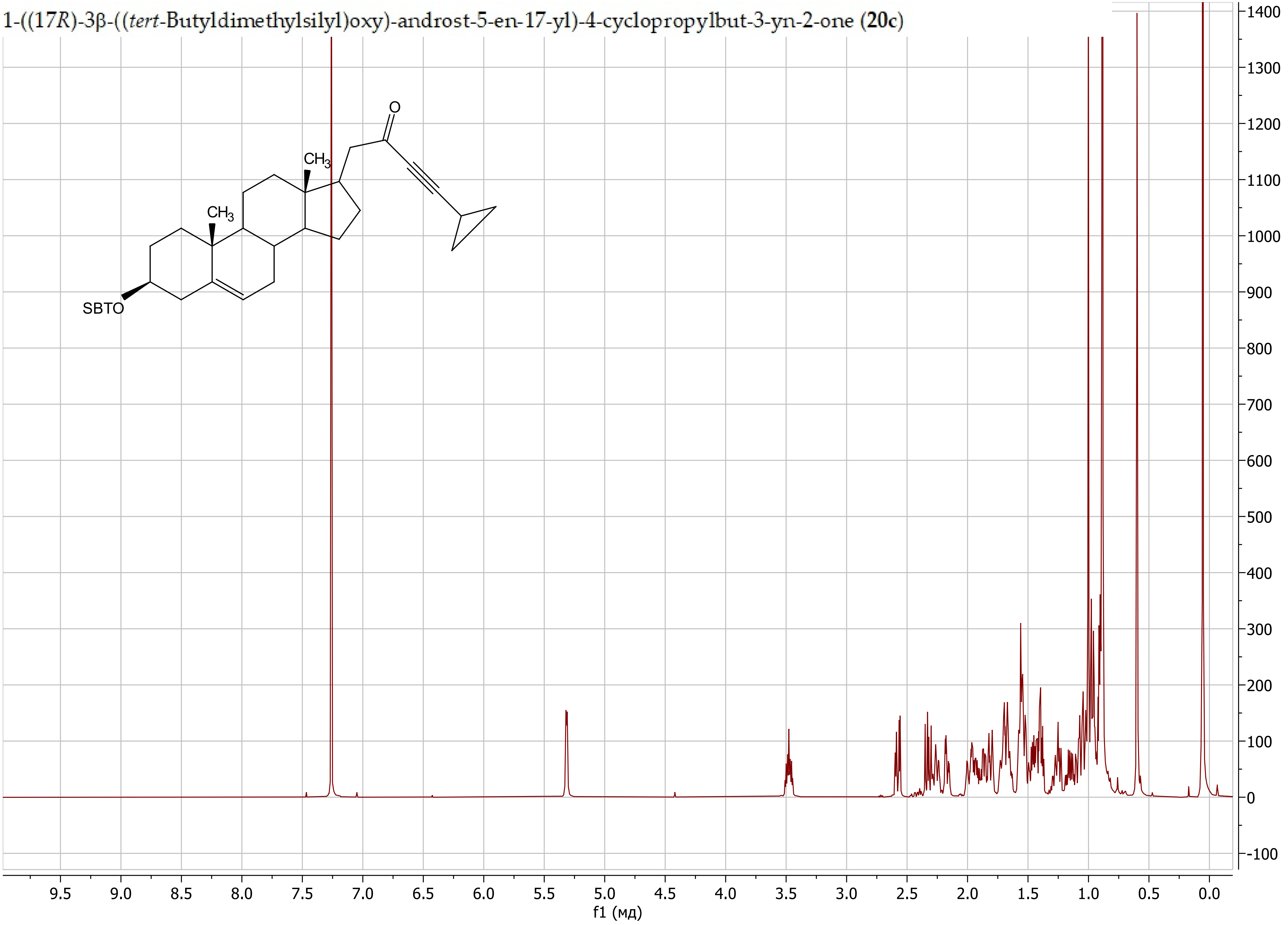

1-((17*R*)-3β-((*tert*-Butyldimethylsilyl)oxy)-androst-5-en-17-yl)-4-cyclopropylbut-3-yn-2-one (**20c**)

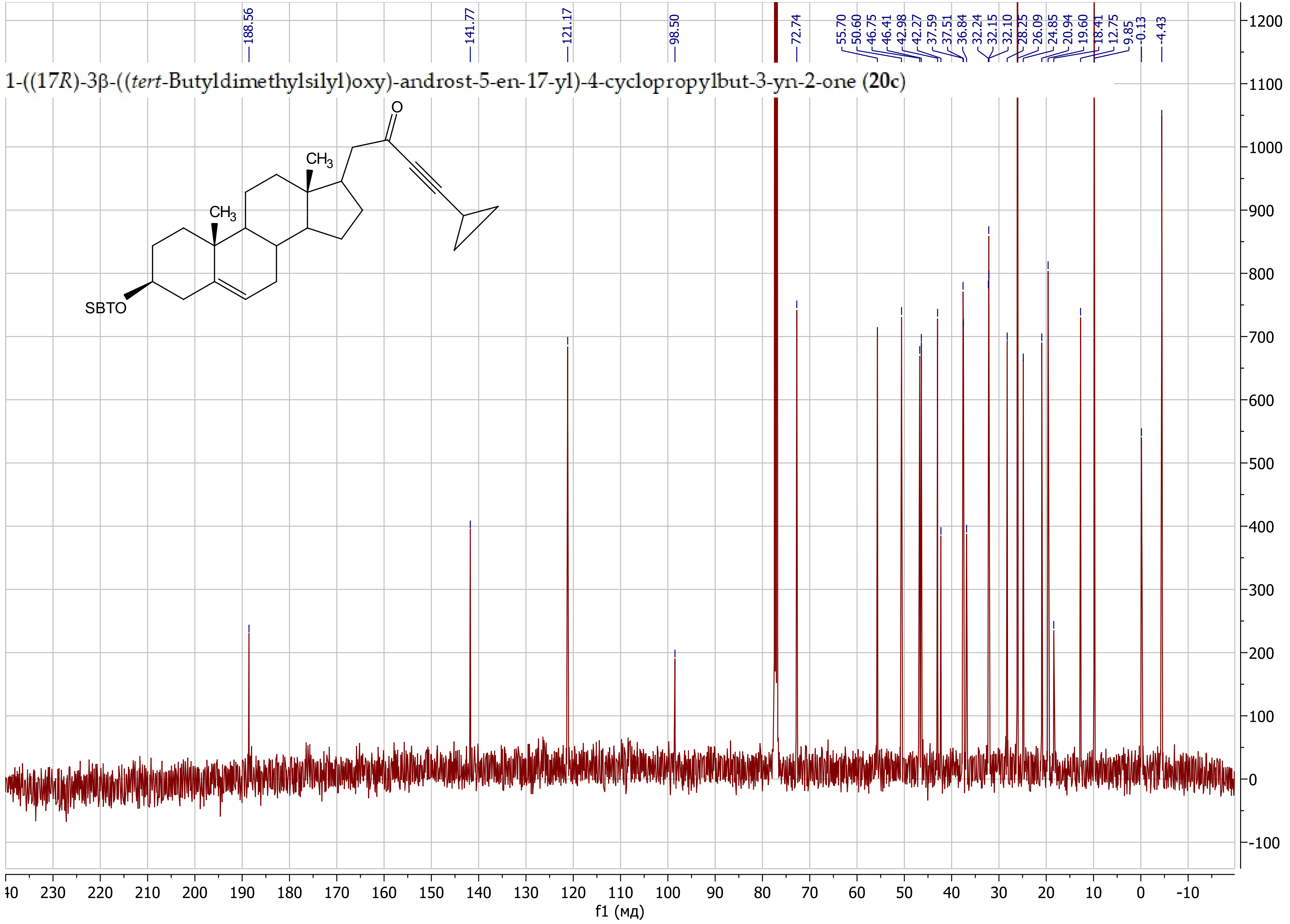

1-((17*R*)-3β-((*tert*-Butyldimethylsilyl)oxy)-androst-5-en-17-yl)oct-3-yn-2-one (**20d**)

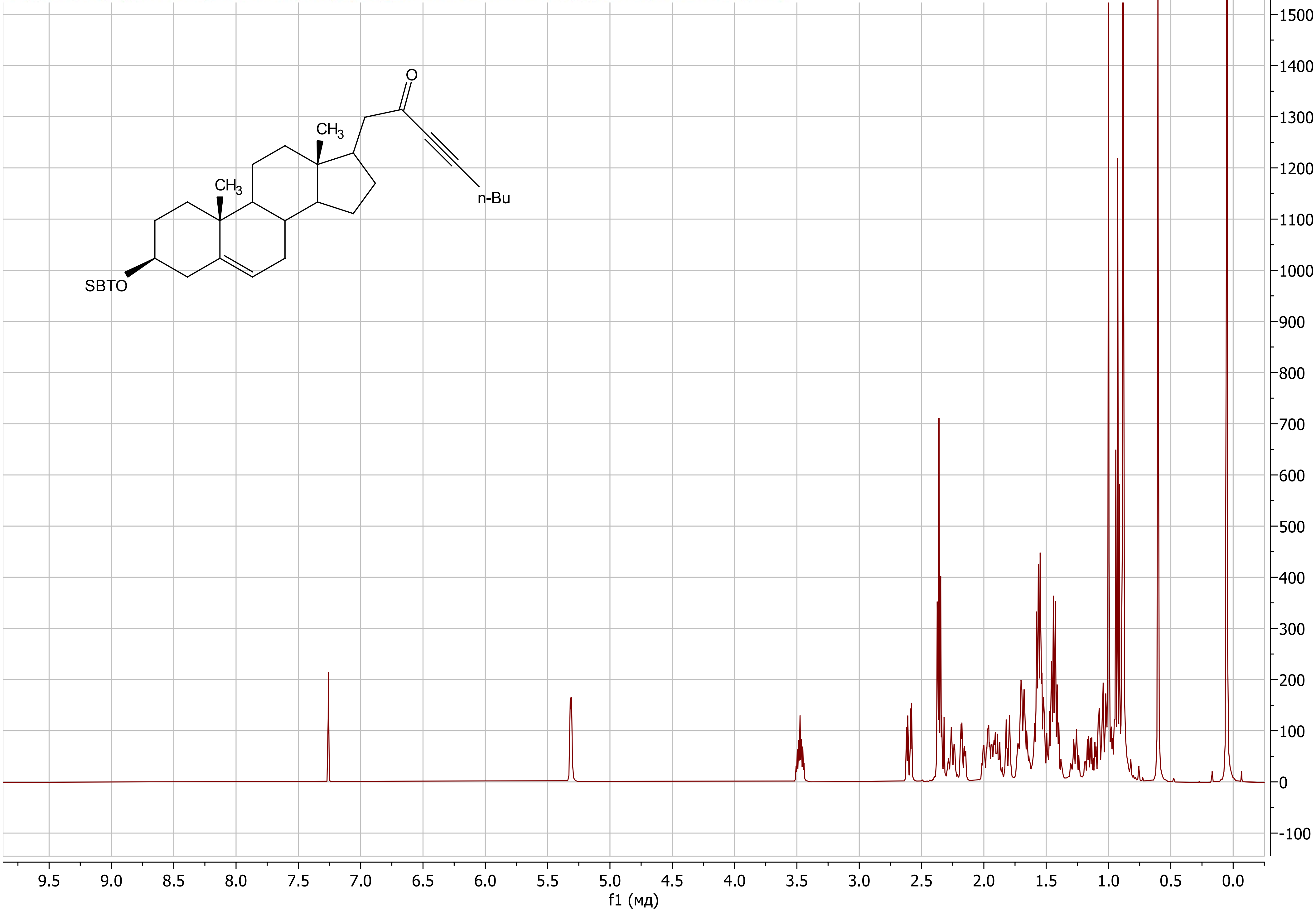

1-((17*R*)-3β-((*tert*-Butyldimethylsilyl)oxy)-androst-5-en-17-yl)oct-3-yn-2-one (**20d**)

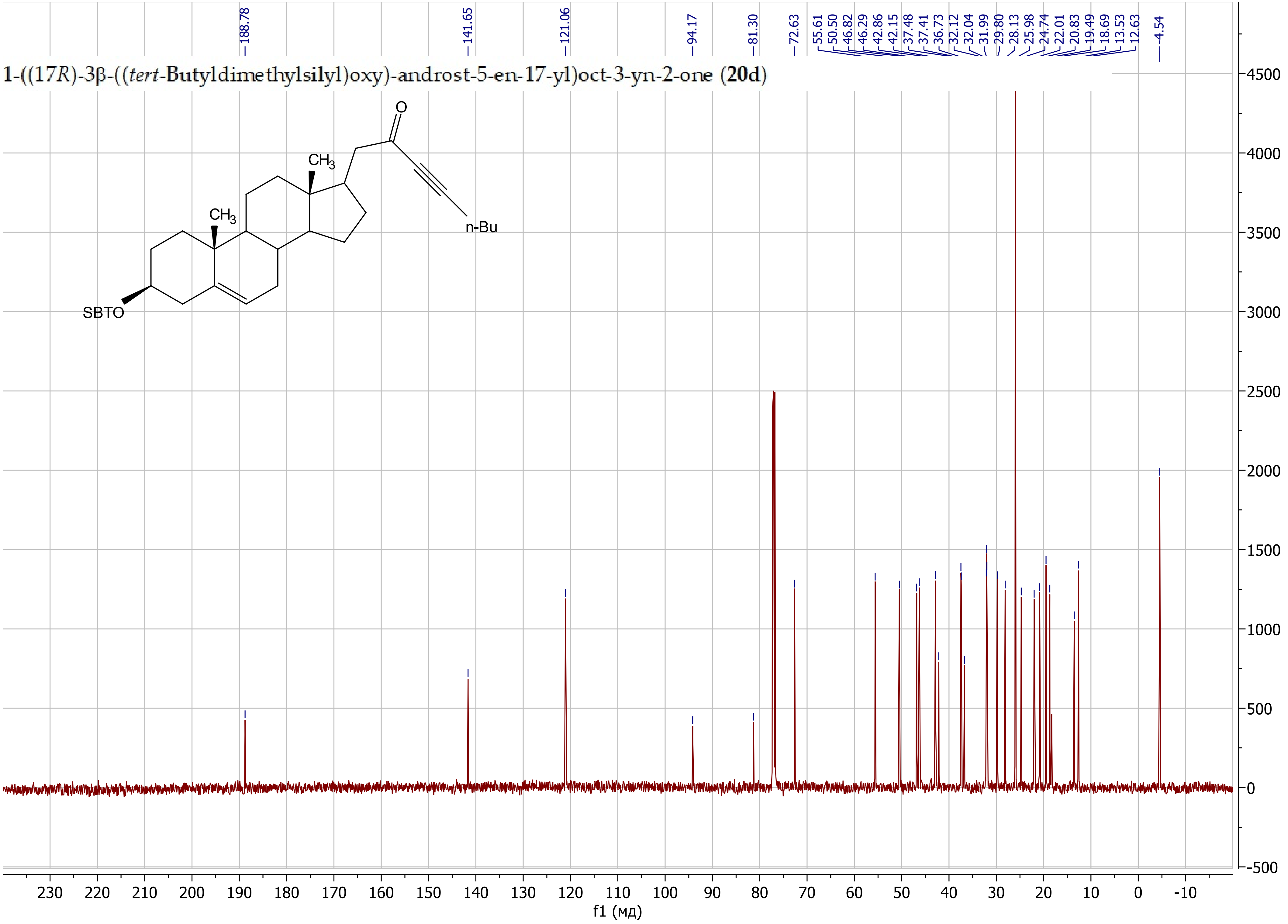

1-((17*R*)-3β-((*tert*-Butyldimethylsilyl)oxy)-androst-5-en-17-yl)-4-phenylbut-3-yn-2-one (**20e**)

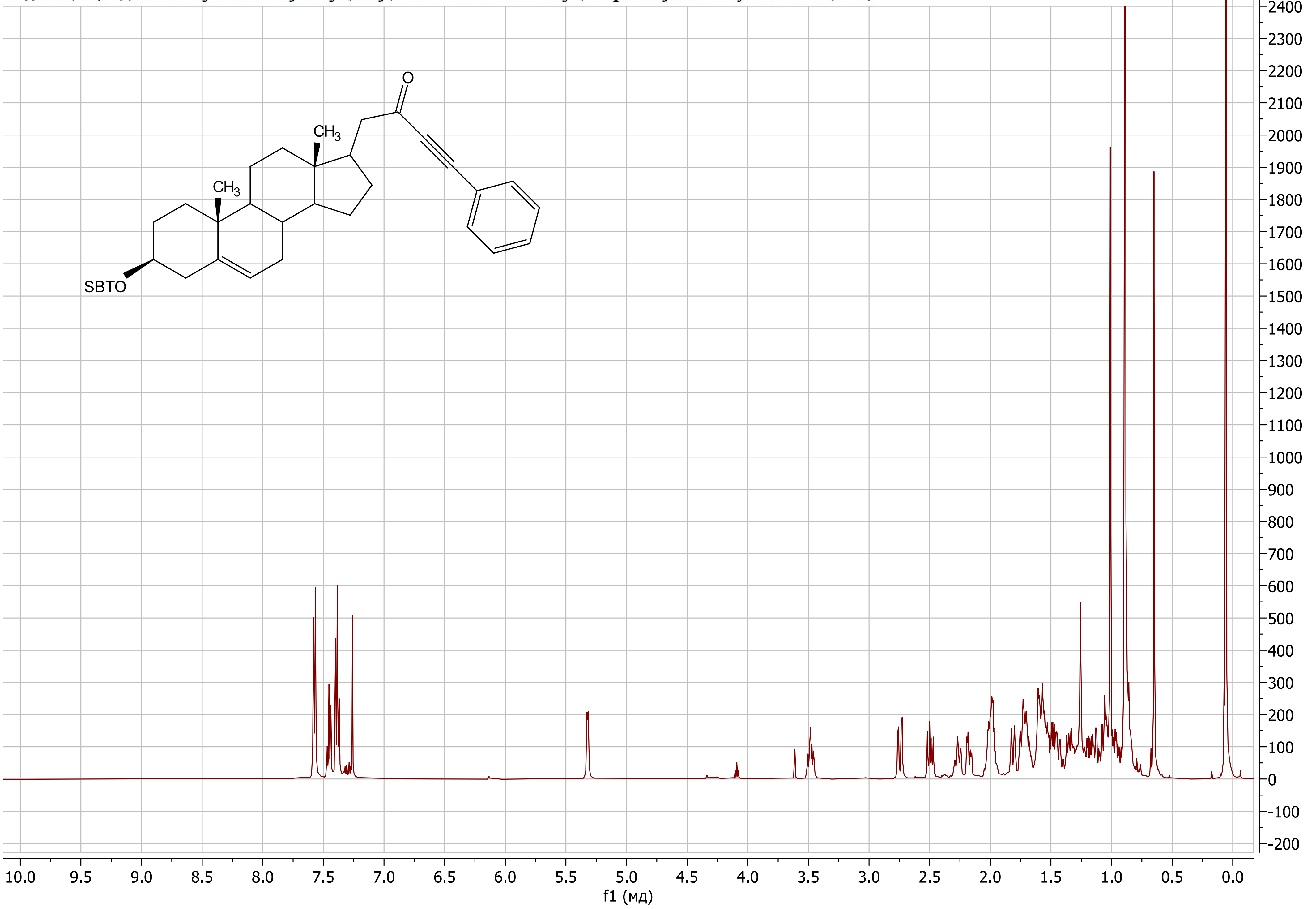

1-((17*R*)-3β-((*tert*-Butyldimethylsilyl)oxy)-androst-5-en-17-yl)-4-phenylbut-3-yn-2-one (**20e**)

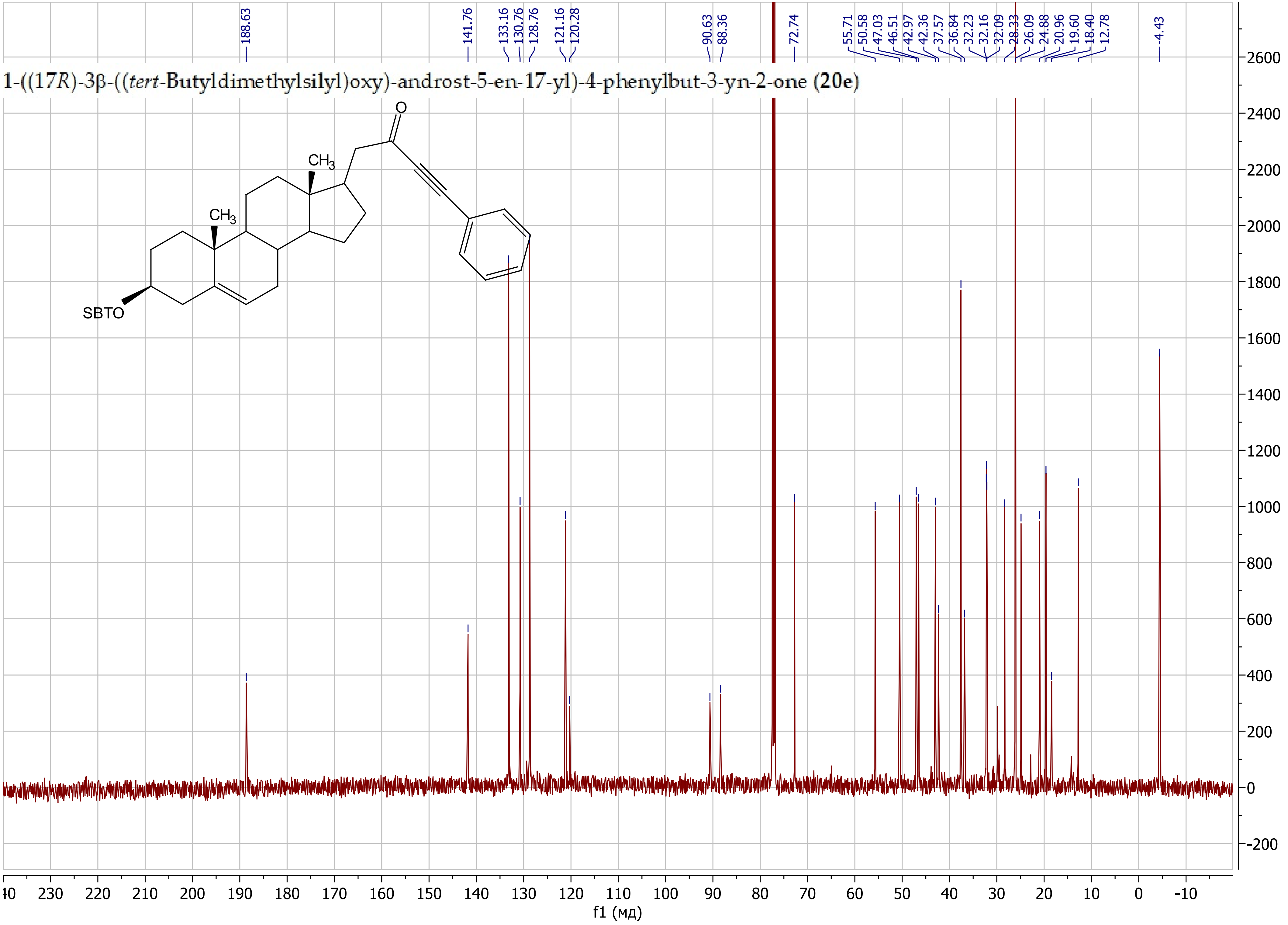

1-((17*R*)-3β-((*tert*-Butyldimethylsilyl)oxy)-androst-5-en-17-yl)-4-(pyridin-3-yl)but-3-yn-2-one (**20f**)

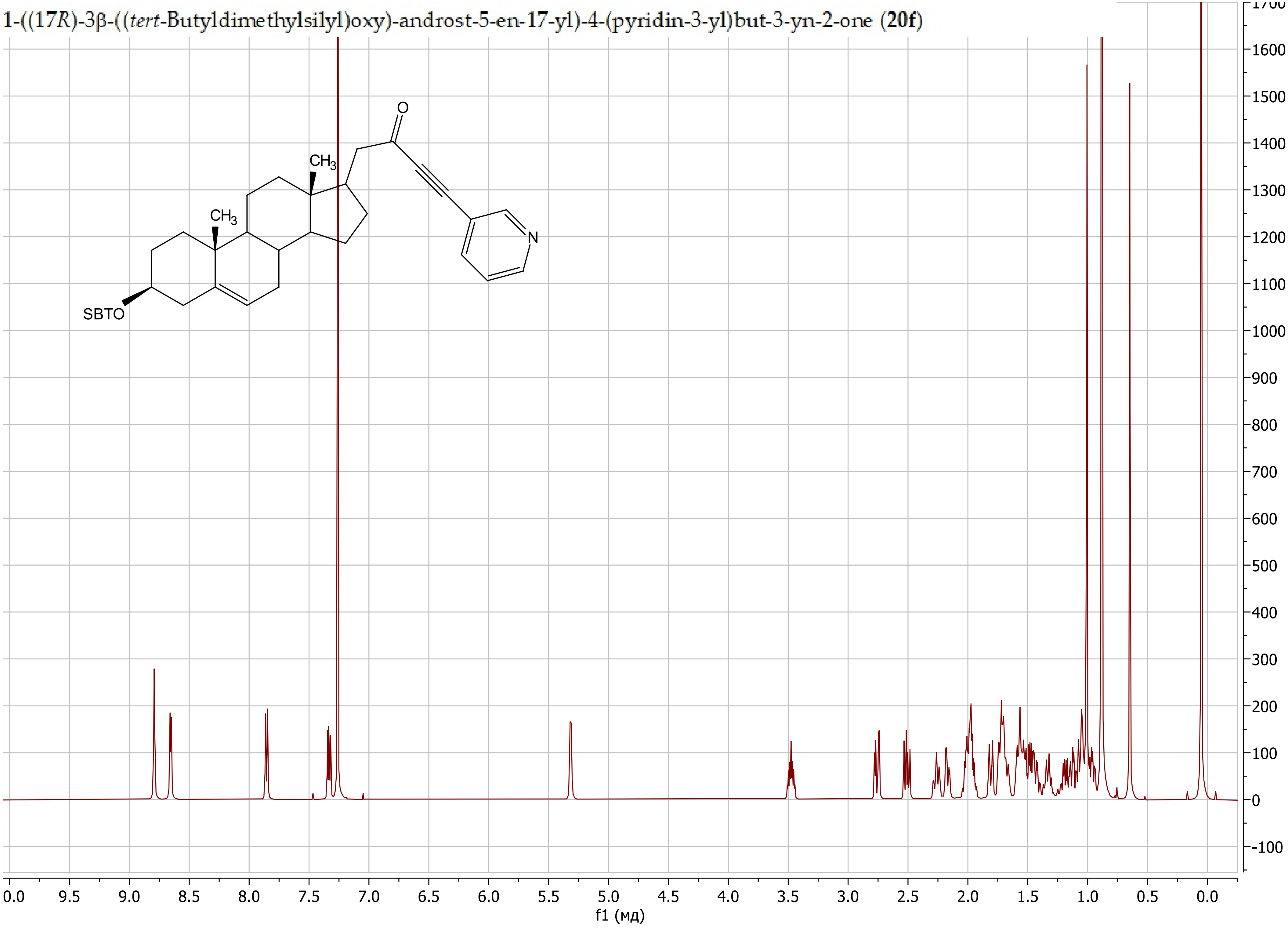

1-((17*R*)-3β-((*tert*-Butyldimethylsilyl)oxy)-androst-5-en-17-yl)-4-(pyridin-3-yl)but-3-yn-2-one (**20f**)

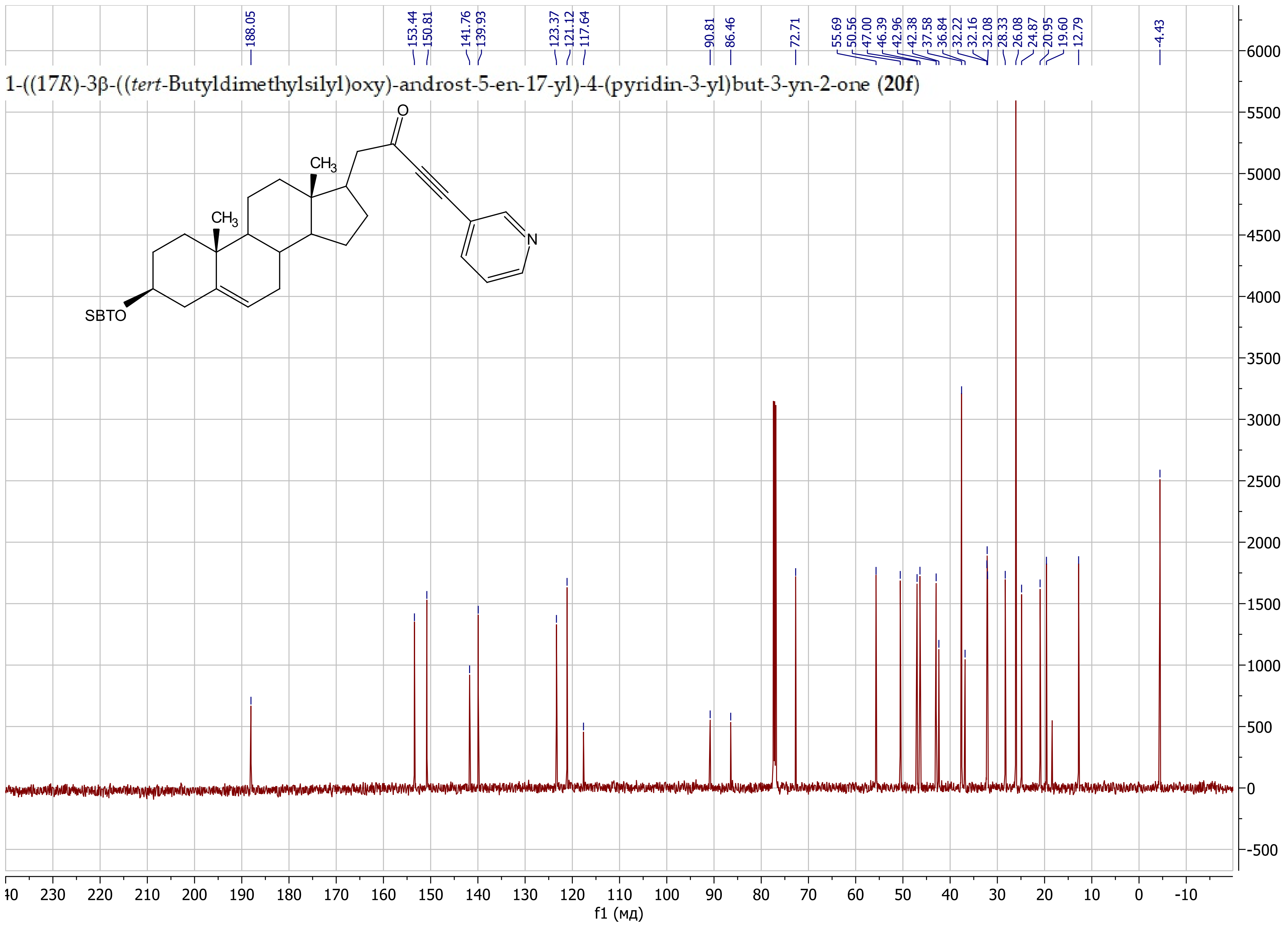

1-((17*R*)-3β-((*tert*-Butyldimethylsilyl)oxy)-androst-5-en-17-yl)-4-(2-fluorophenyl)but-3-yn-2-one (**20g**)

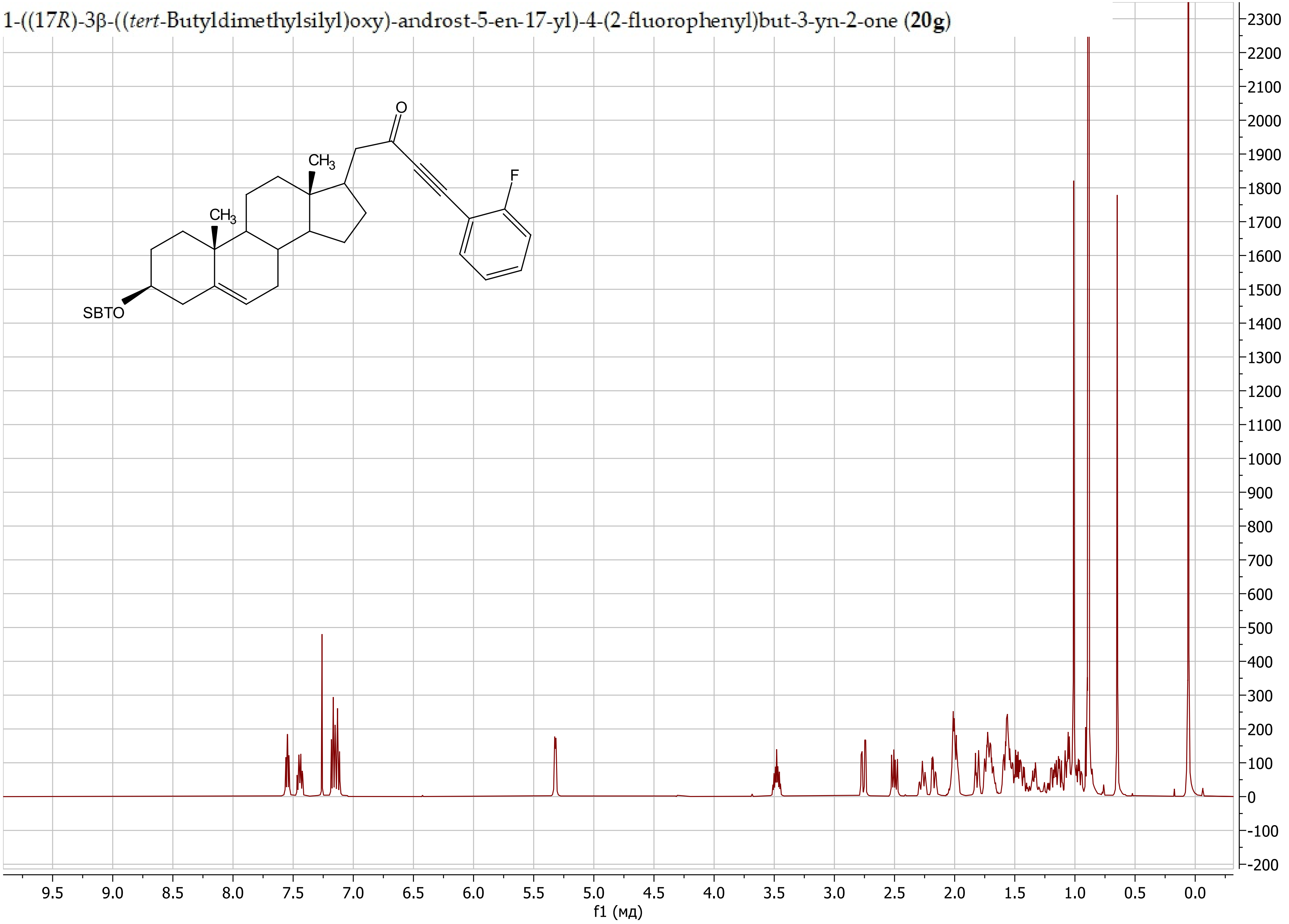

1-((17*R*)-3β-((*tert*-Butyldimethylsilyl)oxy)-androst-5-en-17-yl)-4-(2-fluorophenyl)but-3-yn-2-one (**20g**)

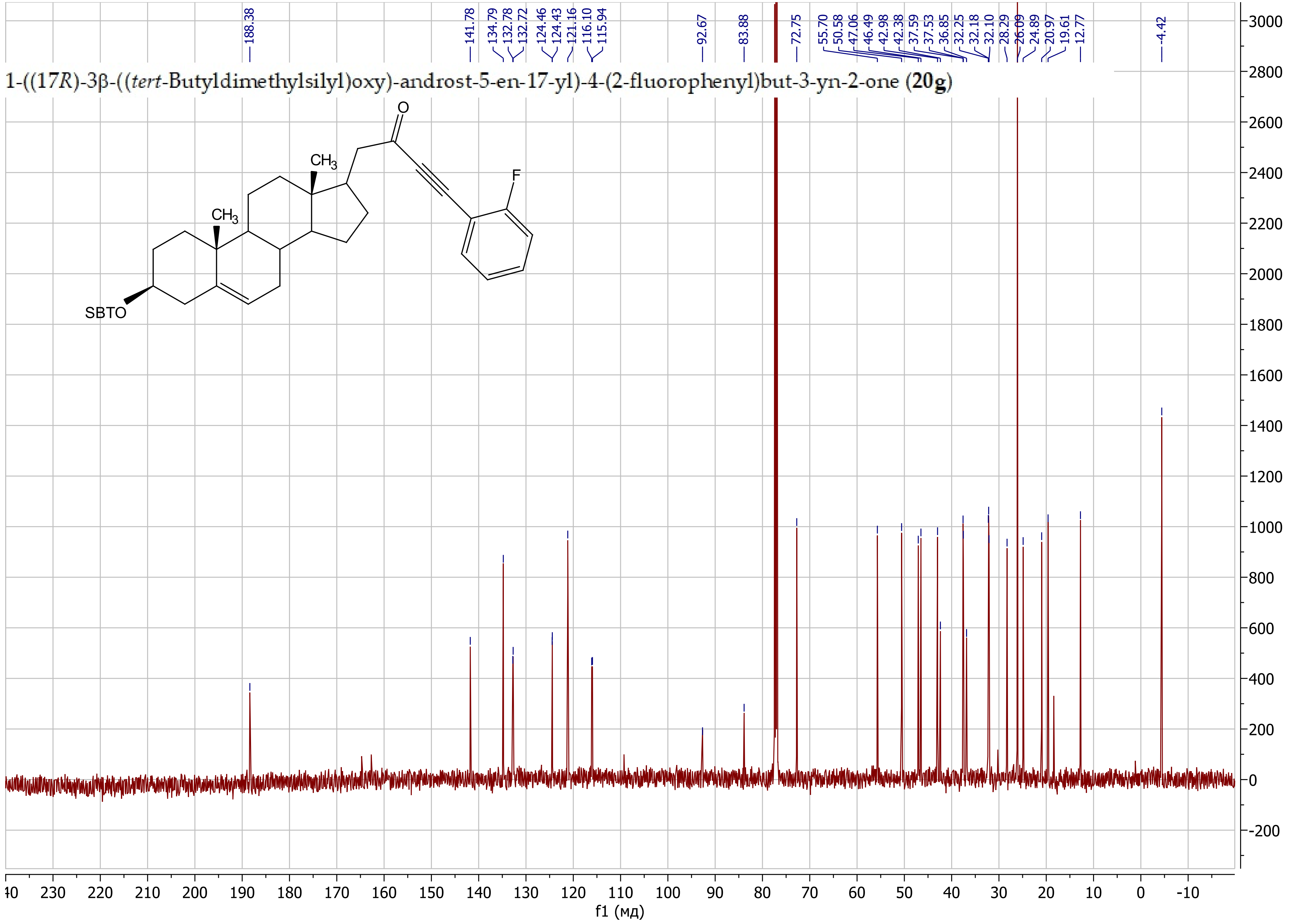

1-((17*R*)-3β-((*tert*-Butyldimethylsilyl)oxy)-androst-5-en-17-yl)-5-methyl-5-((tetrahydro-2*H*-pyran-2-yl)oxy)hex-3-yn-2-one (**20h**)

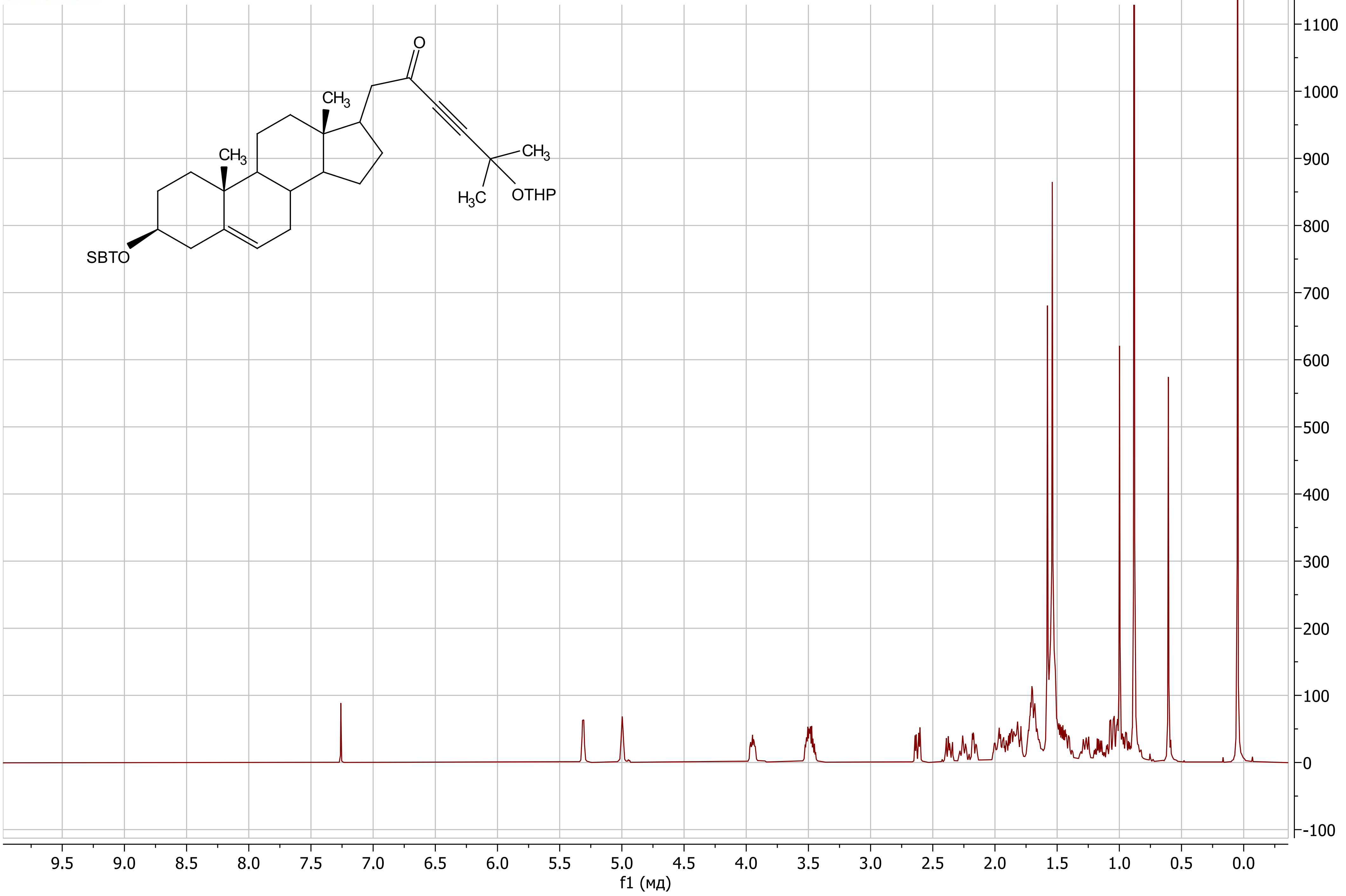

1-((17*R*)-3β-((*tert*-Butyldimethylsilyl)oxy)-androst-5-en-17-yl)-5-methyl-5-((tetrahydro-2*H*-pyran-2-yl)oxy)hex-3-yn-2-one (**20h**)

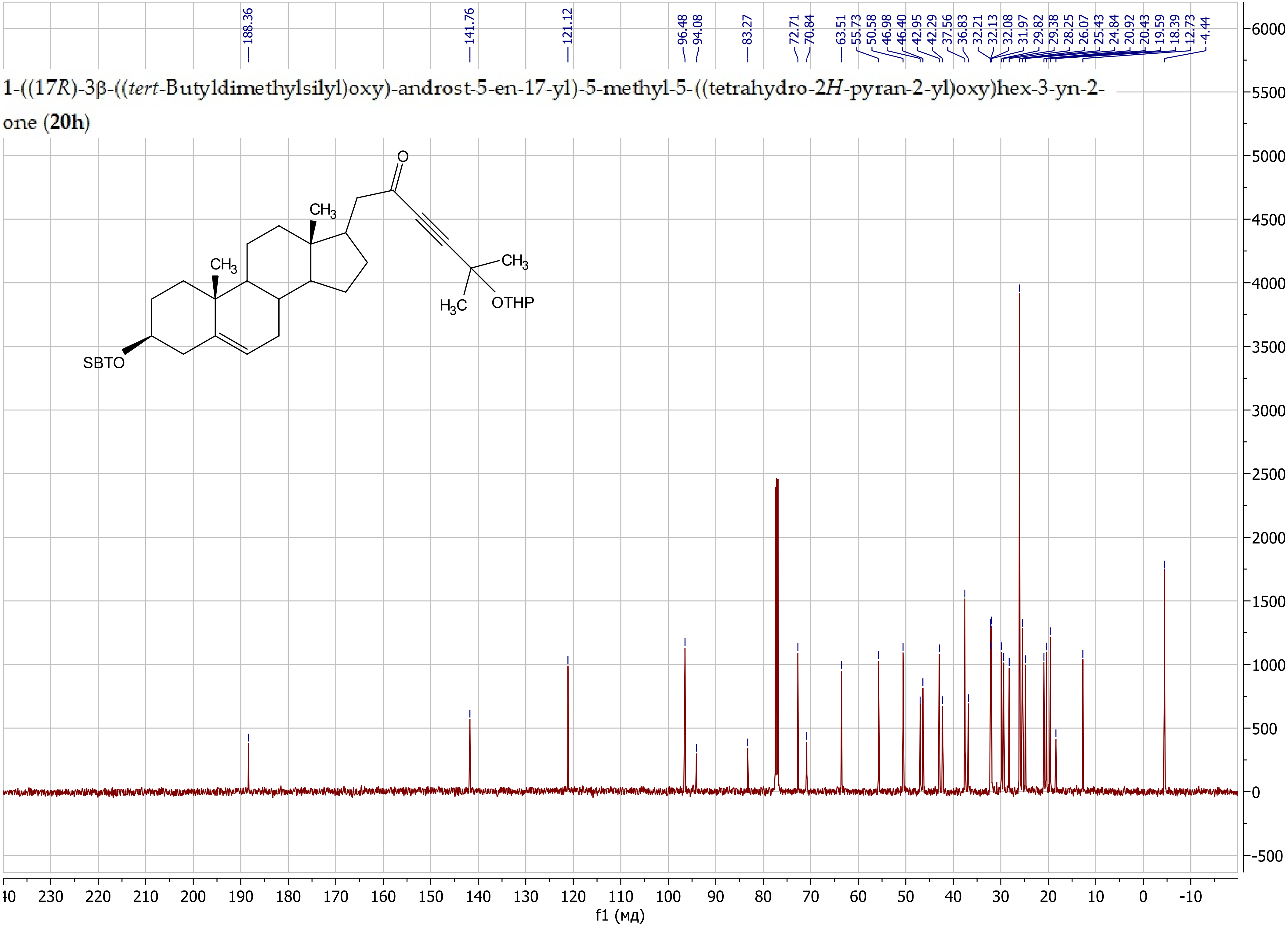

1-((17*R*)-3β-((*tert*-Butyldimethylsilyl)oxy)-androst-5-en-17-yl)-5-((tetrahydro-2*H*-pyran-2-yl)oxy)pent-3-yn-2-one (**20i**)

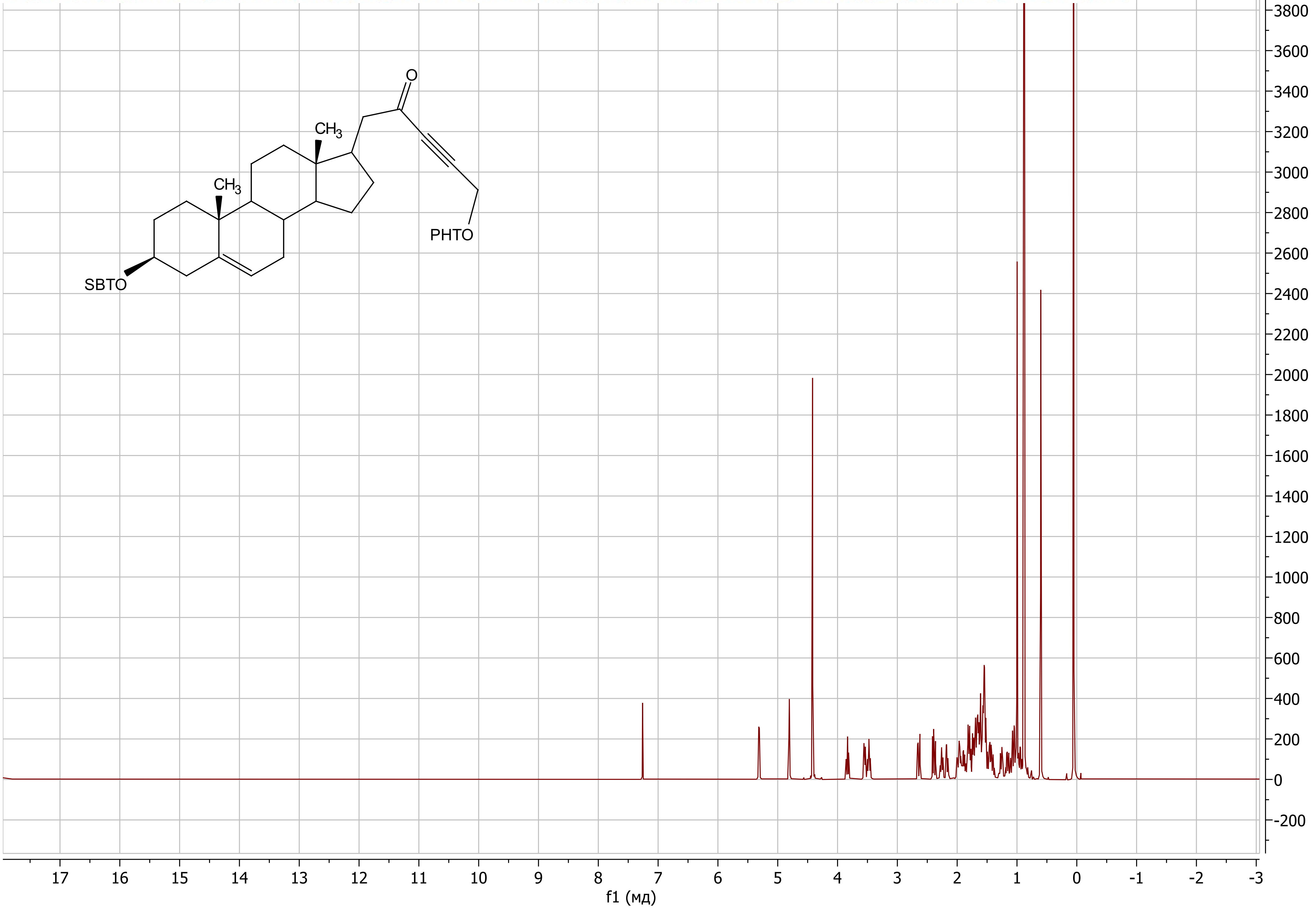

1-((17*R*)-3β-((*tert*-Butyldimethylsilyl)oxy)-androst-5-en-17-yl)-5-((tetrahydro-2*H*-pyran-2-yl)oxy)pent-3-yn-2-one (**20i**)

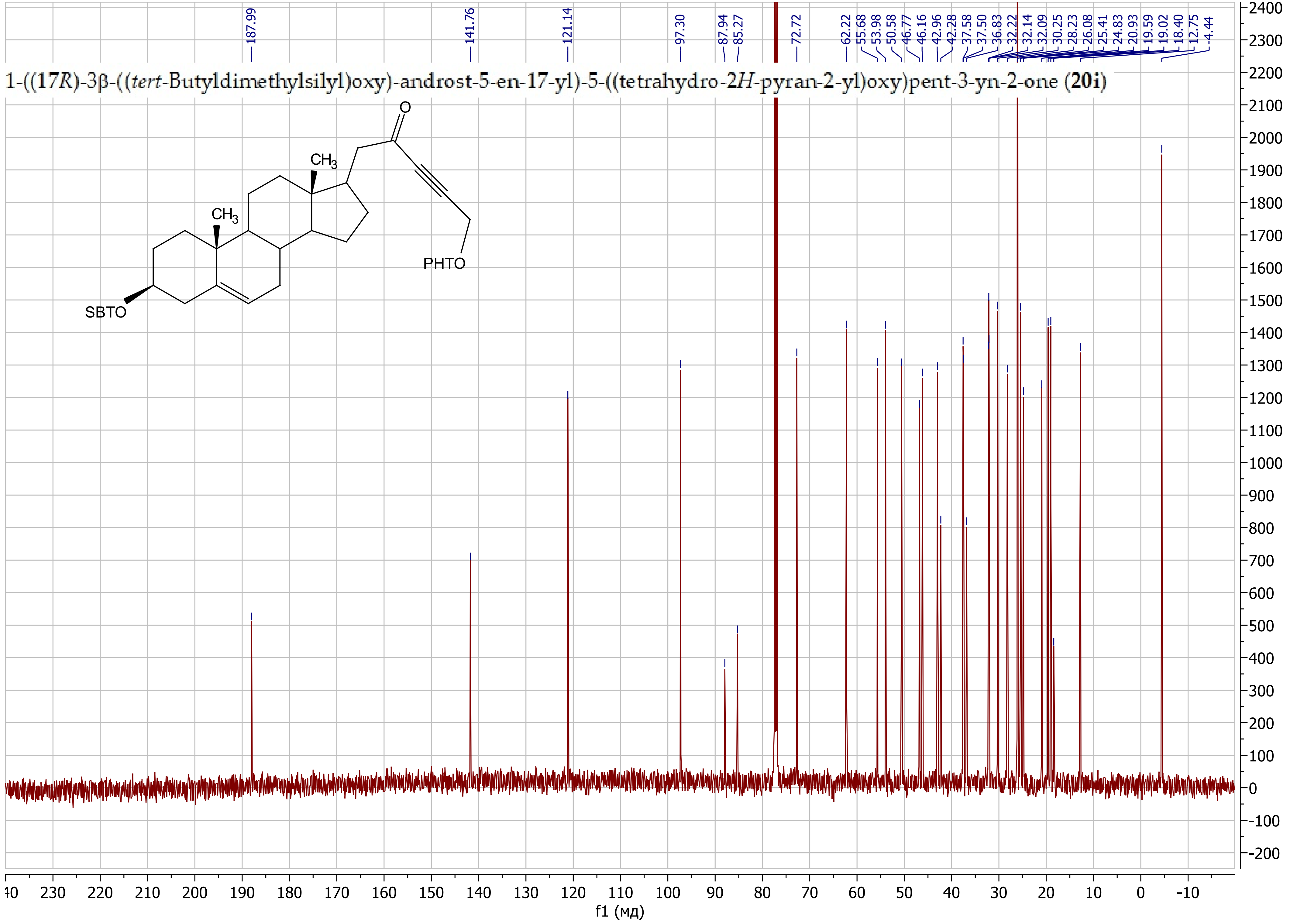

5-(((17*R*)-3β-((*tert*-Butyldimethylsilyl)oxy)-androst-5-en-17-yl)-4,5-dihydroisoxazol-5-ol (**22a**)

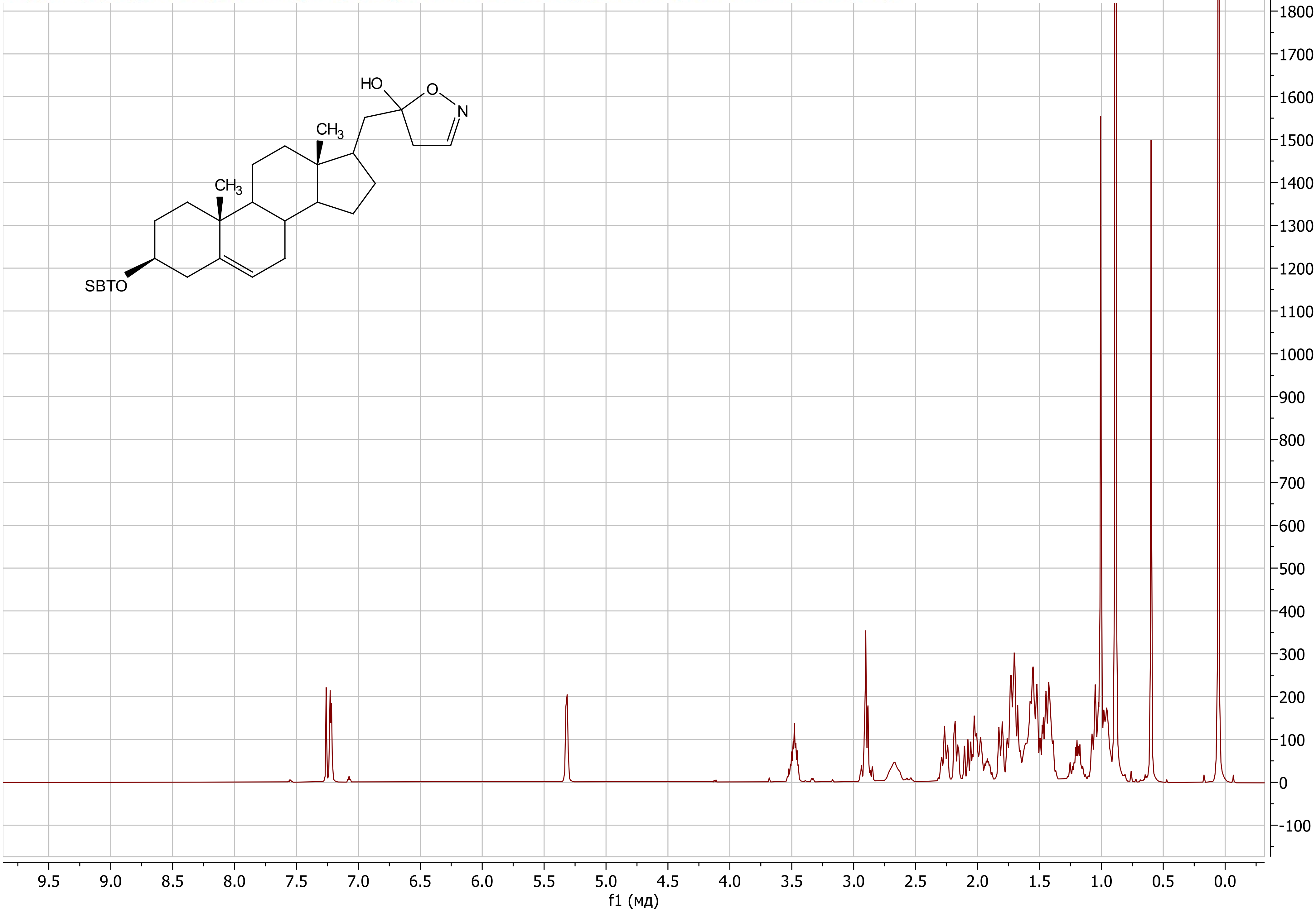

5-(((17*R*)-3β-((*tert*-Butyldimethylsilyl)oxy)-androst-5-en-17-yl)-4,5-dihydroisoxazol-5-ol (**22a**)

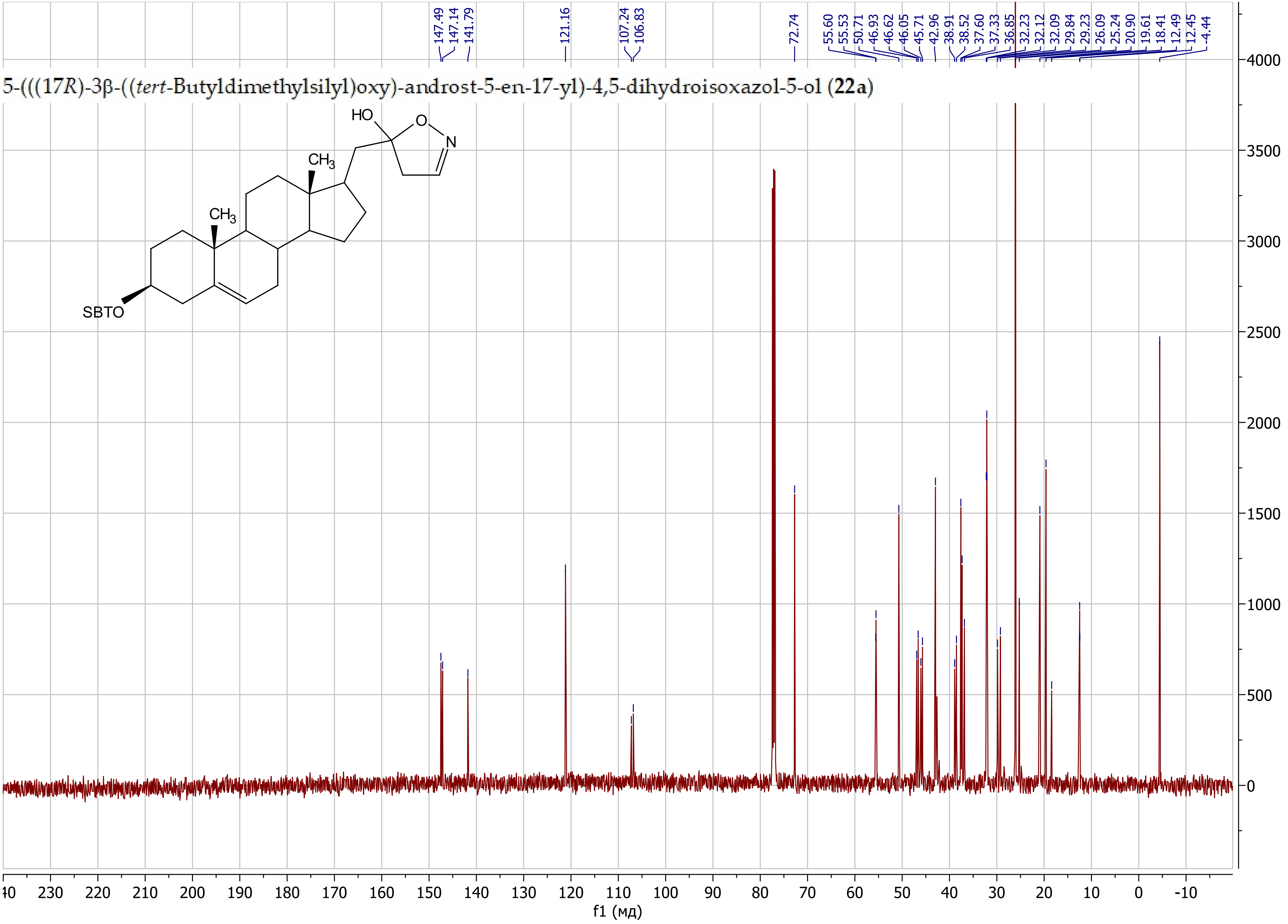

5-(((17*R*)-3β-((*tert*-Butyldimethylsilyl)oxy)-androst-5-en-17-yl)-3-isopropyl-4,5-dihydroisoxazol-5-ol (**22b**)

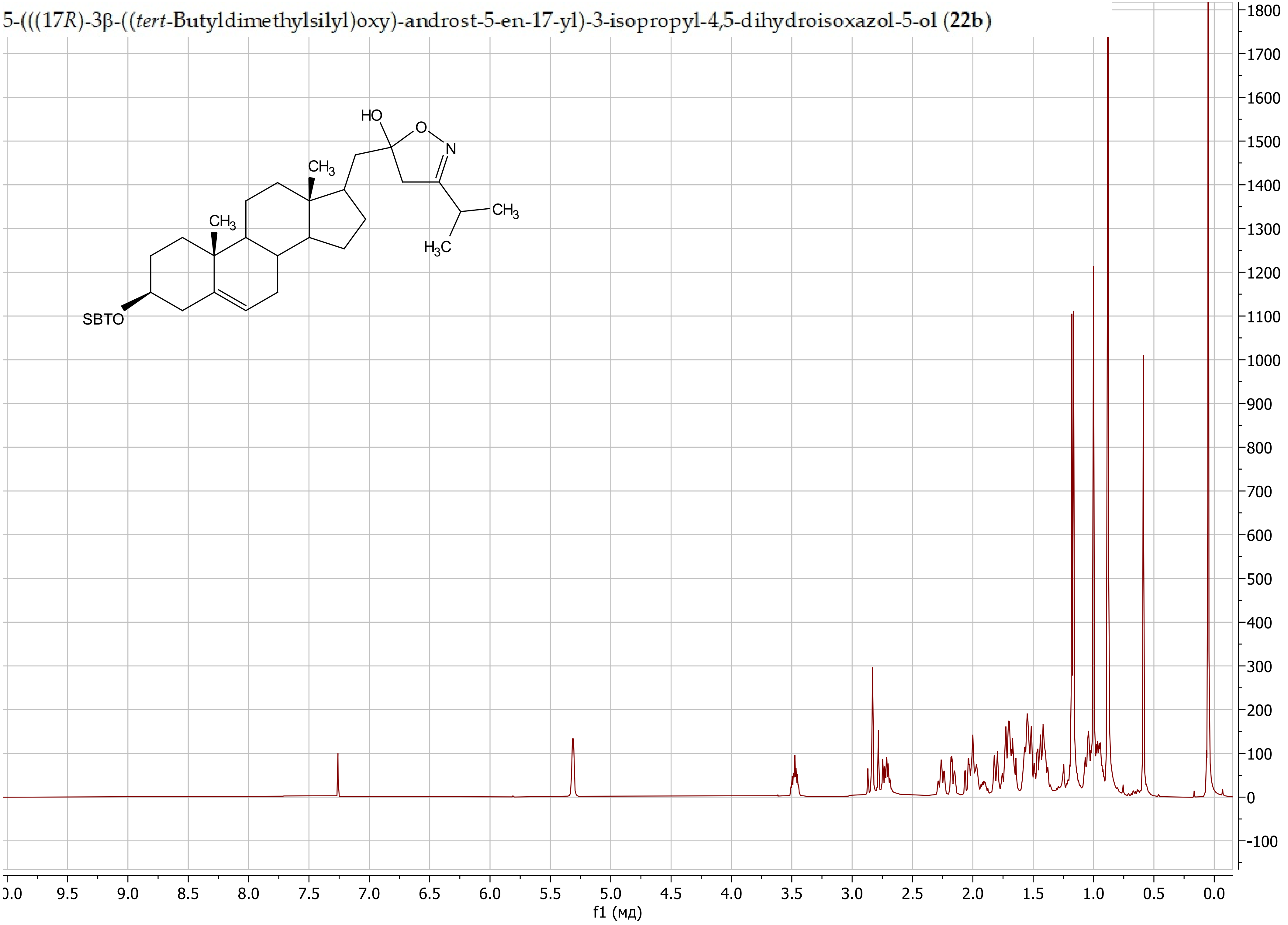

5-(((17*R*)-3β-((*tert*-Butyldimethylsilyl)oxy)-androst-5-en-17-yl)-3-isopropyl-4,5-dihydroisoxazol-5-ol (**22b**)

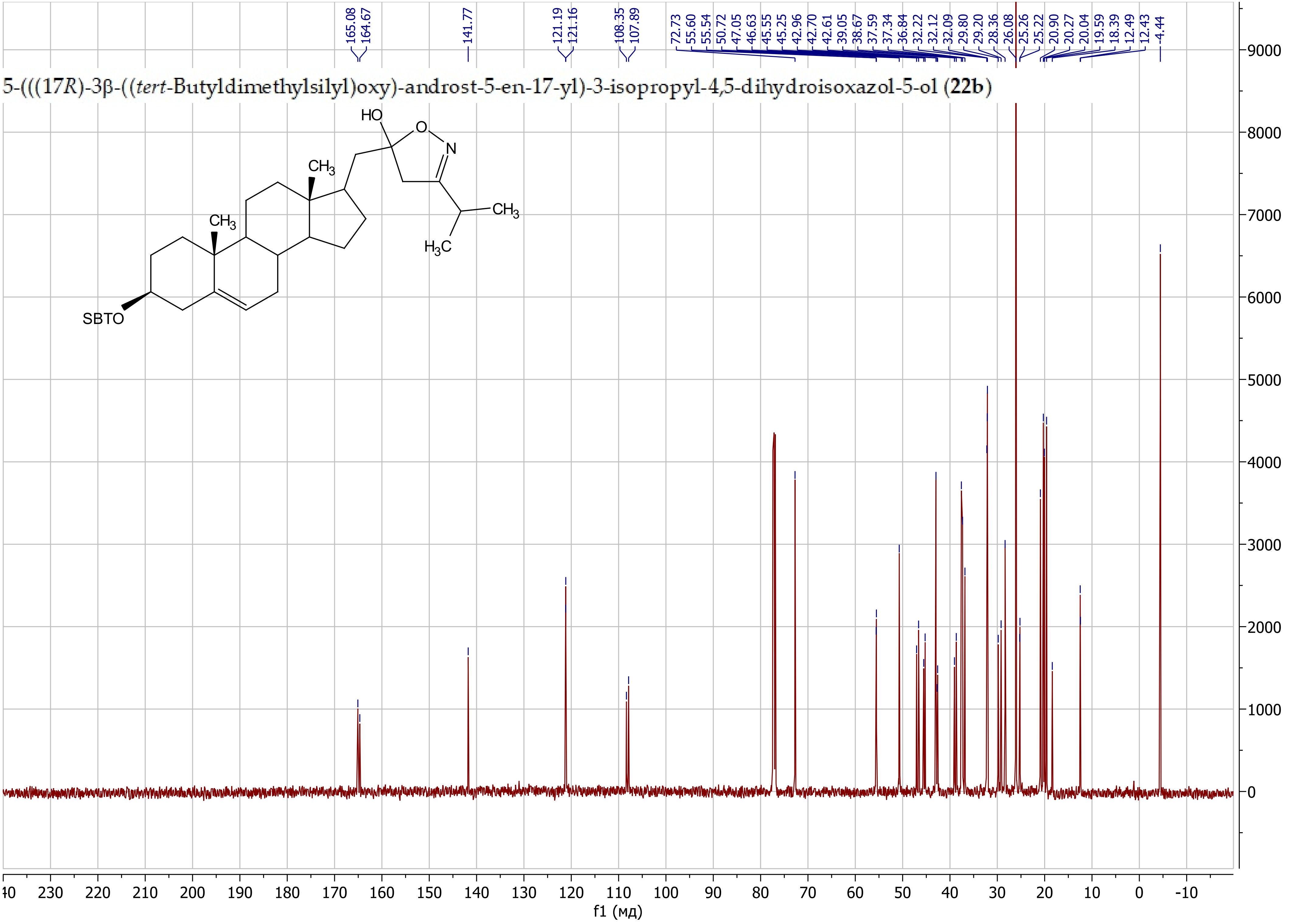

5-(((17*R*)-3β-((*tert*-Butyldimethylsilyl)oxy)-androst-5-en-17-yl)-3-cyclopropyl-4,5-dihydroisoxazol-5-ol (**22c**)

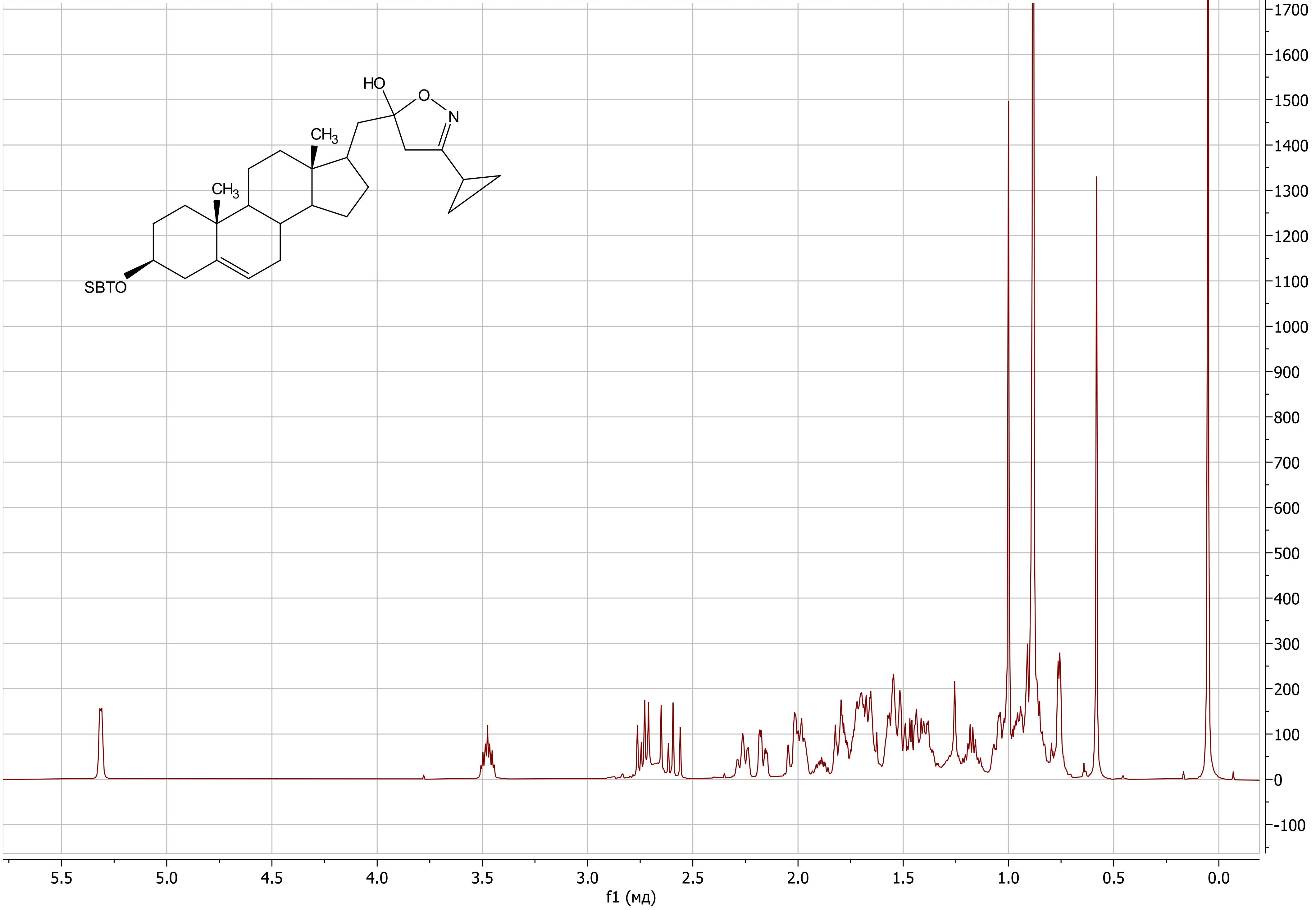

5-(((17*R*)-3β-((*tert*-Butyldimethylsilyl)oxy)-androst-5-en-17-yl)-3-cyclopropyl-4,5-dihydroisoxazol-5-ol (**22c**)

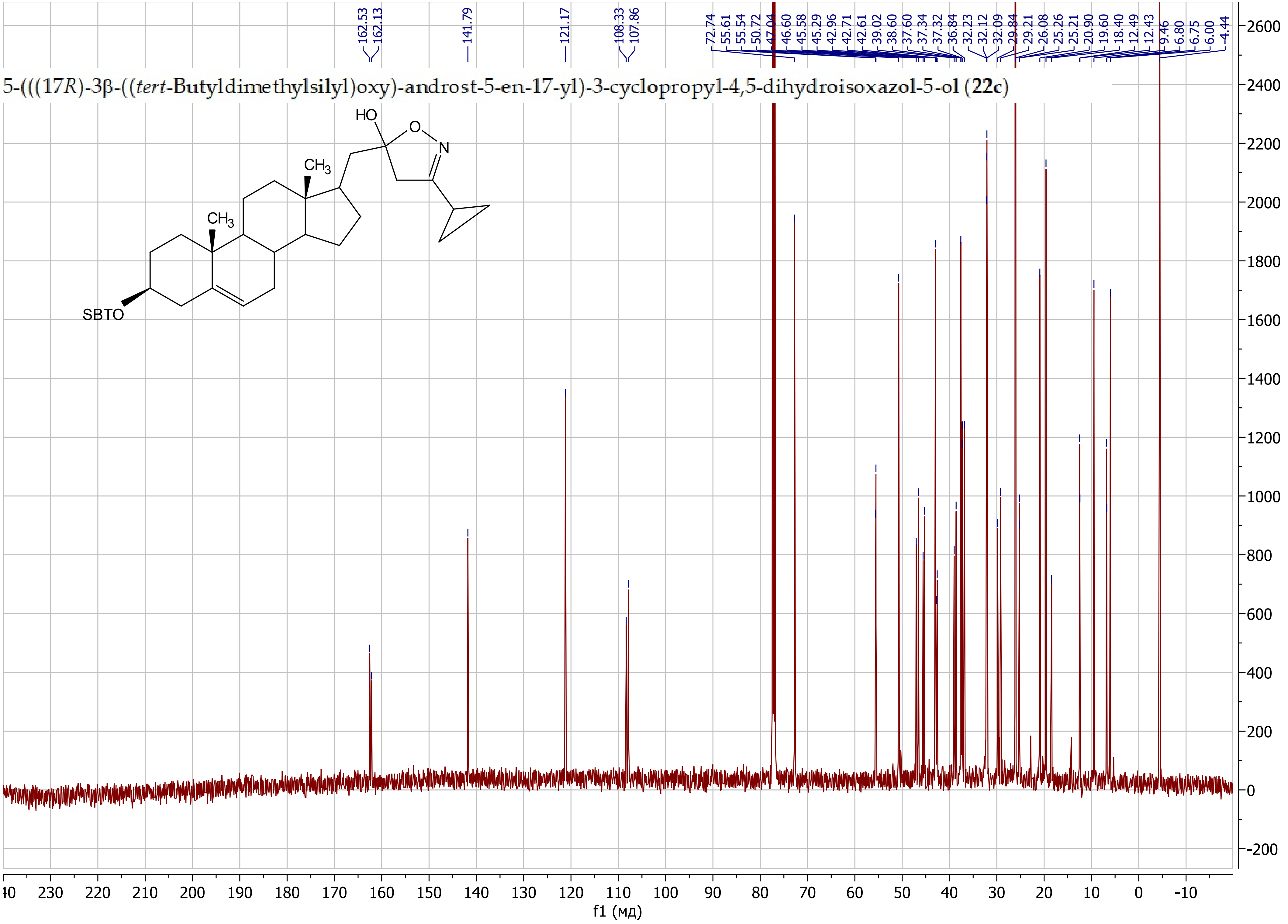

3-Butyl-5-(((17*R*)-3β-((*tert*-butyldimethylsilyl)oxy)-androst-5-en-17-yl)methyl)-4,5-dihydroisoxazol-5-ol (**22d**)

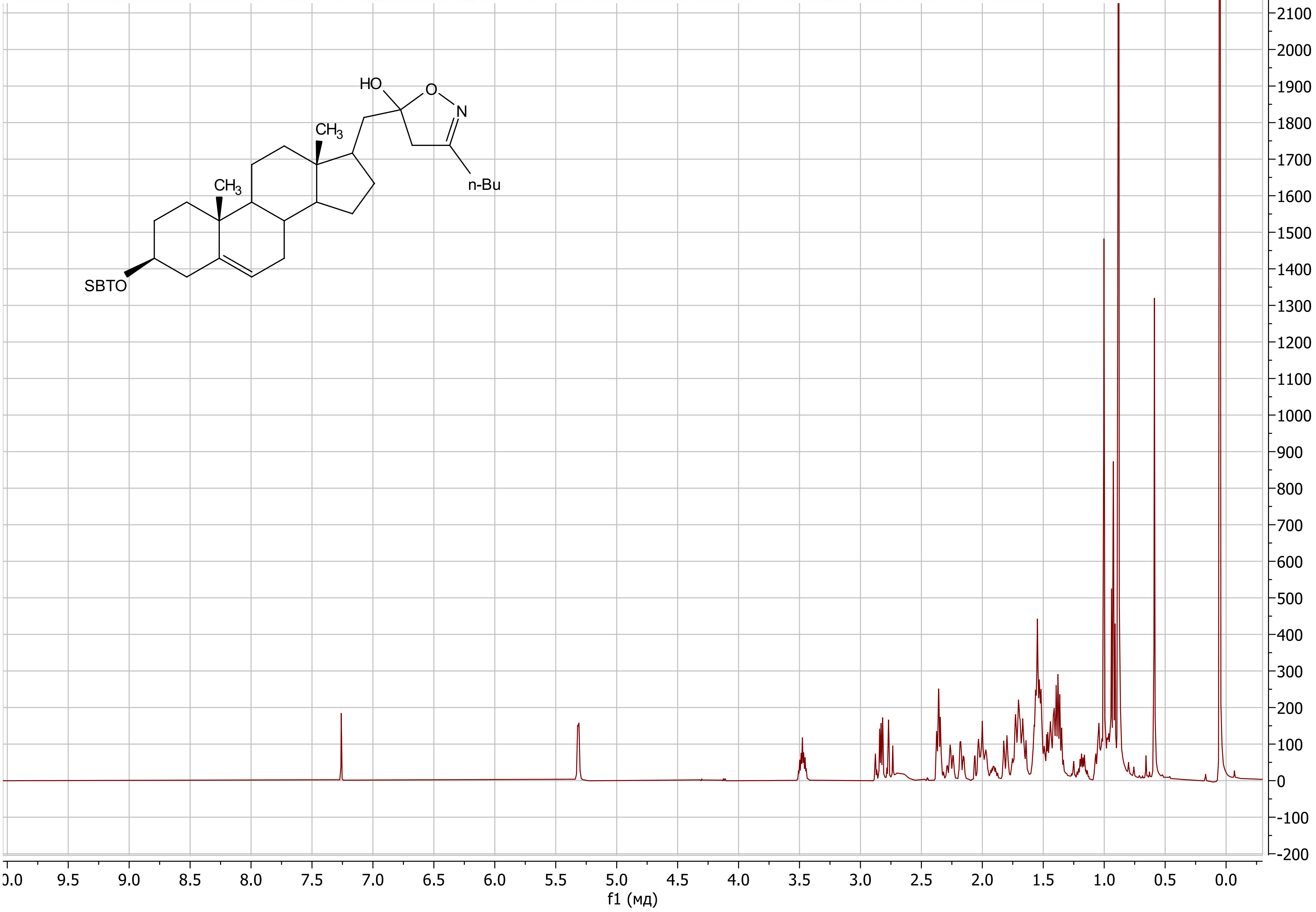

3-Butyl-5-(((17*R*)-3β-((*tert*-butyldimethylsilyl)oxy)-androst-5-en-17-yl)methyl)-4,5-dihydroisoxazol-5-ol (**22d**)

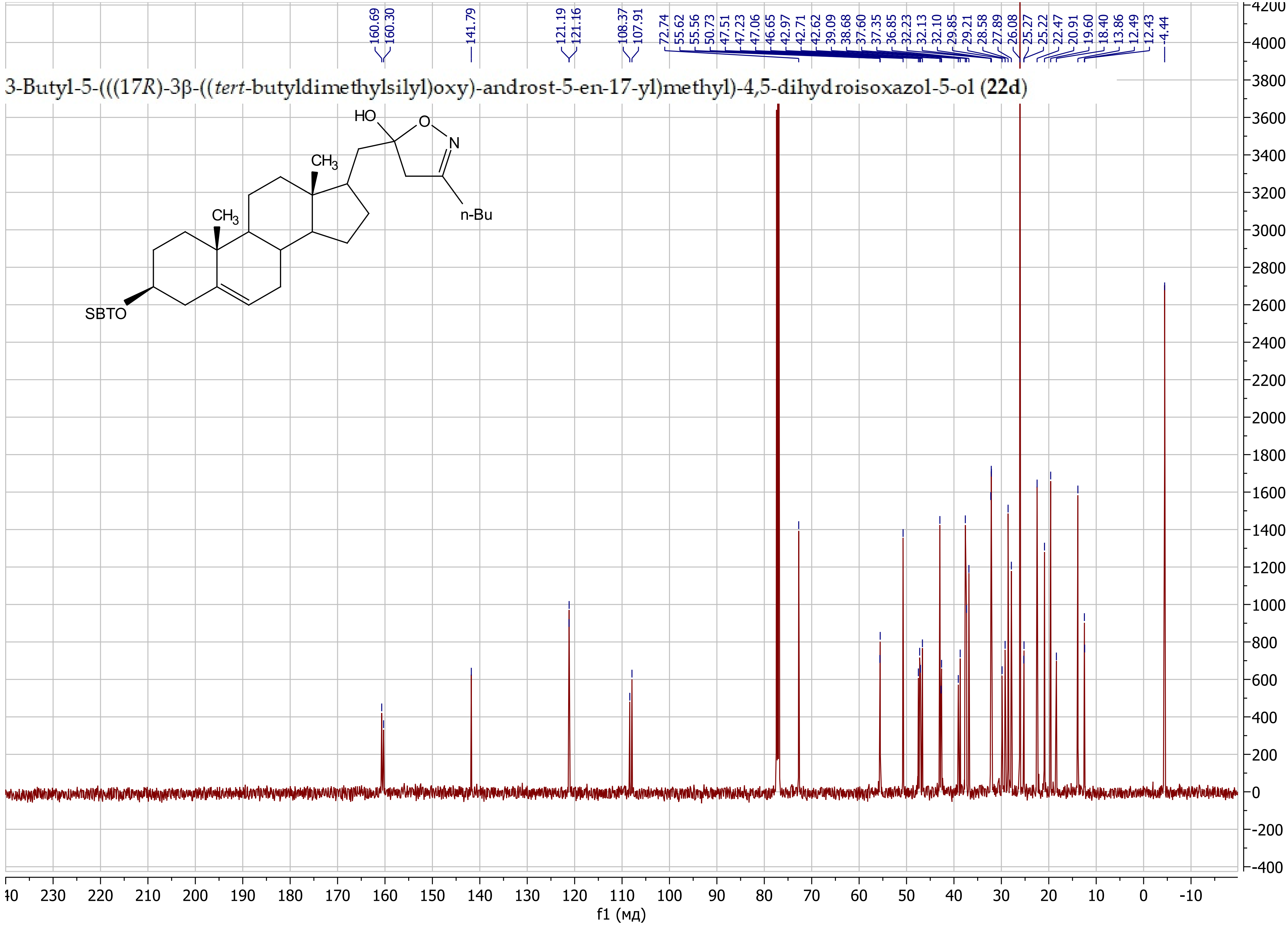

5-(((17R)-3β-((*tert*-Butyldimethylsilyl)oxy)-androst-5-en-17-yl)methyl)-3-phenyl-4,5-dihydroisoxazol-5-ol (**22e**)

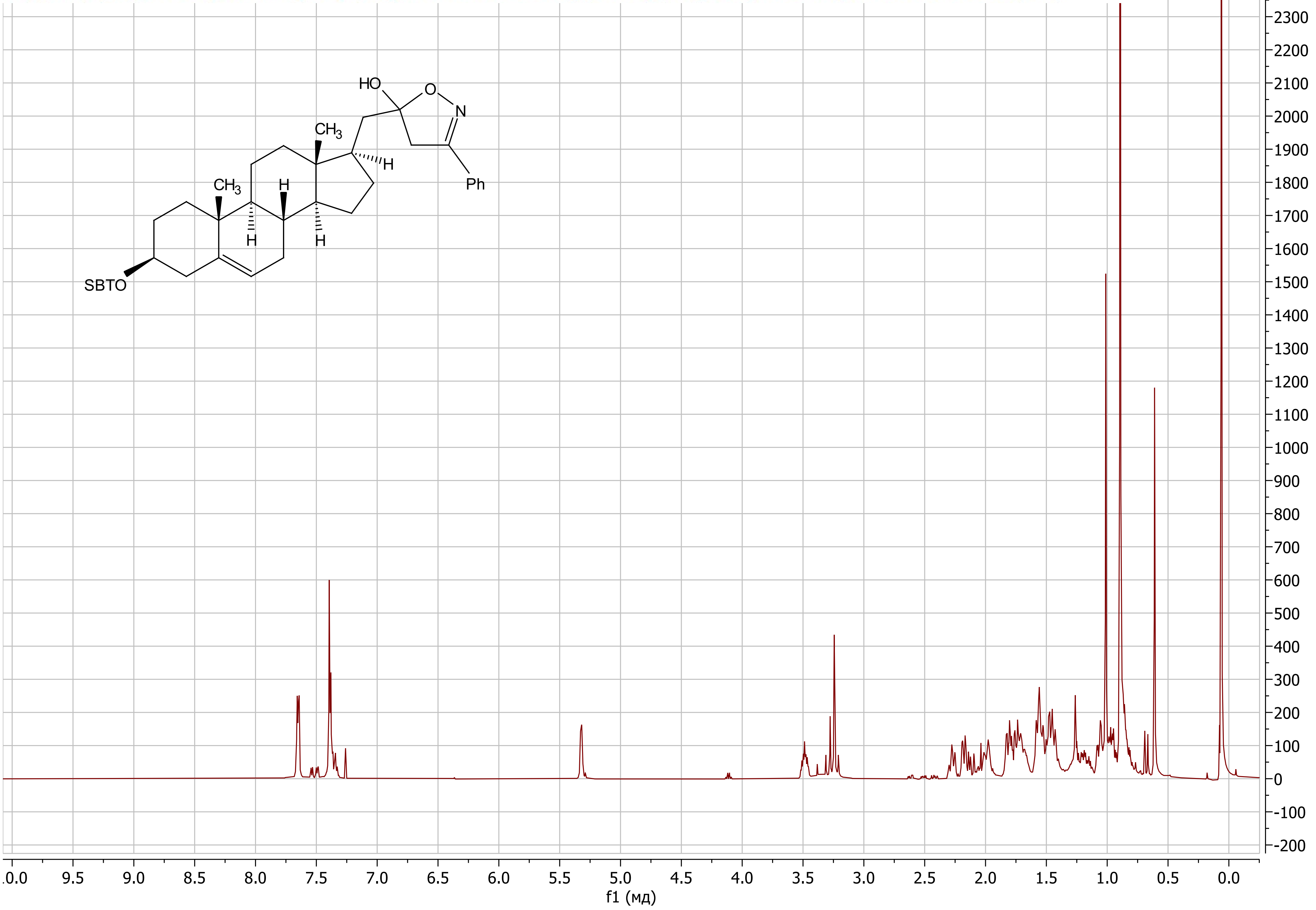

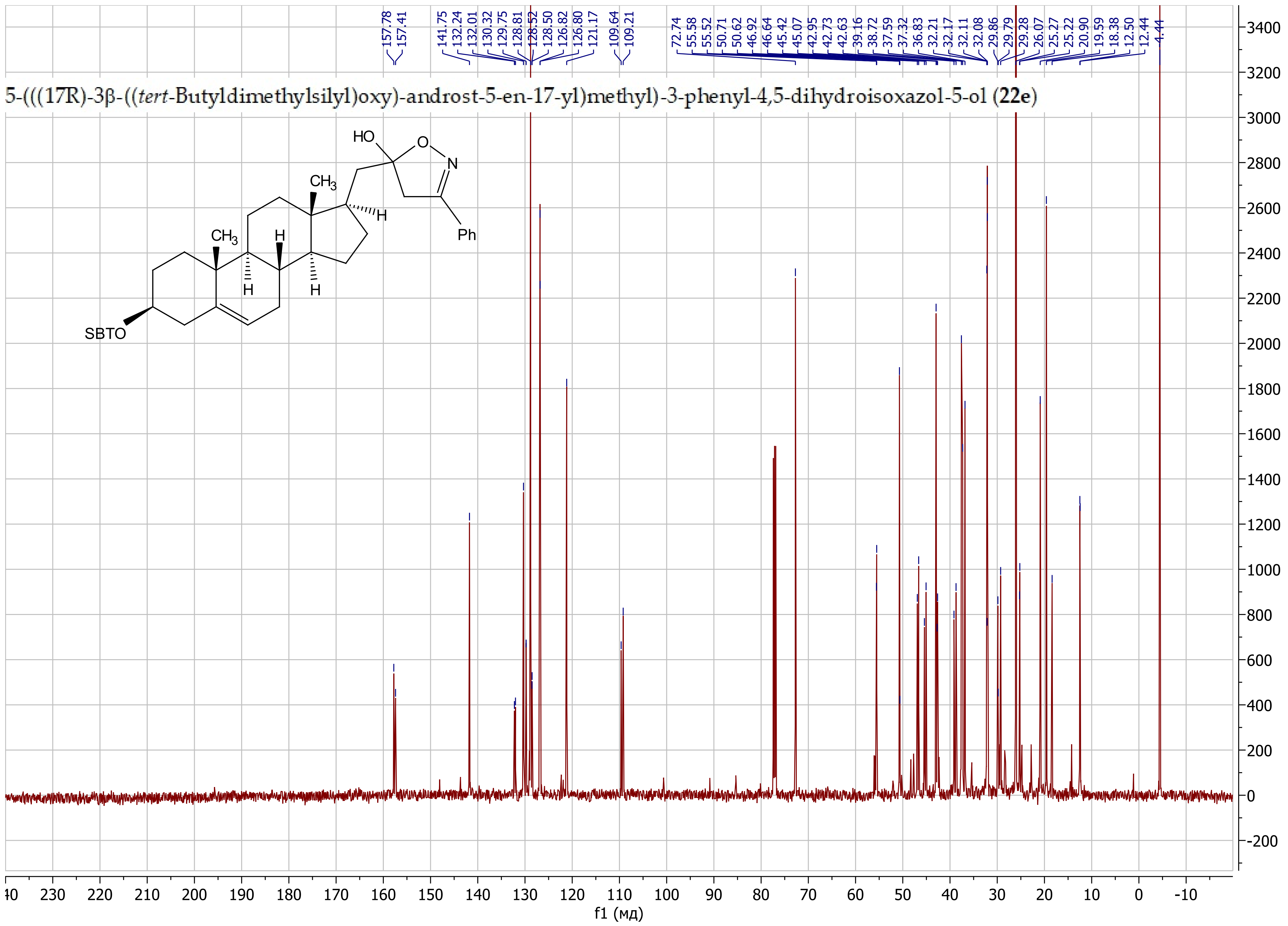
5-(((17R)-3β-((tert-Butyldimethylsilyl)oxy)-androst-5-en-17-yl)methyl)-3-phenyl-4,5-dihydroisoxazol-5-ol (22e)
157.78
157.41
141.75
132.24
132.01
130.32
129.75
128.81
128.52
128.50
126.82
126.80
121.17
109.64
109.21
72.74
55.58
55.52
50.71
50.62
46.92
46.64
45.42
45.07
42.95
42.73
42.63
39.16
38.72
37.59
37.32
36.83
32.21
32.17
32.11
32.08
29.86
29.79
29.28
26.07
25.27
25.22
20.90
19.59
18.38
12.50
12.44
4.44
HO
O
N
CH3
H
Ph
CH3
H
H
H
SBTO
f1 (мд)

5-(((17R)-3β-((*tert*-Butyldimethylsilyl)oxy)-androst-5-en-17-yl)-3-(pyridin-3-yl)-4,5-dihydroisoxazol-5-ol (**22f**)

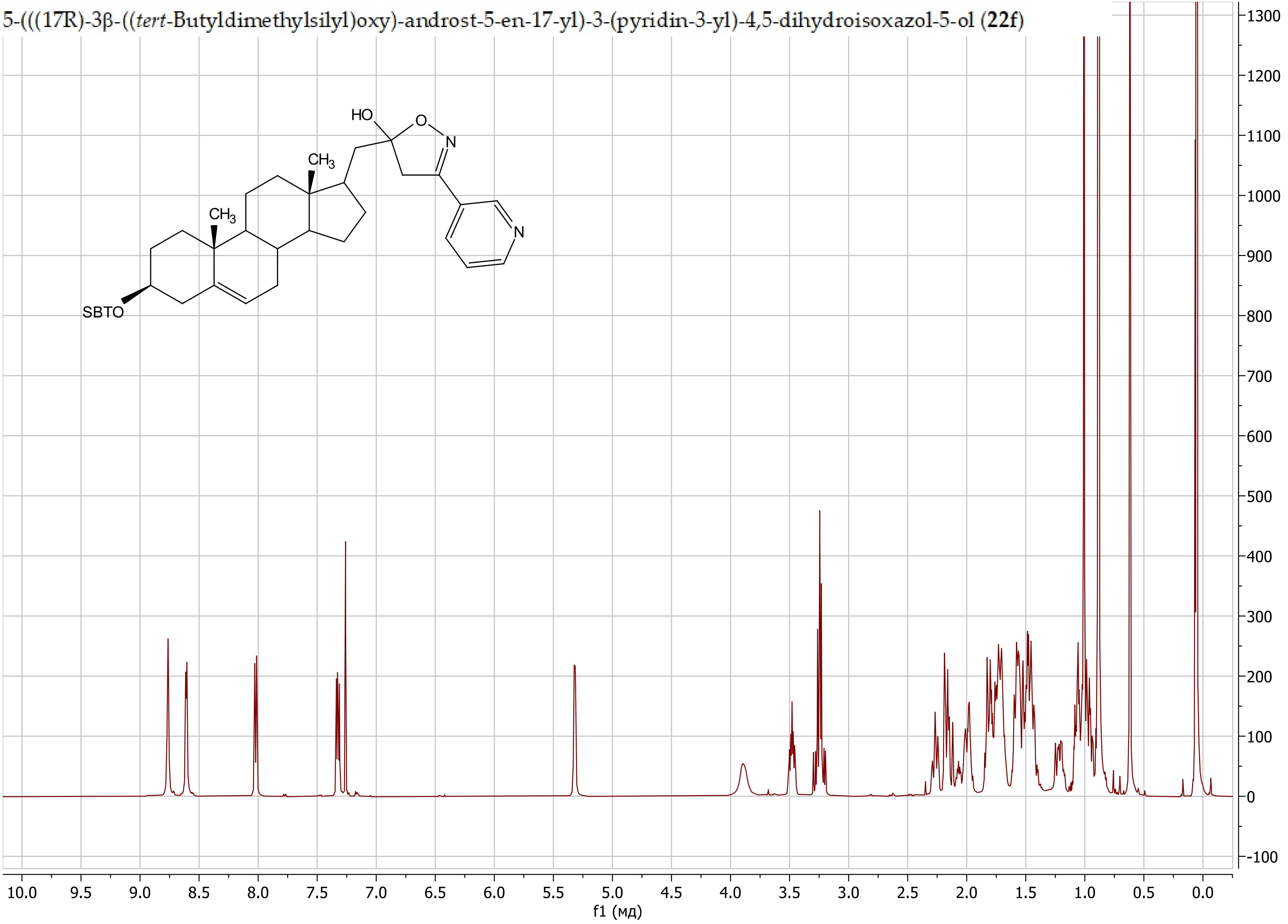

5-(((17R)-3β-((*tert*-Butyldimethylsilyl)oxy)-androst-5-en-17-yl)-3-(pyridin-3-yl)-4,5-dihydroisoxazol-5-ol (**22f**)

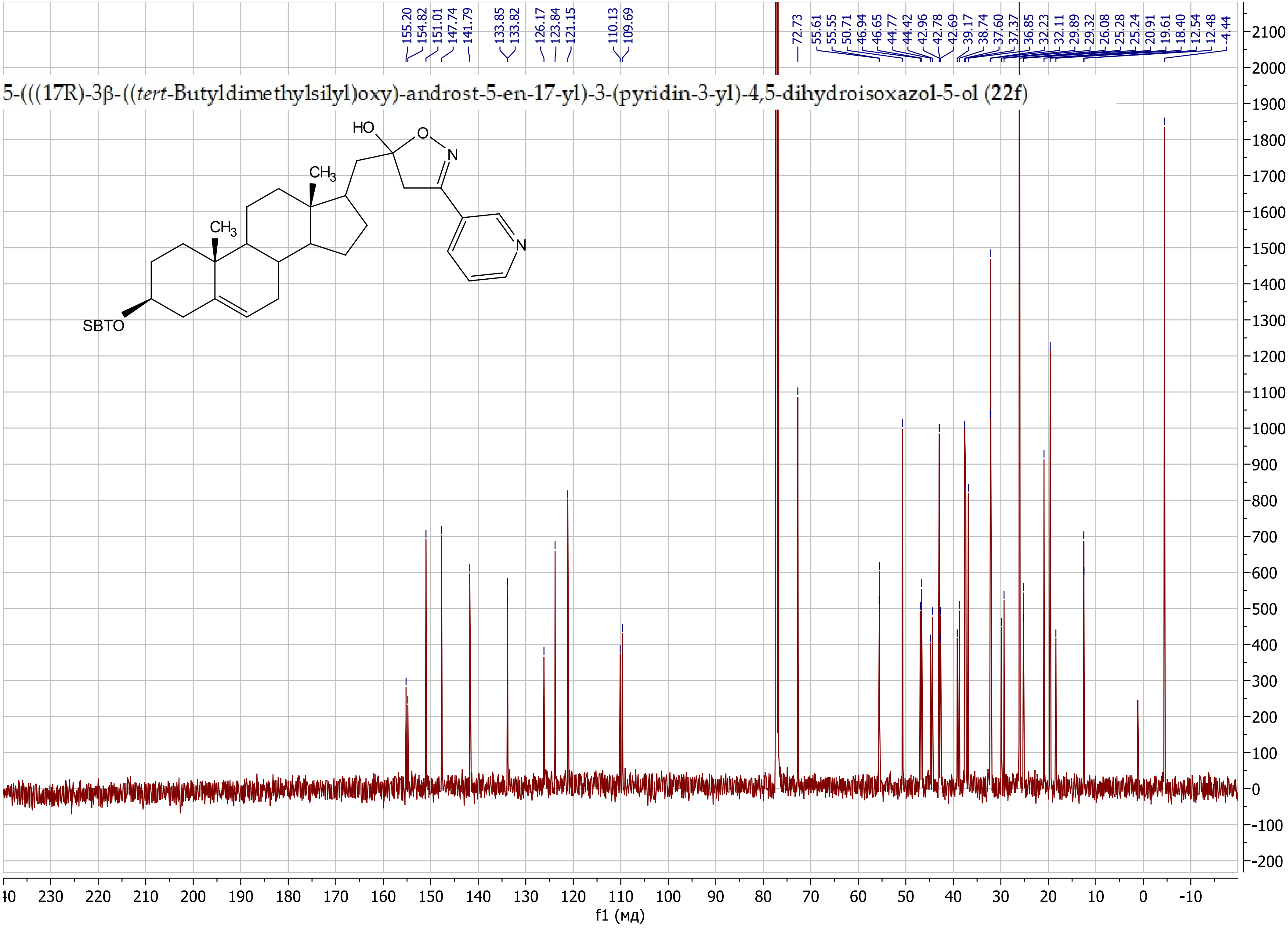

5-(((17R)-3β-((*tert*-Butyldimethylsilyl)oxy)-androst-5-en-17-yl)-3-(2-fluorophenyl)-4,5-dihydroisoxazol-5-ol (**22g**)

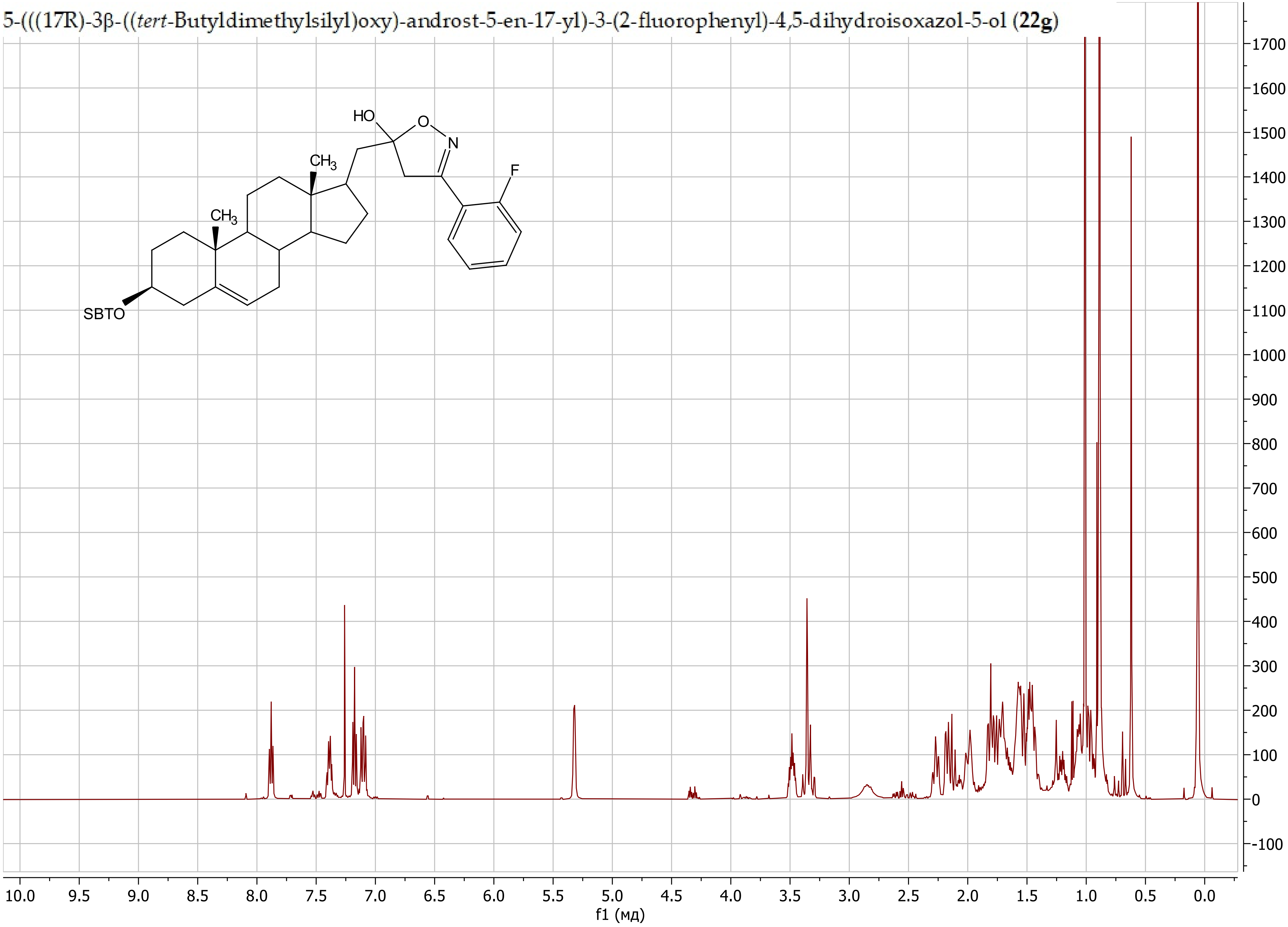

5-(((17R)-3β-((*tert*-Butyldimethylsilyl)oxy)-androst-5-en-17-yl)-3-(2-fluorophenyl)-4,5-dihydroisoxazol-5-ol (**22g**)

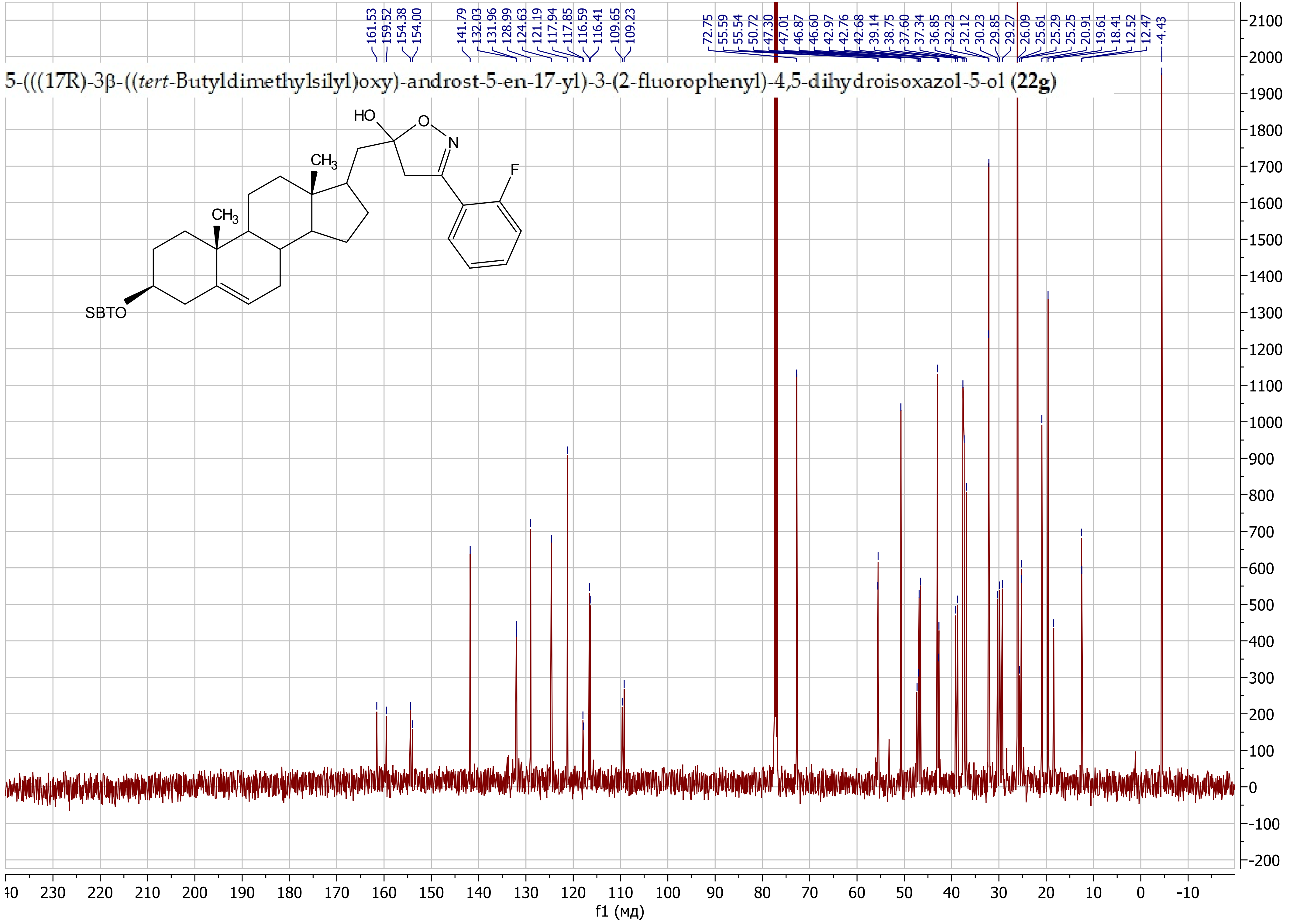

5-(((17R)-3β-((*tert*-Butyldimethylsilyl)oxy)-androst-5-en-17-yl)-3-(2-((tetrahydro-2*H*-pyran-2-yl)oxy)propan-2-yl)-4,5-dihydroisoxazol-5-ol (**22h**)

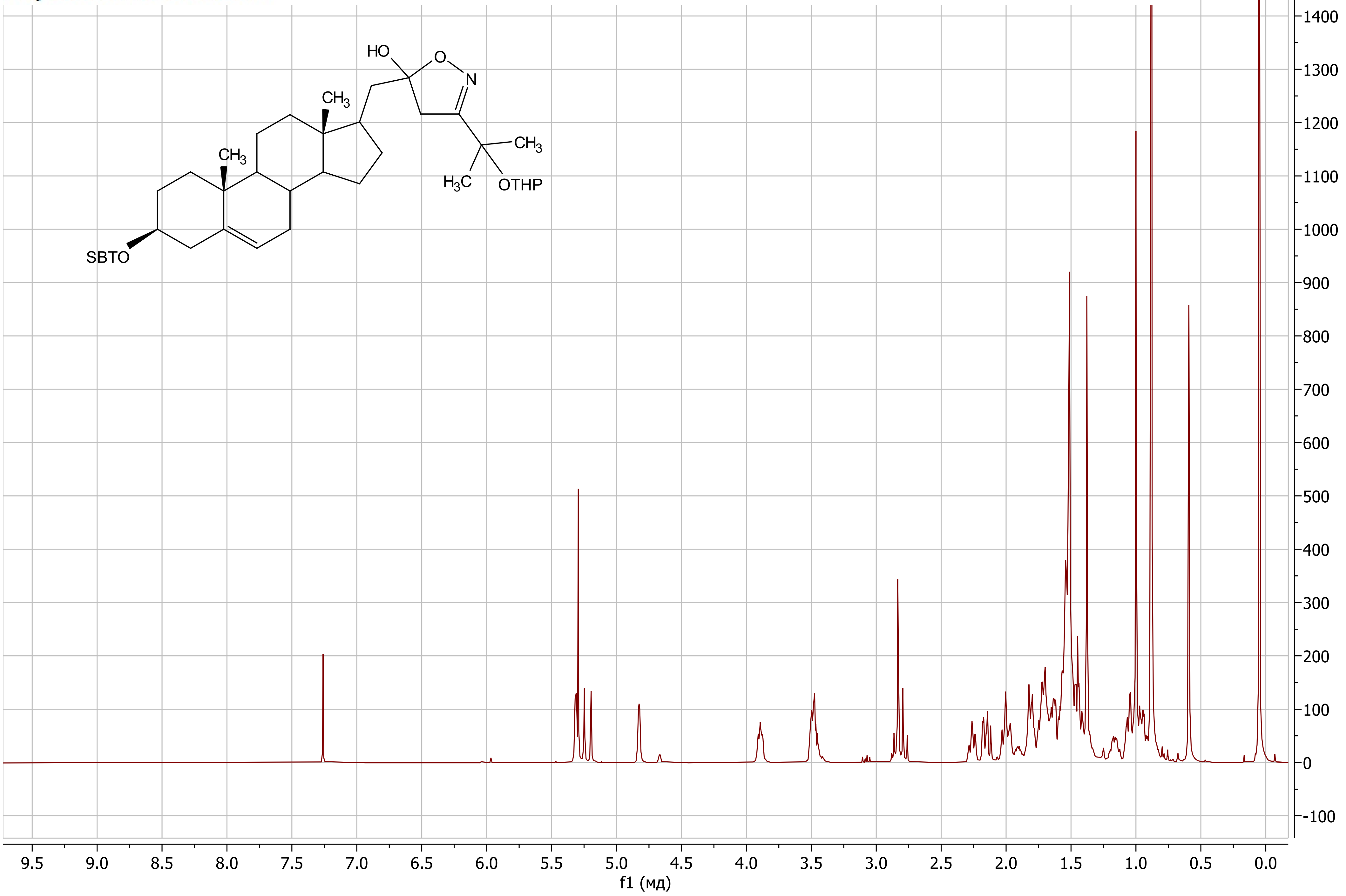

5-(((17R)-3β-((*tert*-Butyldimethylsilyl)oxy)-androst-5-en-17-yl)-3-(2-((tetrahydro-2*H*-pyran-2-yl)oxy)propan-2-yl)-4,5-dihydroisoxazol-5-ol (**22h**)

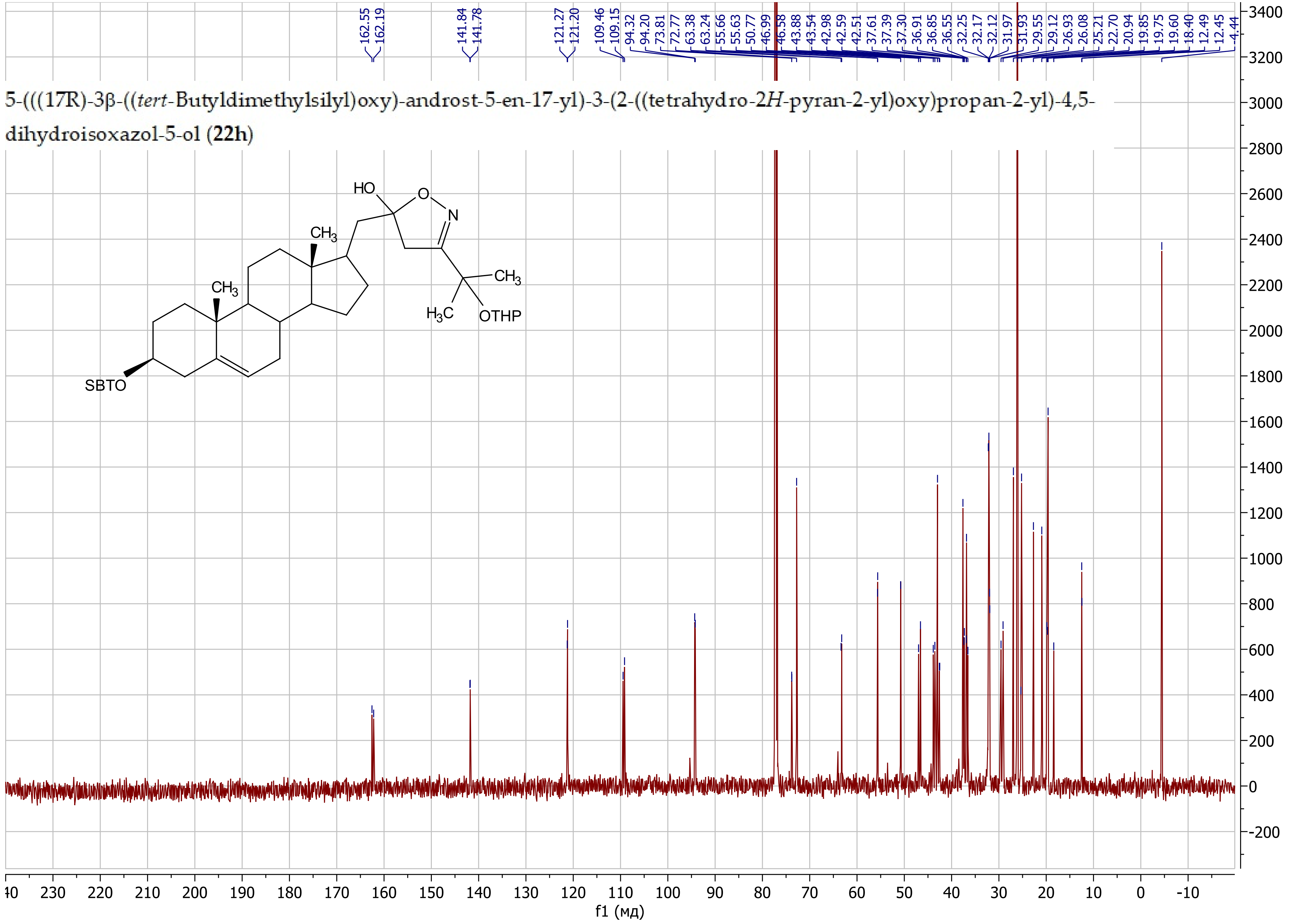

5-(((17R)-3β-((*tert*-Butyldimethylsilyl)oxy)-androst-5-en-17-yl)methyl)-3-(((tetrahydro-2*H*-pyran-2-yl)oxy)methyl)-4,5-dihydroisoxazol-5-ol (**22i**)

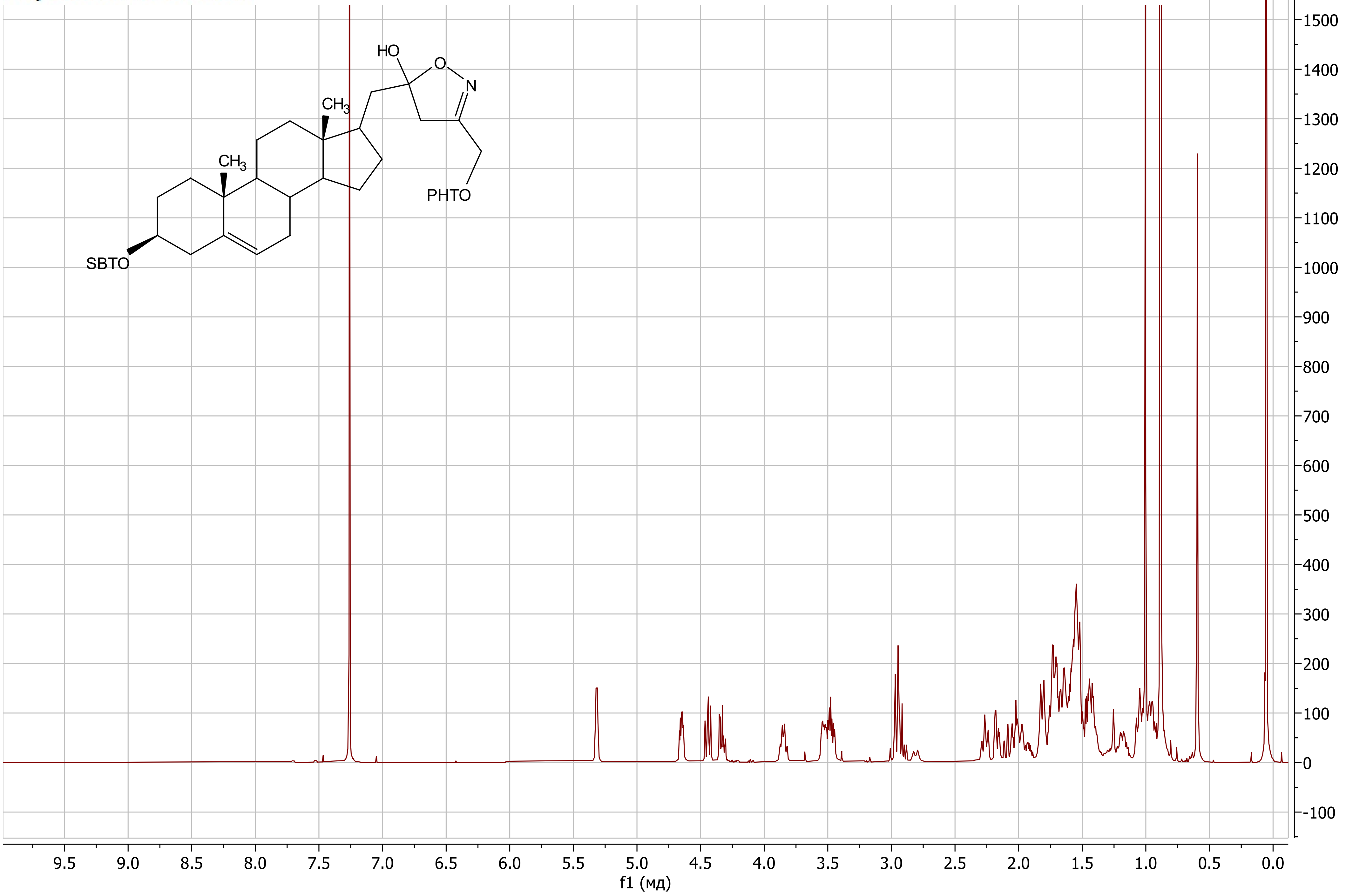

5-(((17R)-3β-((*tert*-Butyldimethylsilyl)oxy)-androst-5-en-17-yl)methyl)-3-((((tetrahydro-2*H*-pyran-2-yl)oxy)methyl)-4,5-dihydroisoxazol-5-ol (**22i**)

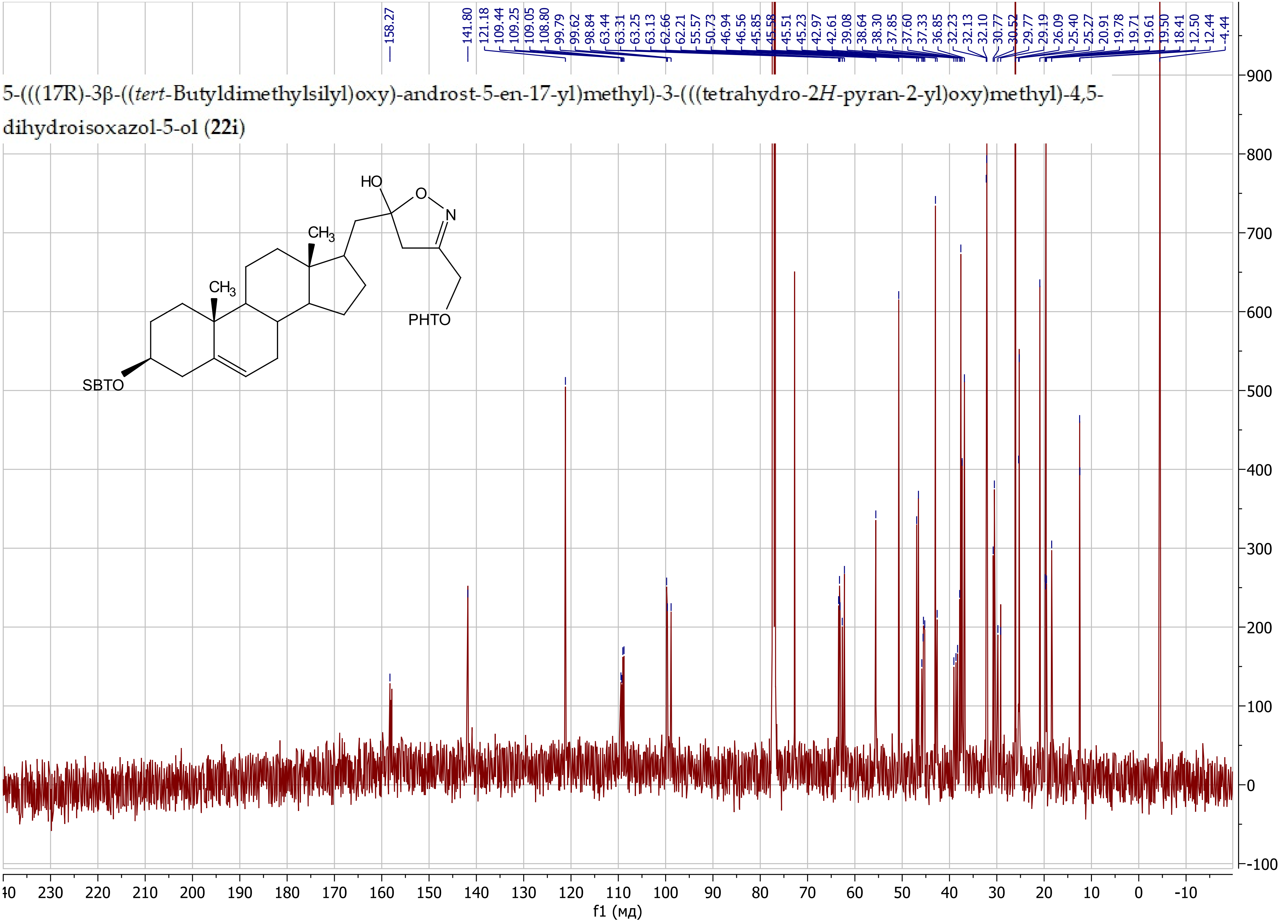

5-(((17*R*)-3β-((*tert*-butyldimethylsilyl)oxy)-androst-5-en-17-yl)methyl)isoxazole (**23a**)

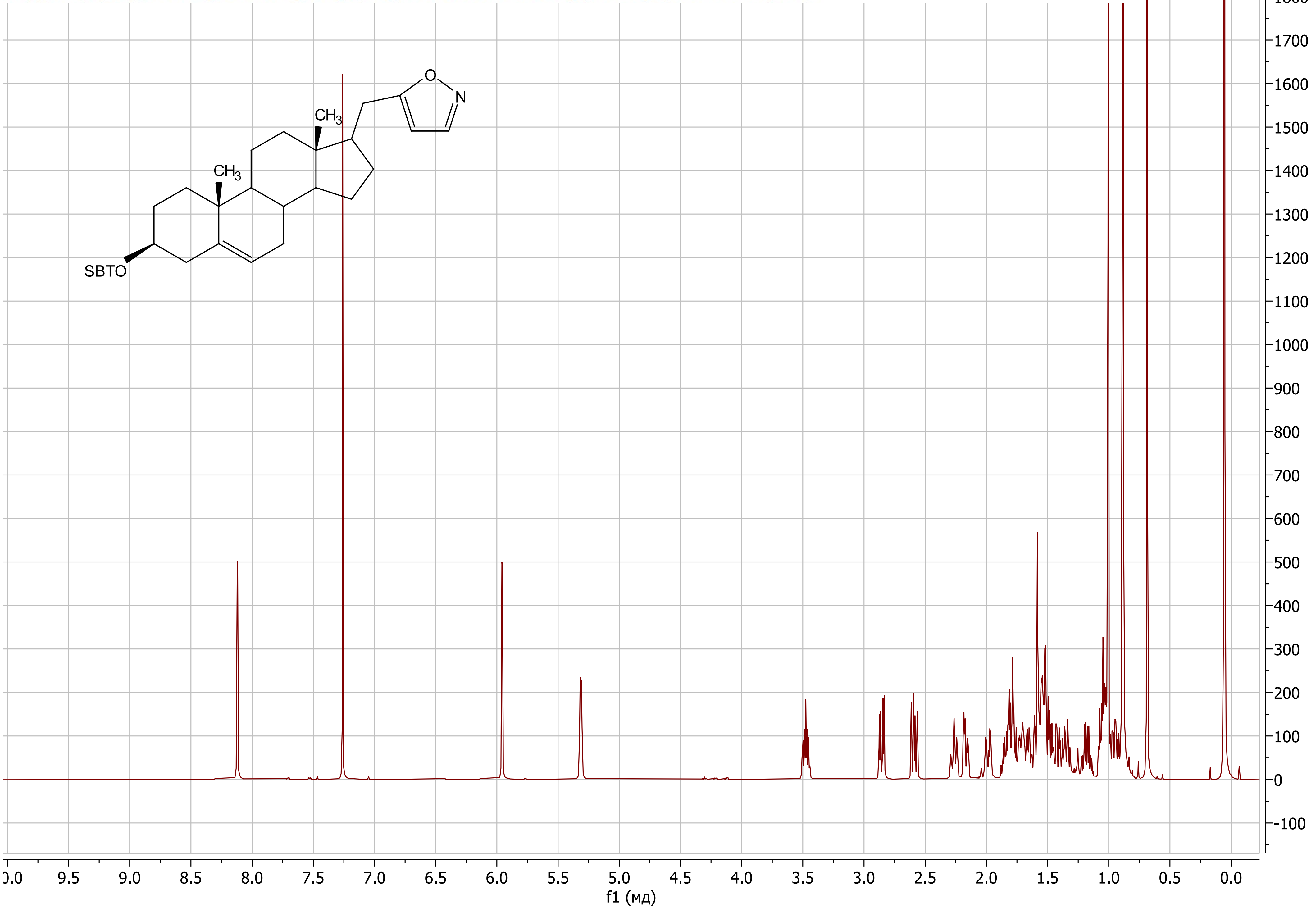

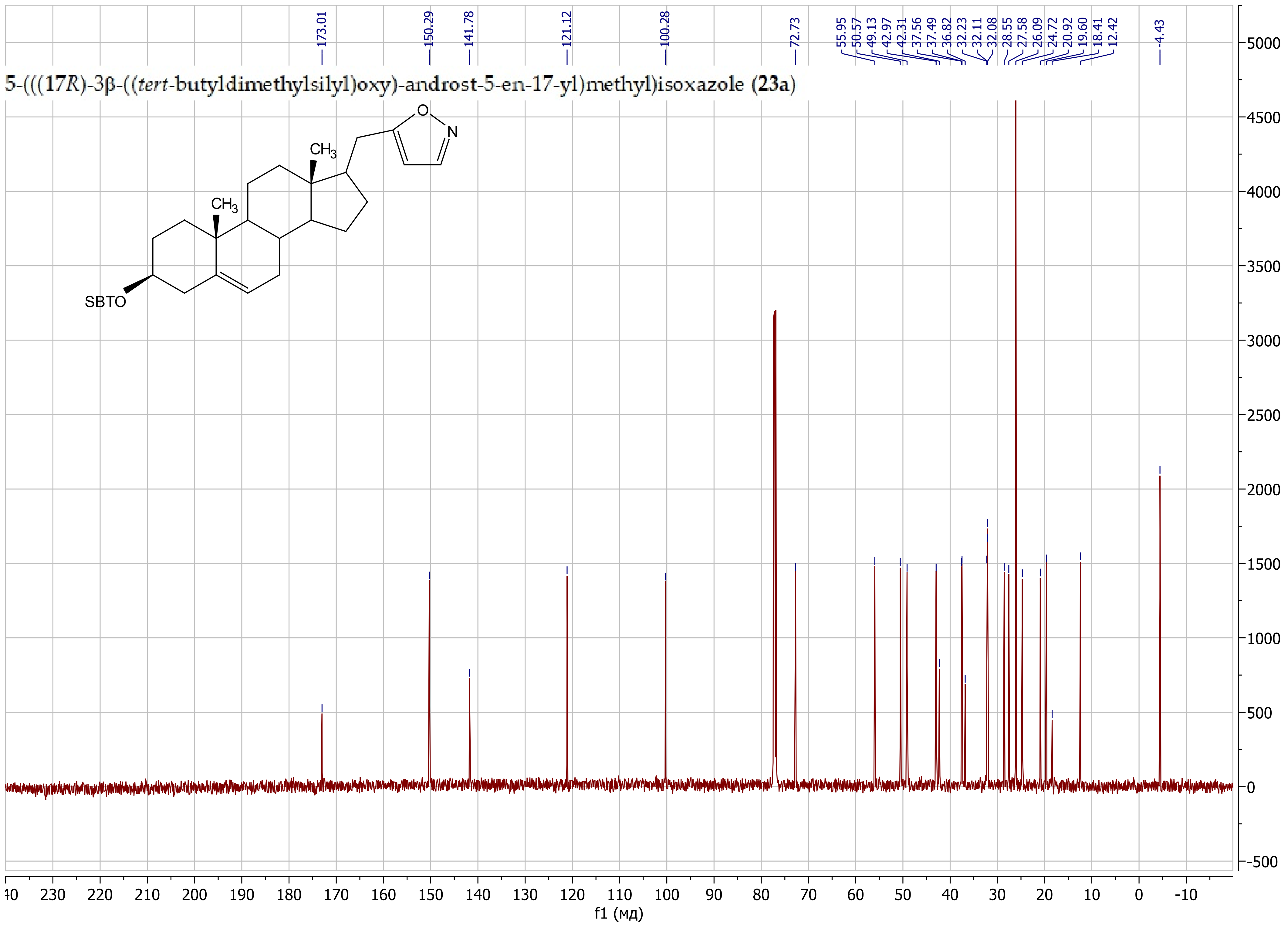
5-(((17R)-3β-((tert-butyldimethylsilyl)oxy)-androst-5-en-17-yl)methyl)isoxazole (23a)
173.01
150.29
141.78
121.12
100.28
72.73
55.95
50.57
49.13
42.97
42.31
37.56
37.49
36.82
32.23
32.11
32.08
28.55
27.58
26.09
24.72
20.92
19.60
18.41
12.42
-4.43
O
N
CH3
CH3
SBTO
f1 (мд)

5-(((17*R*)-3β-((*tert*-Butyldimethylsilyl)oxy)-androst-5-en-17-yl)methyl)-3-isopropylisoxazole (**23b**)

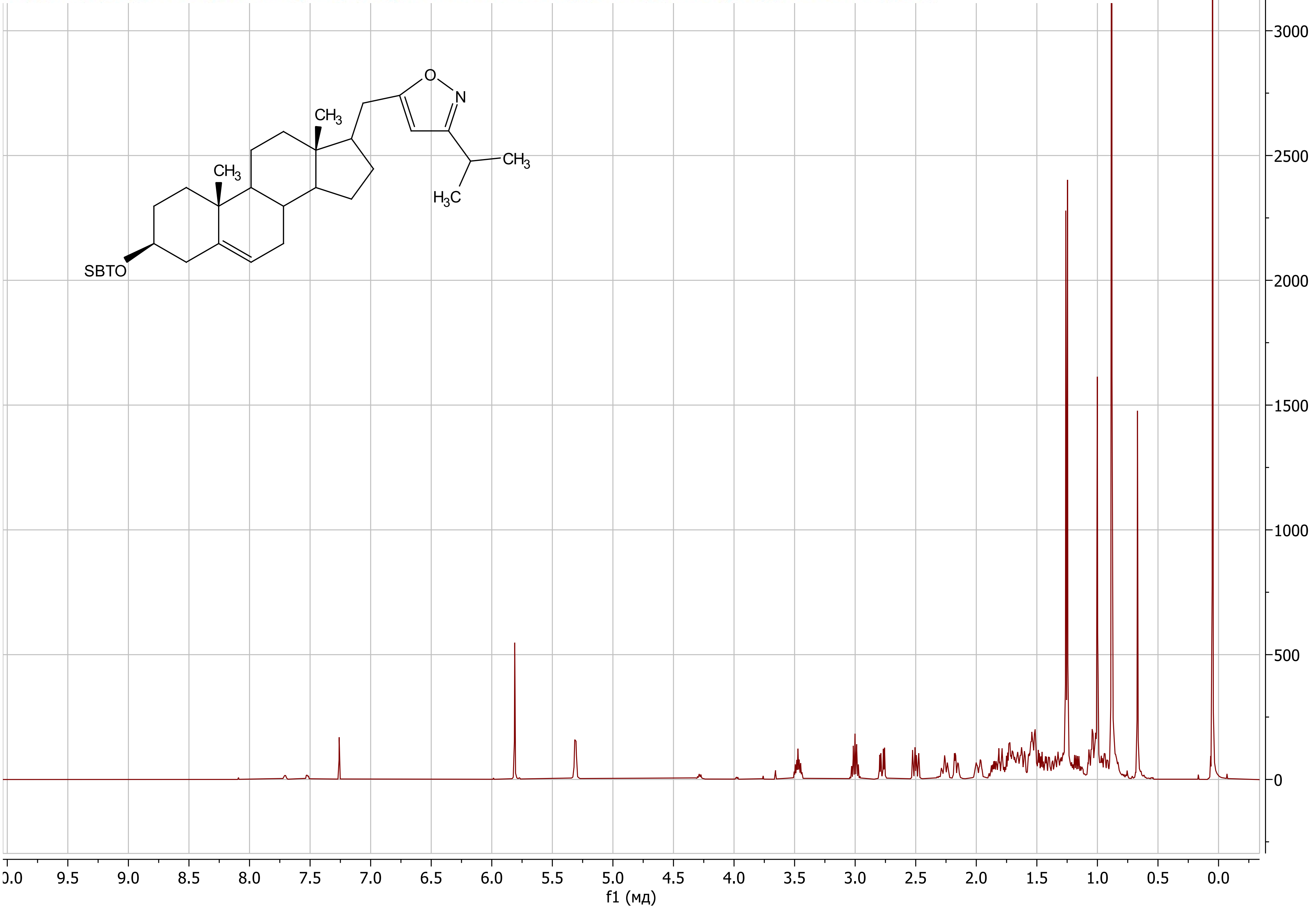

5-(((17*R*)-3β-((*tert*-Butyldimethylsilyl)oxy)-androst-5-en-17-yl)methyl)-3-isopropylisoxazole (**23b**)

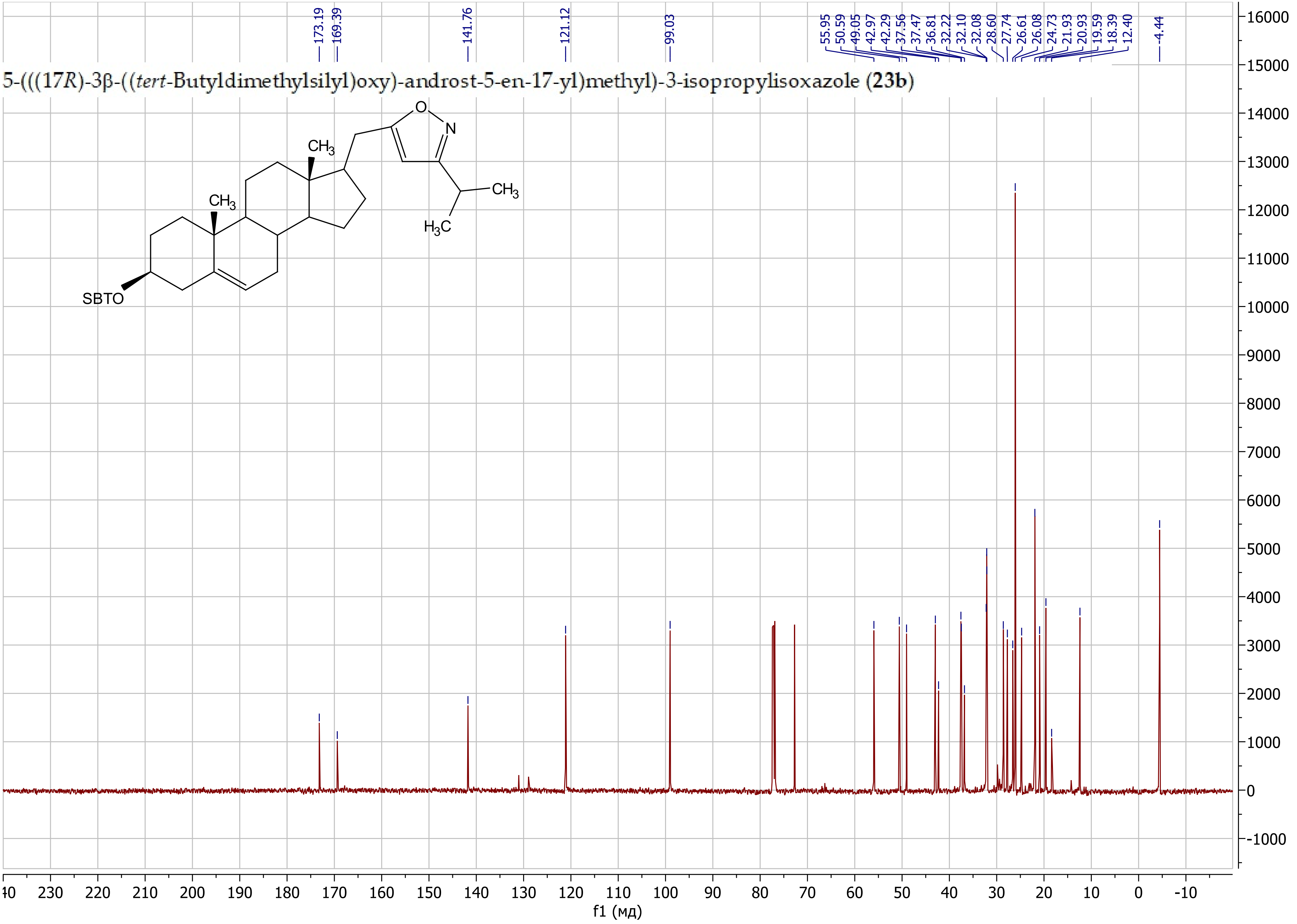

5-(((17*R*)-3β-((*tert*-Butyldimethylsilyl)oxy)-androst-5-en-17-yl)methyl)-3-cyclopropylisoxazole (**23c**)

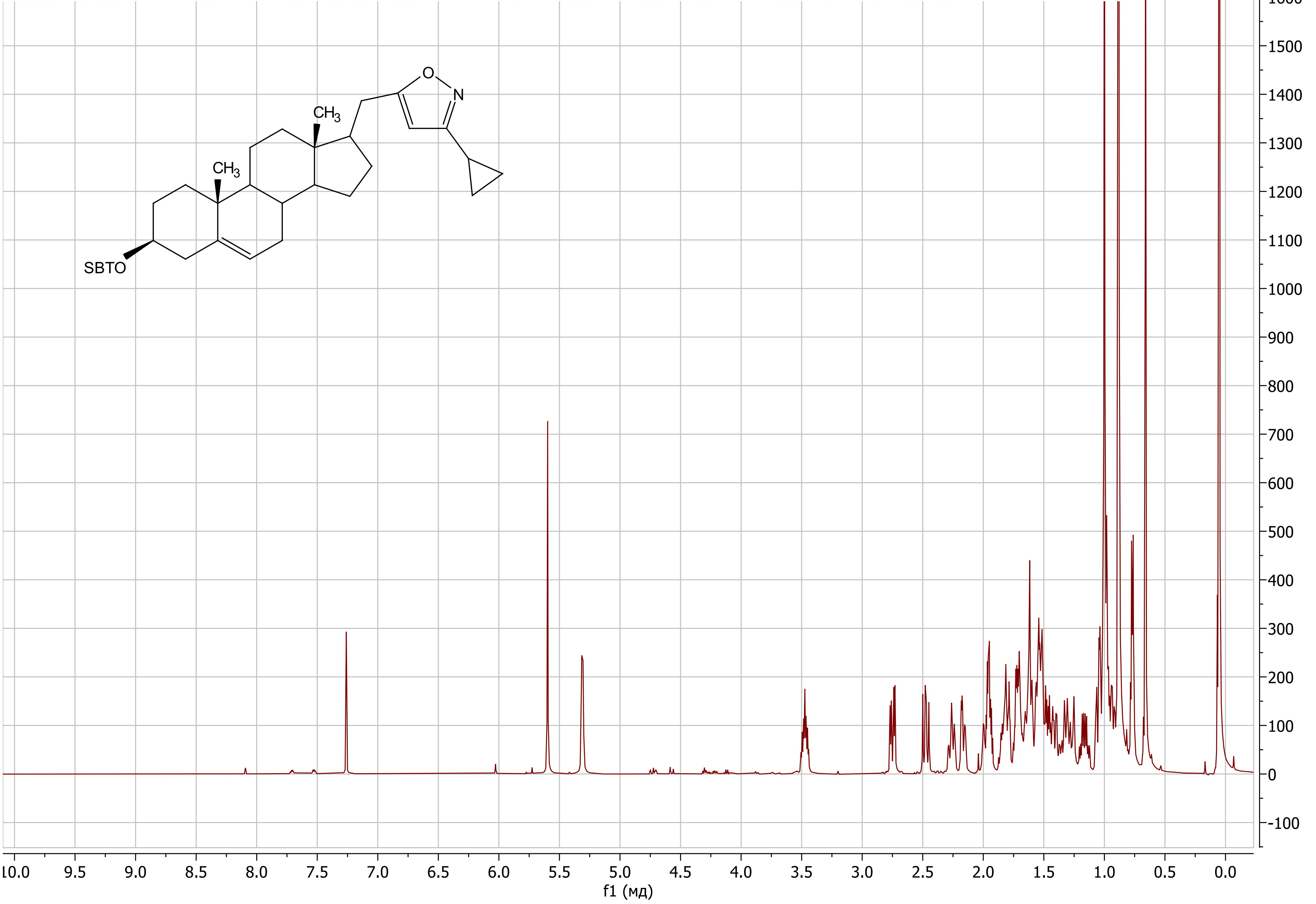

5-(((17*R*)-3β-((*tert*-Butyldimethylsilyl)oxy)-androst-5-en-17-yl)methyl)-3-cyclopropylisoxazole (**23c**)

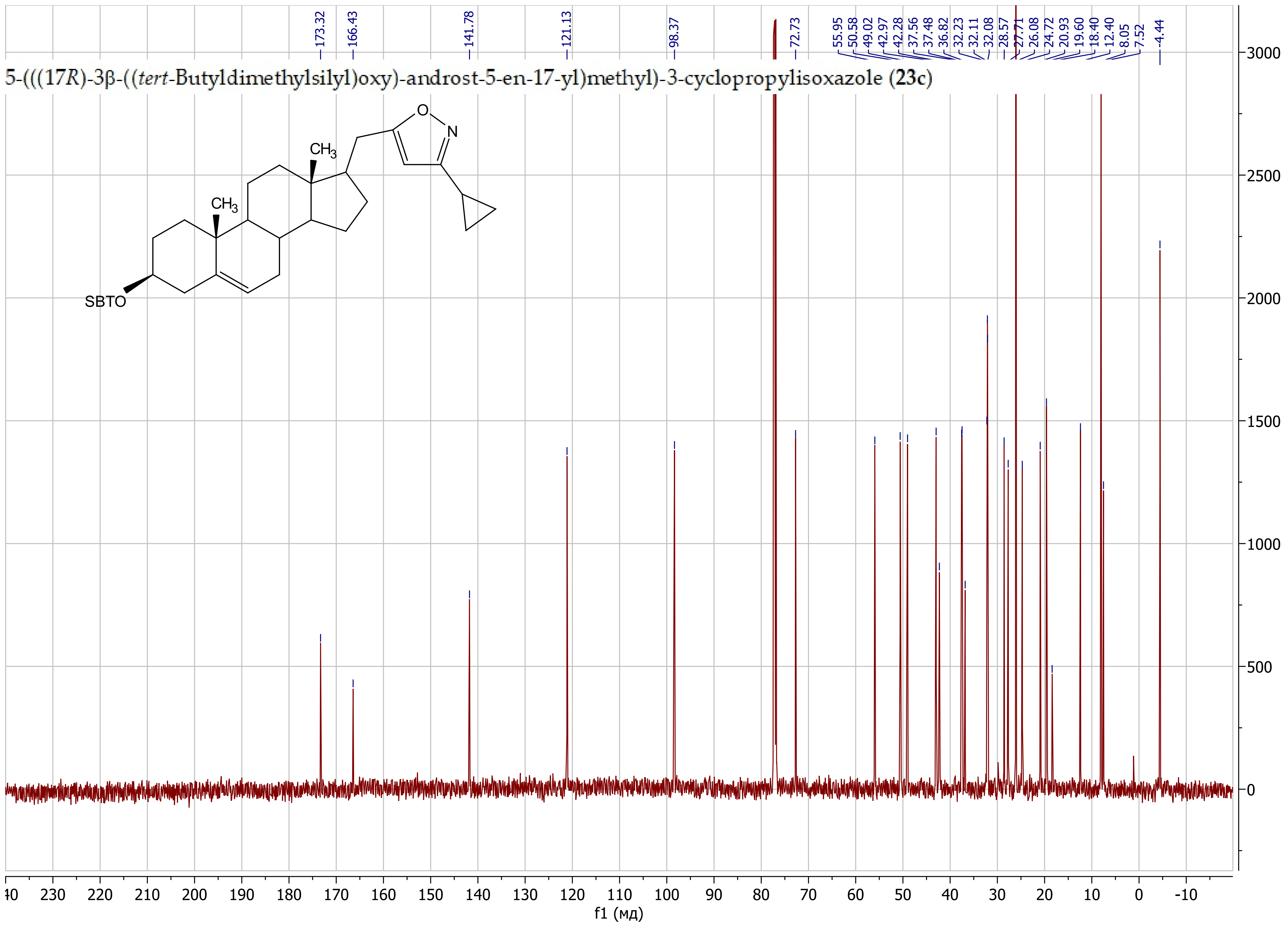

3-Butyl-5-(((17*R*)-3β-((*tert*-butyldimethylsilyl)oxy)-androst-5-en-17-yl)methyl)isoxazole (**23d**)

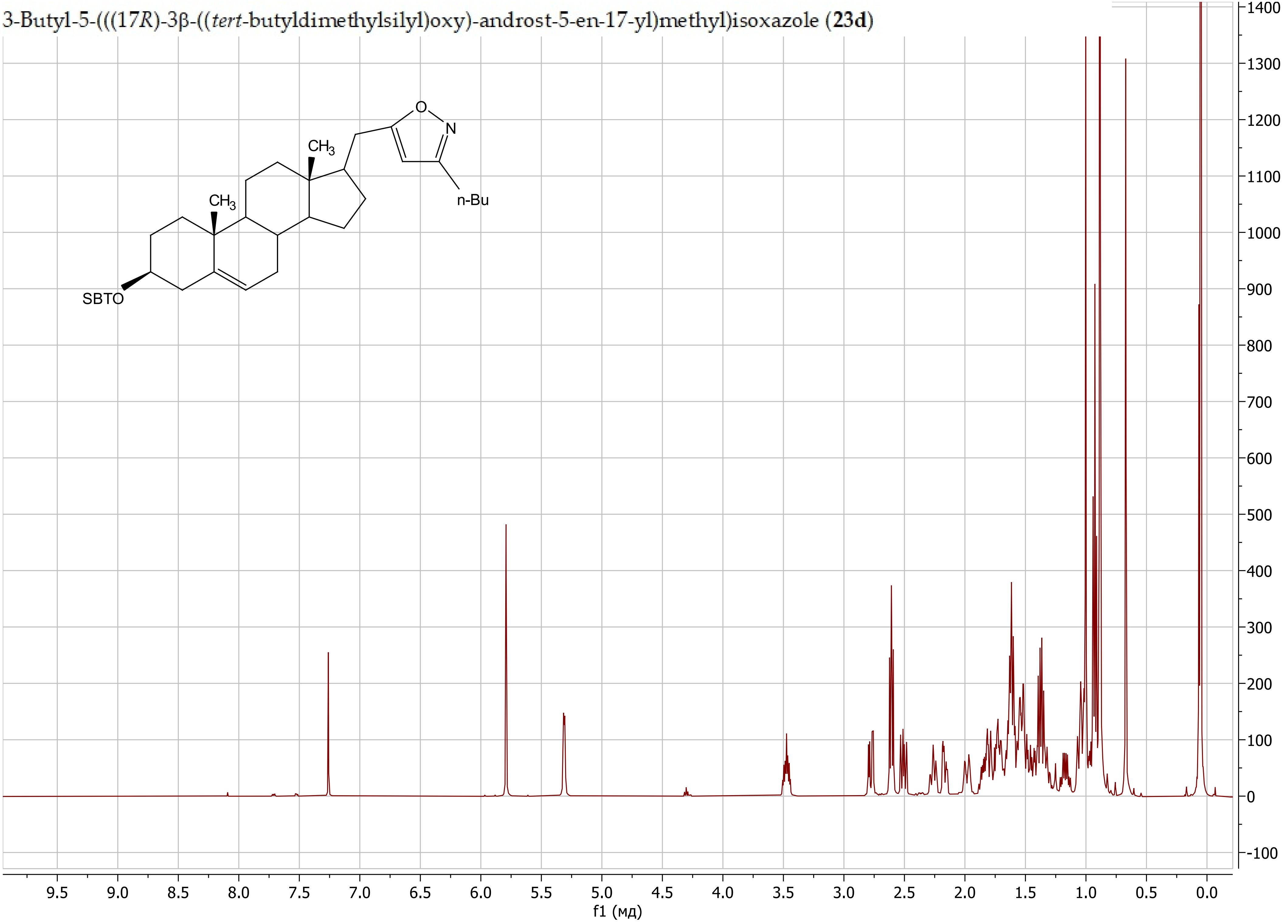

3-Butyl-5-(((17*R*)-3β-((*tert*-butyldimethylsilyl)oxy)-androst-5-en-17-yl)methyl)isoxazole (**23d**)

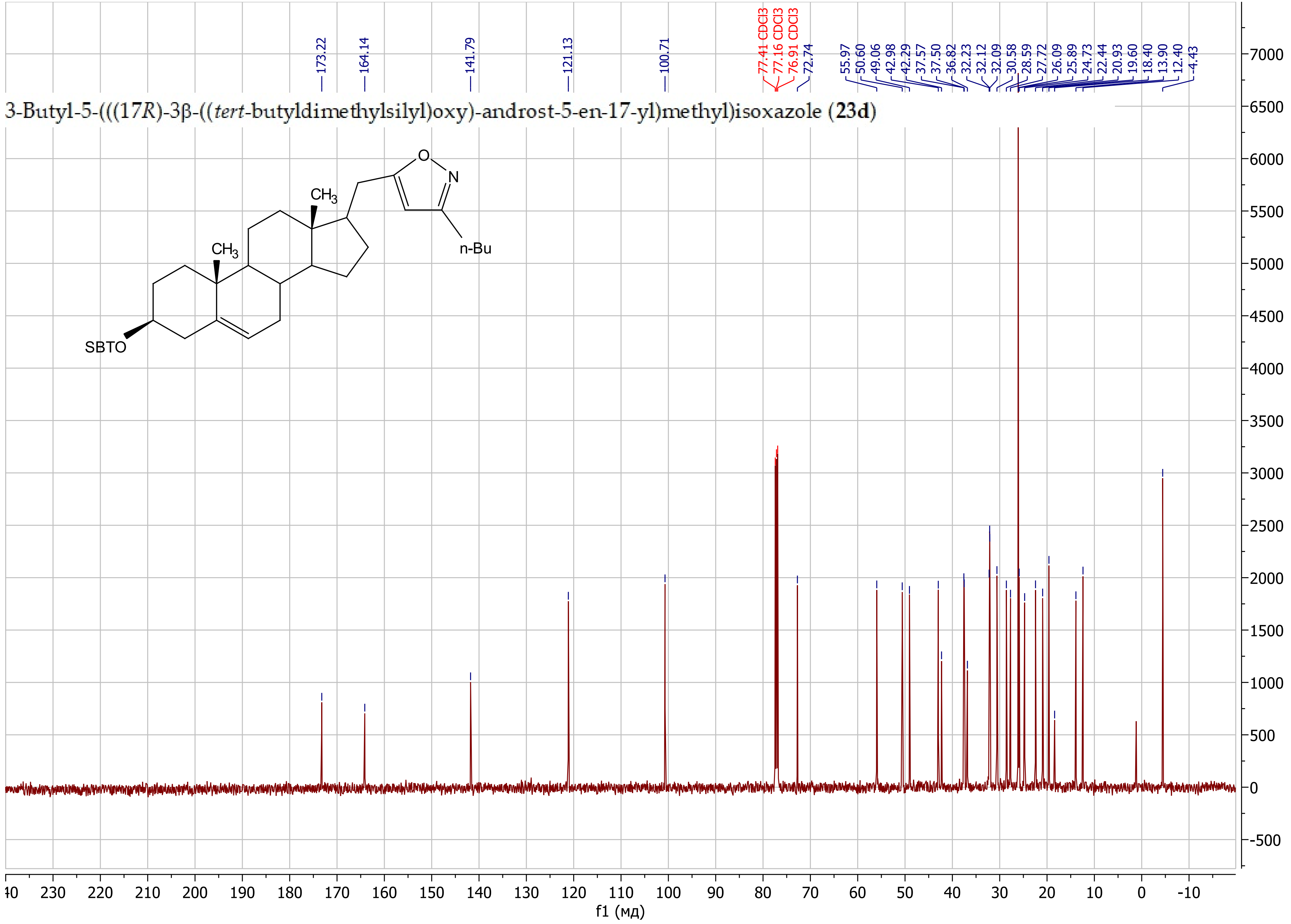

5-(((17*R*)-3β-((*tert*-Butyldimethylsilyl)oxy)-androst-5-en-17-yl)methyl)-3-phenylisoxazole (**23e**)

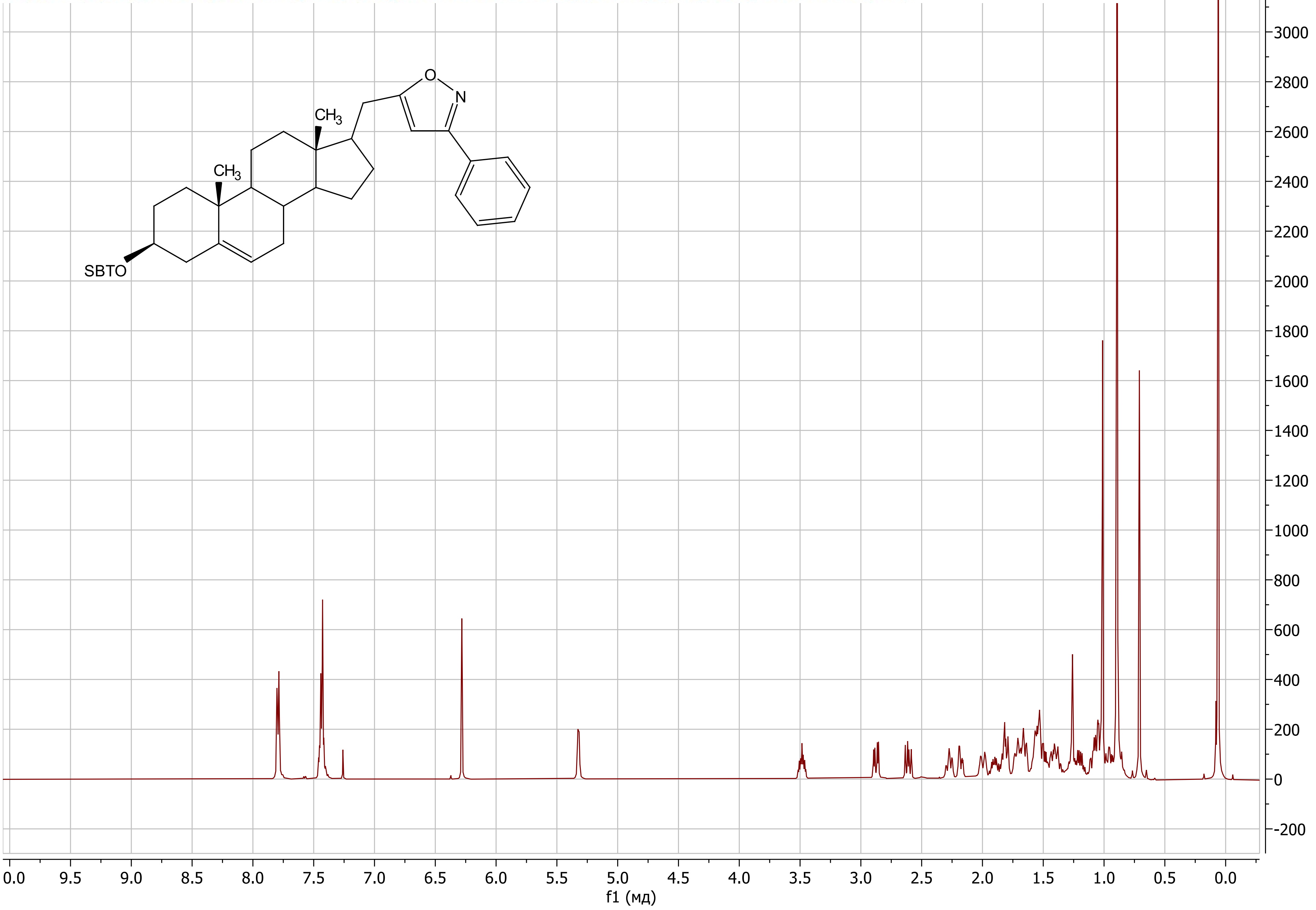

5-(((17*R*)-3β-((*tert*-Butyldimethylsilyl)oxy)-androst-5-en-17-yl)methyl)-3-phenylisoxazole (**23e**)

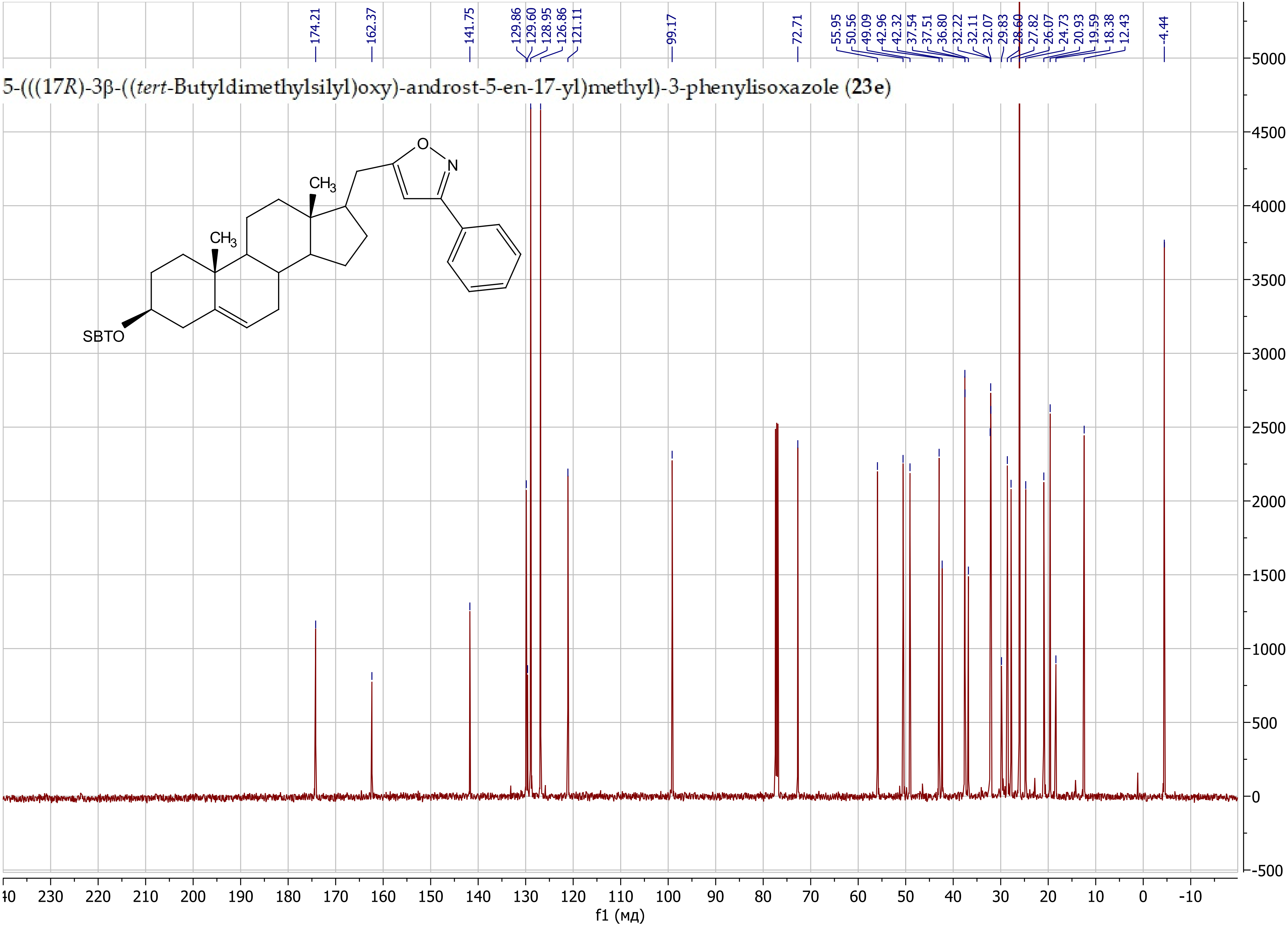

5-(((17*R*)-3β-((*tert*-Butyldimethylsilyl)oxy)-androst-5-en-17-yl)methyl)-3-(pyridin-3-yl)isoxazole (**23f**)

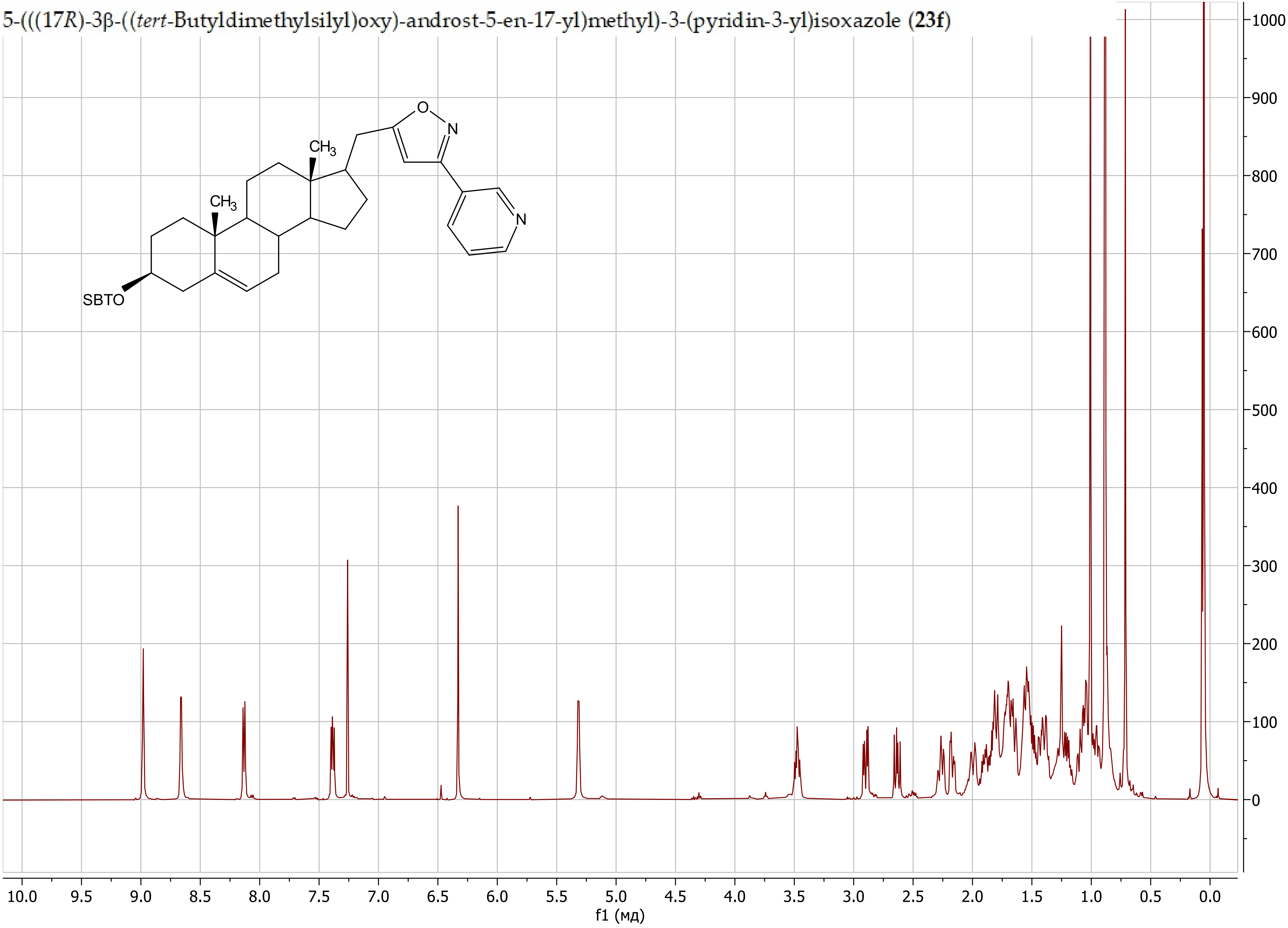

5-(((17*R*)-3β-((*tert*-Butyldimethylsilyl)oxy)-androst-5-en-17-yl)methyl)-3-(pyridin-3-yl)isoxazole (**23f**)

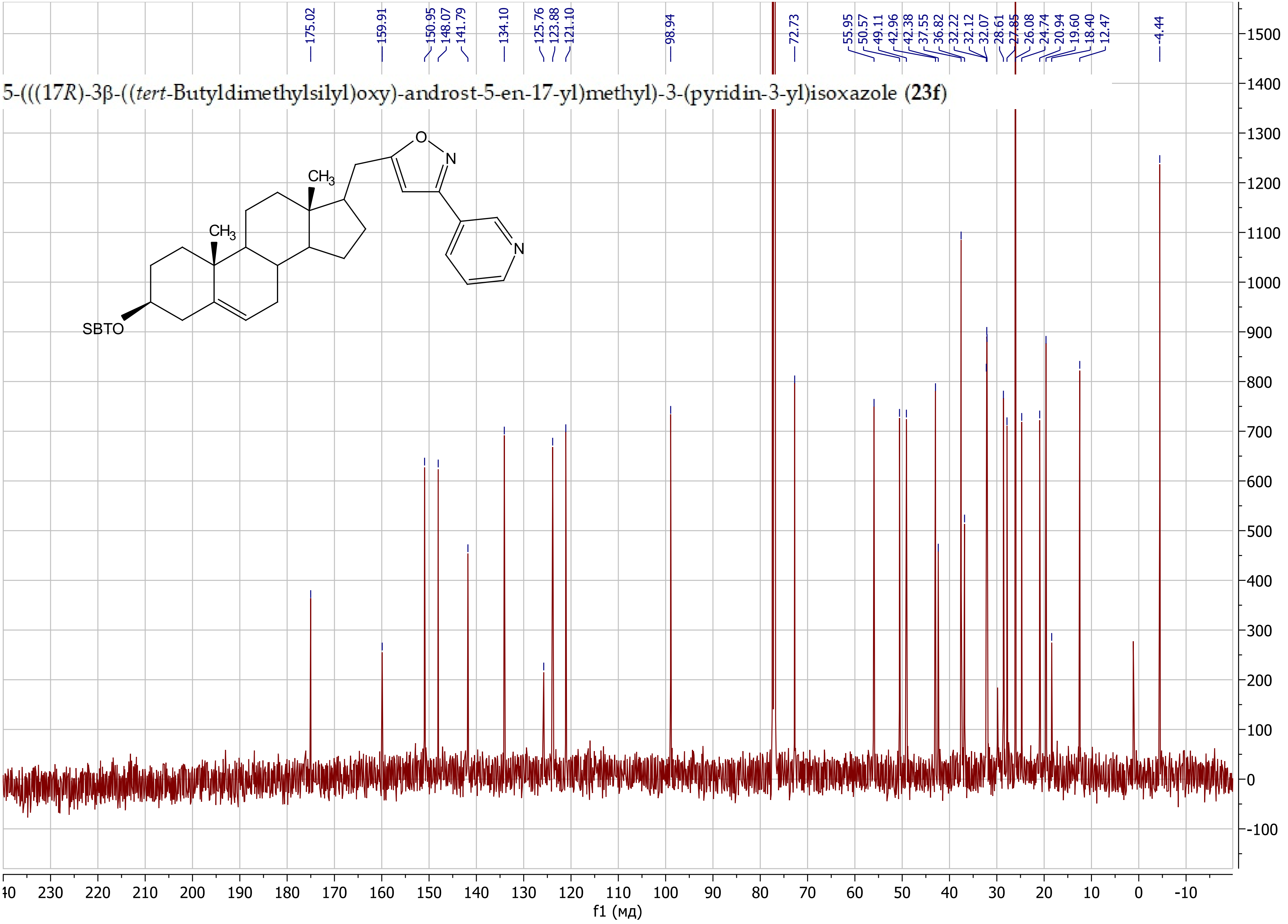

5-(((17*R*)-3β-((*tert*-Butyldimethylsilyl)oxy)-androst-5-en-17-yl)methyl)-3-(2-fluorophenyl)isoxazole (**23g**)

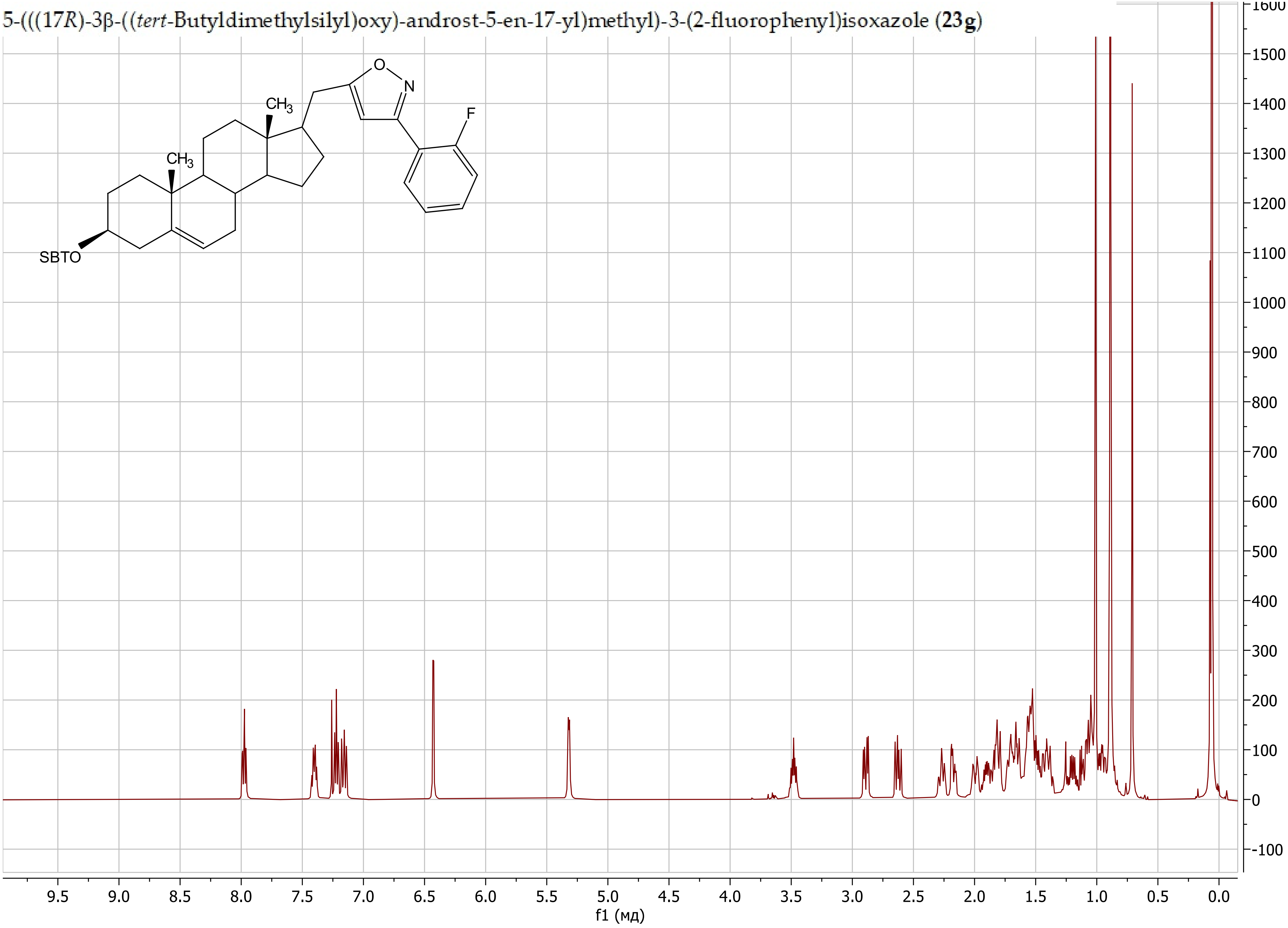

5-(((17*R*)-3β-((*tert*-Butyldimethylsilyl)oxy)-androst-5-en-17-yl)methyl)-3-(2-fluorophenyl)isoxazole (**23g**)

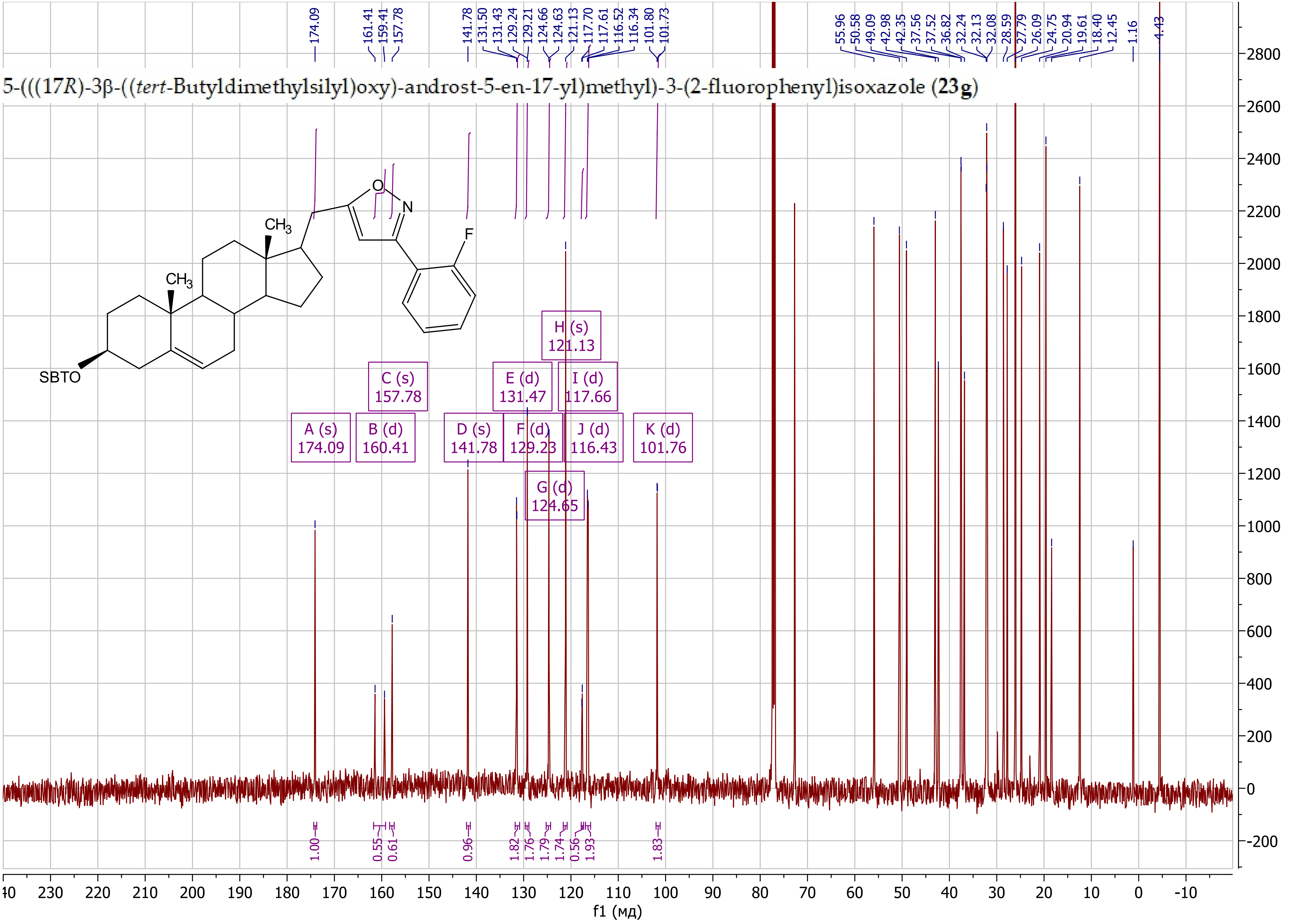

5-(((17*R*)-3β-((*tert*-Butyldimethylsilyl)oxy)-androst-5-en-17-yl)-3-(2-((tetrahydro-2*H*-pyran-2-yl)oxy)propan-2-yl)isoxazole (**23h**)

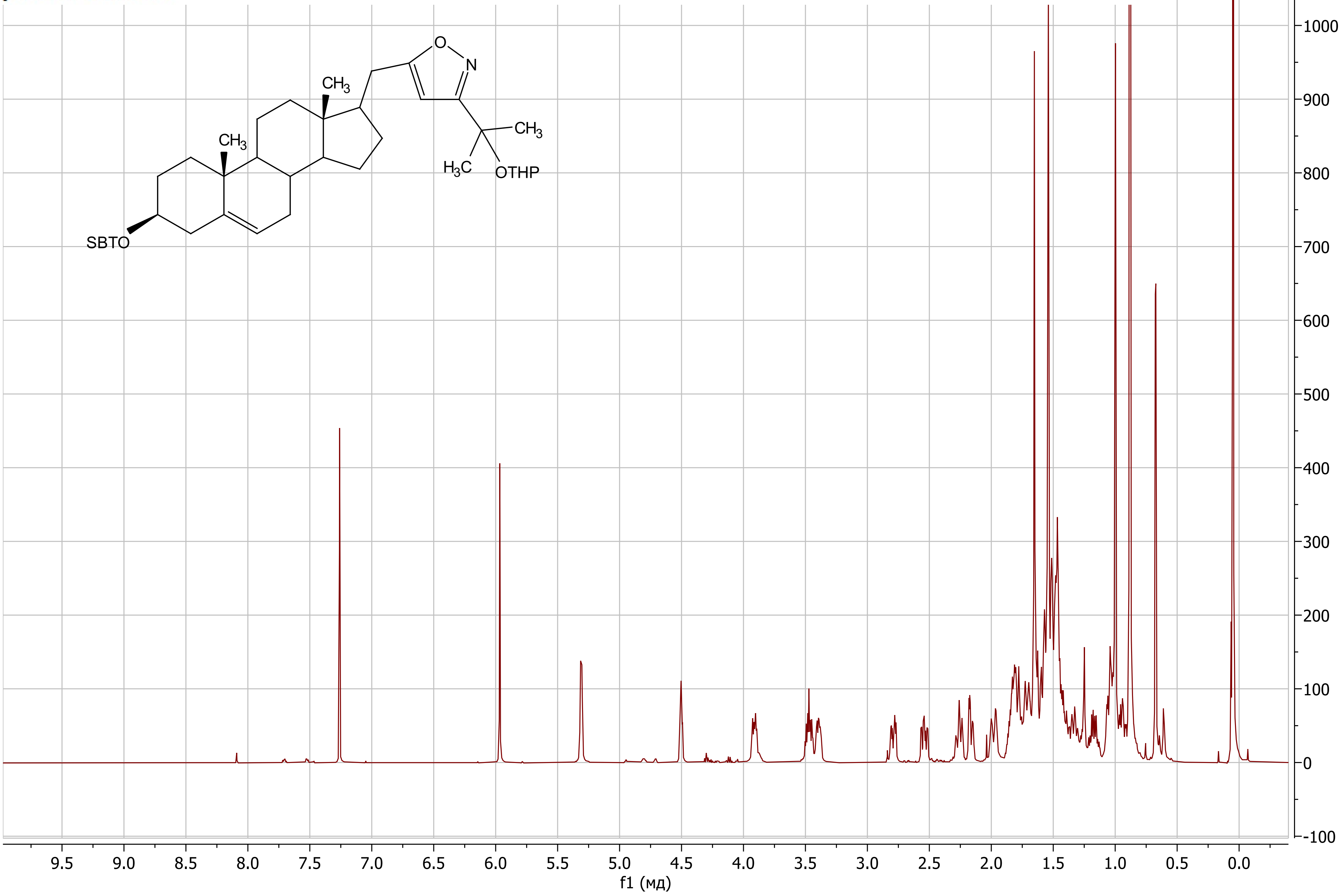

5-(((17*R*)-3β-((*tert*-Butyldimethylsilyl)oxy)-androst-5-en-17-yl)-3-(2-((tetrahydro-2*H*-pyran-2-yl)oxy)propan-2-yl)isoxazole (**23h**)

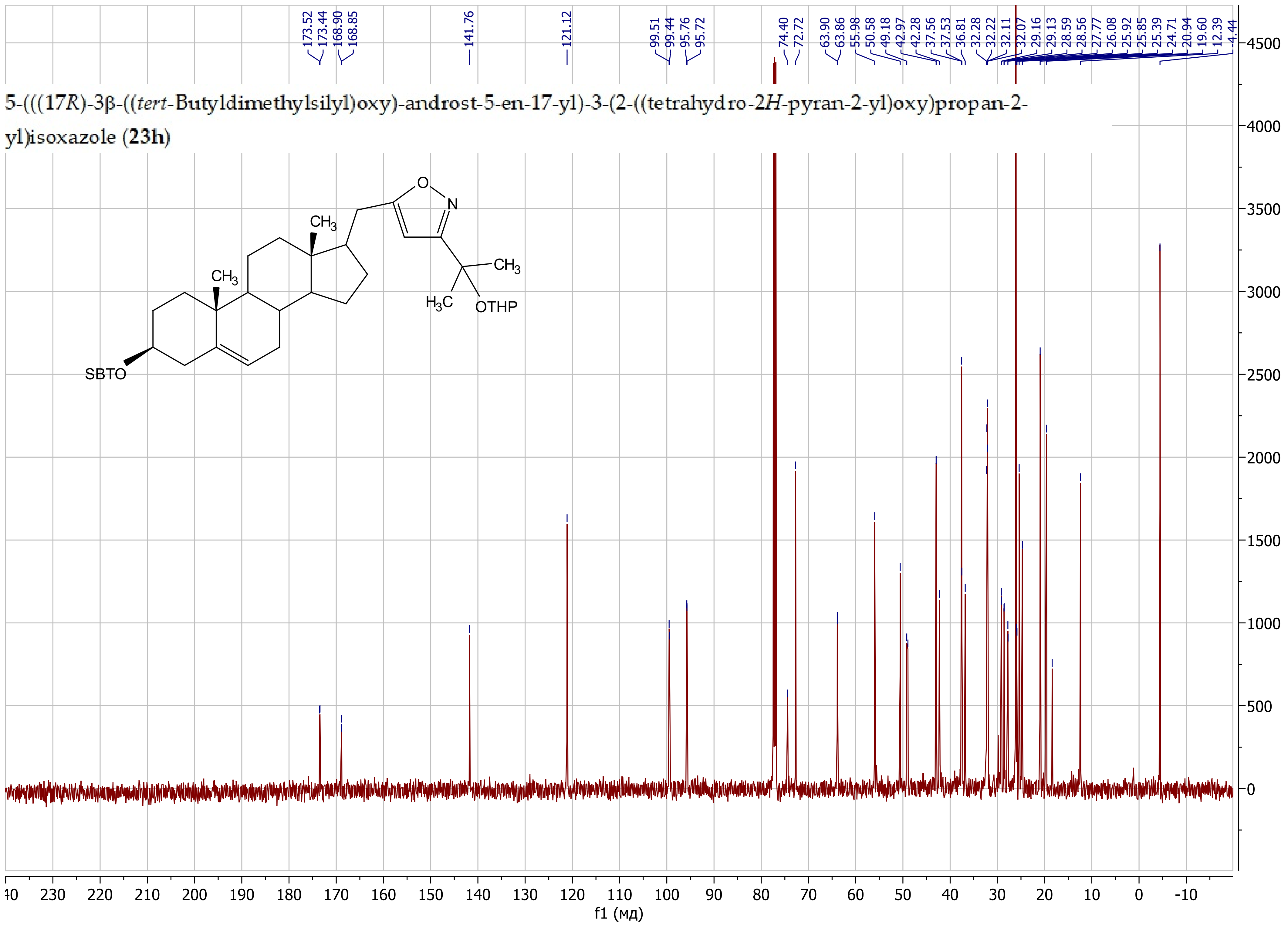

5-(((17*R*)-3β-((*tert*-Butyldimethylsilyl)oxy)-androst-5-en-17-yl)-3-(((tetrahydro-2*H*-pyran-2-yl)oxy)methyl)isoxazole (**23i**)

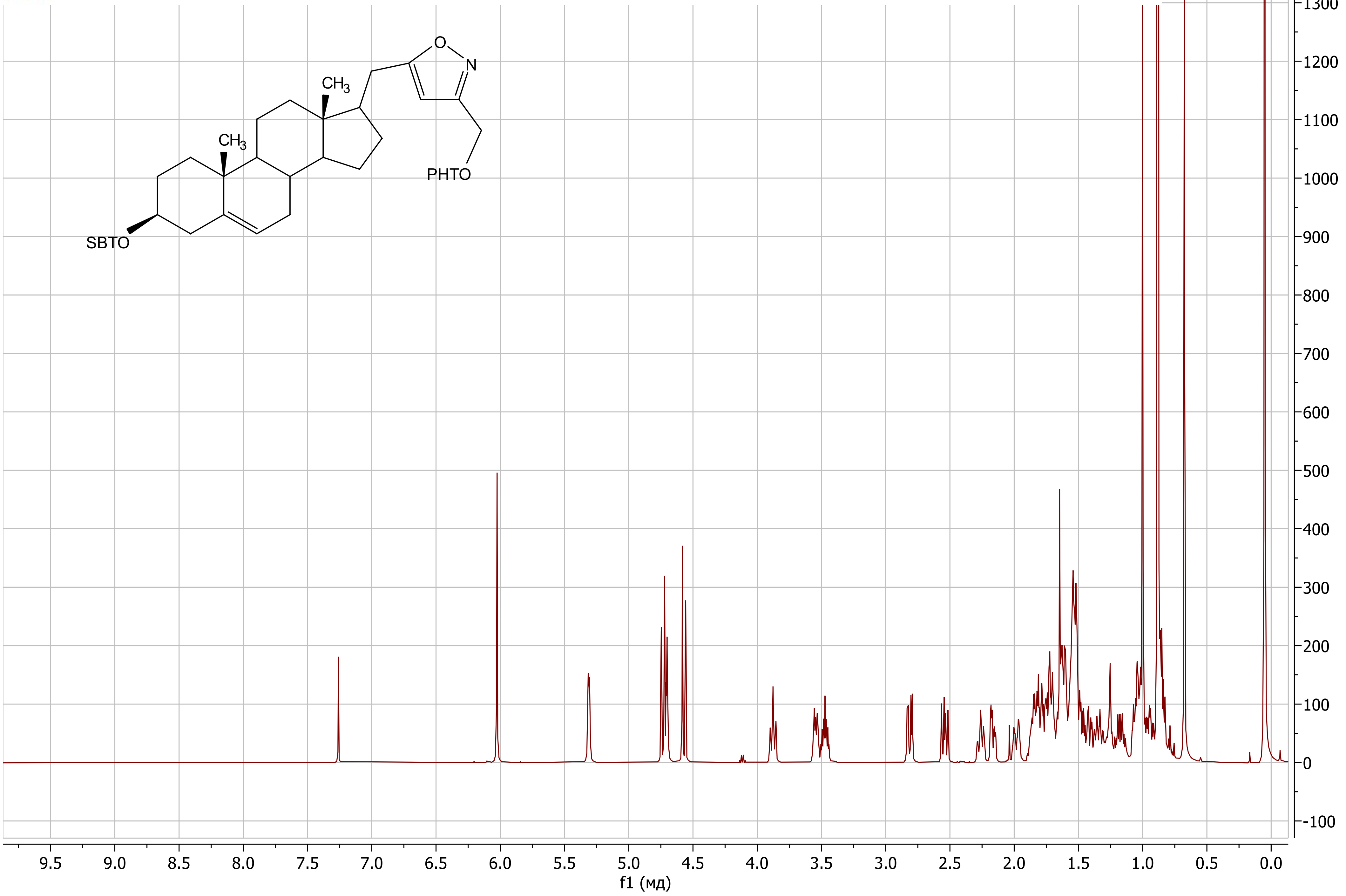

5-(((17*R*)-3β-((*tert*-Butyldimethylsilyl)oxy)-androst-5-en-17-yl)-3-(((tetrahydro-2*H*-pyran-2-yl)oxy)methyl)isoxazole (**23i**)

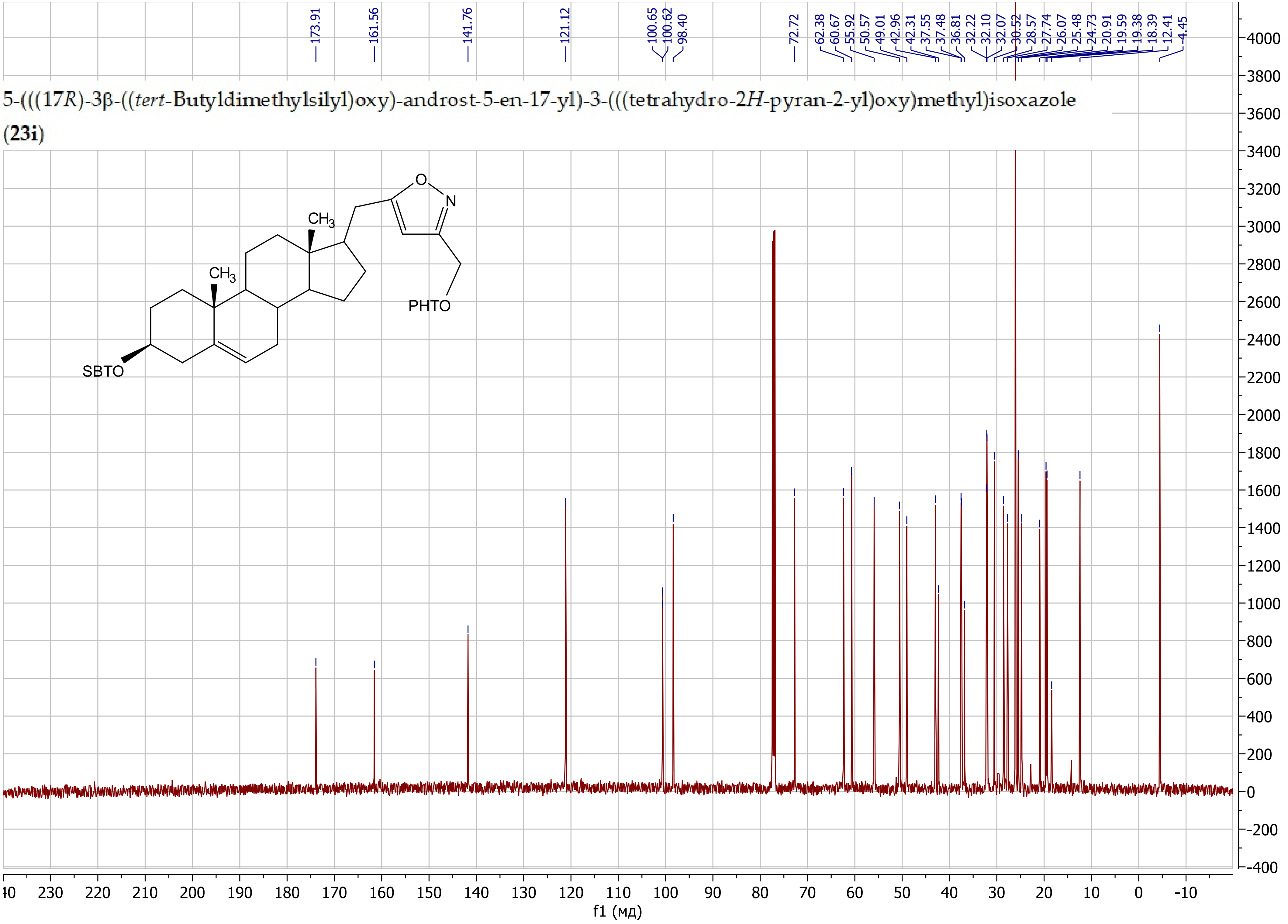

(17*R*)-(Isoxazol-5-ylmethyl)-androst-5-en-3β-ol (**24a**)

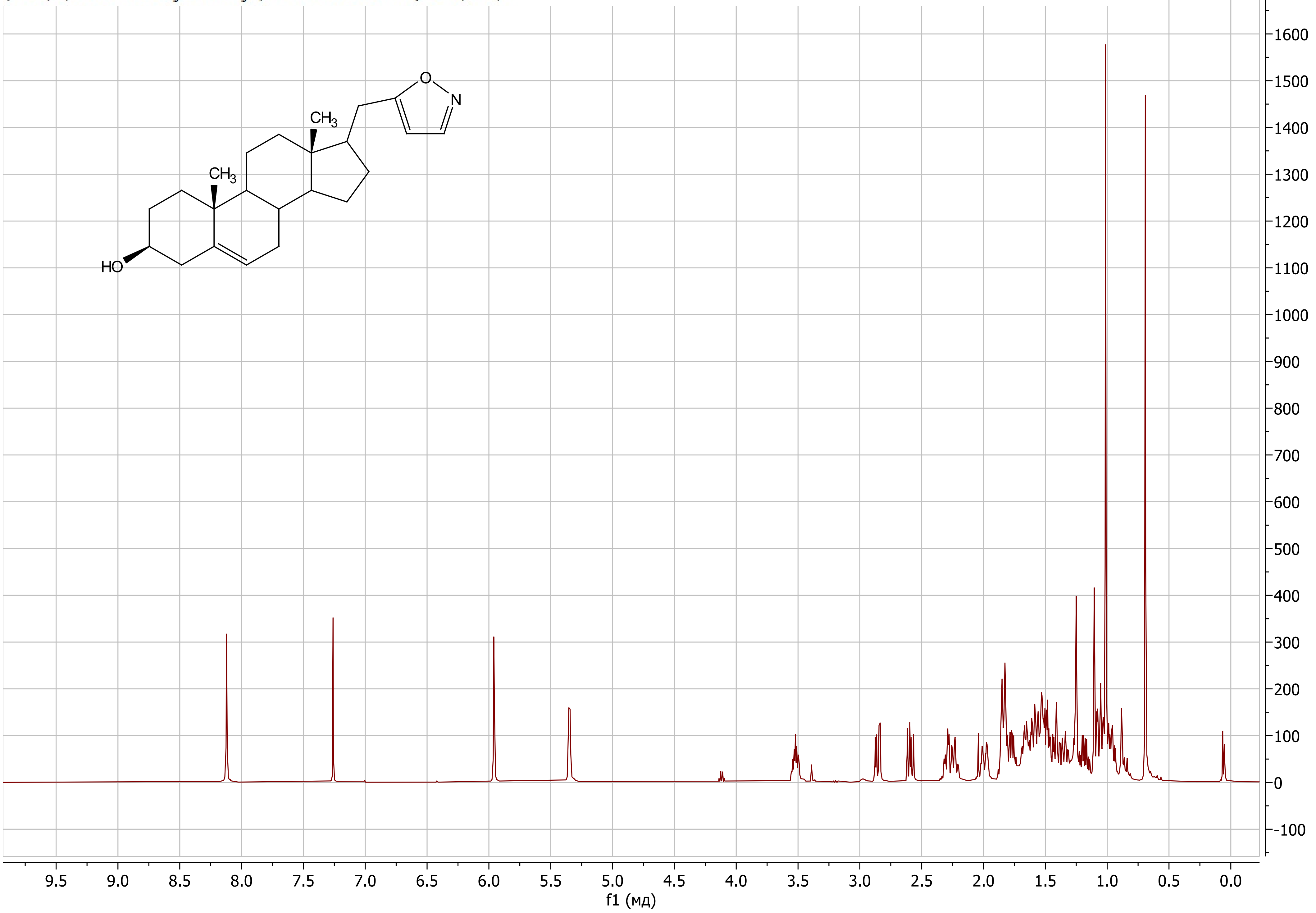

(17*R*)-(Isoxazol-5-ylmethyl)-androst-5-en-3β-ol (**24a**)

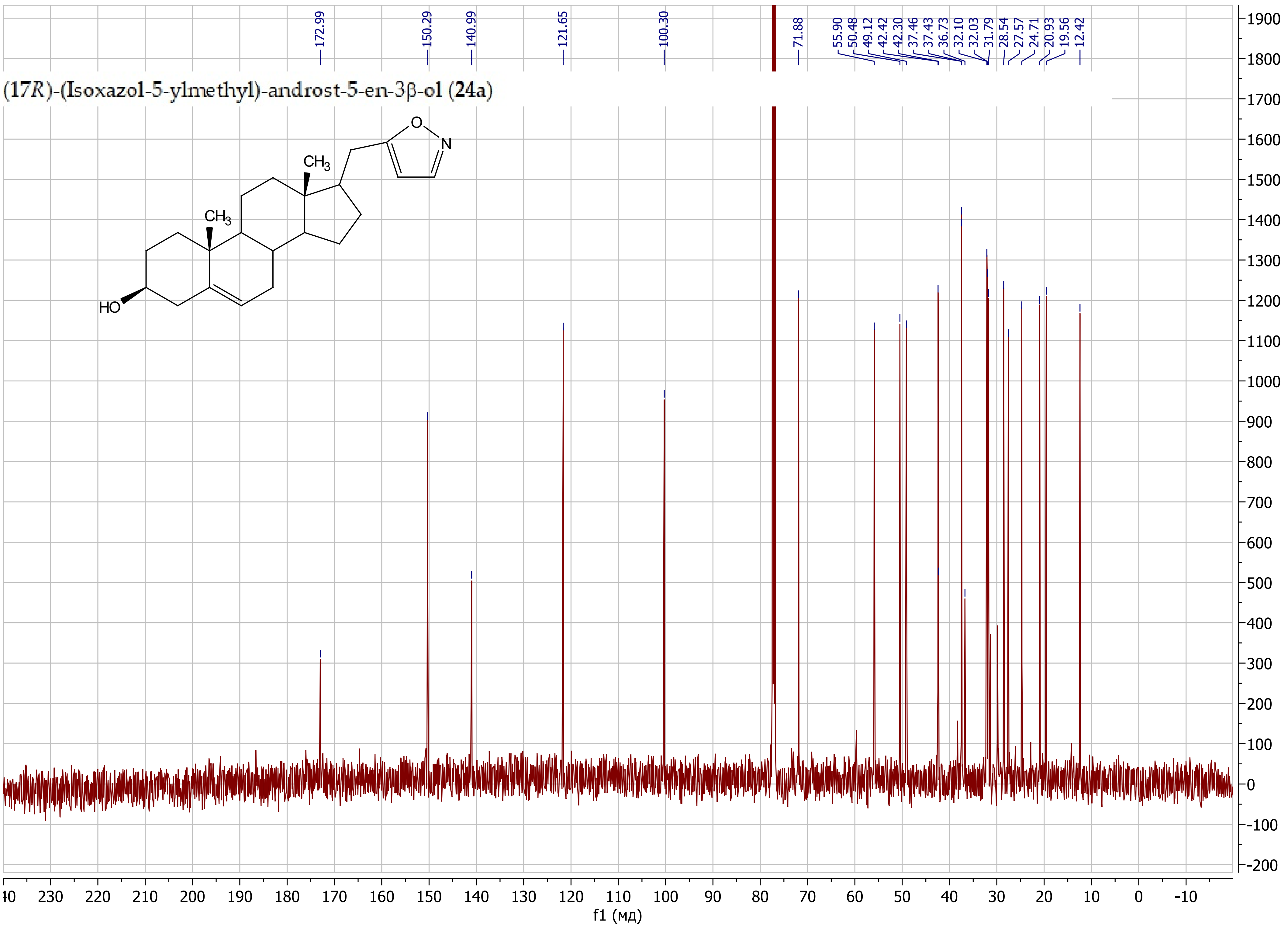

(17*R*)-17-((3-isopropylisoxazol-5-yl)methyl)-androst-5-en-3β-ol (**24b**)

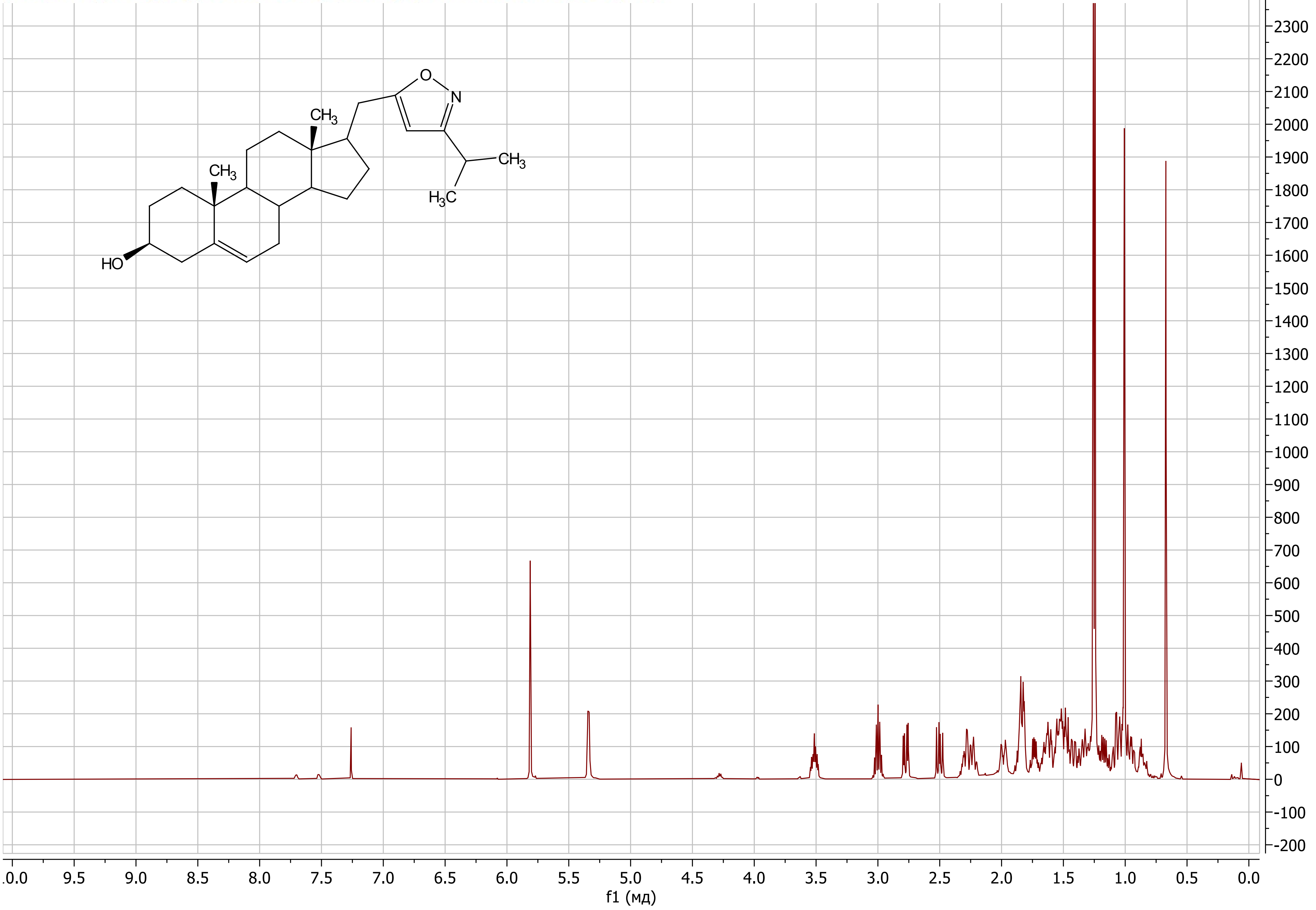

(17*R*)-17-((3-isopropylisoxazol-5-yl)methyl)-androst-5-en-3β-ol (**24b**)

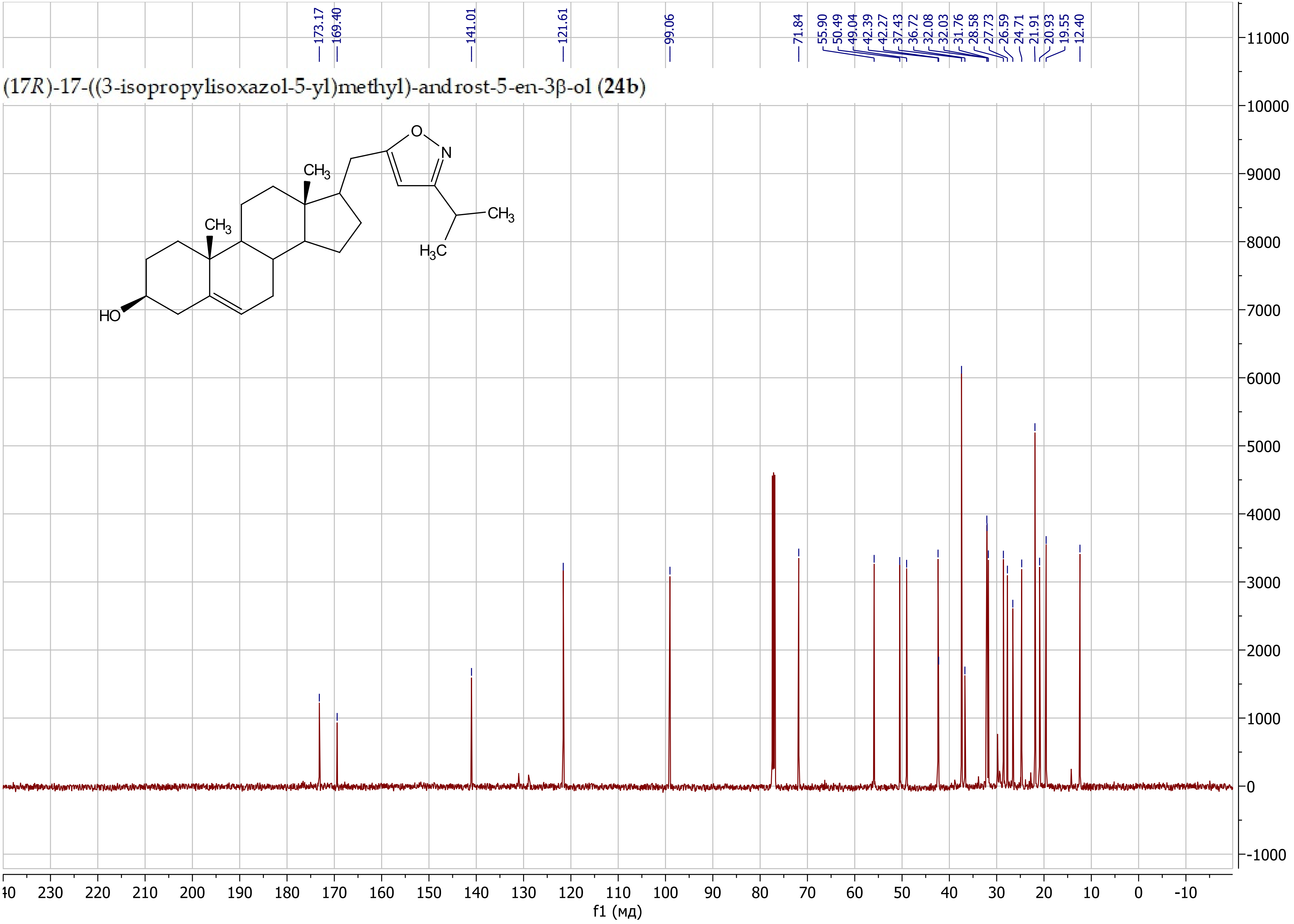

17β-((3-Butylisoxazol-5-yl)methyl)-androst-5-en-3β-ol (**24d**)

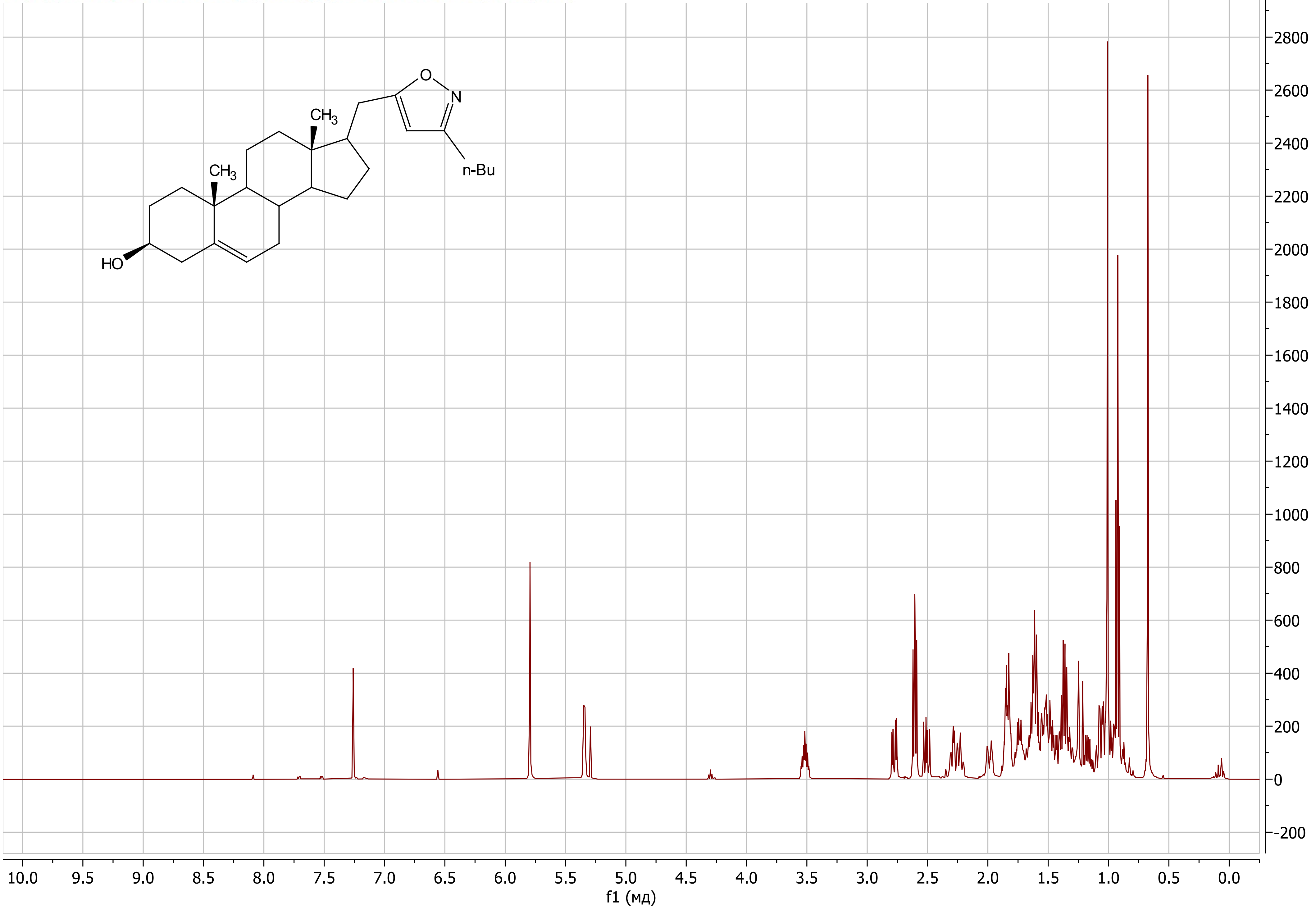

17β-((3-Butylisoxazol-5-yl)methyl)-androst-5-en-3β-ol (**24d**)

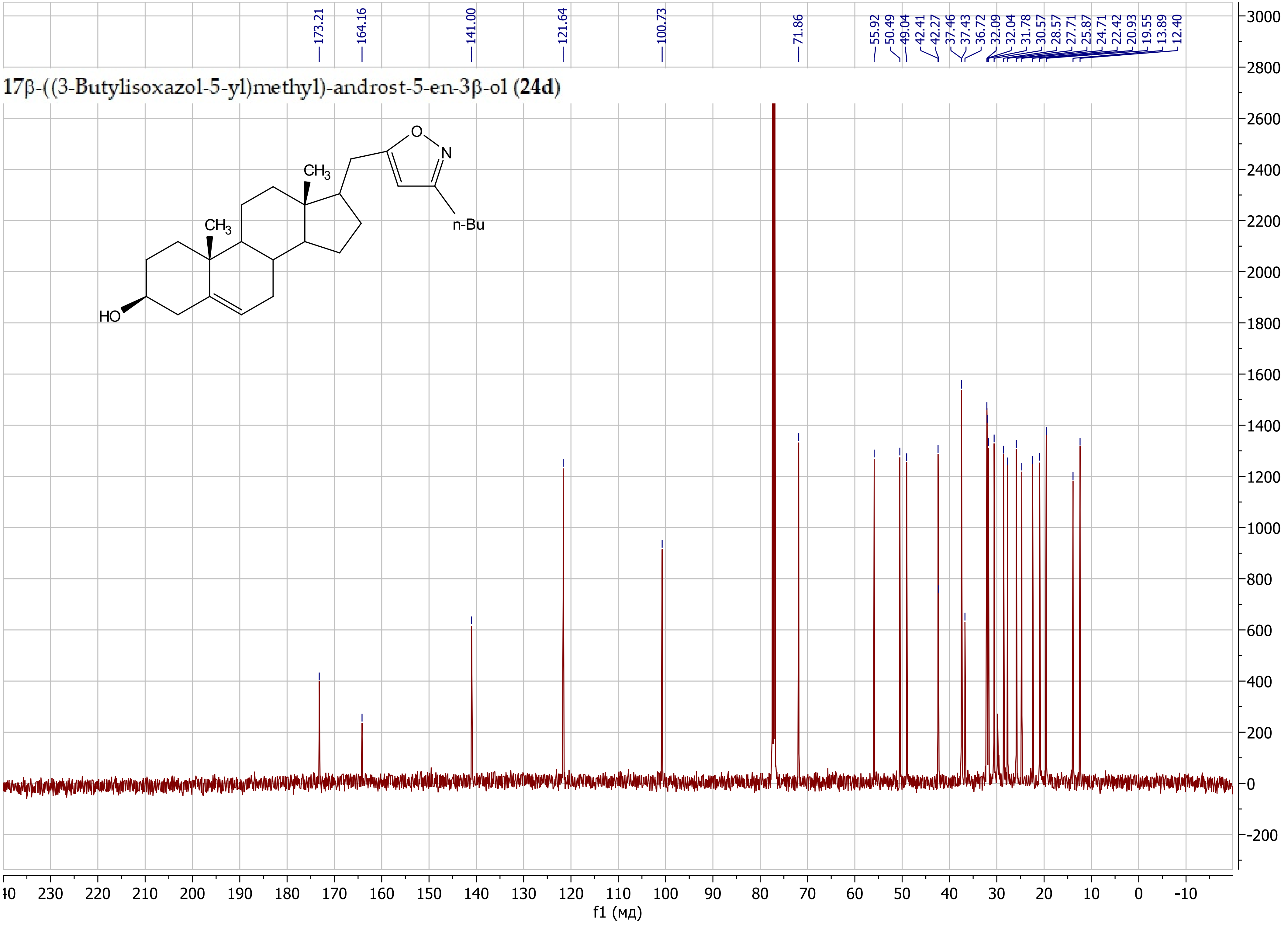

(17*R*)-17-((3-Phenylisoxazol-5-yl)methyl)-androst-5-en-3β-ol (**24e**)

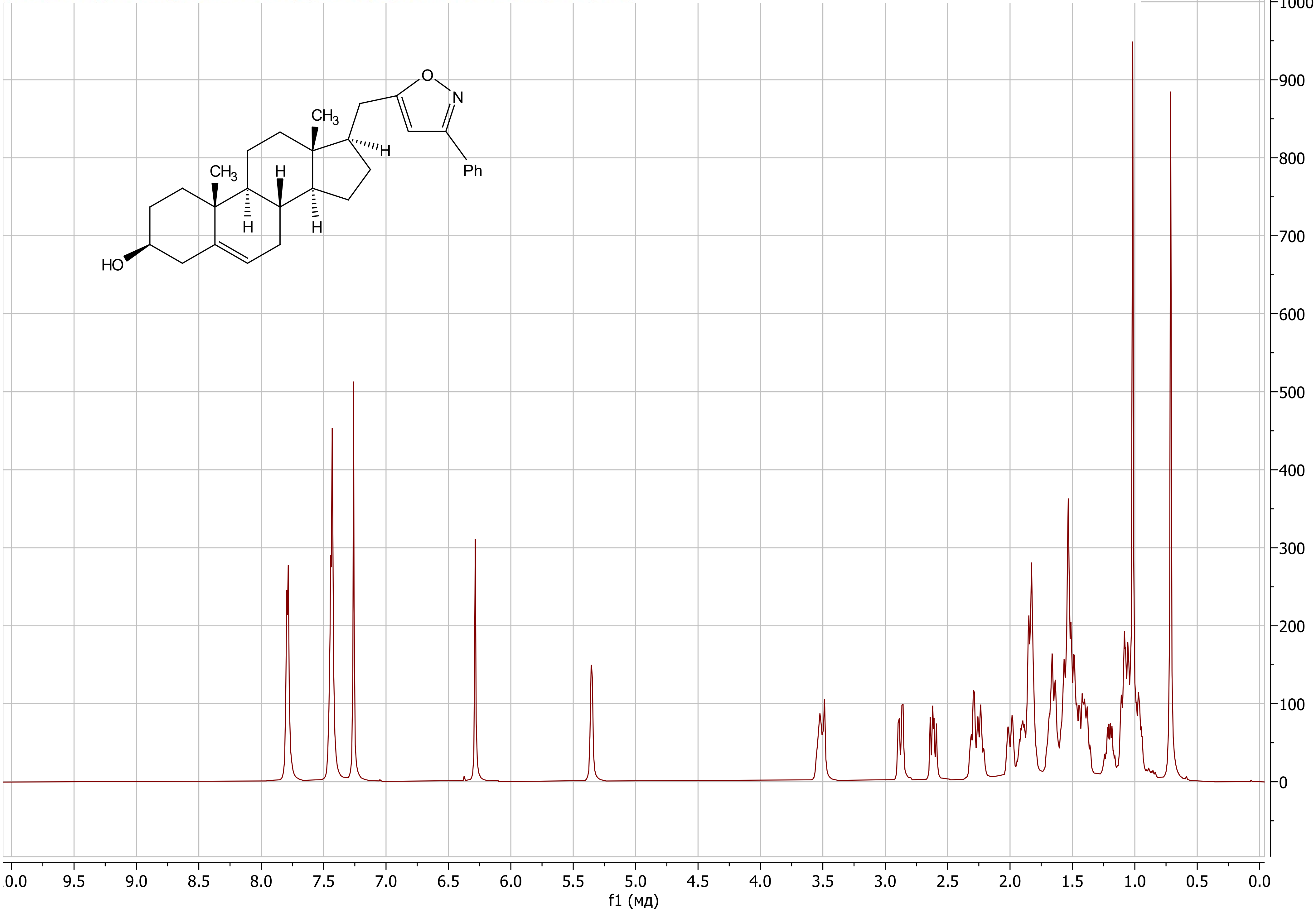

(17*R*)-17-((3-Phenylisoxazol-5-yl)methyl)-androst-5-en-3β-ol (**24e**)

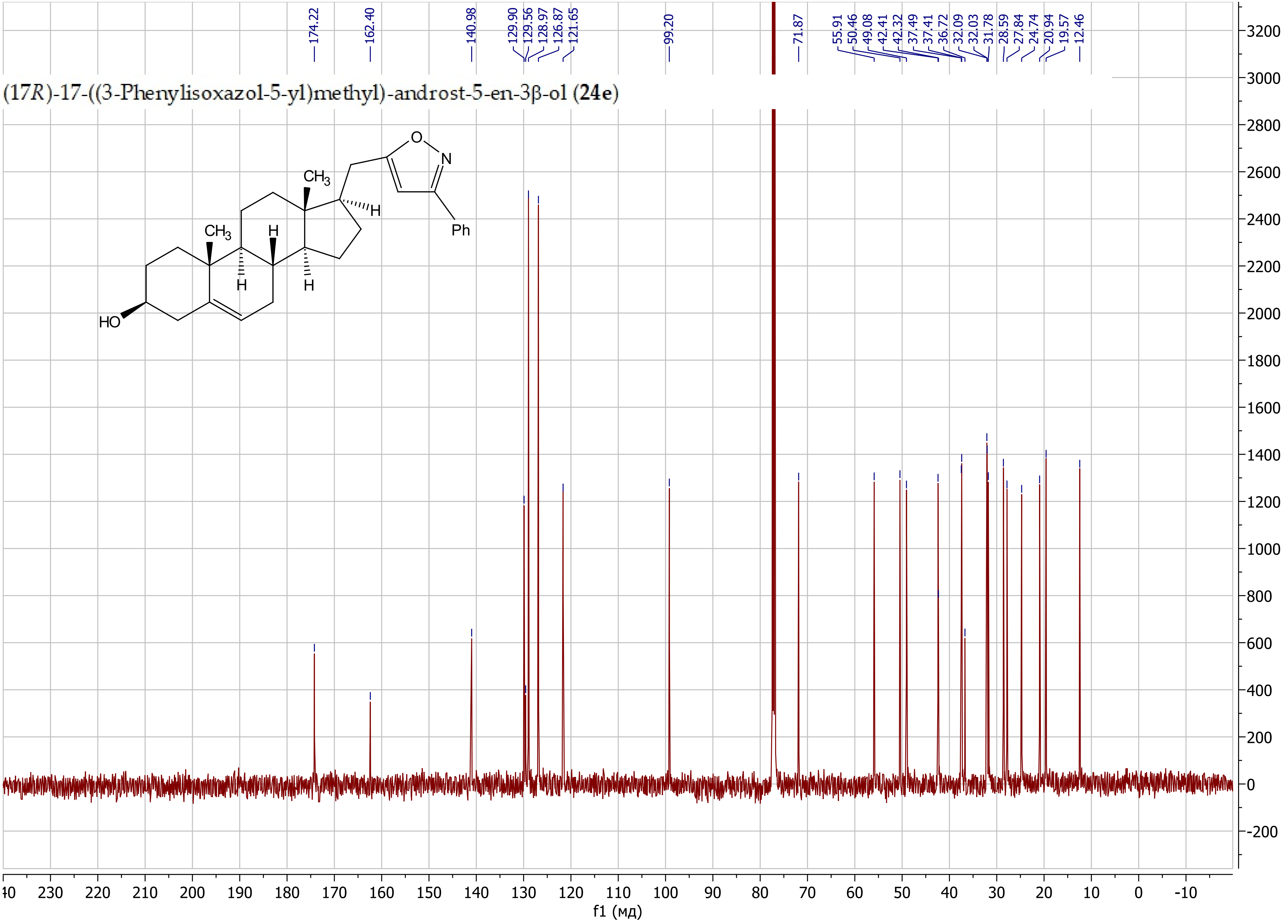

(17*R*)-17-((3-(Pyridin-3-yl)isoxazol-5-yl)methyl)-androst-5-en-3β-ol (**24f**)

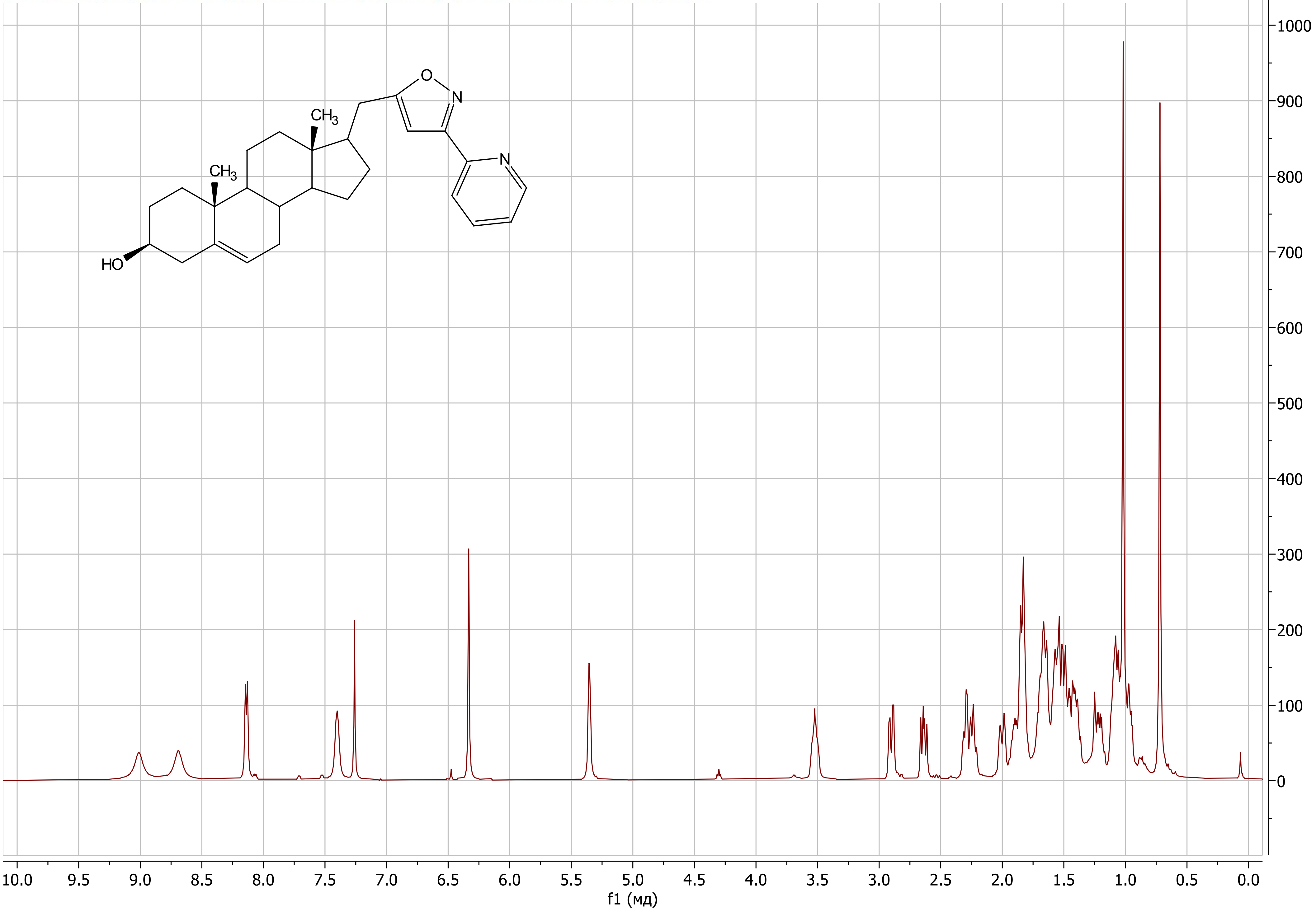

(17*R*)-17-((3-(Pyridin-3-yl)isoxazol-5-yl)methyl)-androst-5-en-3β-ol (**24f**)

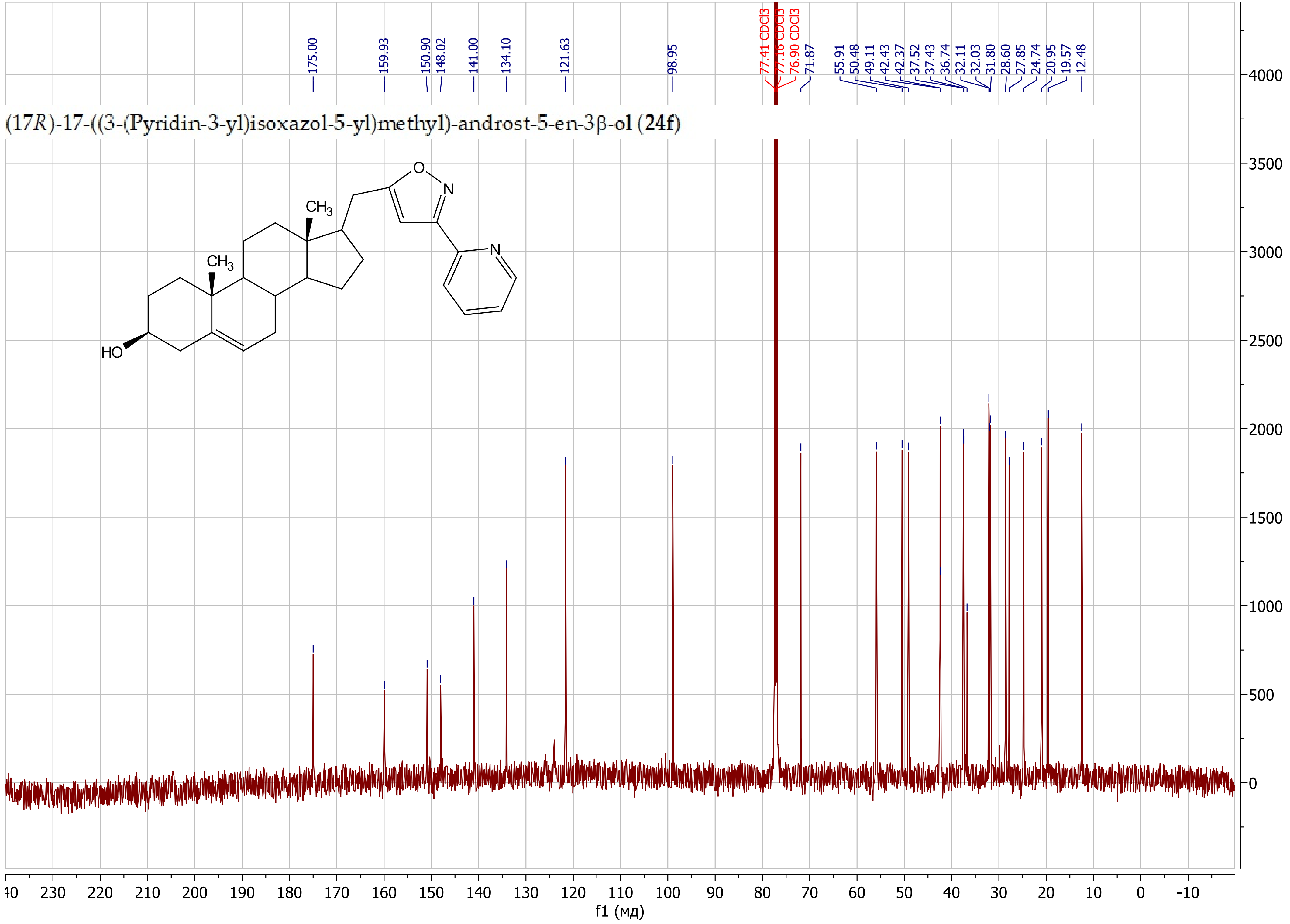

17β-((3-(2-Fluorophenyl)isoxazol-5-yl)methyl)-androst-5-en-3β-ol (**24g**)

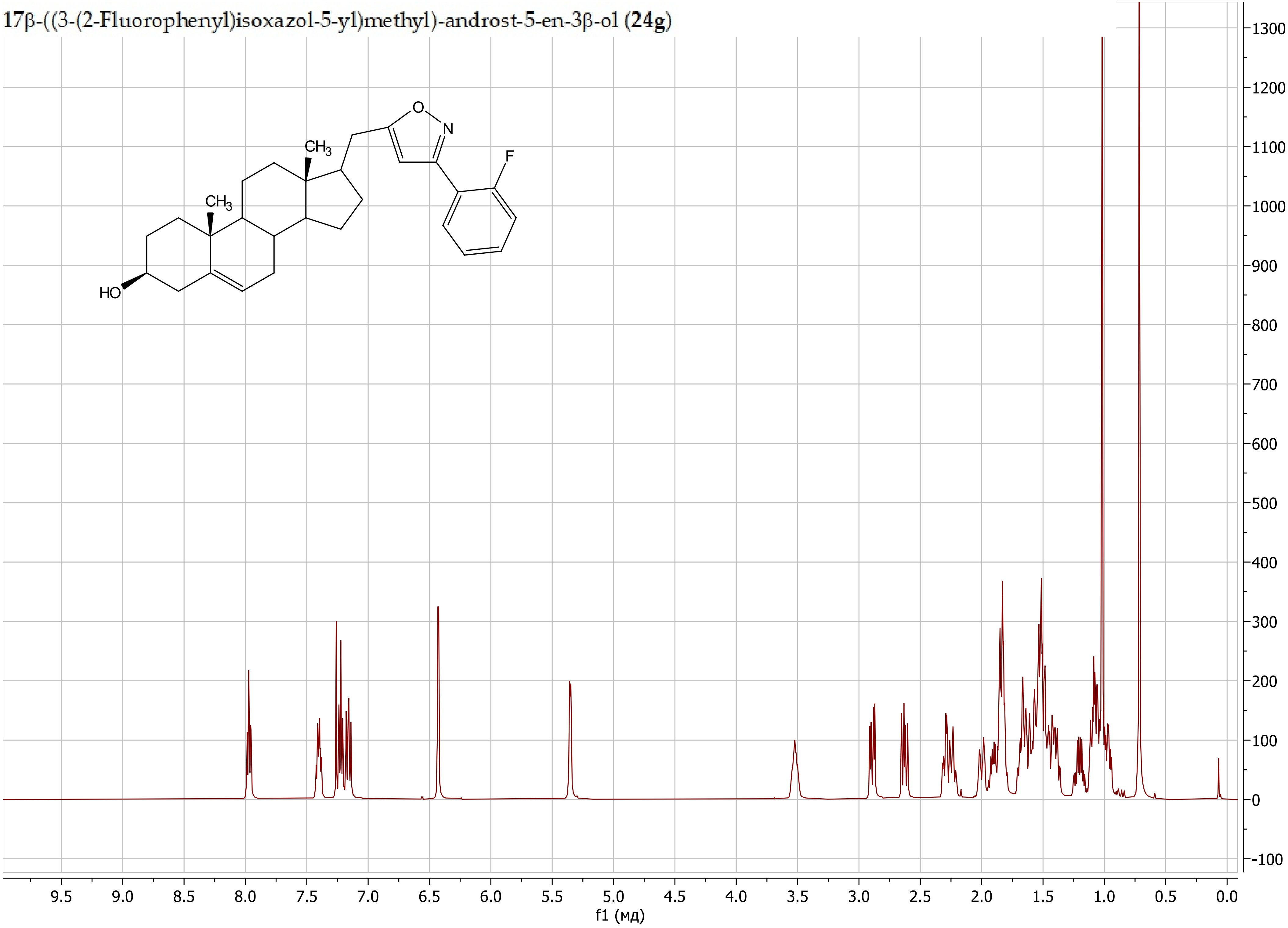

17β-((3-(2-Fluorophenyl)isoxazol-5-yl)methyl)-androst-5-en-3β-ol (**24g**)

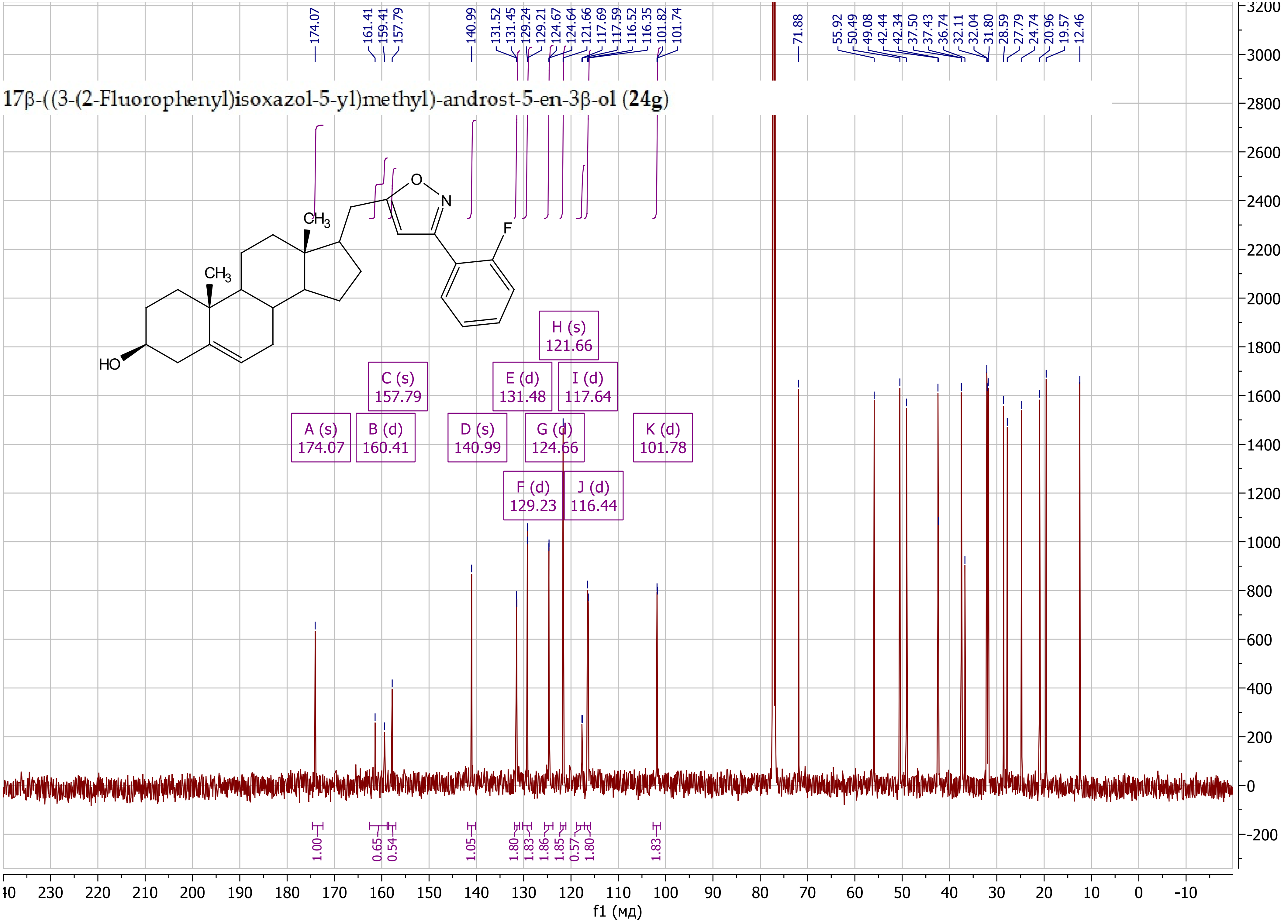

(17*R*)-17-((3-(2-Hydroxypropan-2-yl)isoxazol-5-yl)methyl)-androst-5-en-3β-ol (**24j**)

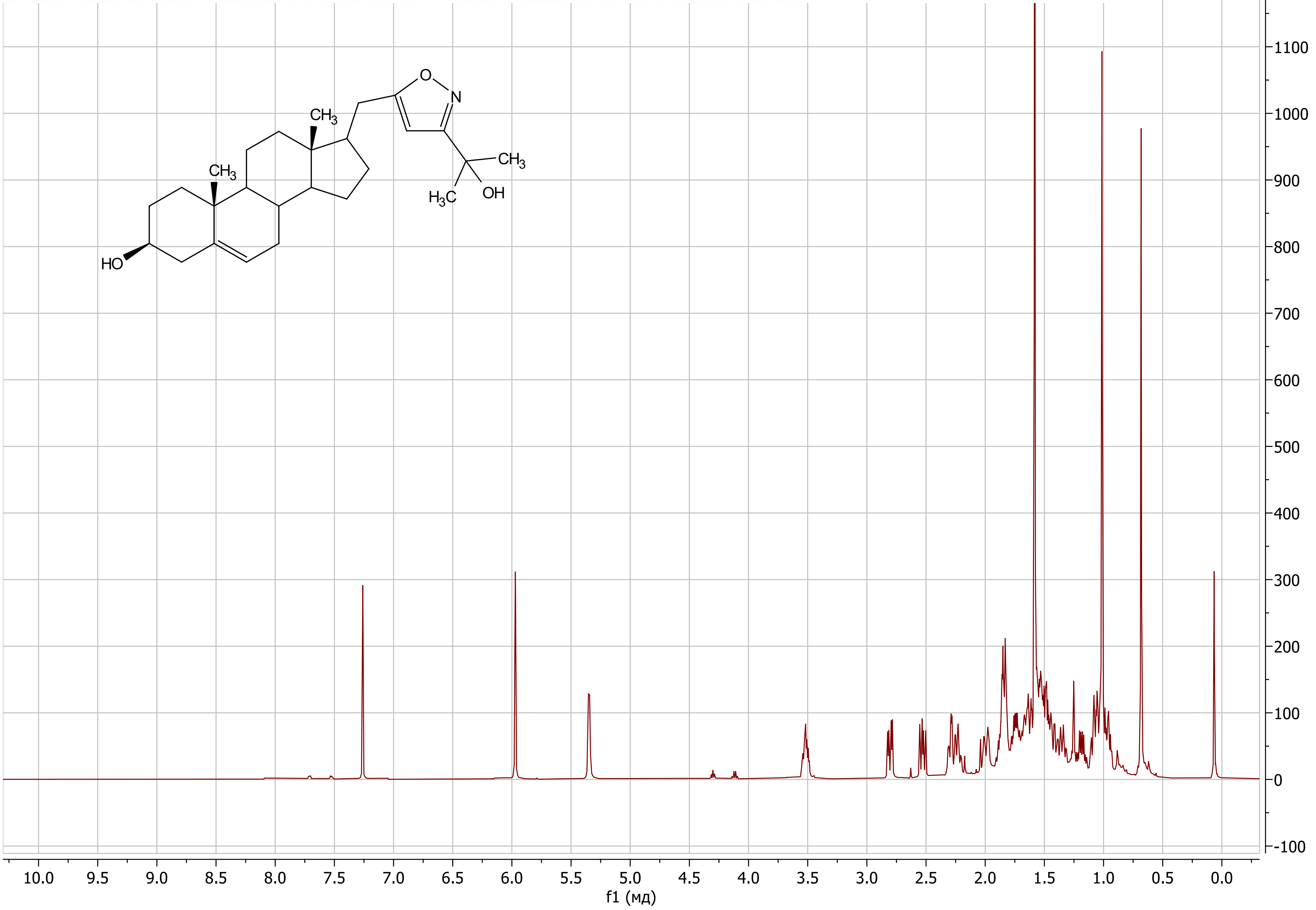

(17*R*)-17-((3-(2-Hydroxypropan-2-yl)isoxazol-5-yl)methyl)-androst-5-en-3β-ol (**24j**)

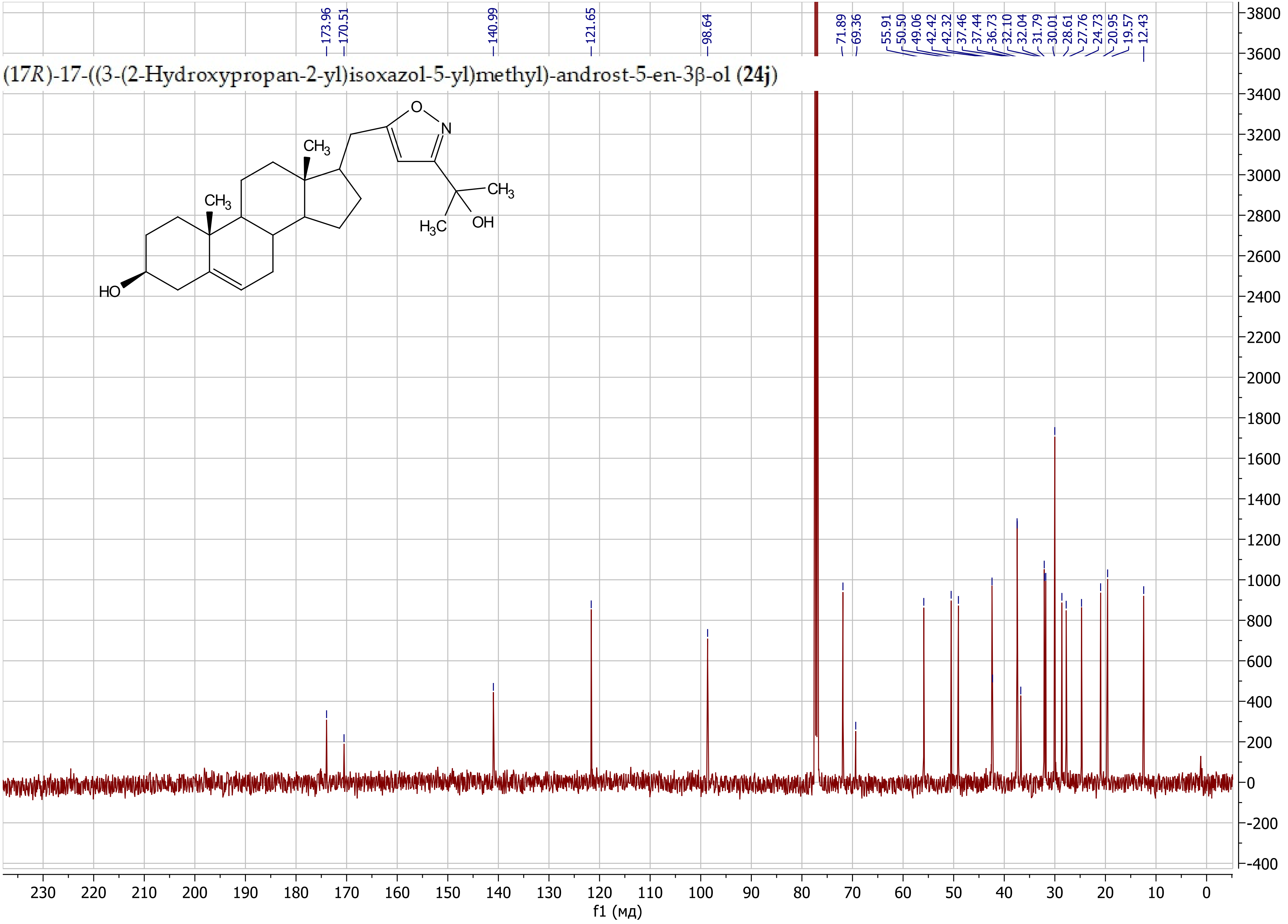

(17*R*)-17-((3-Cyclopropylisoxazol-5-yl)methyl)-androst-5-en-3β-ol (**24c**)

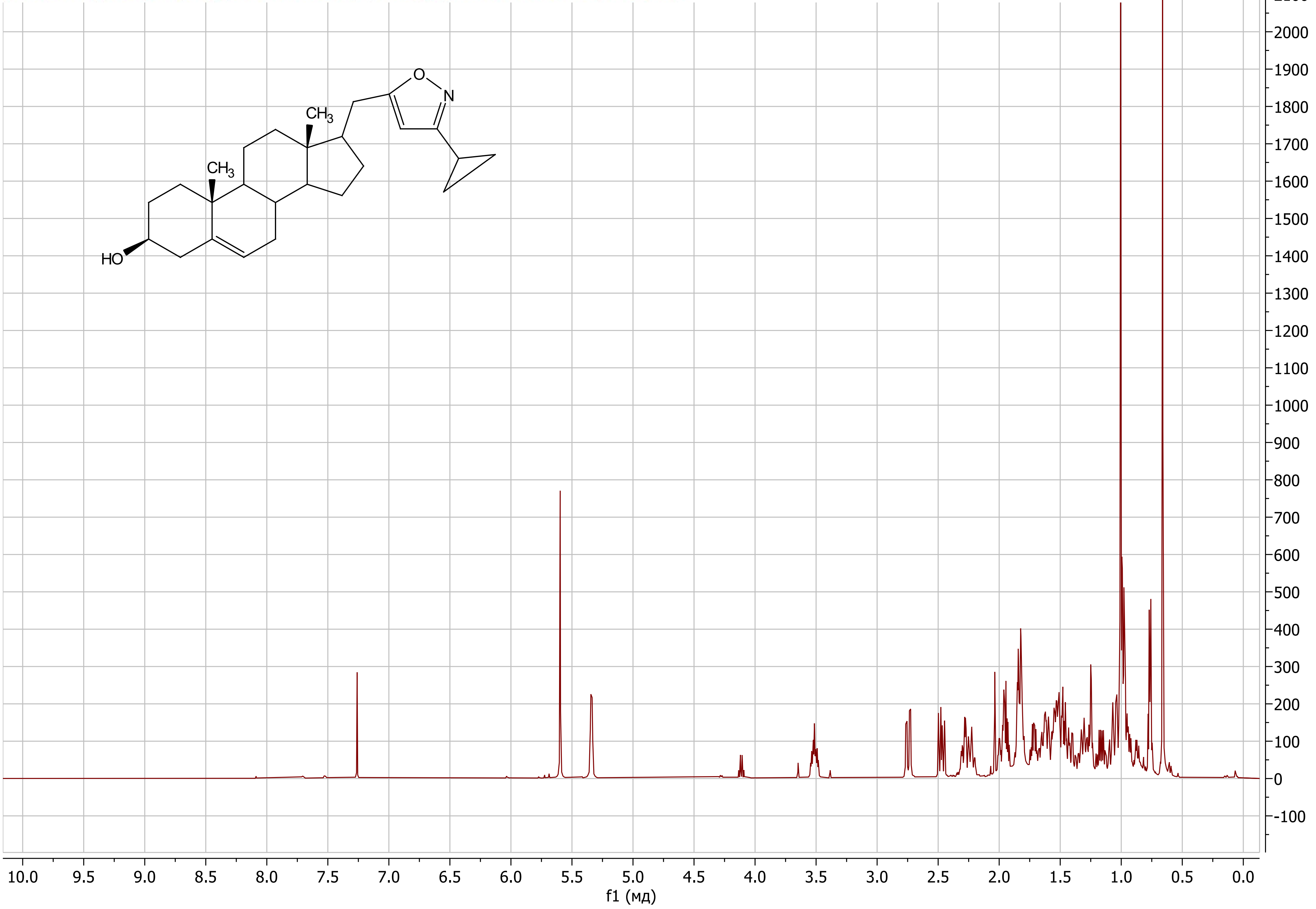

(17*R*)-17-((3-Cyclopropylisoxazol-5-yl)methyl)-androst-5-en-3β-ol (24c)

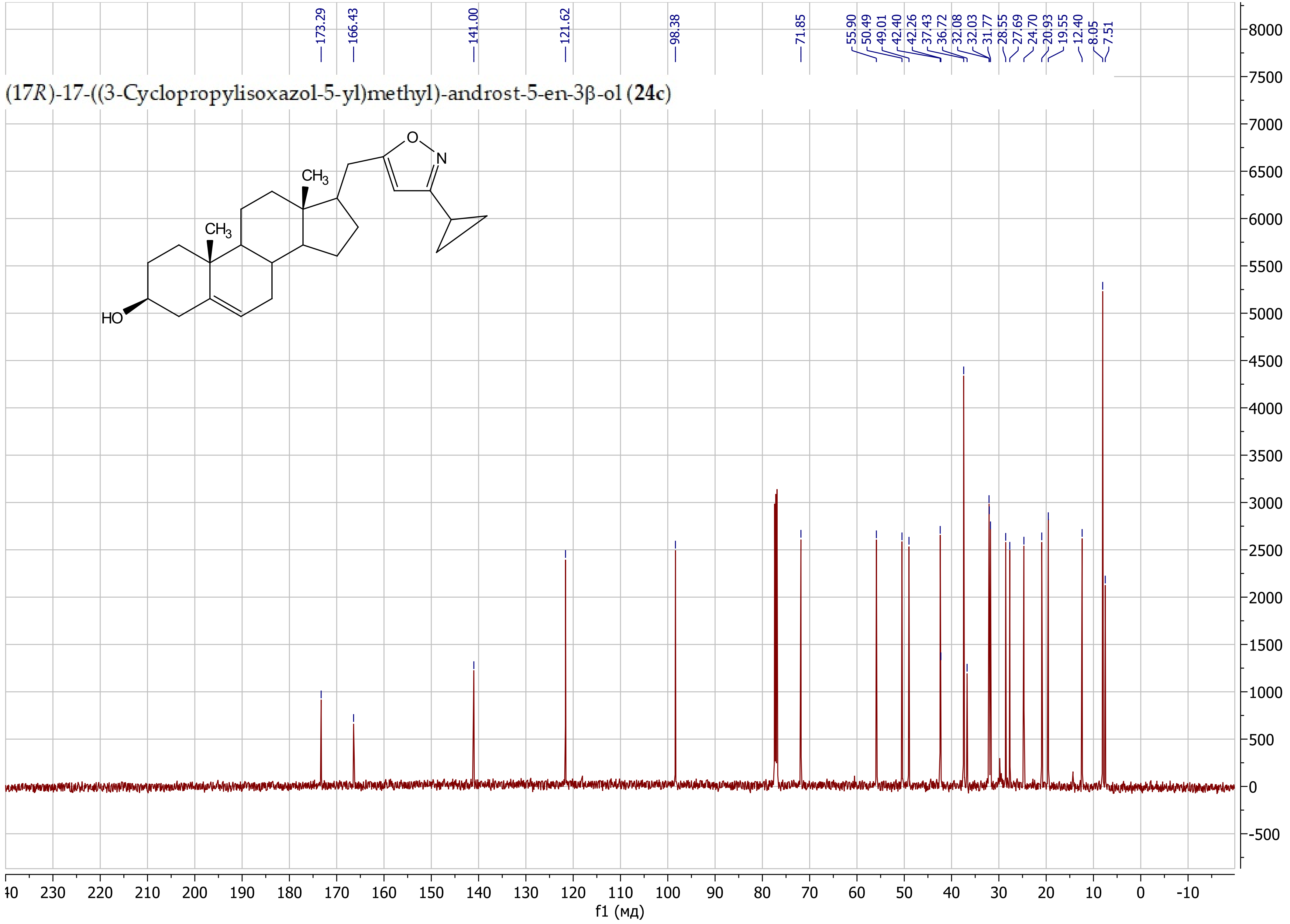

(*E*)-4-Amino-1-((17*R*)-3β-((*tert*-butyldimethylsilyl)oxy)-androst-5-en-17-yl)-5-methyl-2-oxohex-3-en-3-yl 1*H*-imidazole-1-carboxylate (**26b**)

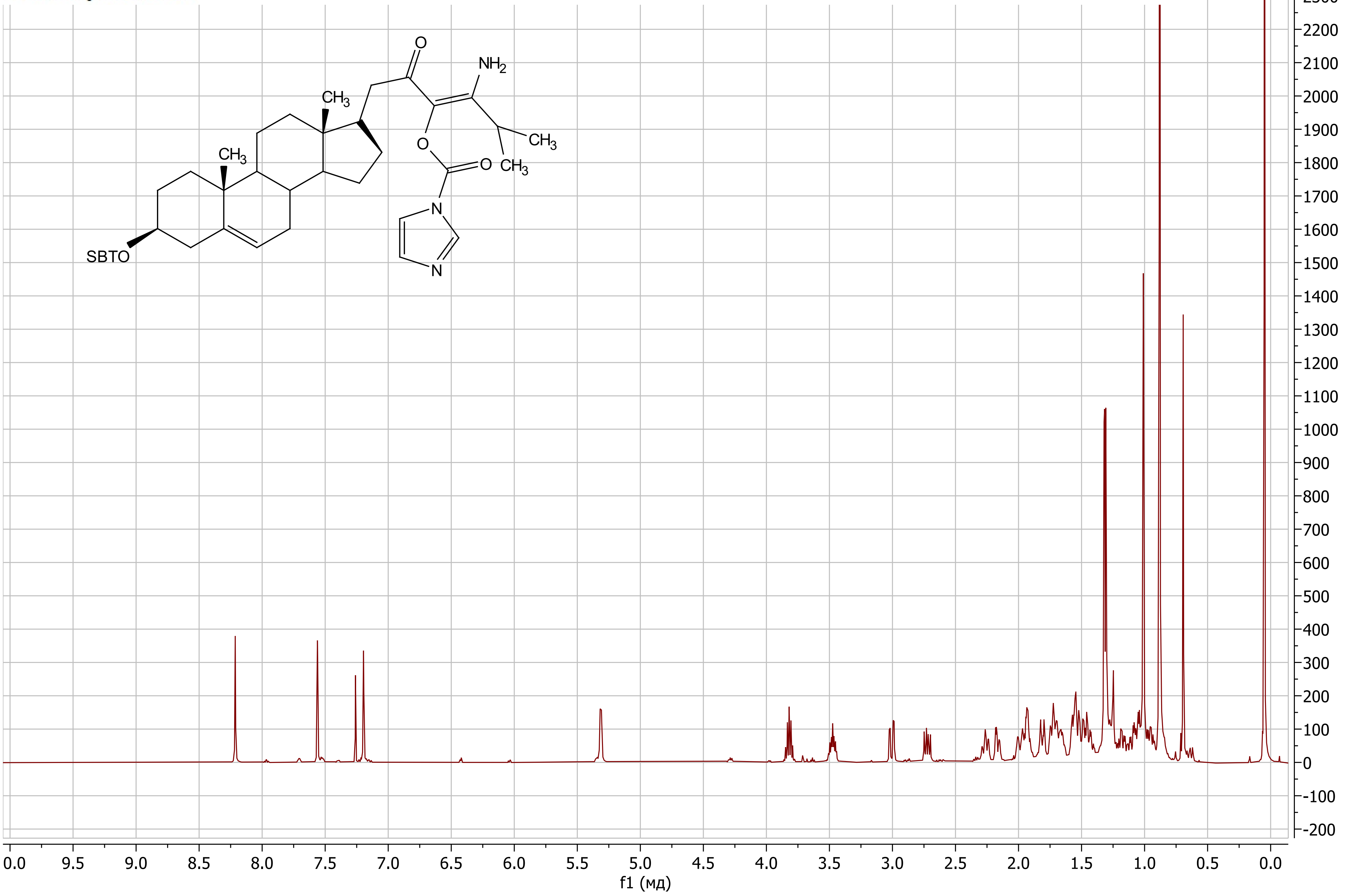

(*E*)-4-Amino-1-((17*R*)-3β-((*tert*-butyldimethylsilyl)oxy)-androst-5-en-17-yl)-5-methyl-2-oxohex-3-en-3-yl 1*H*-imidazole-1-carboxylate (**26b**)

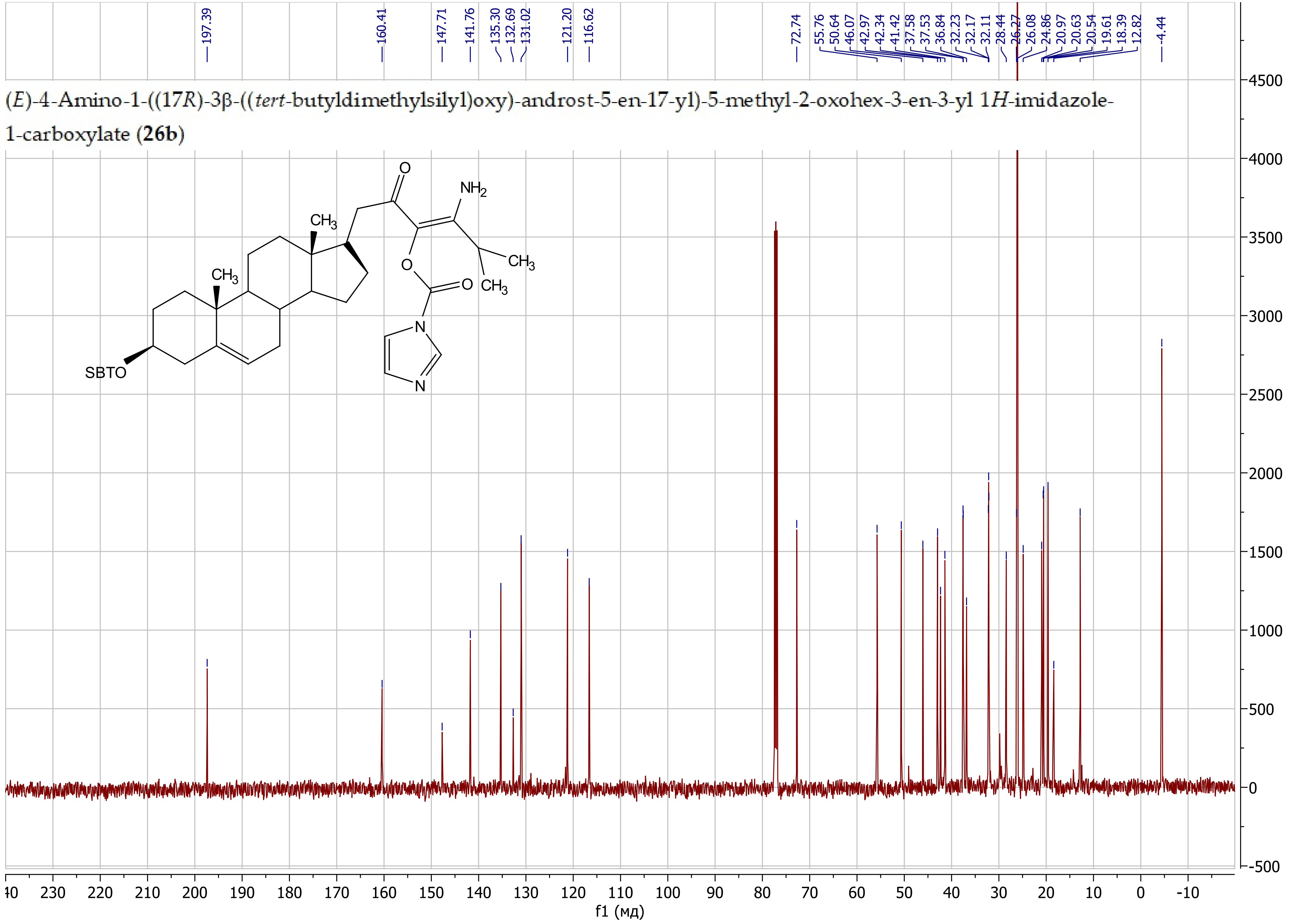

(*E*)-1-Amino-4-((17*R*)-3β-((*tert*-butyldimethylsilyl)oxy)-androst-5-en-17-yl)-1-cyclopropyl-3-oxobut-1-en-2-yl 1*H*-imidazole-1-carboxylate (**26c**)

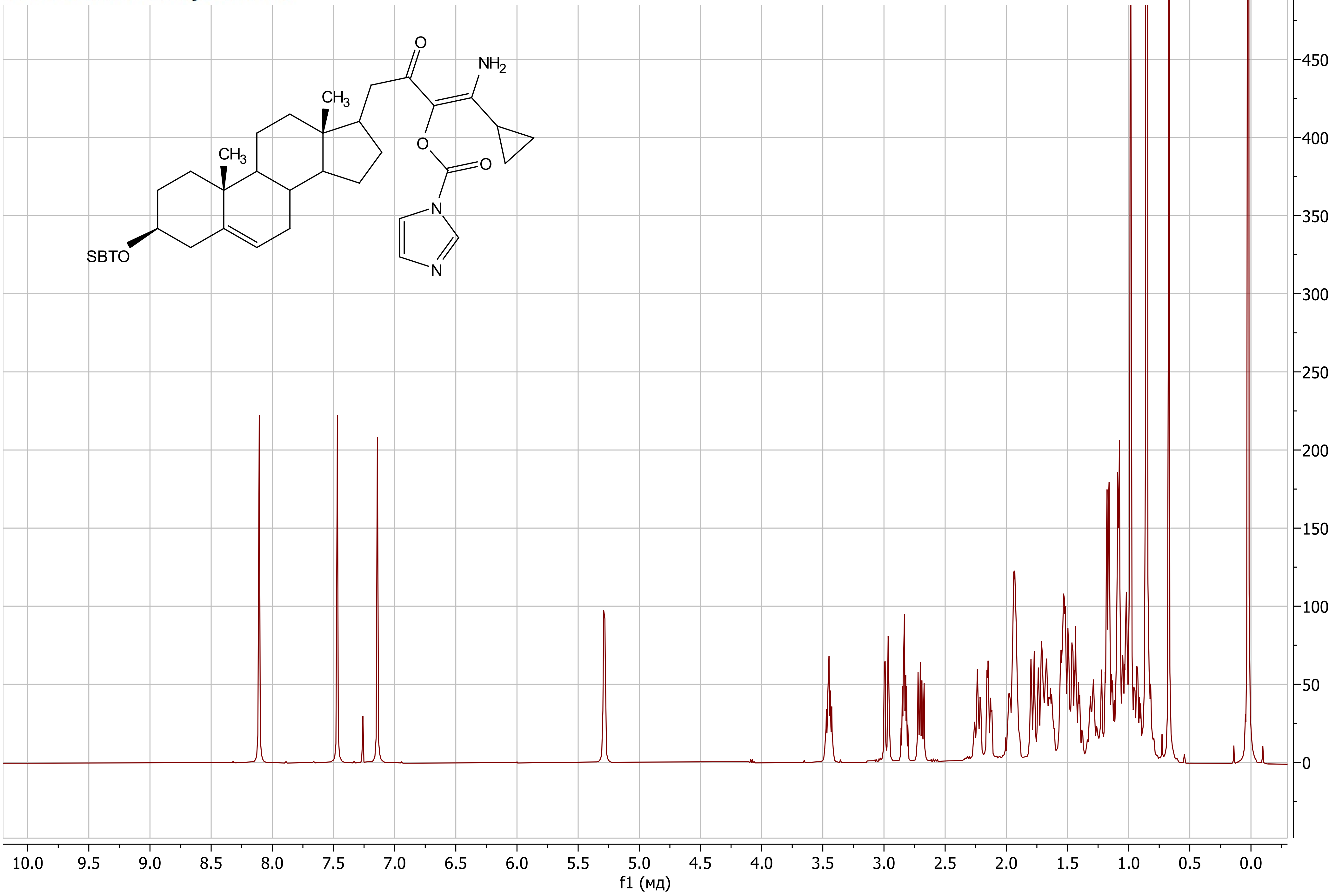

(*E*)-1-Amino-4-((17*R*)-3β-((*tert*-butyldimethylsilyl)oxy)-androst-5-en-17-yl)-1-cyclopropyl-3-oxobut-1-en-2-yl 1*H*-imidazole-1-carboxylate (**26c**)

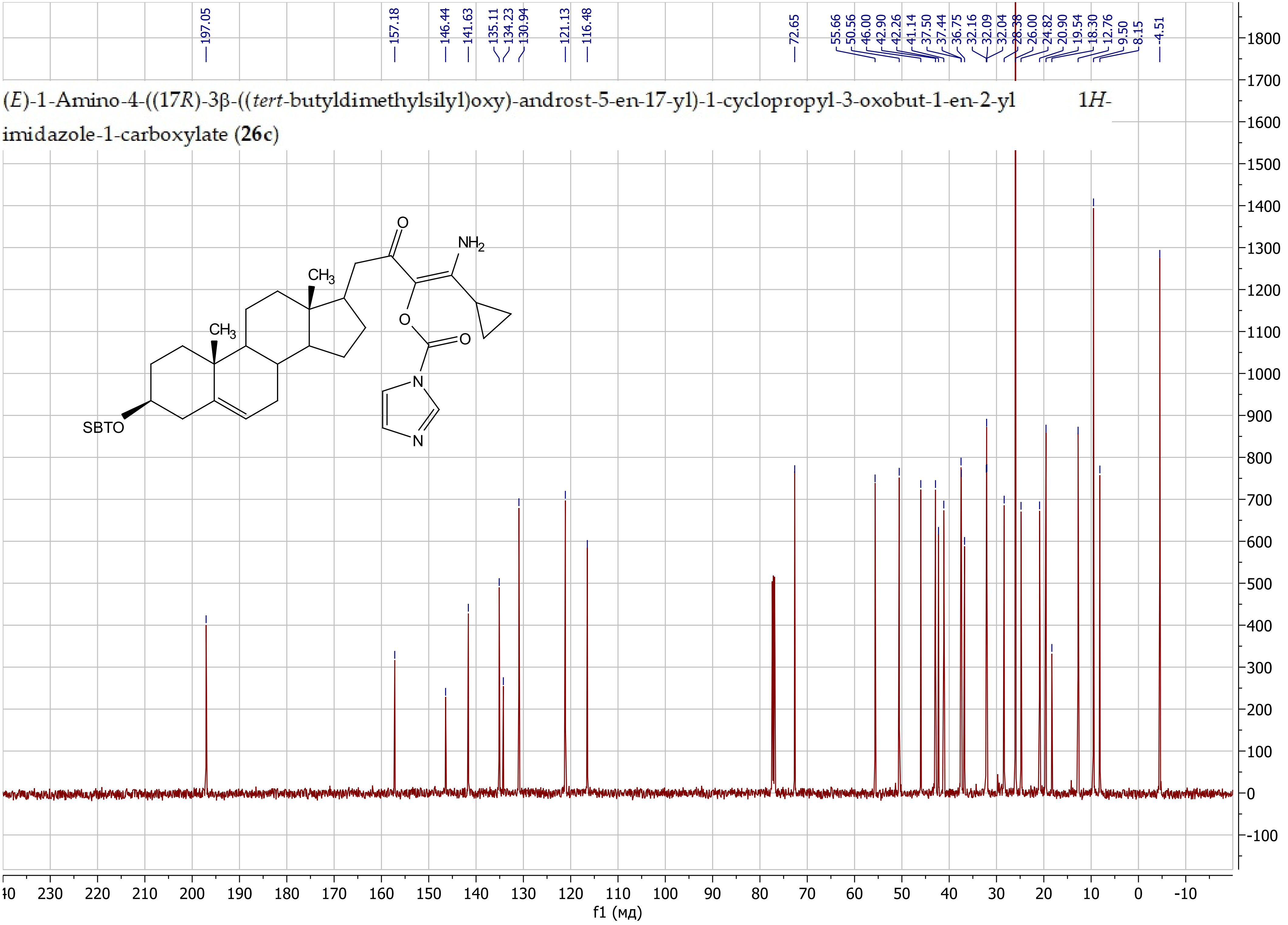

(*E*)-1-Amino-4-((17*R*)-3β-hydroxy-androst-5-en-17-yl)-1-cyclopropyl-3-oxobut-1-en-2-yl 1H-imidazole-1-carboxylate (**27**)

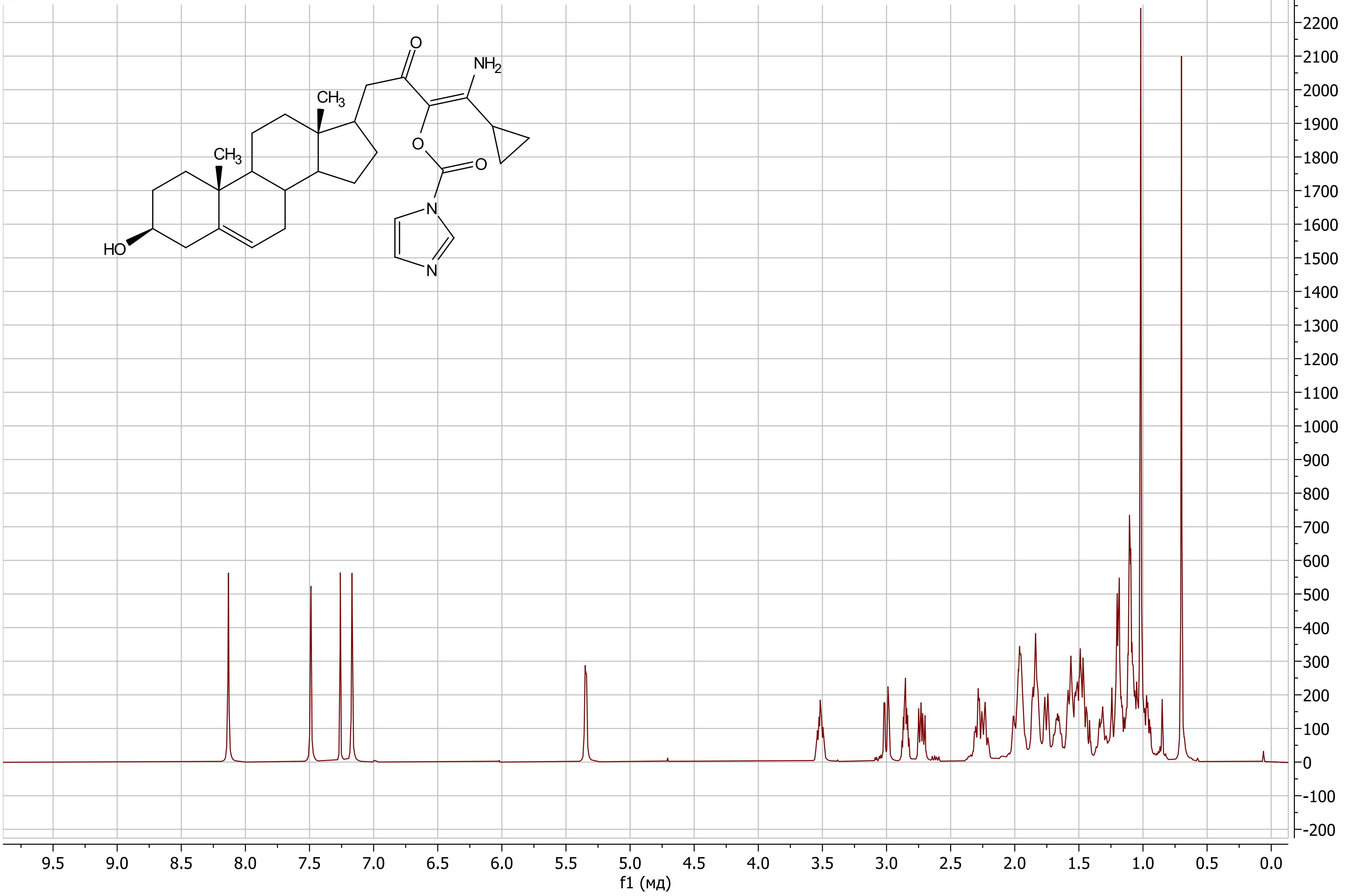

(*E*)-1-Amino-4-((17*R*)-3β-hydroxy-androst-5-en-17-yl)-1-cyclopropyl-3-oxobut-1-en-2-yl 1H-imidazole-1-carboxylate (**27**)

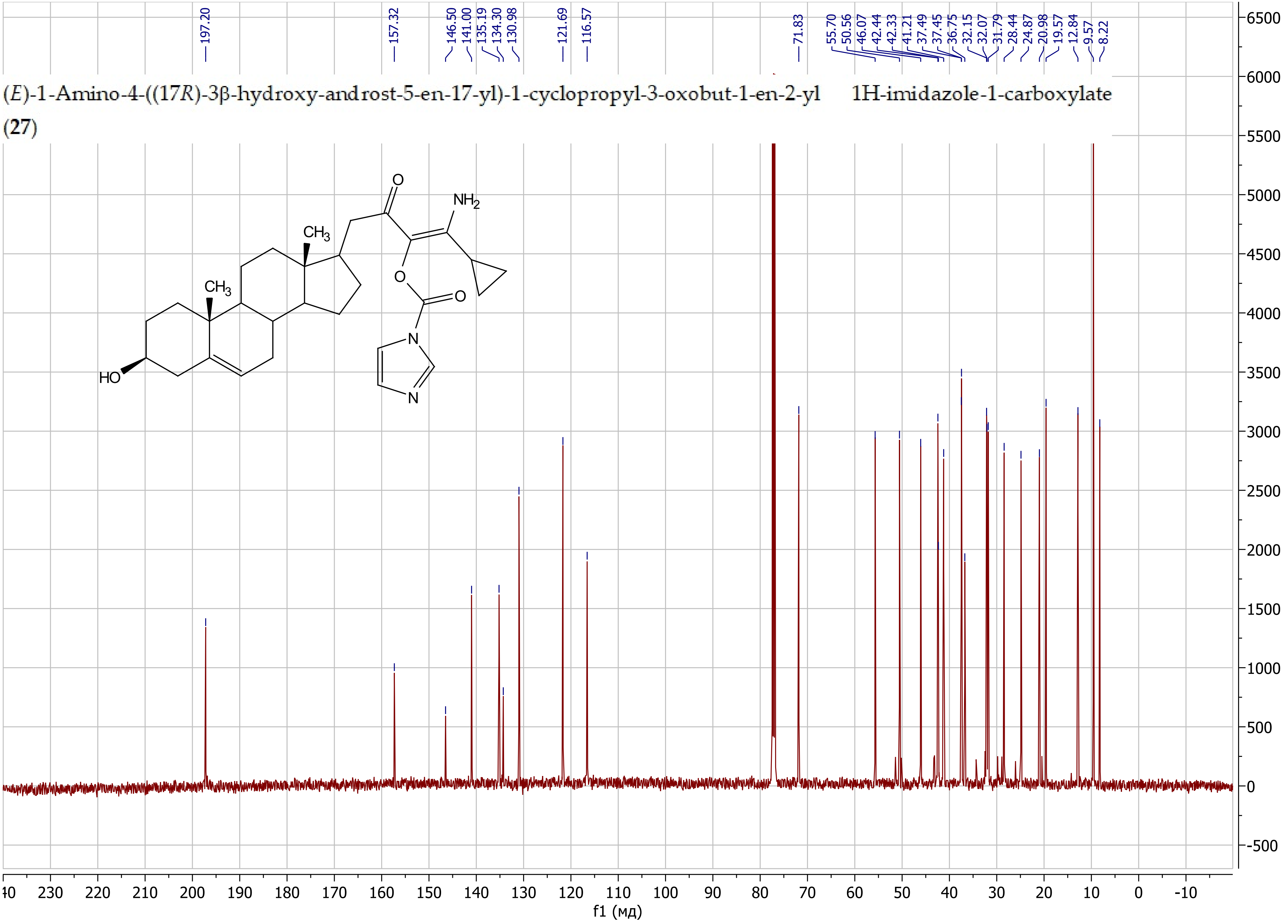

5-(2-((17*R*)-3β-((*tert*-Butyldimethylsilyl)oxy)-androst-5-en-17-yl)acetyl)-4-cyclopropyloxazol-2(3*H*)-one (**28**)

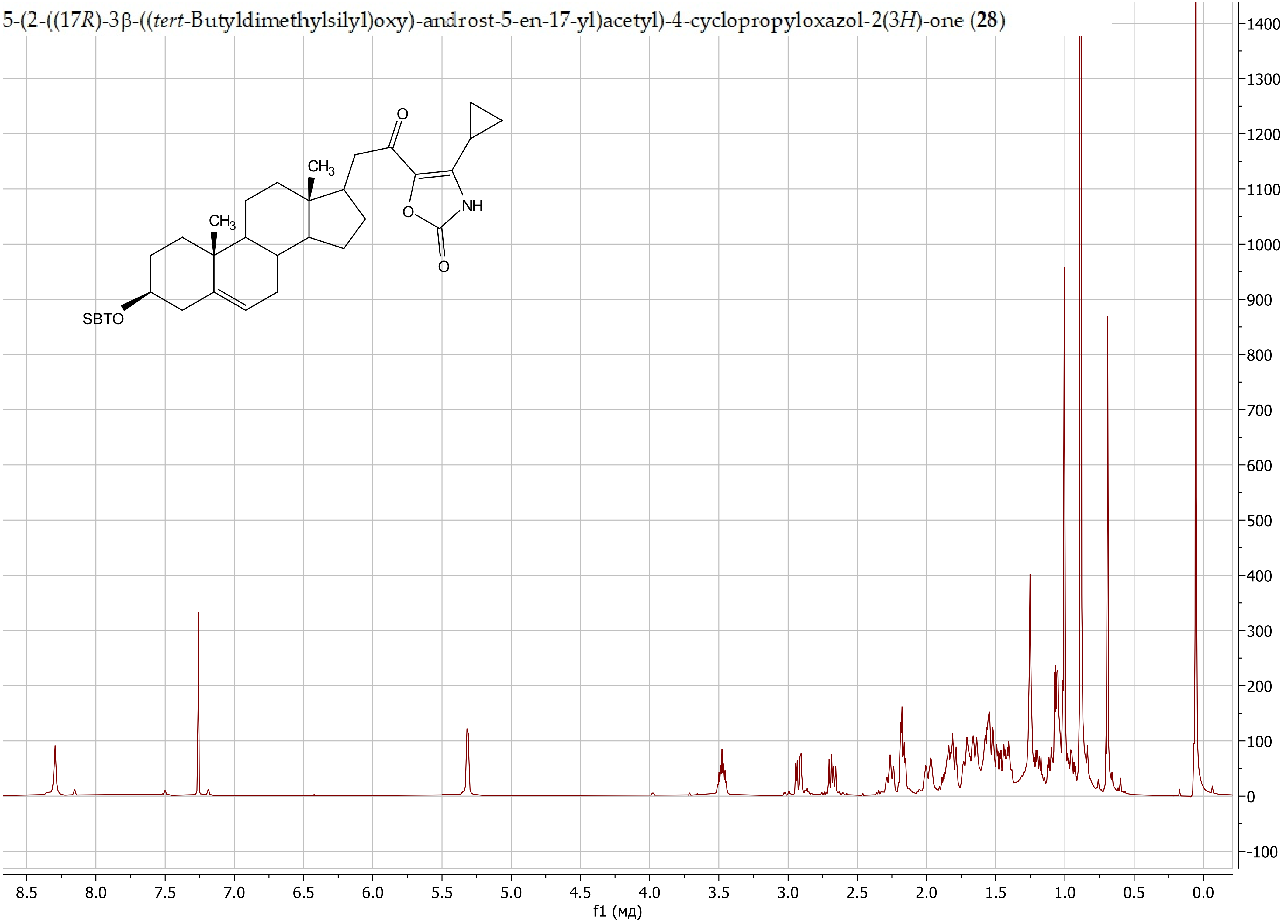

5-(2-((17*R*)-3β-((*tert*-Butyldimethylsilyl)oxy)-androst-5-en-17-yl)acetyl)-4-cyclopropyloxazol-2(3*H*)-one (**28**)

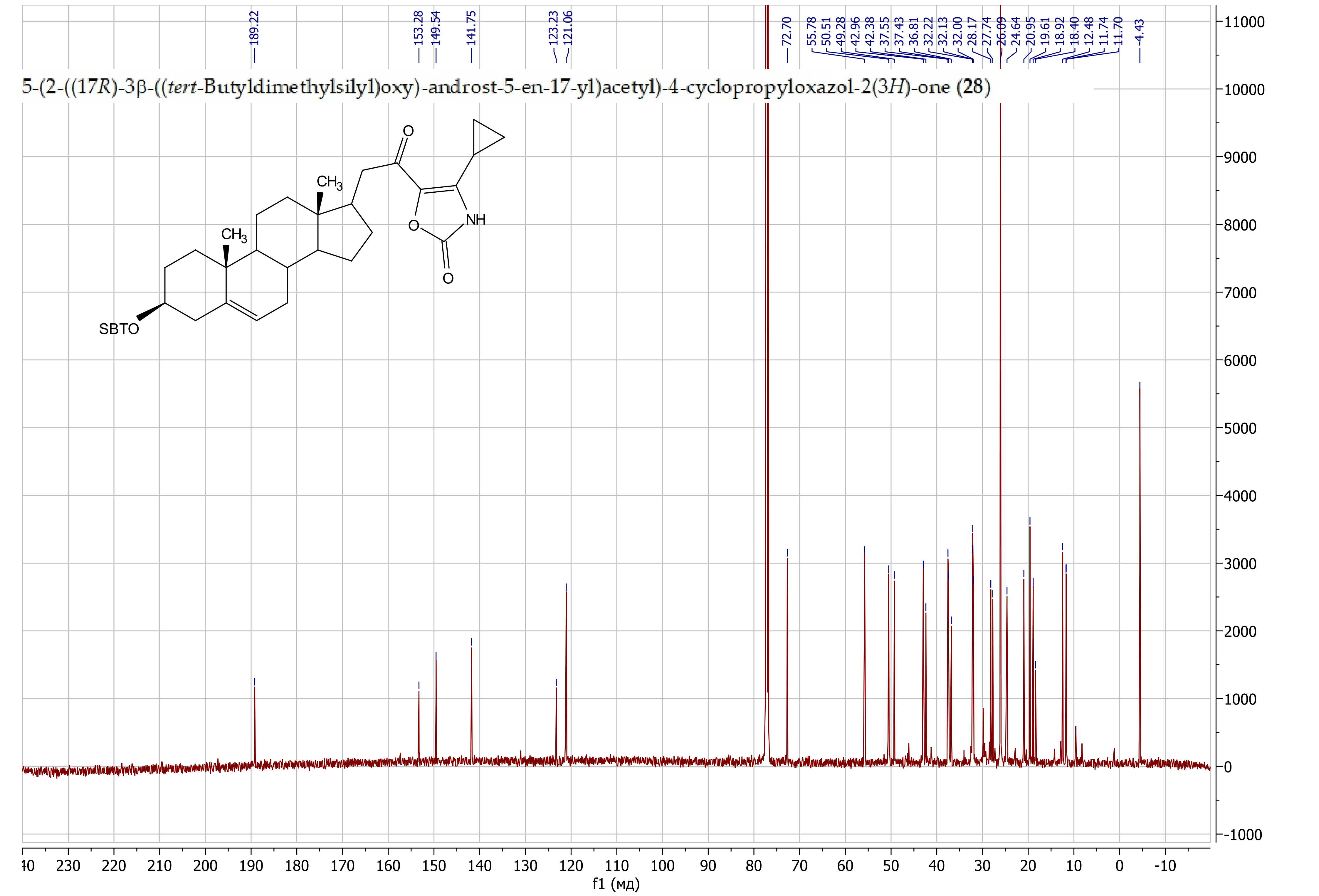

4-(3β-((*tert*-Butyldimethylsilyl)oxy)-androst-5-en-17-yl)-3-oxobutanenitrile (**31**)

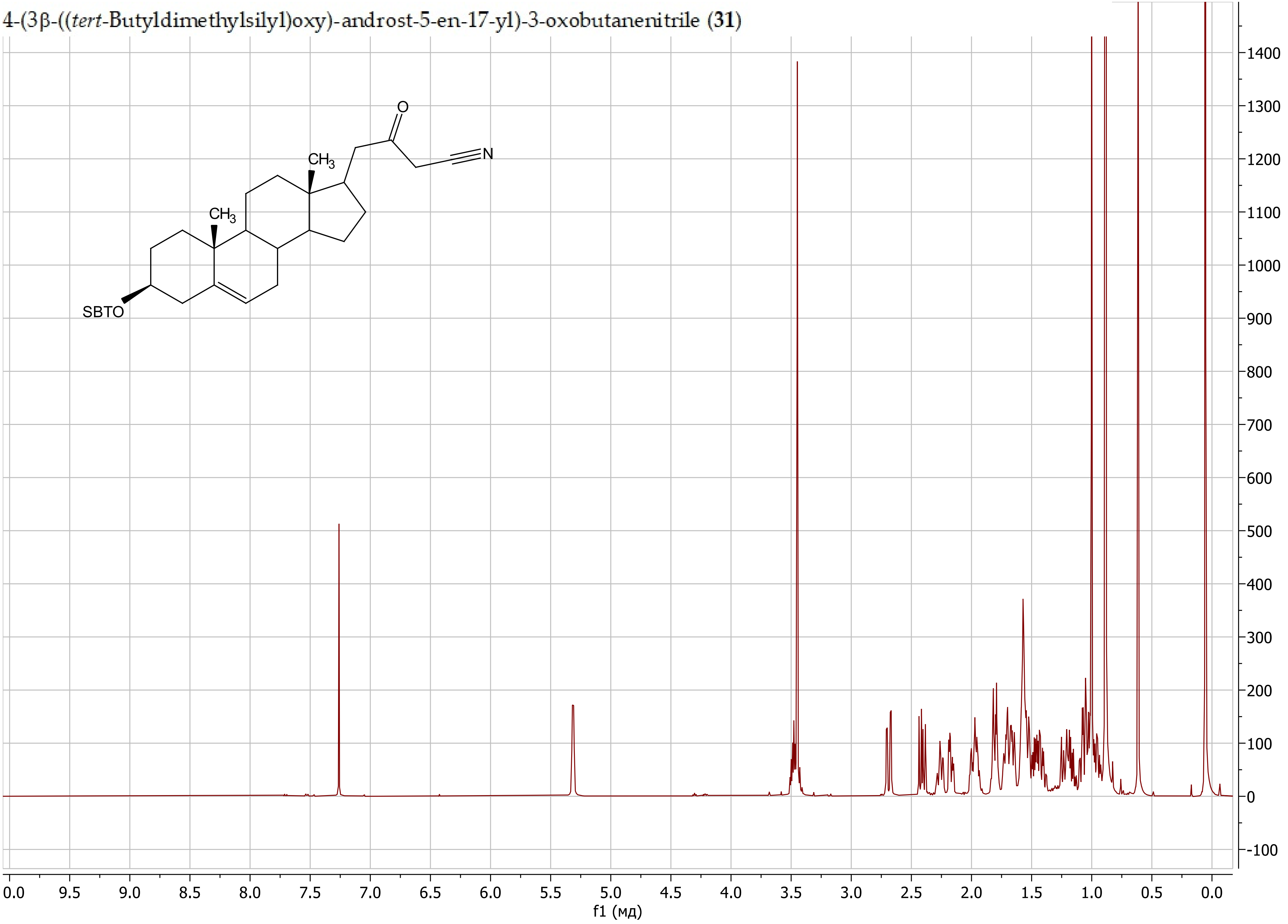

4-(3β-((*tert*-Butyldimethylsilyl)oxy)-androst-5-en-17-yl)-3-oxobutanenitrile (**31**)

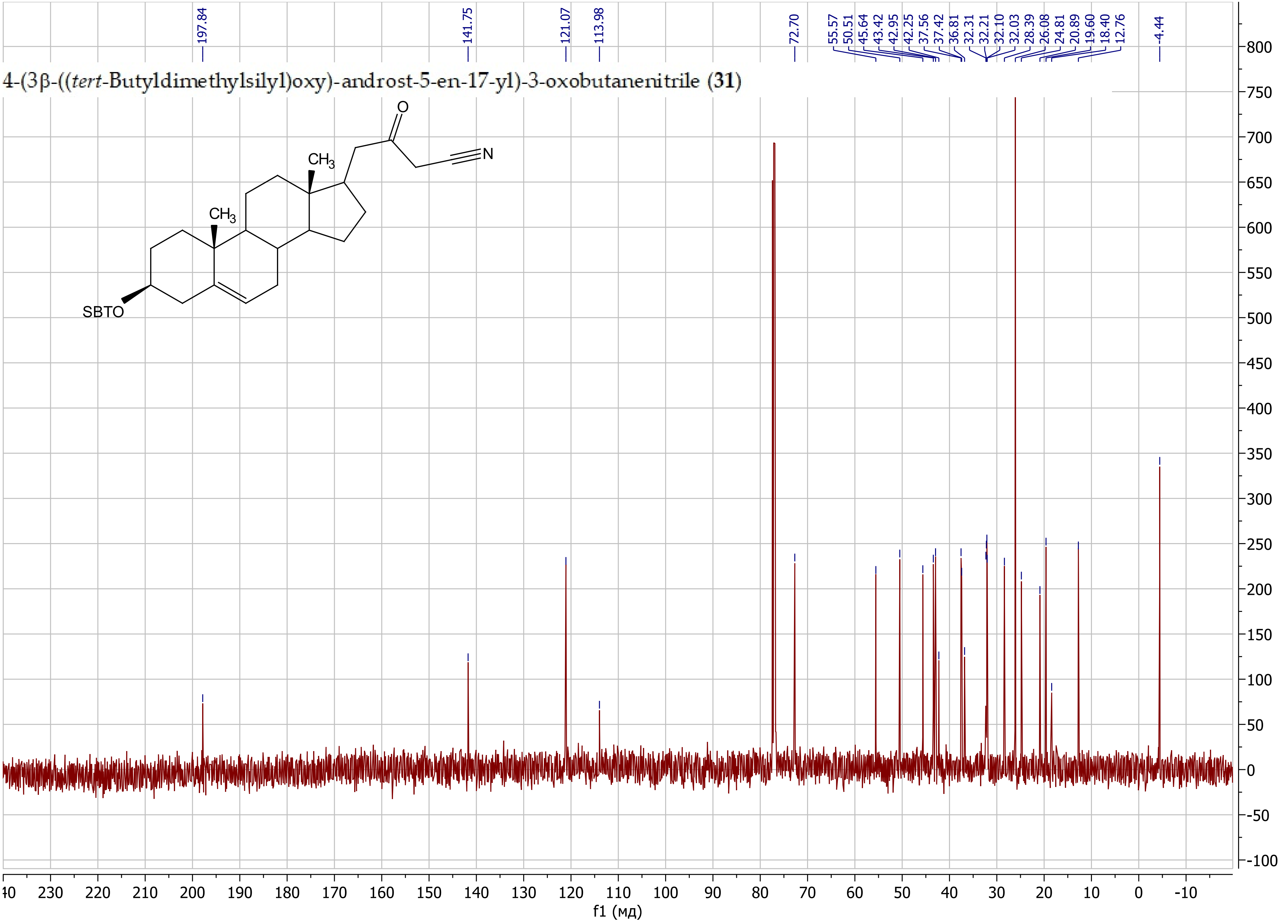

(17*R*)-17-((3-(Hydroxymethyl)isoxazol-5-yl)methyl)-androst-5-en-3β-ol (**32**)

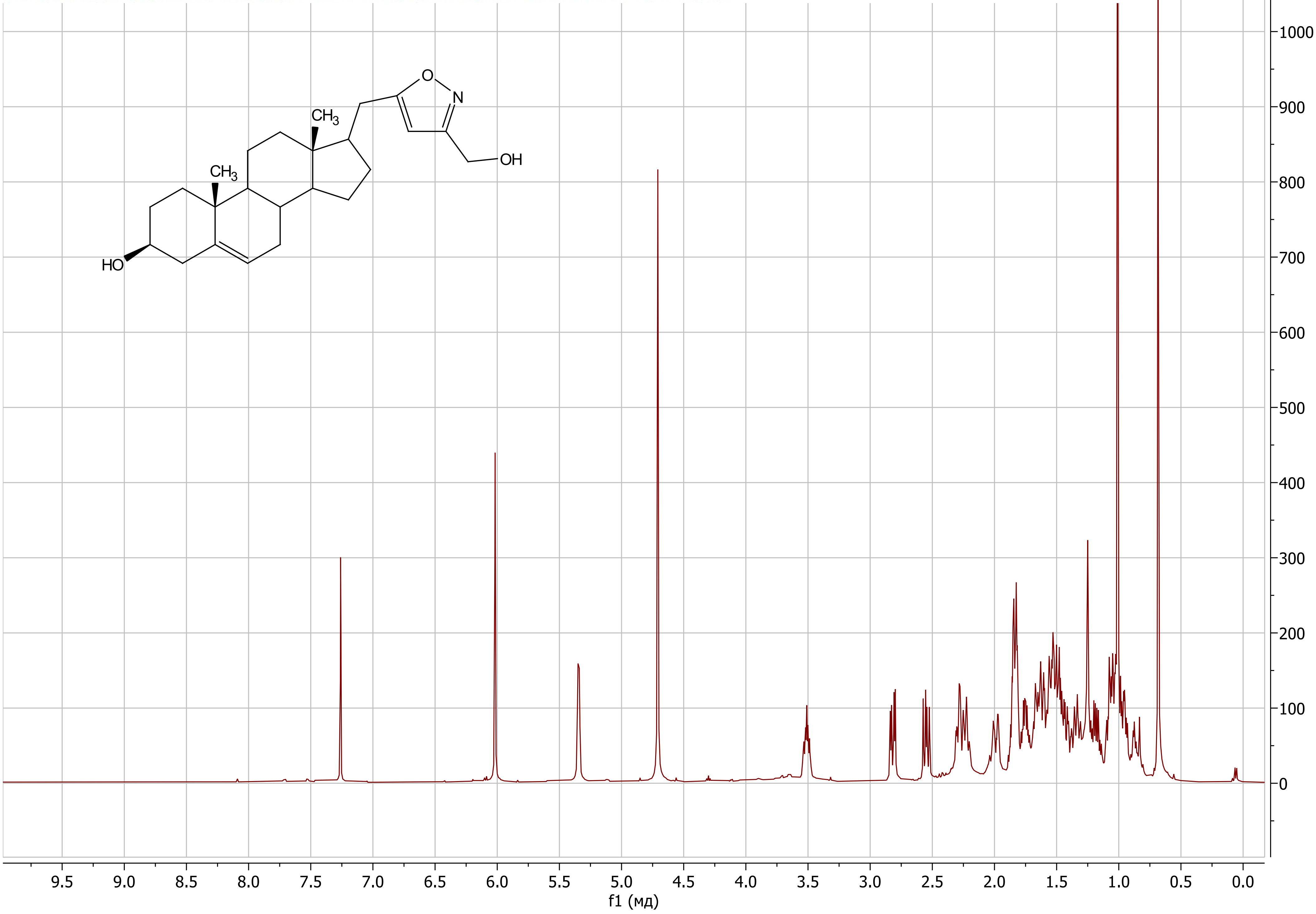

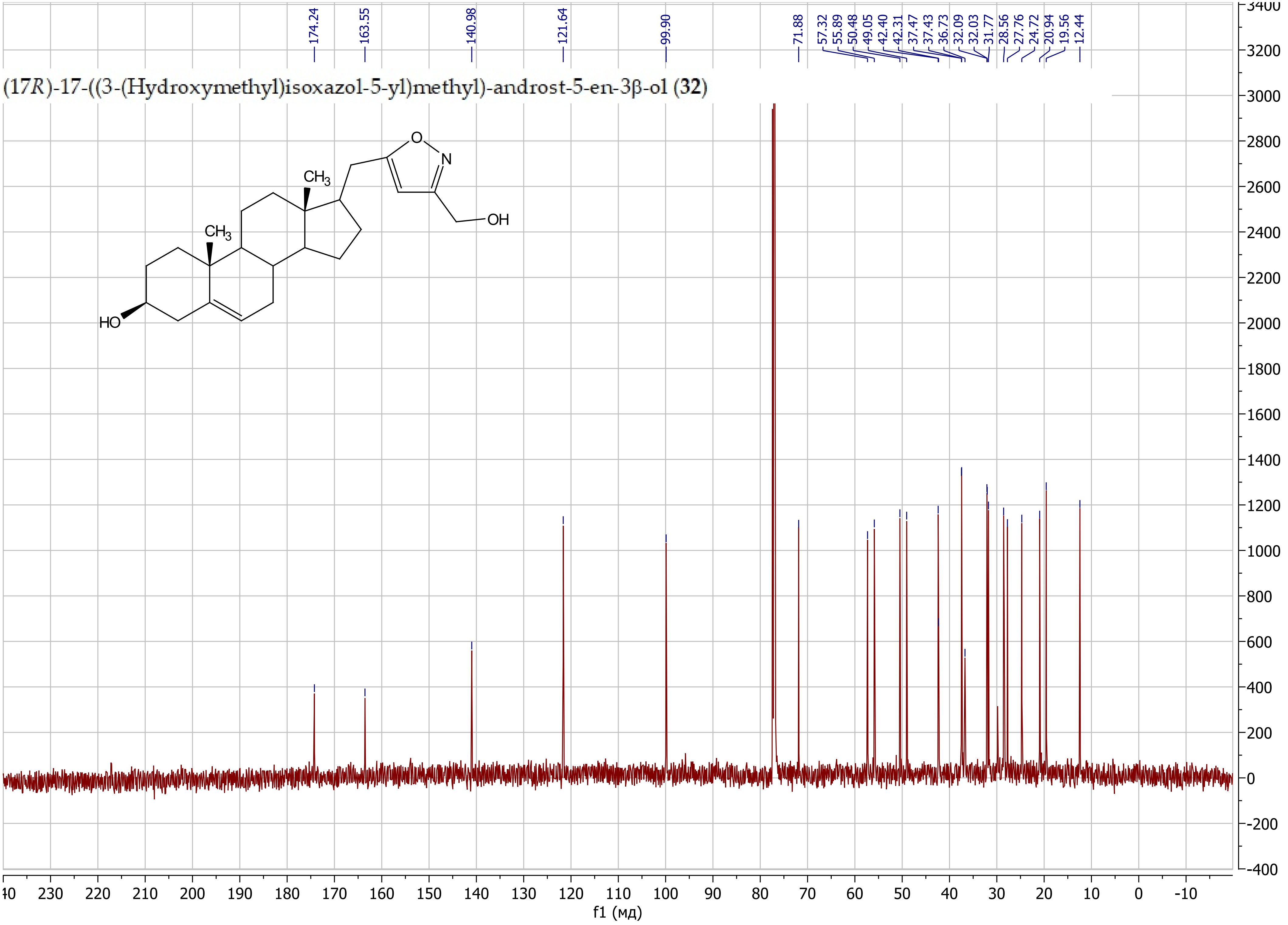
(17*R*)-17-((3-(Hydroxymethyl)isoxazol-5-yl)methyl)-androst-5-en-3β-ol (**32**)
174.24
163.55
140.98
121.64
99.90
71.88
57.32
55.89
50.48
49.05
42.40
42.31
37.47
37.43
36.73
32.09
32.03
31.77
28.56
27.76
24.72
20.94
19.56
12.44
CH3
HO
OH
O
N
f1 (мд)

5-(((17*R*)-3β-((*tert*-Butyldimethylsilyl)oxy)-androst-5-en-17-yl)methyl)isoxazol-3-yl)methanol (**33**)

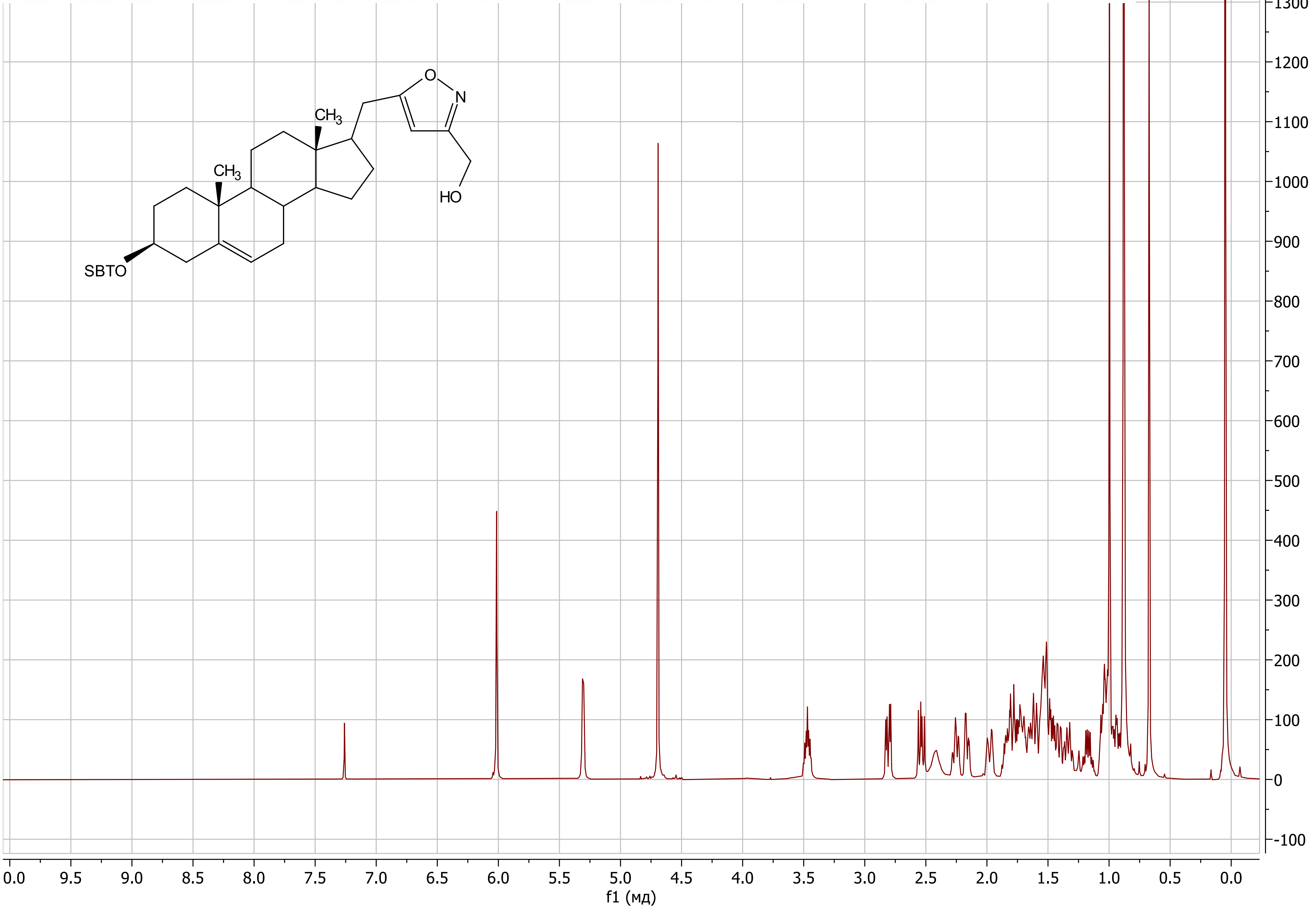

5-(((17*R*)-3β-((*tert*-Butyldimethylsilyl)oxy)-androst-5-en-17-yl)methyl)isoxazol-3-yl)methanol (**33**)

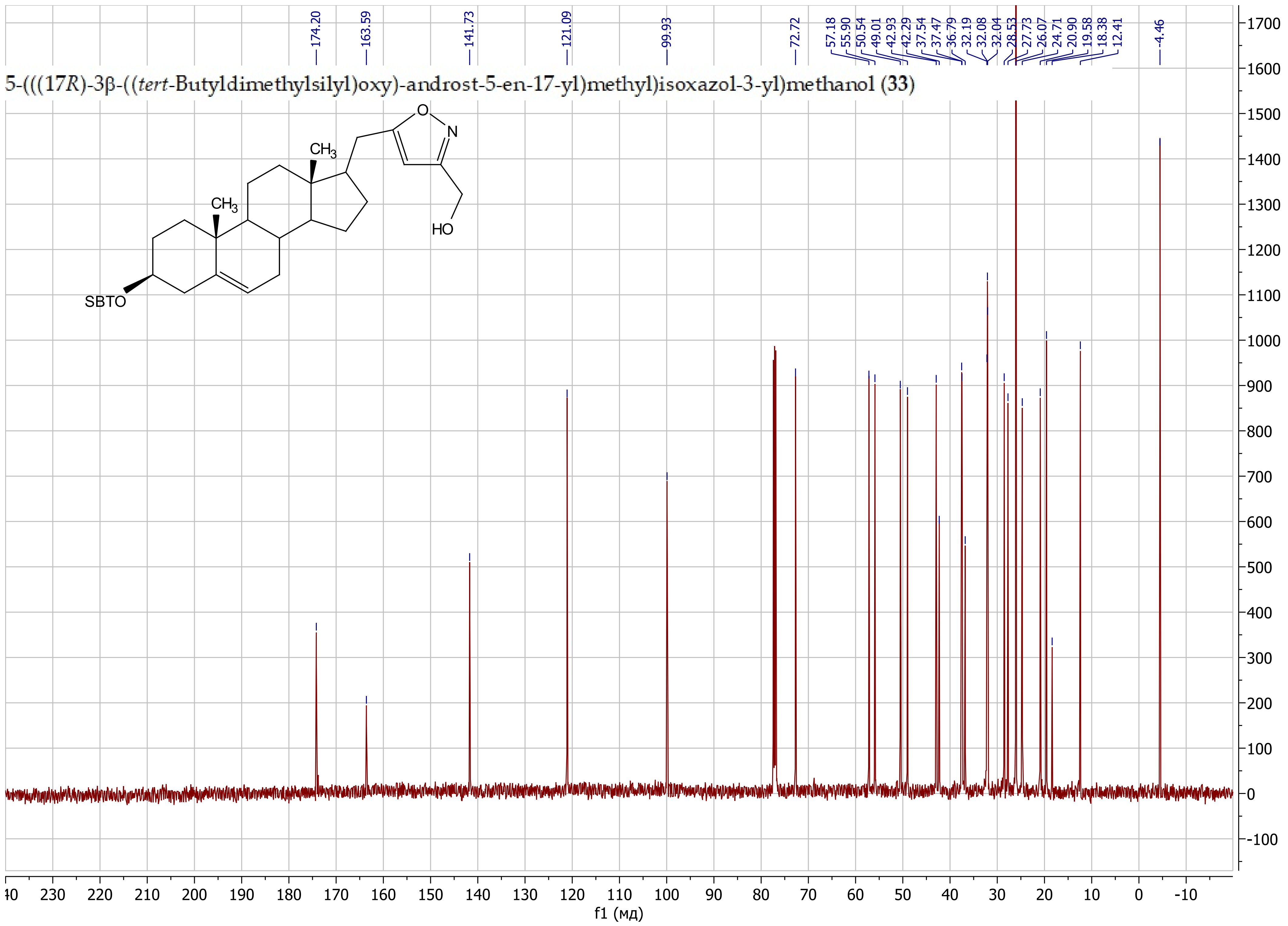

5-((3β-((*tert*-Butyldimethylsilyl)oxy)-androst-5-en-17-yl)methyl)isoxazol-3-yl)methyl methanesulfonate (**34**)

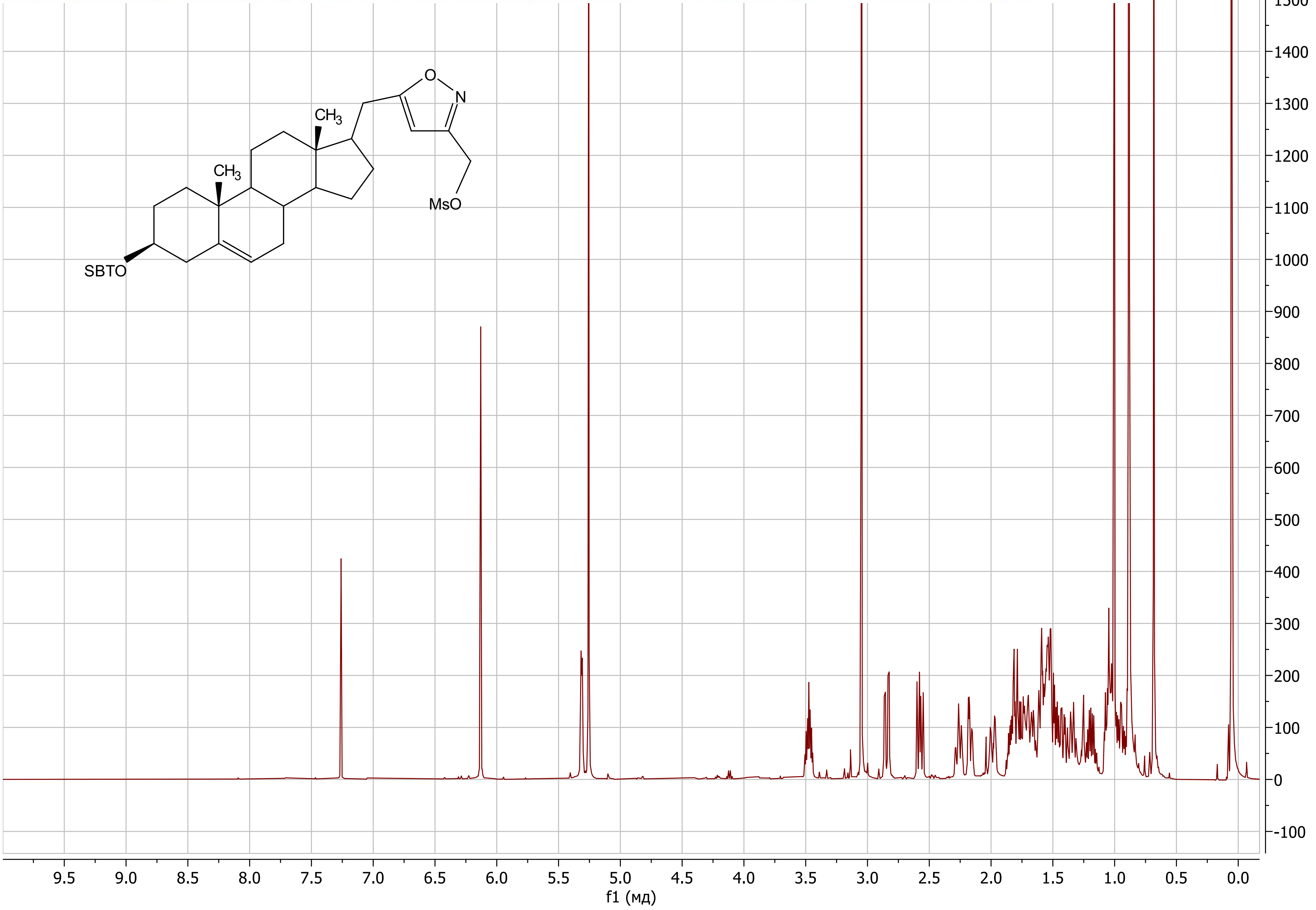

5-((3β-((*tert*-Butyldimethylsilyl)oxy)-androst-5-en-17-yl)methyl)isoxazol-3-yl)methyl methanesulfonate (**34**)

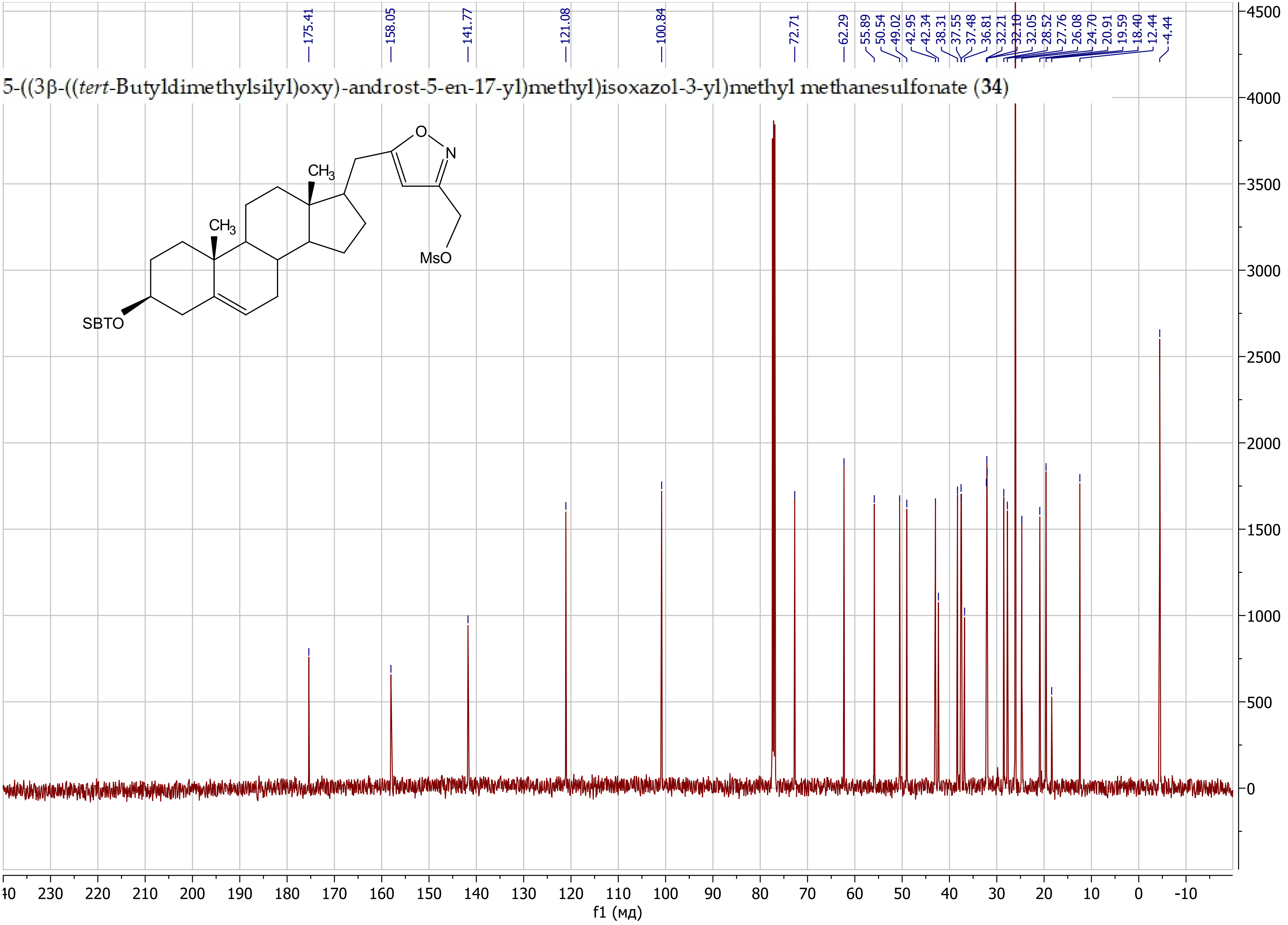

3-(Azidomethyl)-5-(((17*R*)-3β-((*tert*-butyldimethylsilyl)oxy)-androst-5-en-17-yl)methyl)isoxazole (**35**)

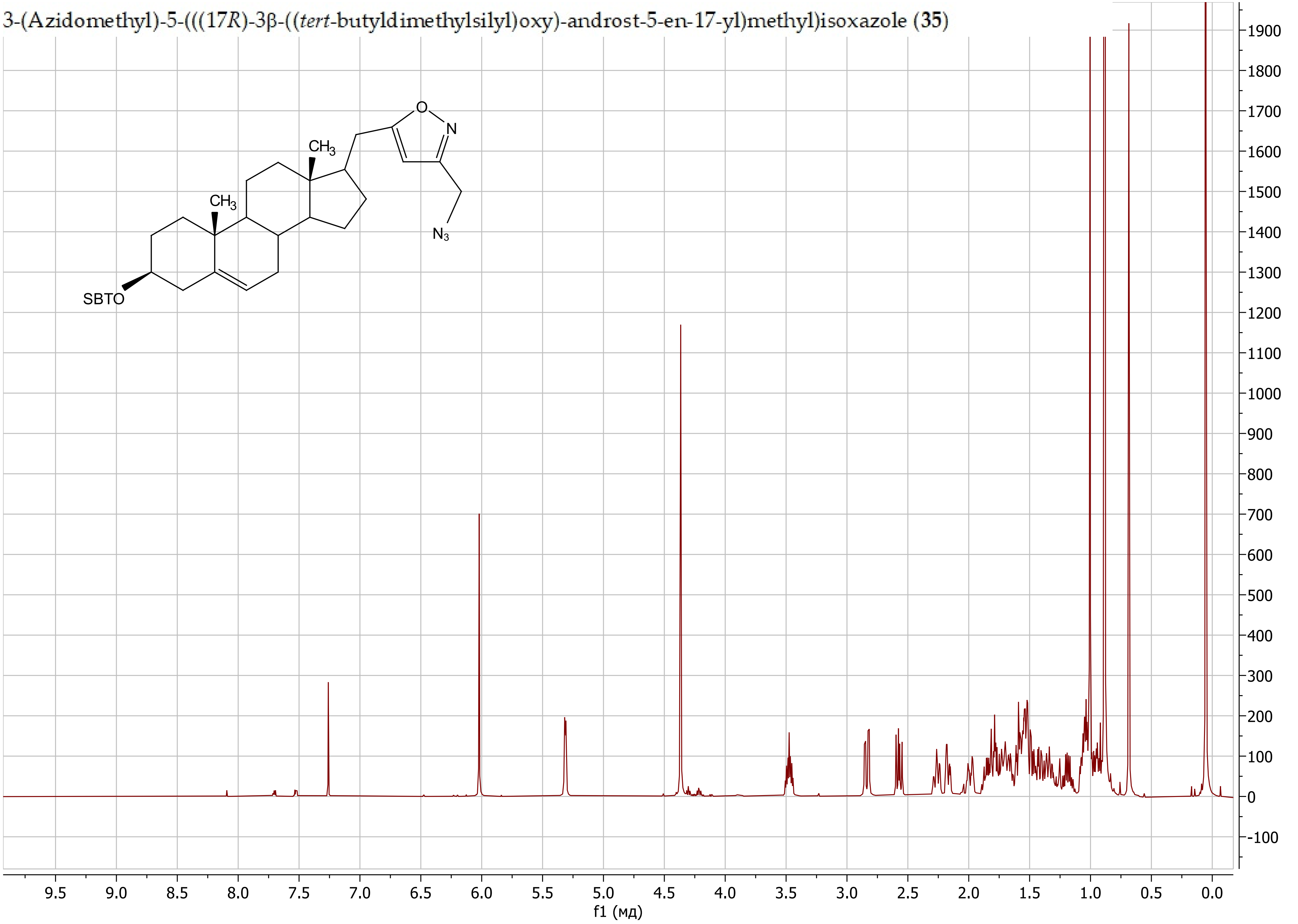

3-(Azidomethyl)-5-(((17*R*)-3β-((*tert*-butyldimethylsilyl)oxy)-androst-5-en-17-yl)methyl)isoxazole (**35**)

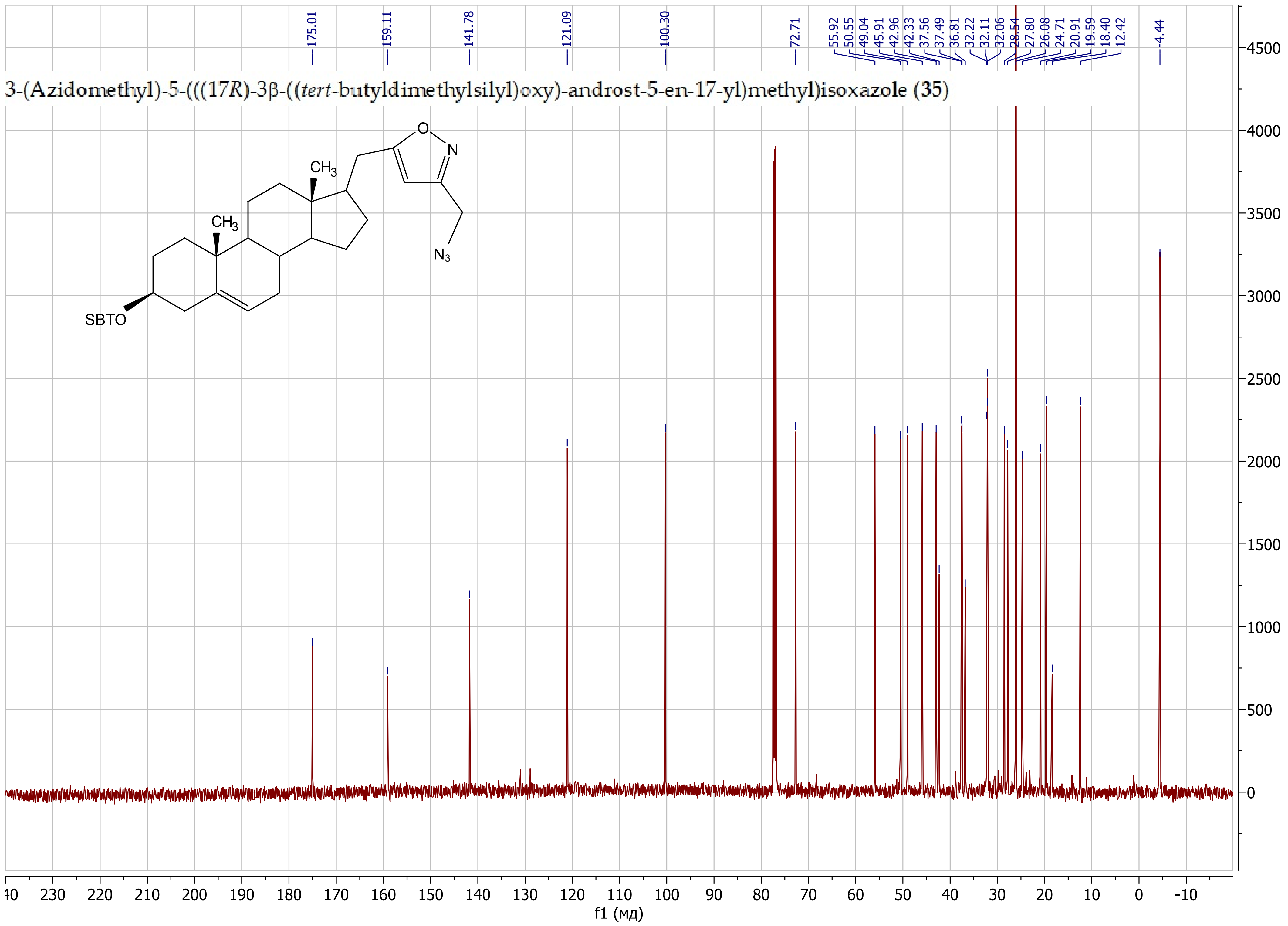

(17*R*)-17-((3-(Azidomethyl)isoxazol-5-yl)methyl)-androst-5-en-3β-ol (**36**)

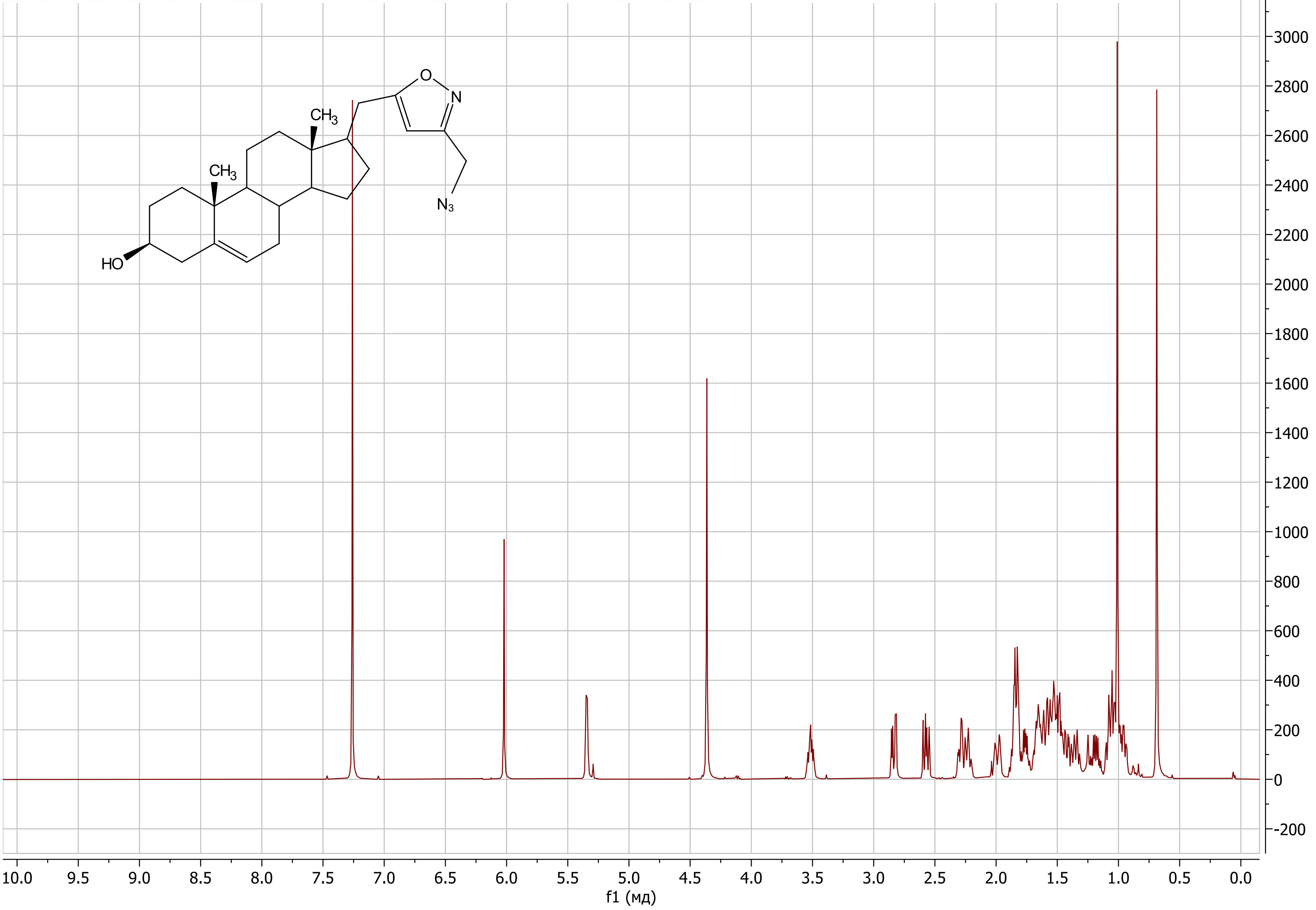

(17*R*)-17-((3-(Azidomethyl)isoxazol-5-yl)methyl)-androst-5-en-3β-ol (**36**)

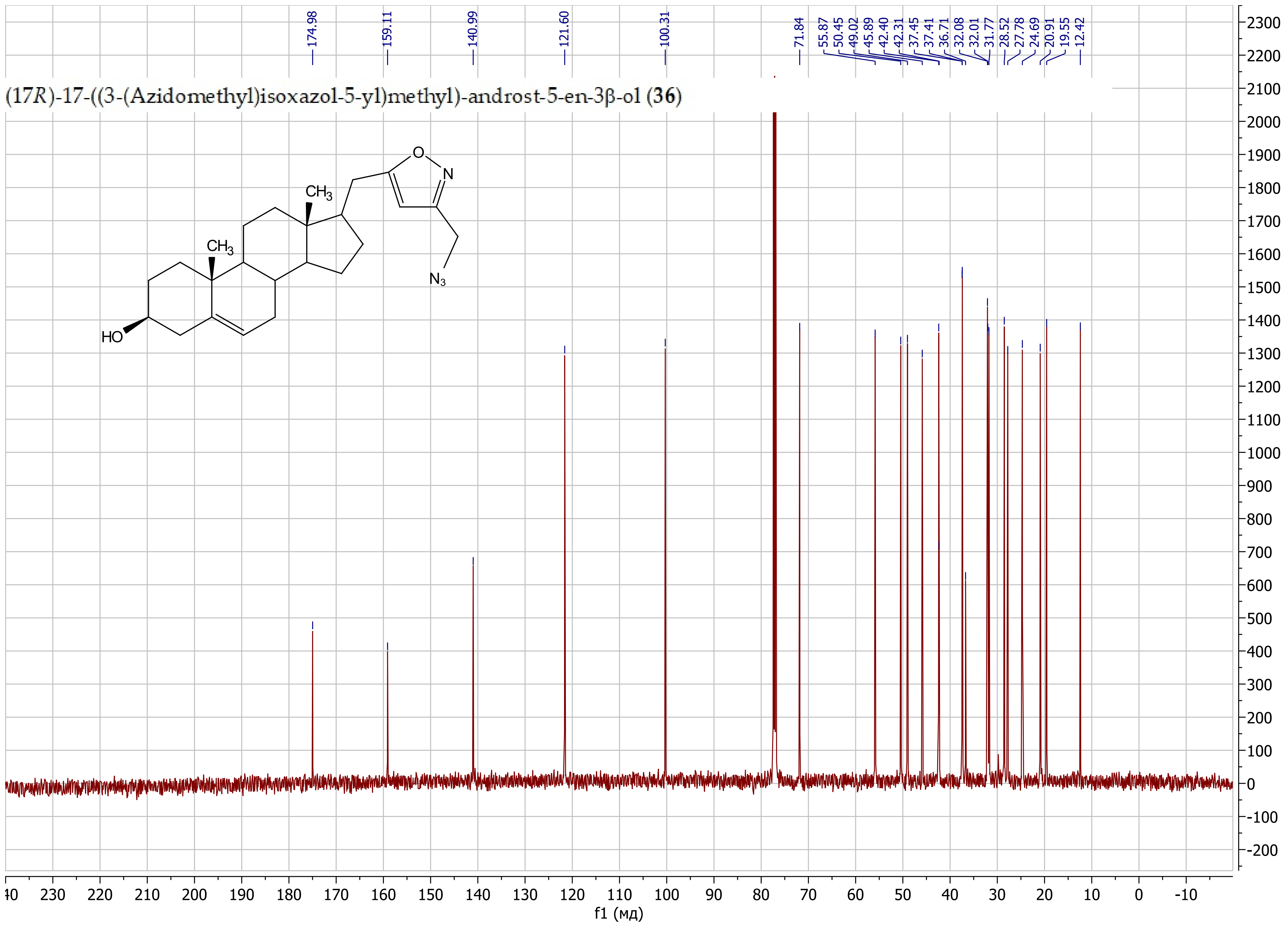

5-(((17*R*)-3β-((*tert*-Butyldimethylsilyl)oxy)-androst-5-en-17-yl)methyl)-3-(chloromethyl)isoxazole (**37**)

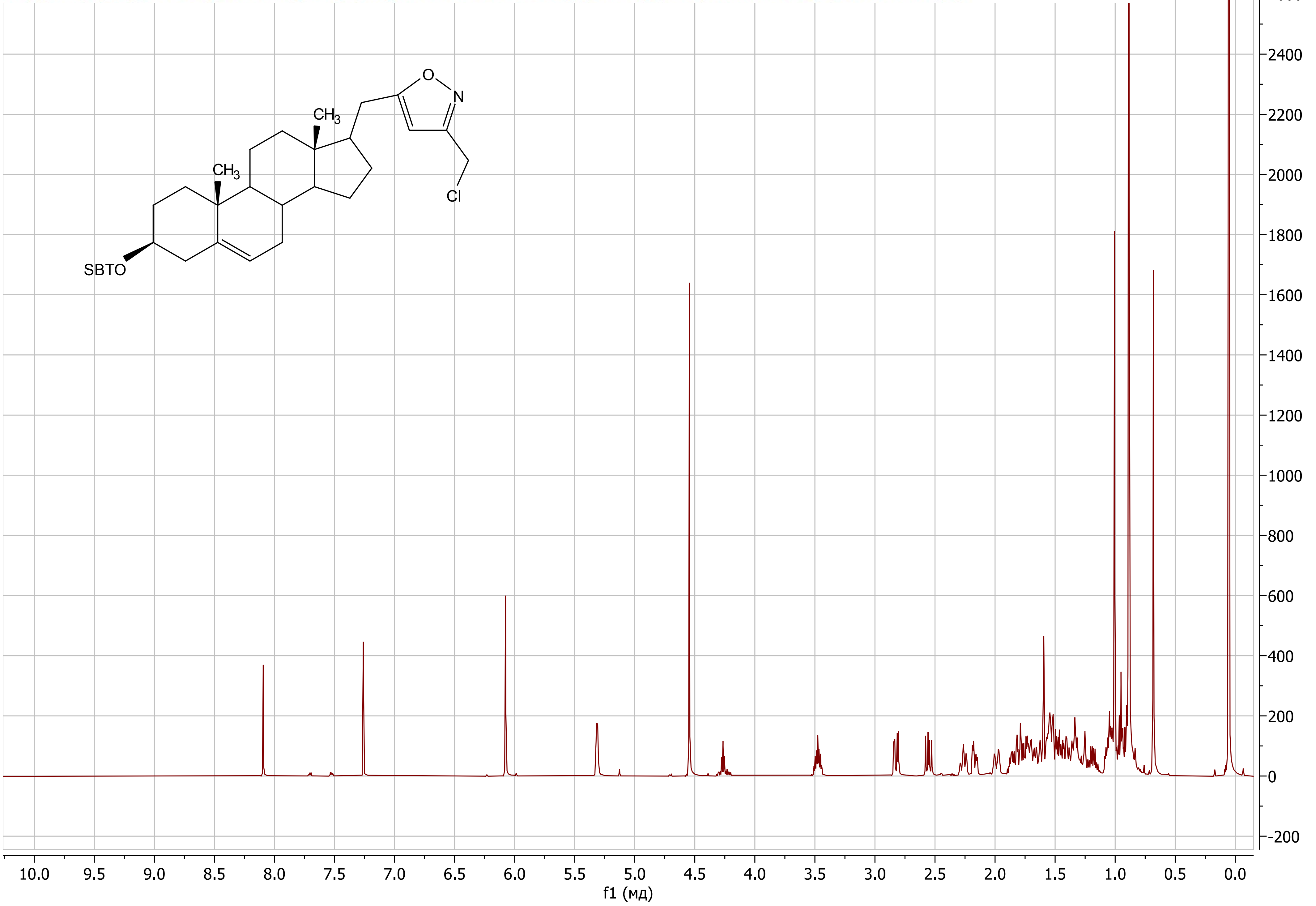

5-(((17*R*)-3β-((*tert*-Butyldimethylsilyl)oxy)-androst-5-en-17-yl)methyl)-3-(chloromethyl)isoxazole (**37**)

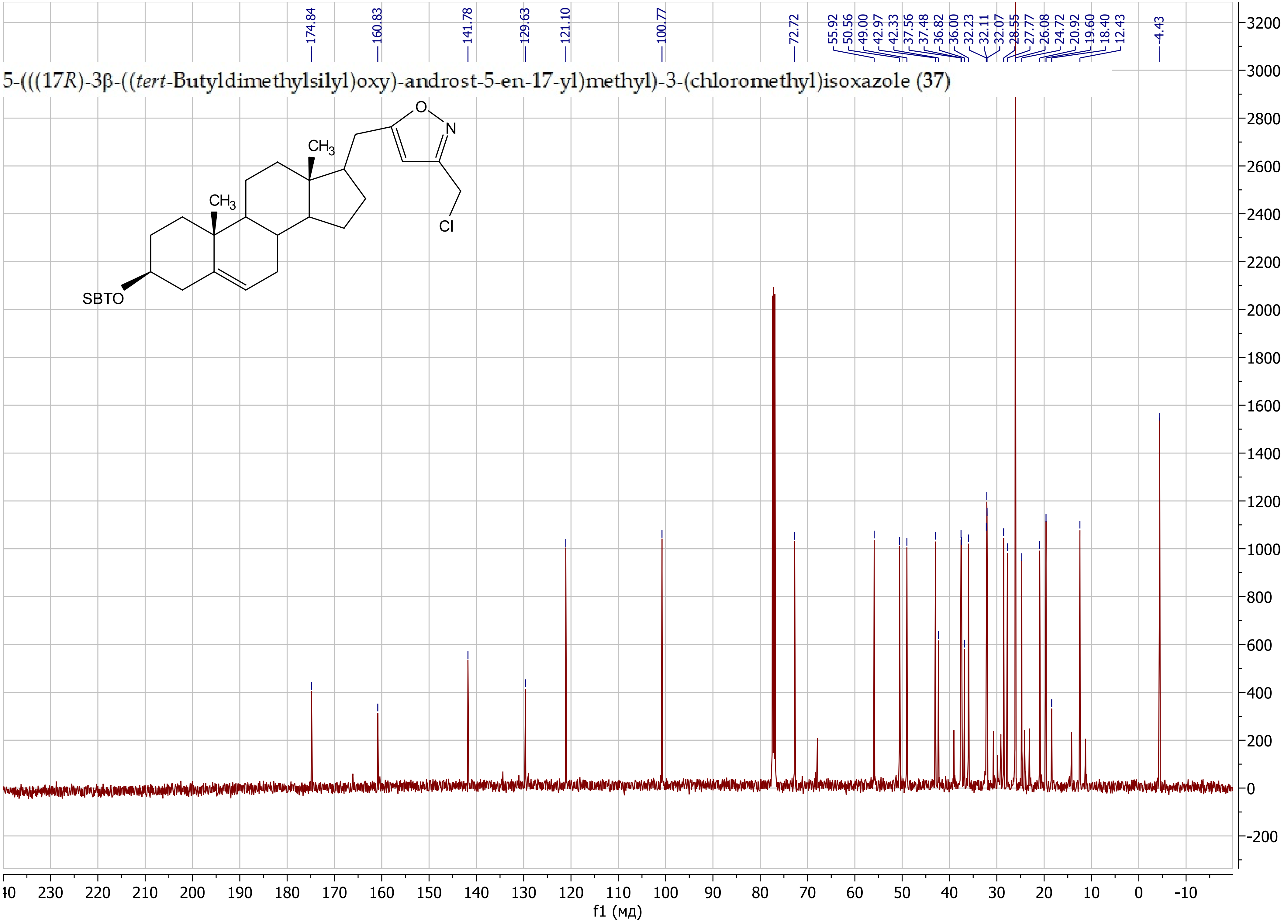

(17*R*)-17-((3-(Chloromethyl)isoxazol-5-yl)methyl)-androst-5-en-3β-ol (38)

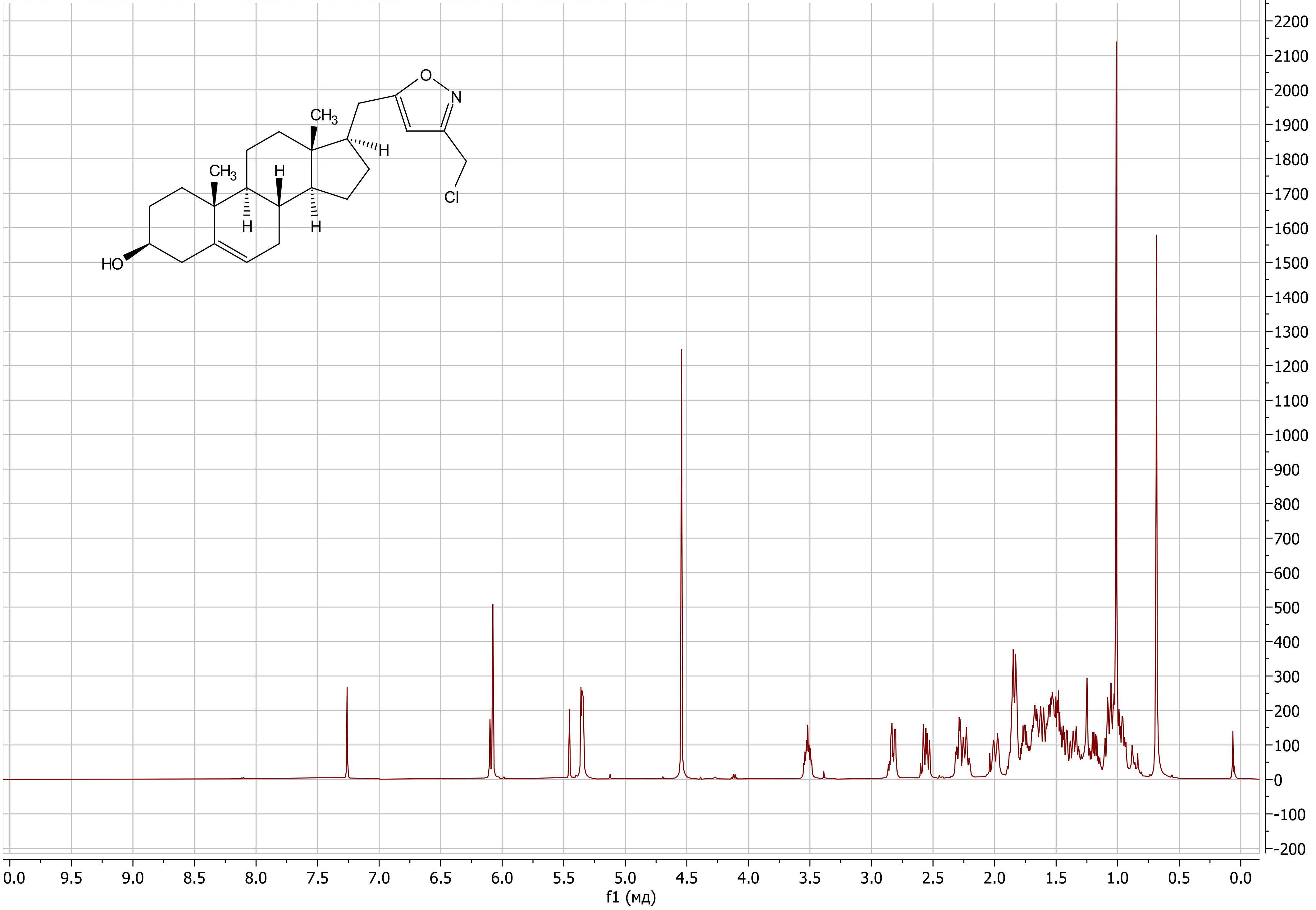

(17*R*)-17-((3-(Chloromethyl)isoxazol-5-yl)methyl)-androst-5-en-3β-ol (38)

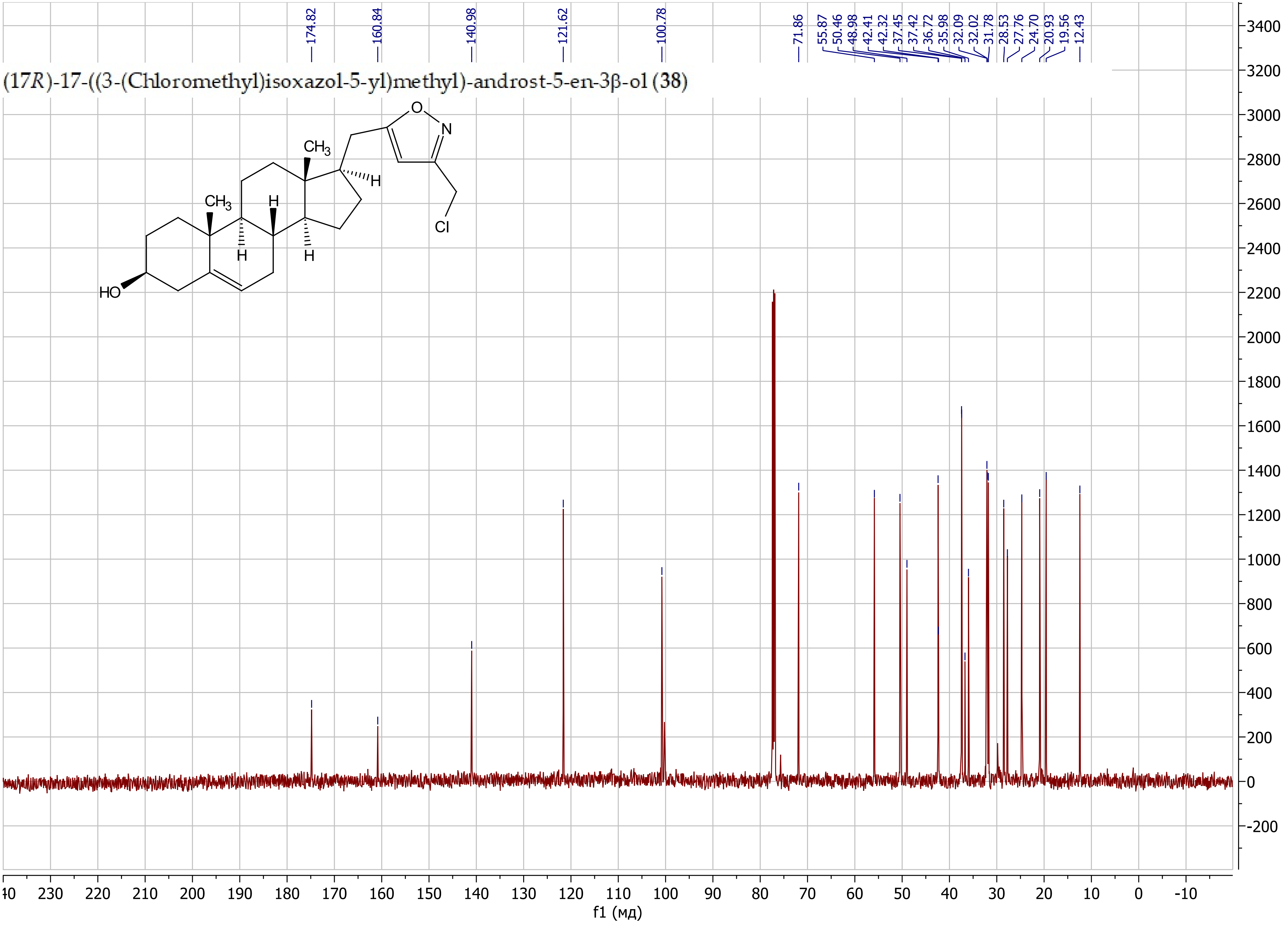

3-(((17*R*)-3β-((*tert*-Butyldimethylsilyl)oxy)-androst-5-en-17-yl)methyl)isoxazole (**40a**)

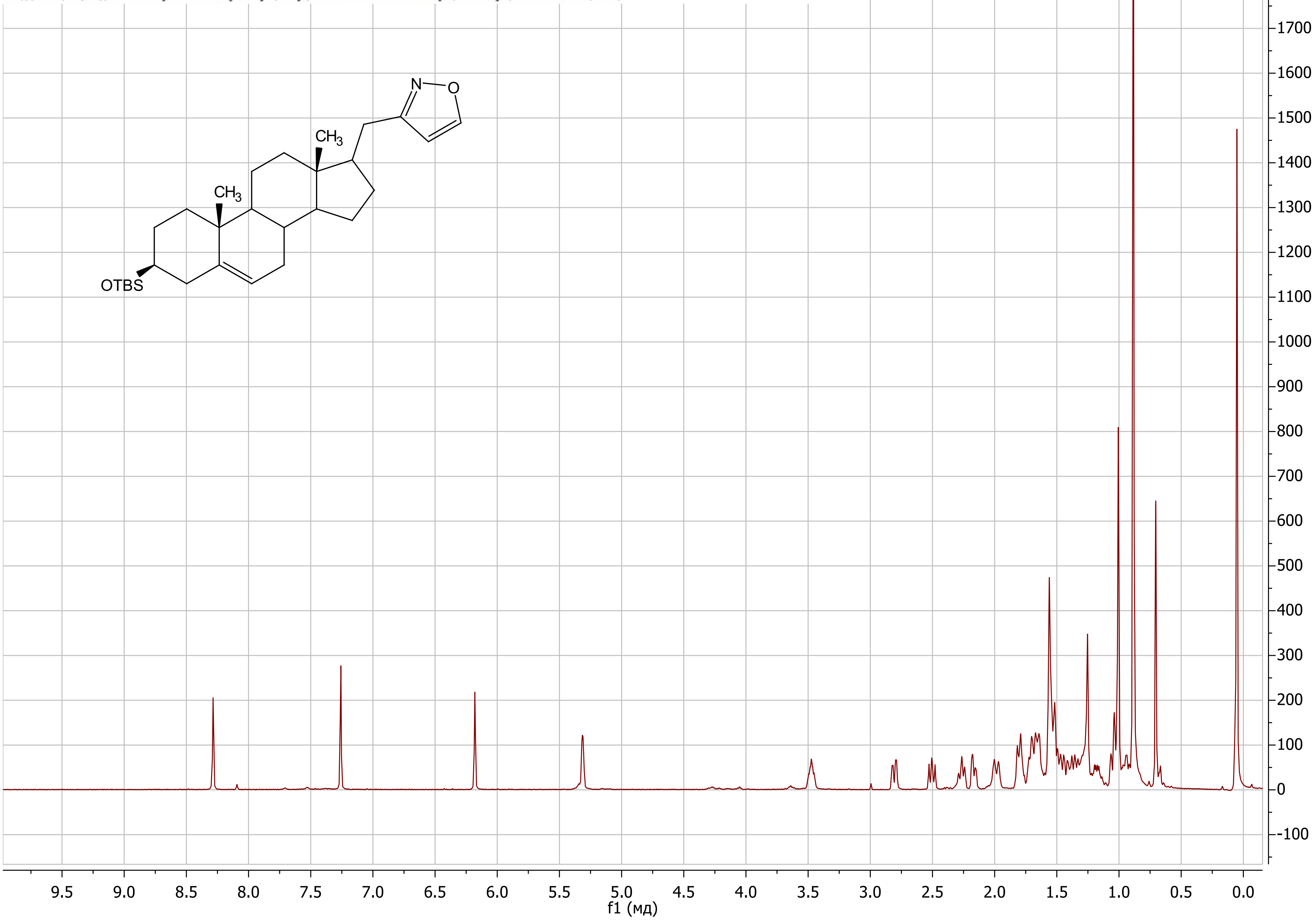

3-(((17*R*)-3β-((*tert*-Butyldimethylsilyl)oxy)-androst-5-en-17-yl)methyl)isoxazole (**40a**)

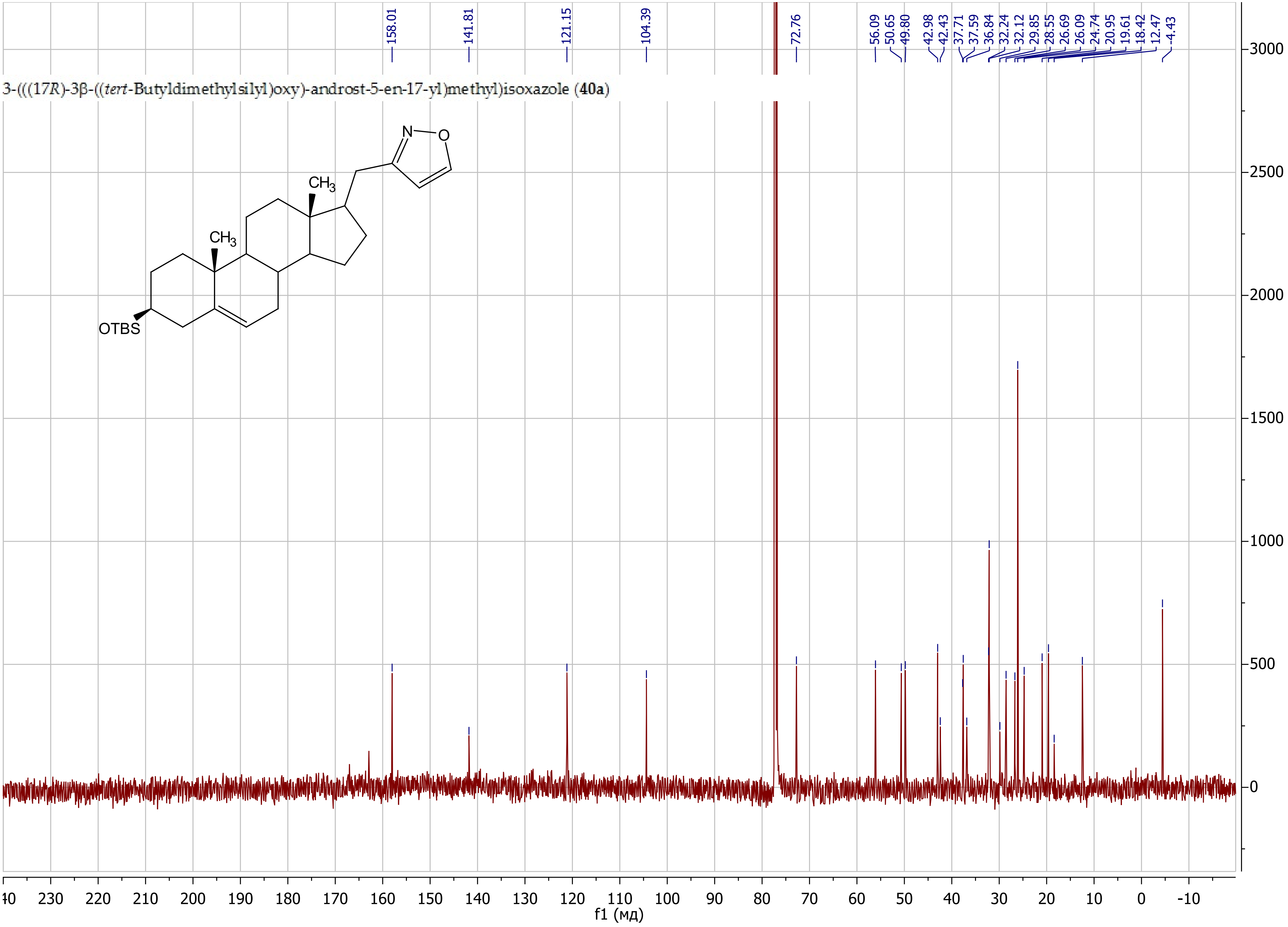

5-Butyl-3-(((17*R*)-3β-((*tert*-butyldimethylsilyl)oxy)-androst-5-en-17-yl)methyl)isoxazole (**40d**)

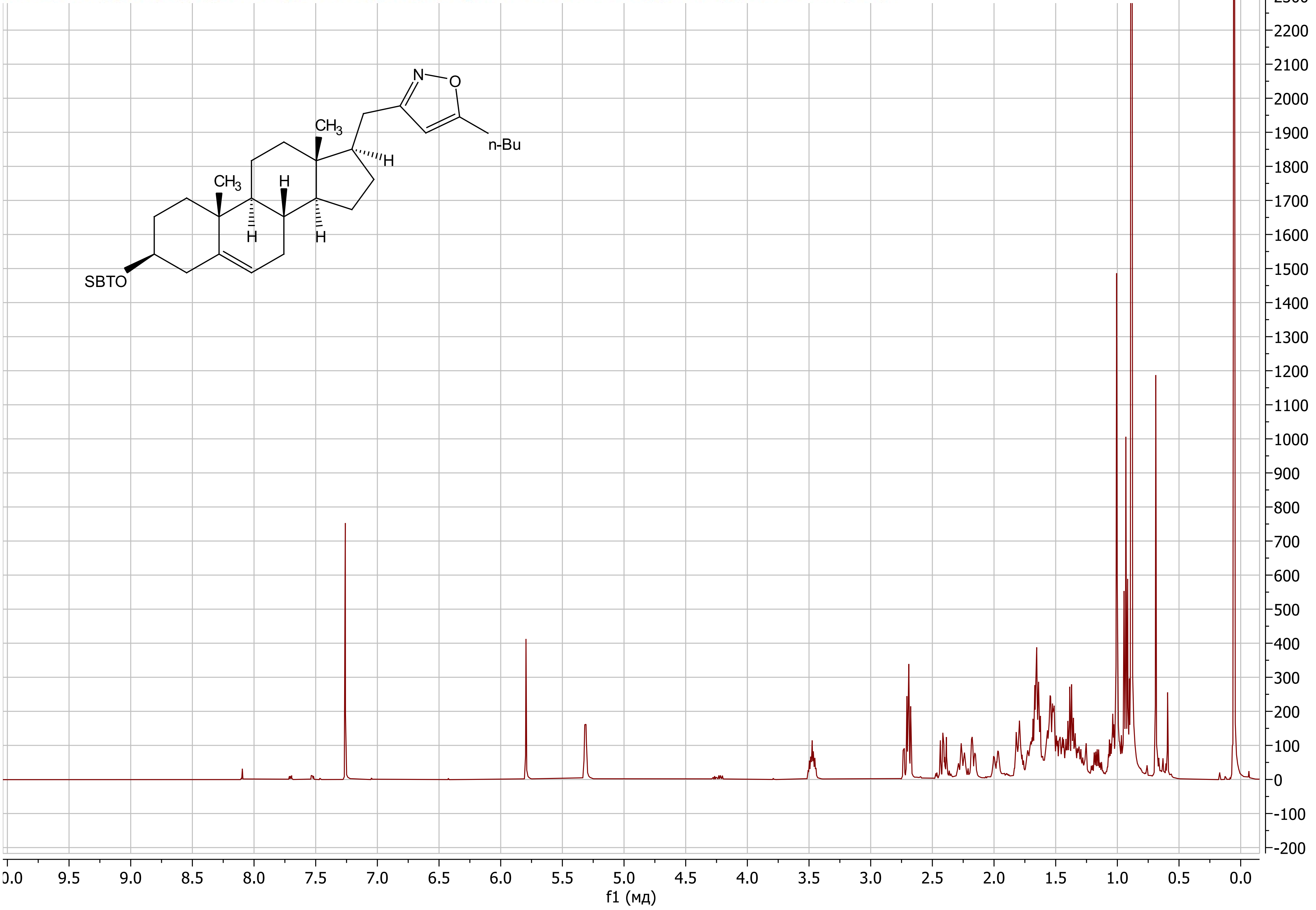

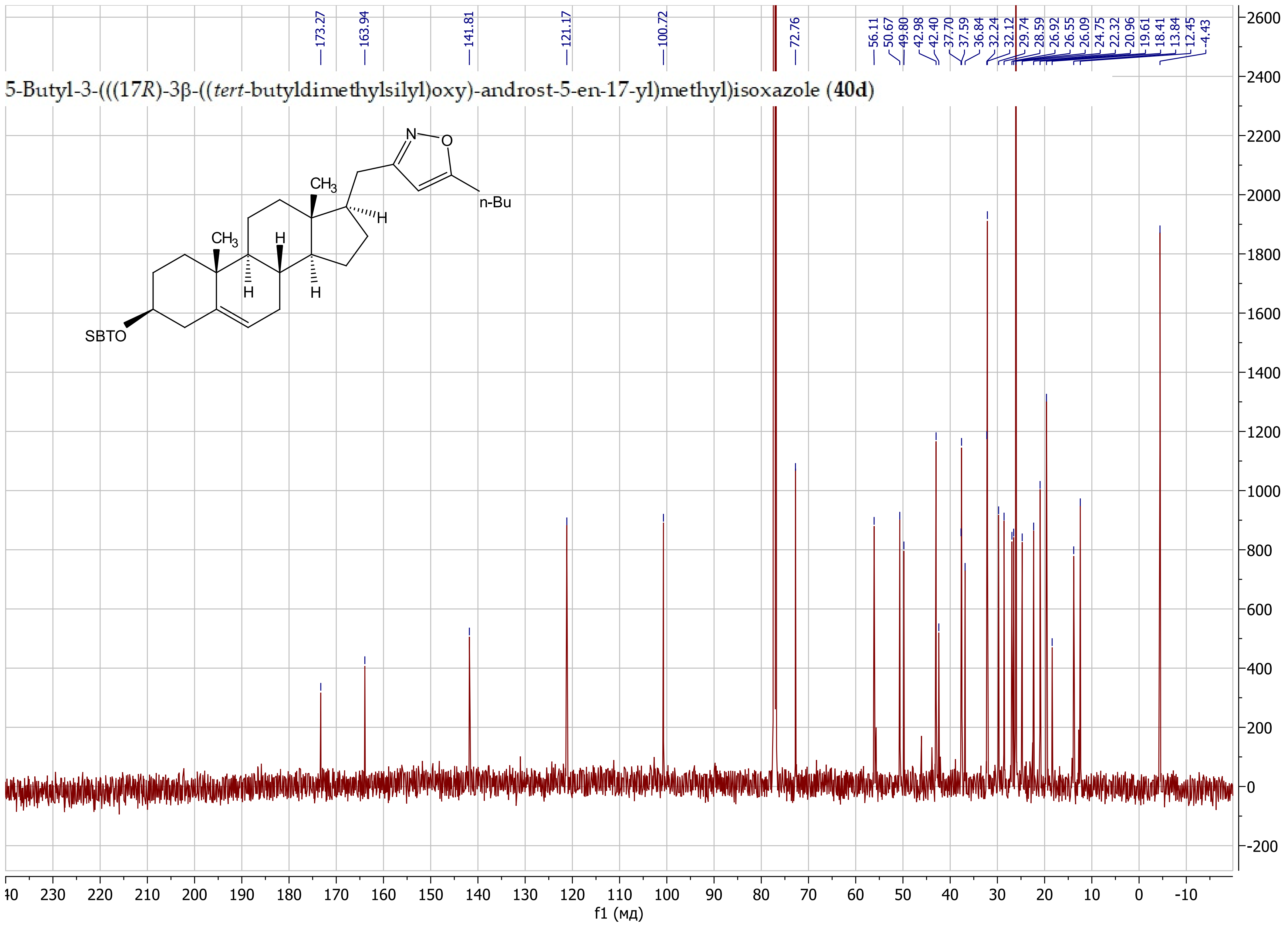
5-Butyl-3-(((17*R*)-3β-((*tert*-butyldimethylsilyl)oxy)-androst-5-en-17-yl)methyl)isoxazole (**40d**)
173.27
163.94
141.81
121.17
100.72
72.76
56.11
50.67
49.80
42.98
42.40
37.70
37.59
36.84
32.24
32.12
29.74
28.59
26.92
26.55
26.09
24.75
22.32
20.96
19.61
18.41
13.84
12.45
-4.43
N
O
n-Bu
CH3
CH3
H
H
H
H
SBTO
f1 (мд)

3-(((17*R*)-3β-((*tert*-Butyldimethylsilyl)oxy)-androst-5-en-17-yl)methyl)-5-phenylisoxazole (**40e**)

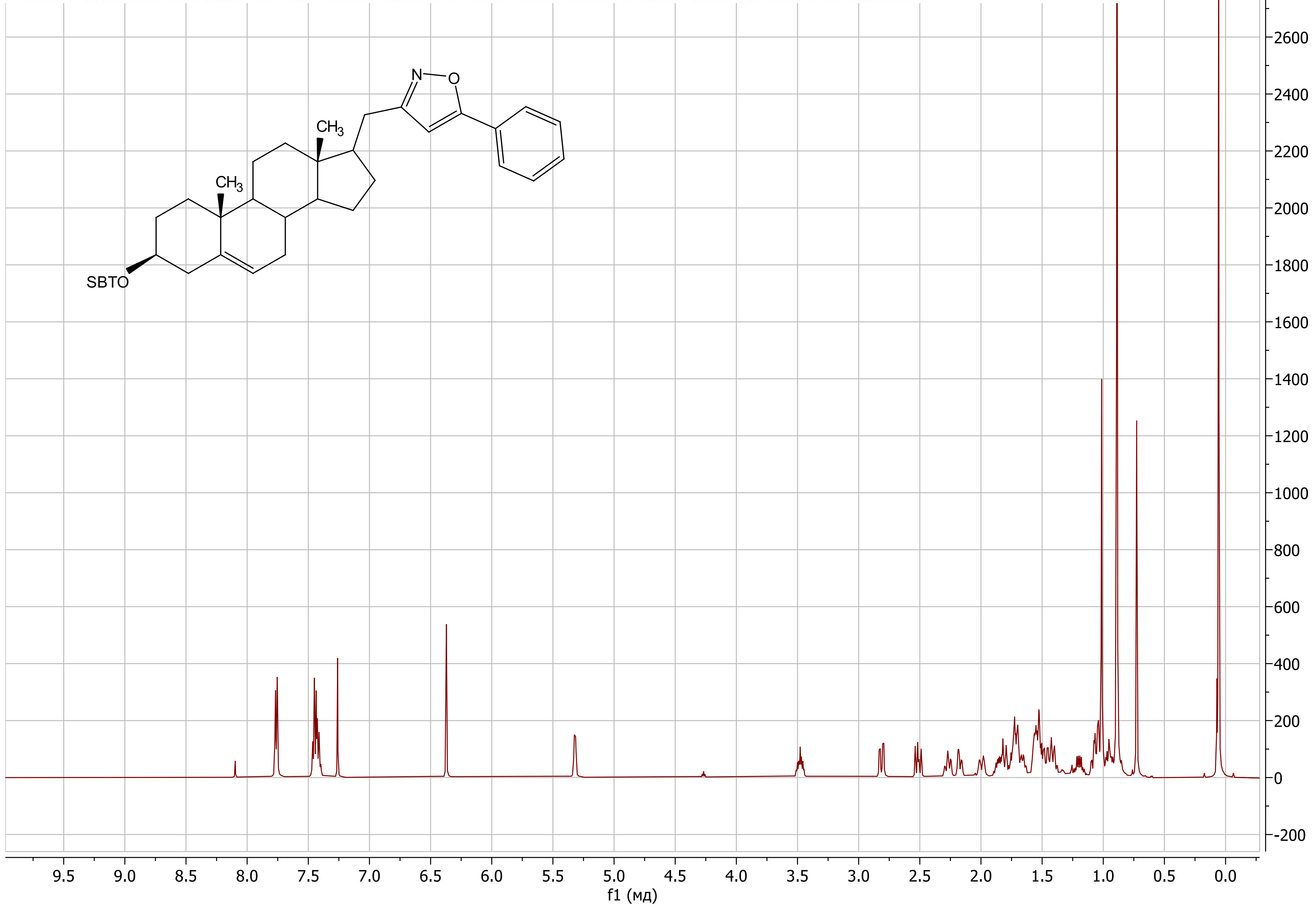

3-(((17*R*)-3β-((*tert*-Butyldimethylsilyl)oxy)-androst-5-en-17-yl)methyl)-5-phenylisoxazole (**40e**)

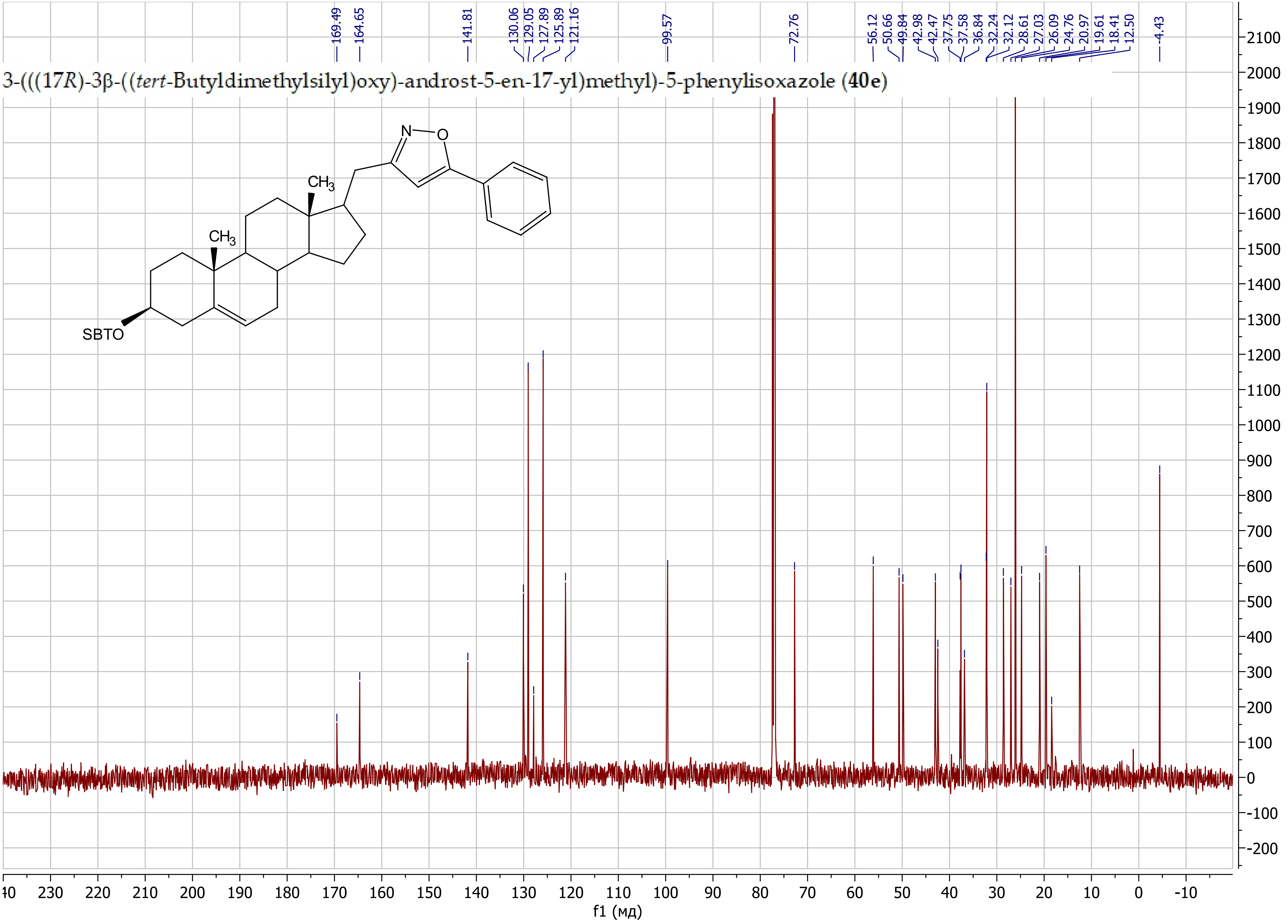

3-(((17*R*)-3β-((*tert*-Butyldimethylsilyl)oxy)-androst-5-en-17-yl)methyl)-5-(pyridin-3-yl)isoxazole (**40f**)

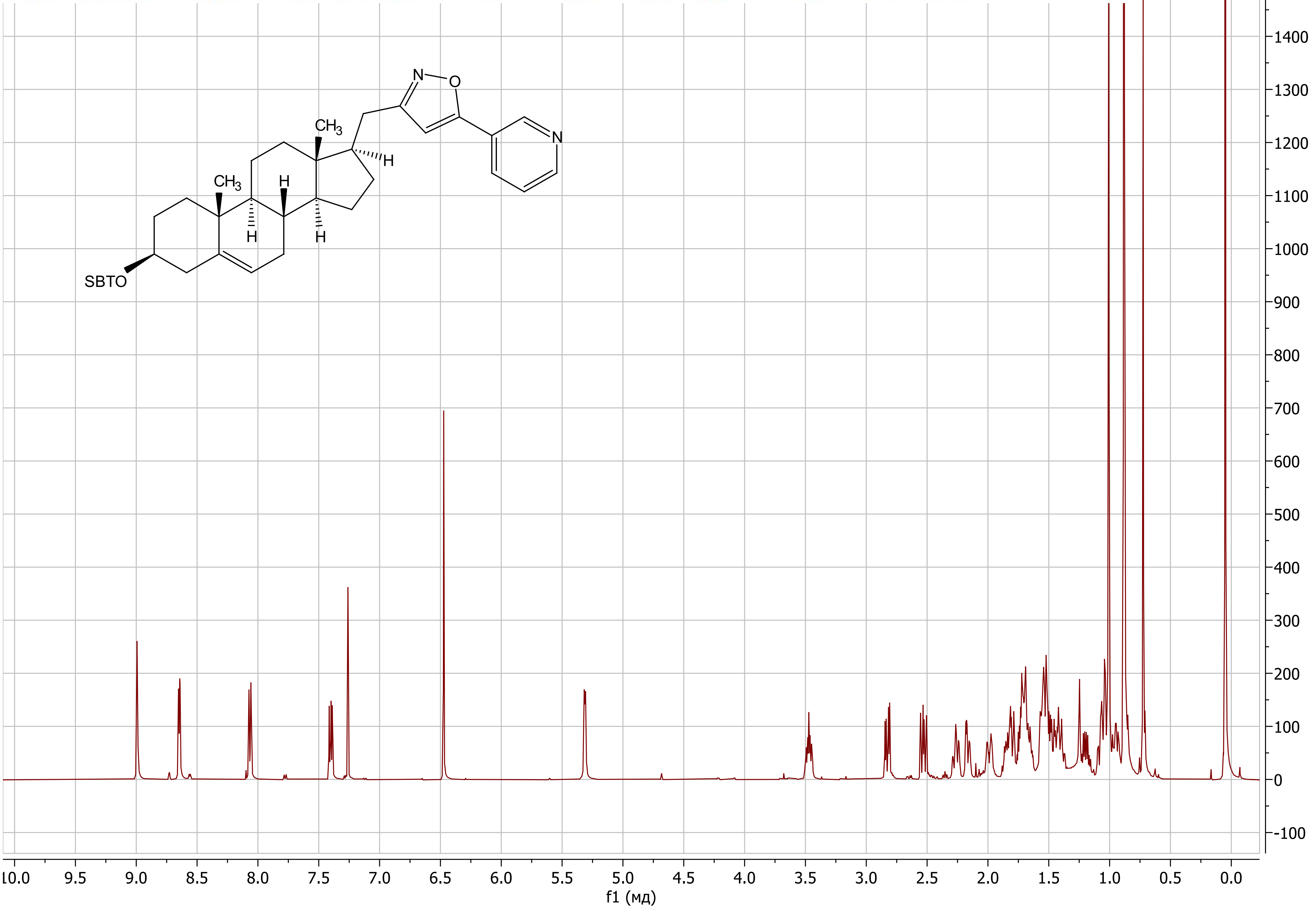

3-(((17*R*)-3β-((*tert*-Butyldimethylsilyl)oxy)-androst-5-en-17-yl)methyl)-5-(pyridin-3-yl)isoxazole (**40f**)

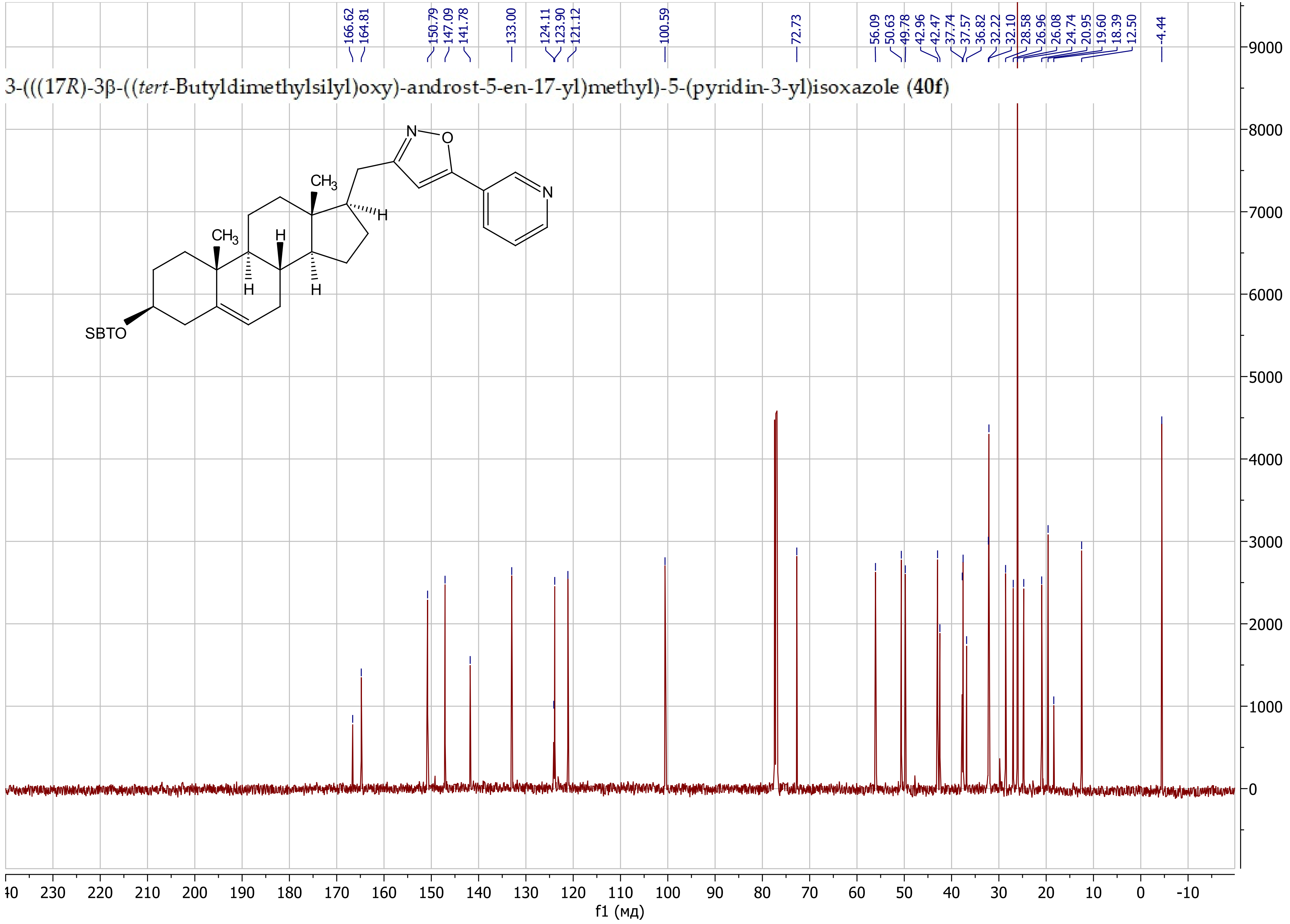

3-(((17R)-3β-((*tert*-Butyldimethylsilyl)oxy)-androst-5-en-17-yl)methyl)-5-(2-fluorophenyl)isoxazole (**40g**)

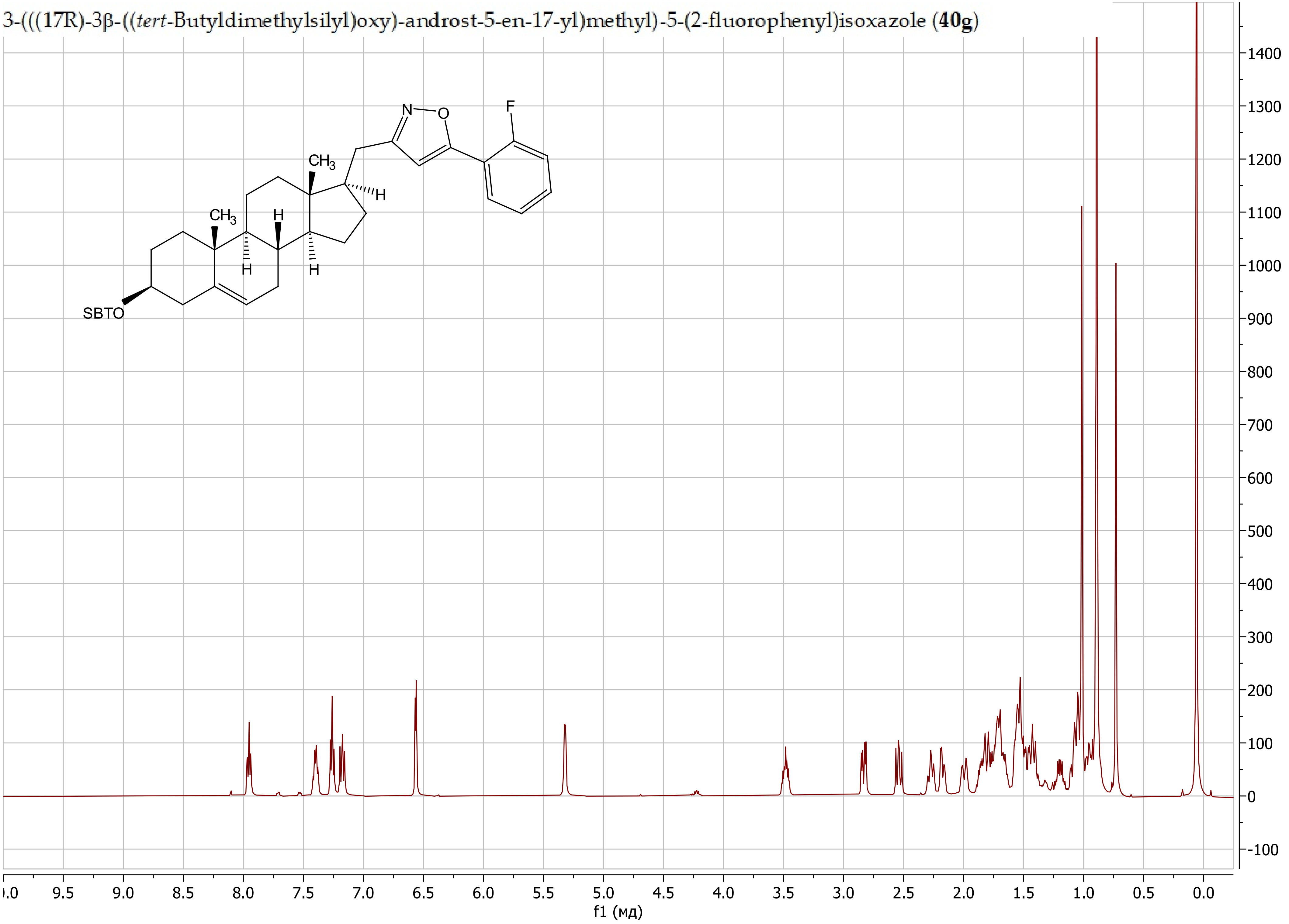

3-(((17R)-3β-((*tert*-Butyldimethylsilyl)oxy)-androst-5-en-17-yl)methyl)-5-(2-fluorophenyl)isoxazole (**40g**)

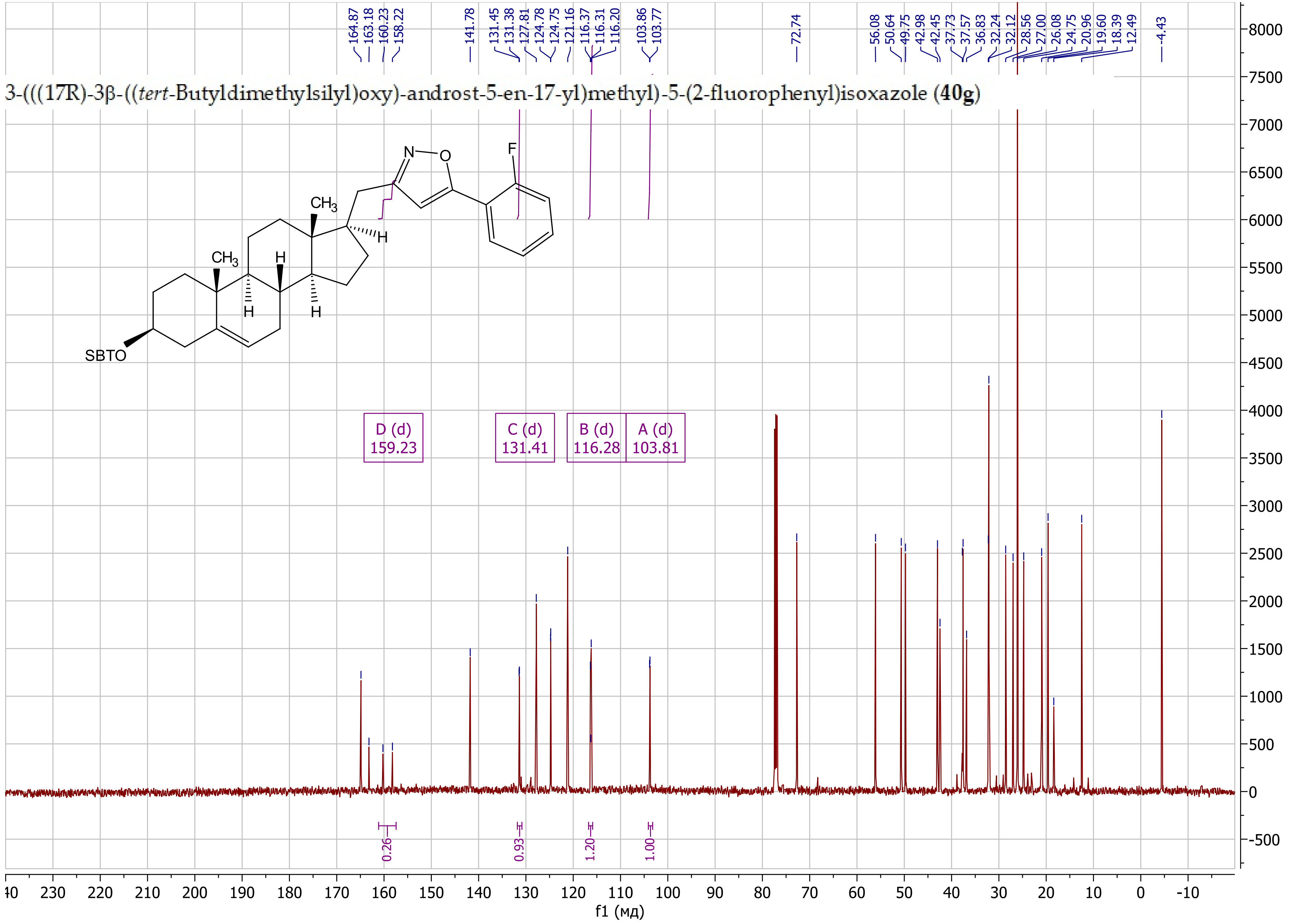

3-(((17*R*)-3β-((*tert*-Butyldimethylsilyl)oxy)-androst-5-en-17-yl)methyl)-5-(2-((tetrahydro-2*H*-pyran-2-yl)oxy)propan-2-yl)isoxazole (**40h**)

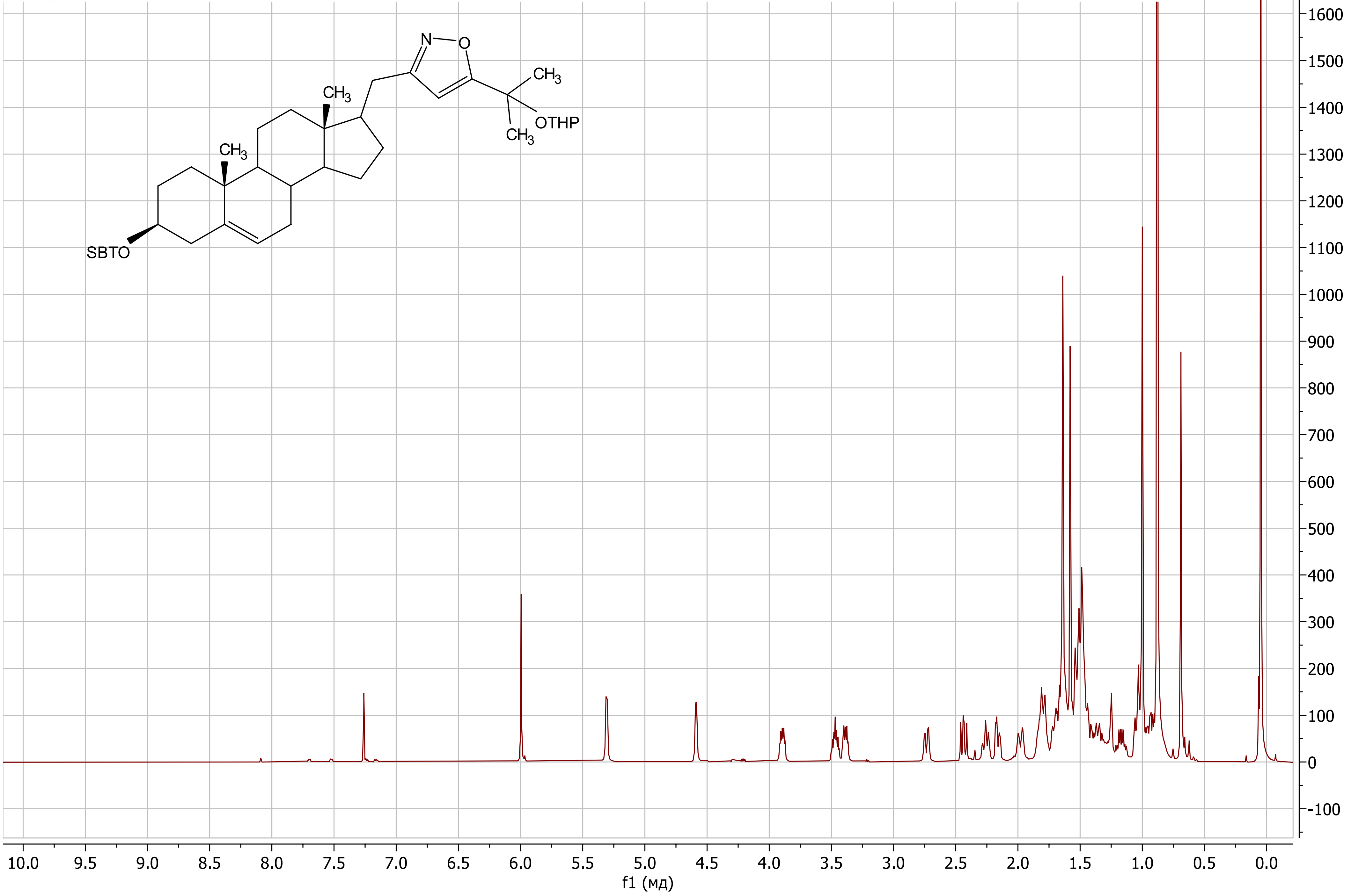

3-(((17*R*)-3β-((*tert*-Butyldimethylsilyl)oxy)-androst-5-en-17-yl)methyl)-5-(2-((tetrahydro-2*H*-pyran-2-yl)oxy)propan-2-yl)isoxazole (**40h**)

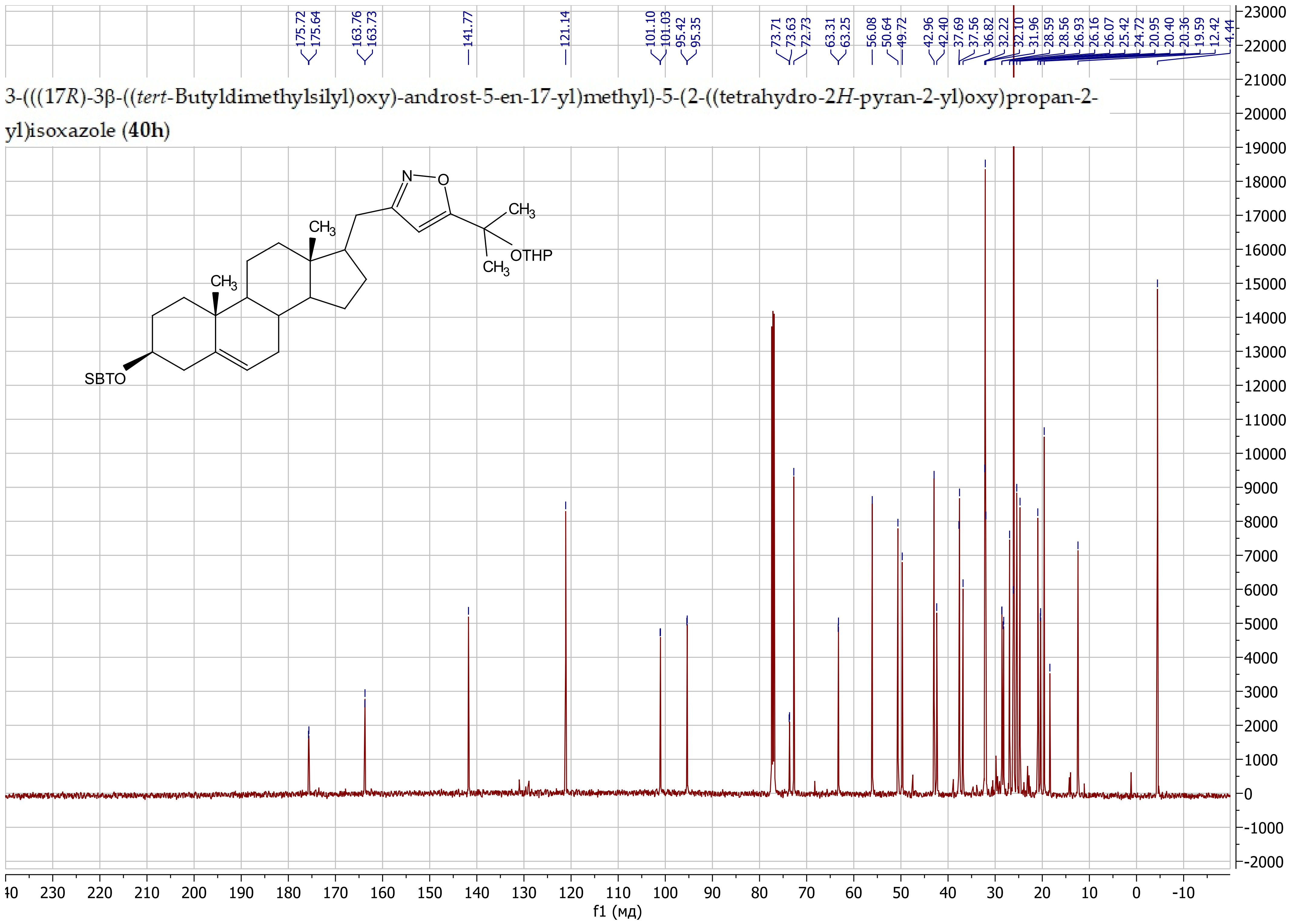

3-(((17*R*)-3β-((*tert*-Butyldimethylsilyl)oxy)-androst-5-en-17-yl)methyl)-5-(((tetrahydro-2*H*-pyran-2-yl)oxy)methyl)isoxazole (**40i**)

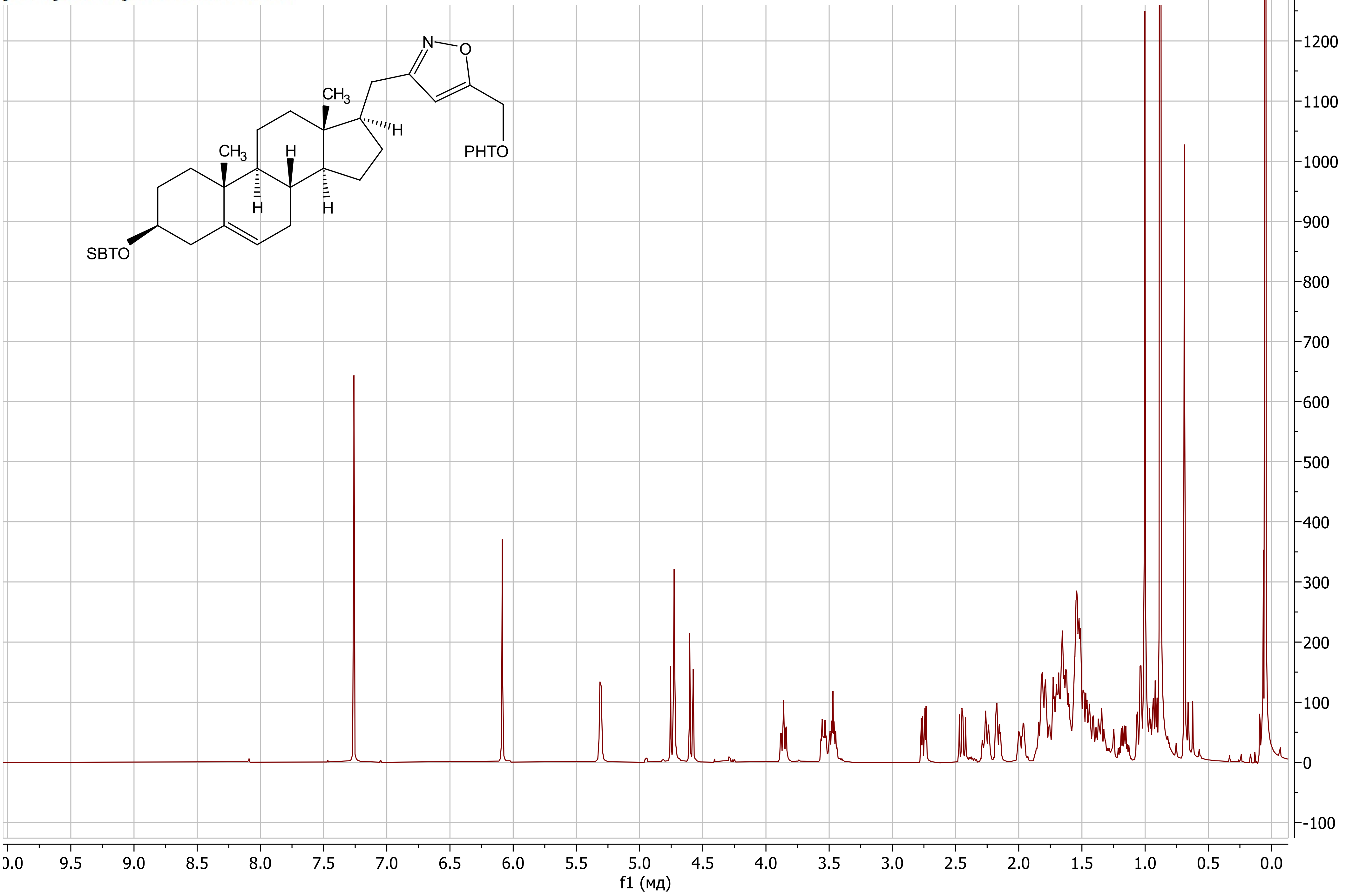

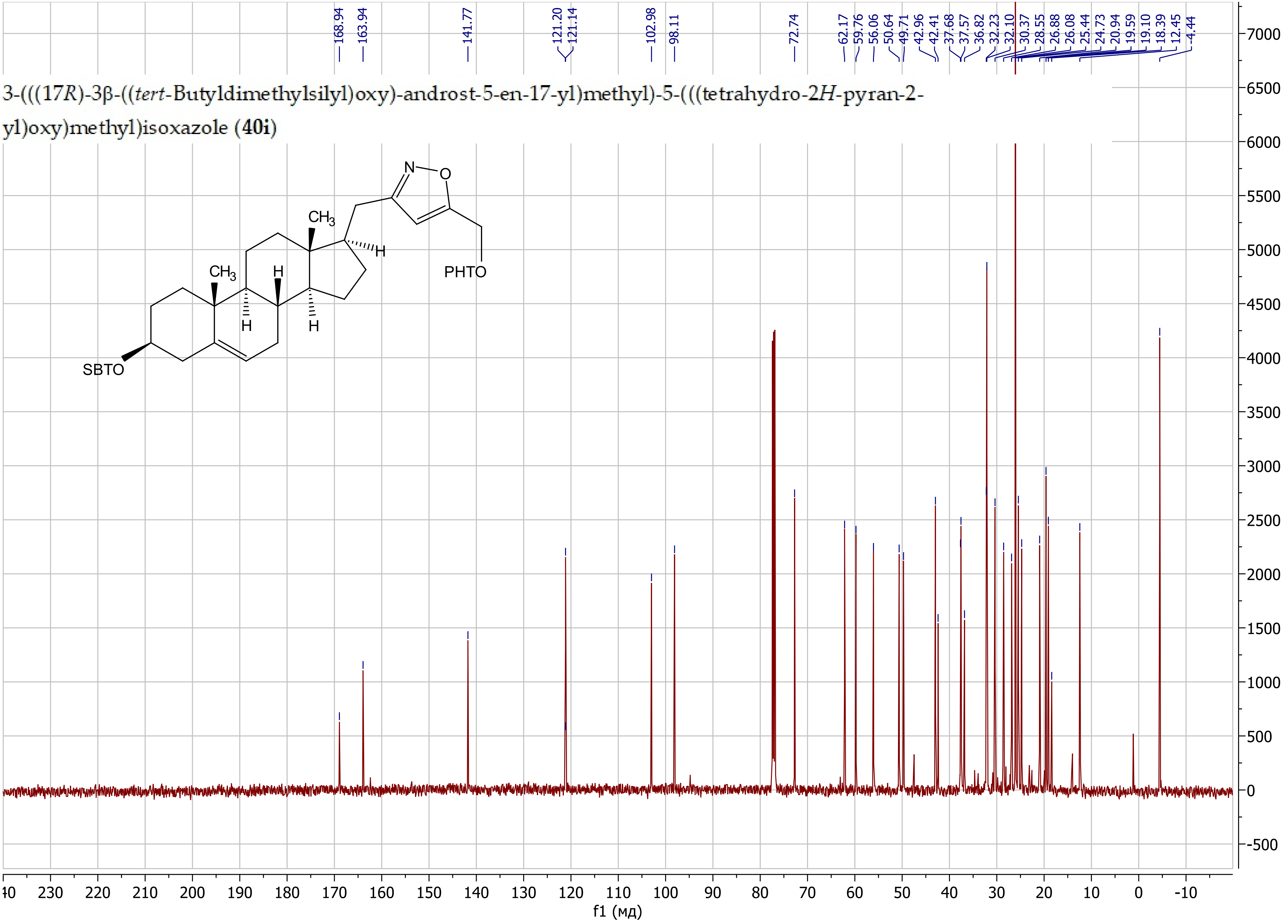

3-(((17*R*)-3β-((*tert*-Butyldimethylsilyl)oxy)-androst-5-en-17-yl)methyl)-5-(((tetrahydro-2*H*-pyran-2-yl)oxy)methyl)isoxazole (**40i**)

(17*R*)-17-(Isoxazol-3-ylmethyl)-androst-5-en-3β-ol (**41a**)

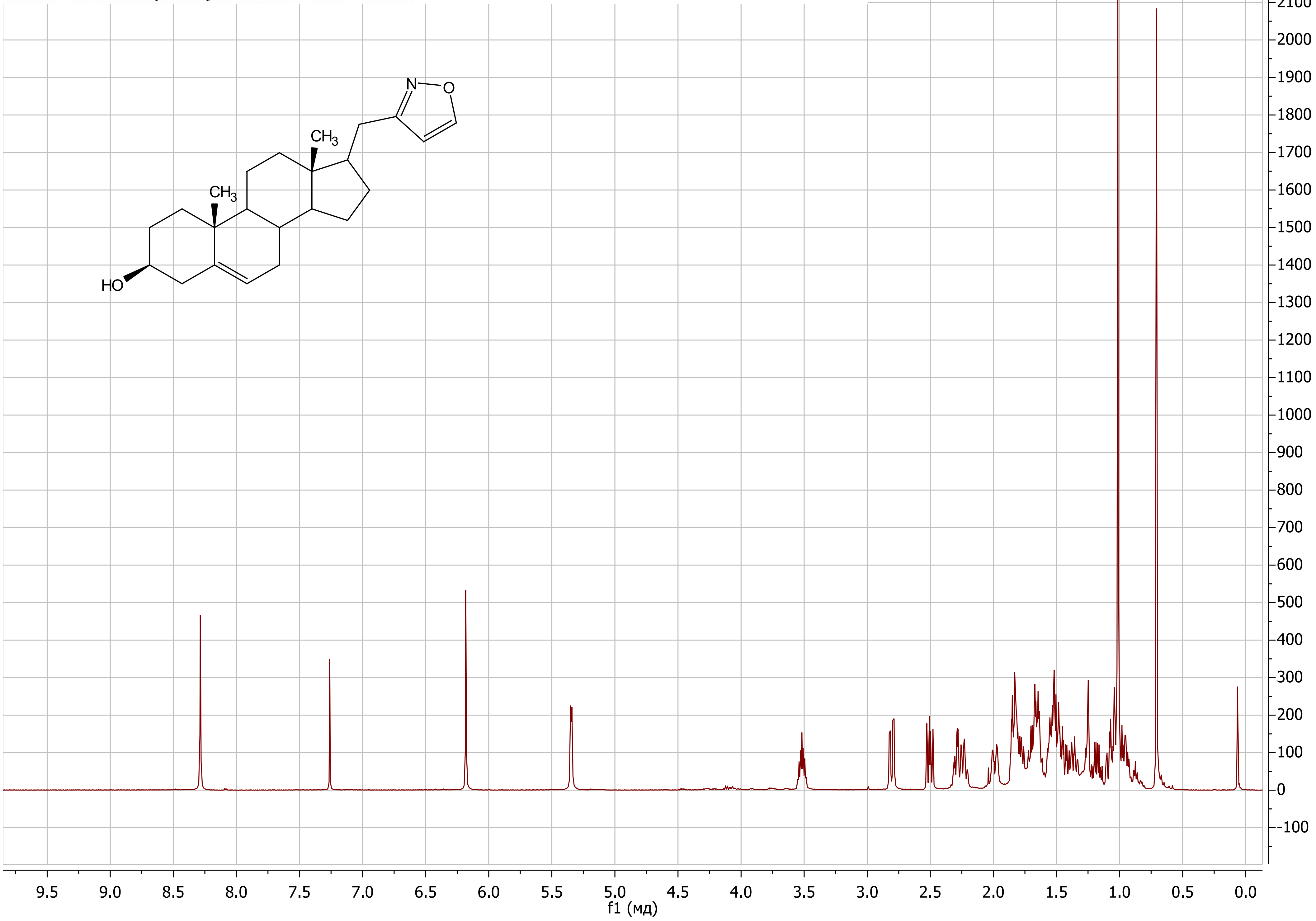

(17*R*)-17-(Isoxazol-3-ylmethyl)-androst-5-en-3β-ol (**41a**)

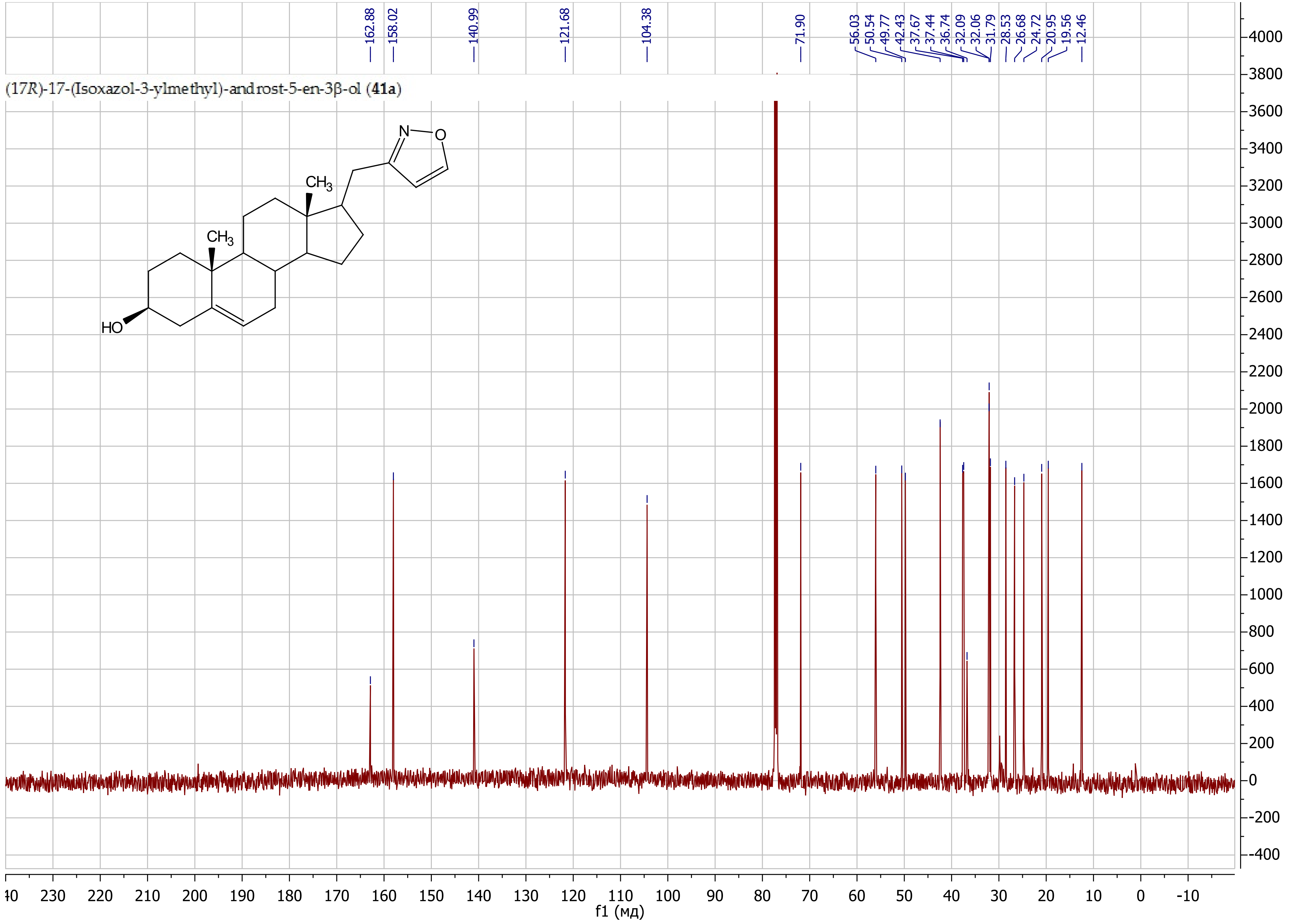

(17*R*)-17-((5-Butylisoxazol-3-yl)methyl)-androst-5-en-3β-ol (**41d**)

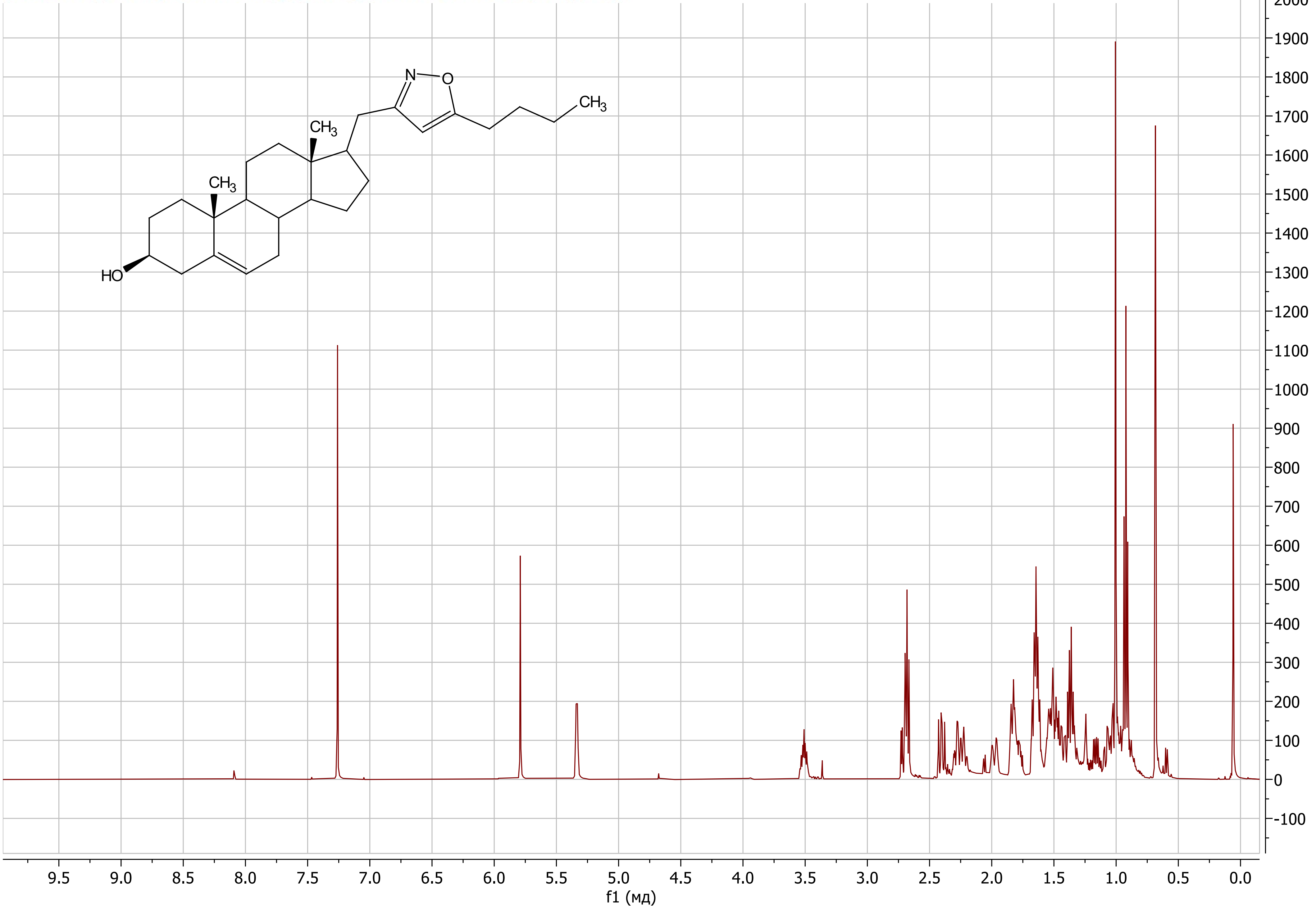

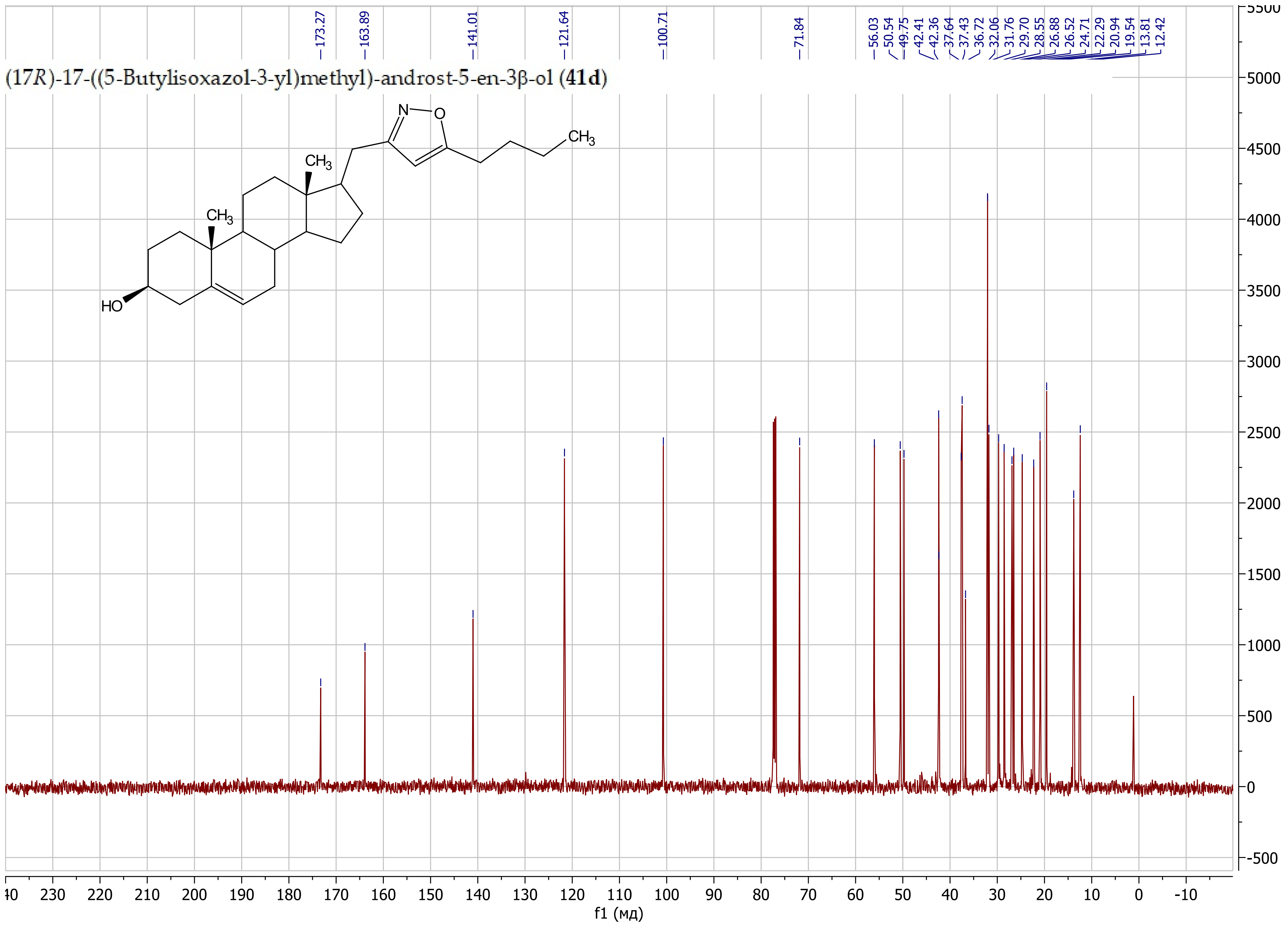

(17R)-17-((5-Butylisoxazol-3-yl)methyl)-androst-5-en-3β-ol (41d)
173.27
163.89
141.01
121.64
100.71
71.84
56.03
50.54
49.75
42.41
42.36
37.64
37.43
36.72
32.06
31.76
29.70
28.55
26.88
26.52
24.71
22.29
20.94
19.54
13.81
12.42
N
O
CH3
CH3
CH3
HO
f1 (мд)

(17*R*)-17-((5-Phenylisoxazol-3-yl)methyl)-androst-5-en-3β-ol (**41e**)

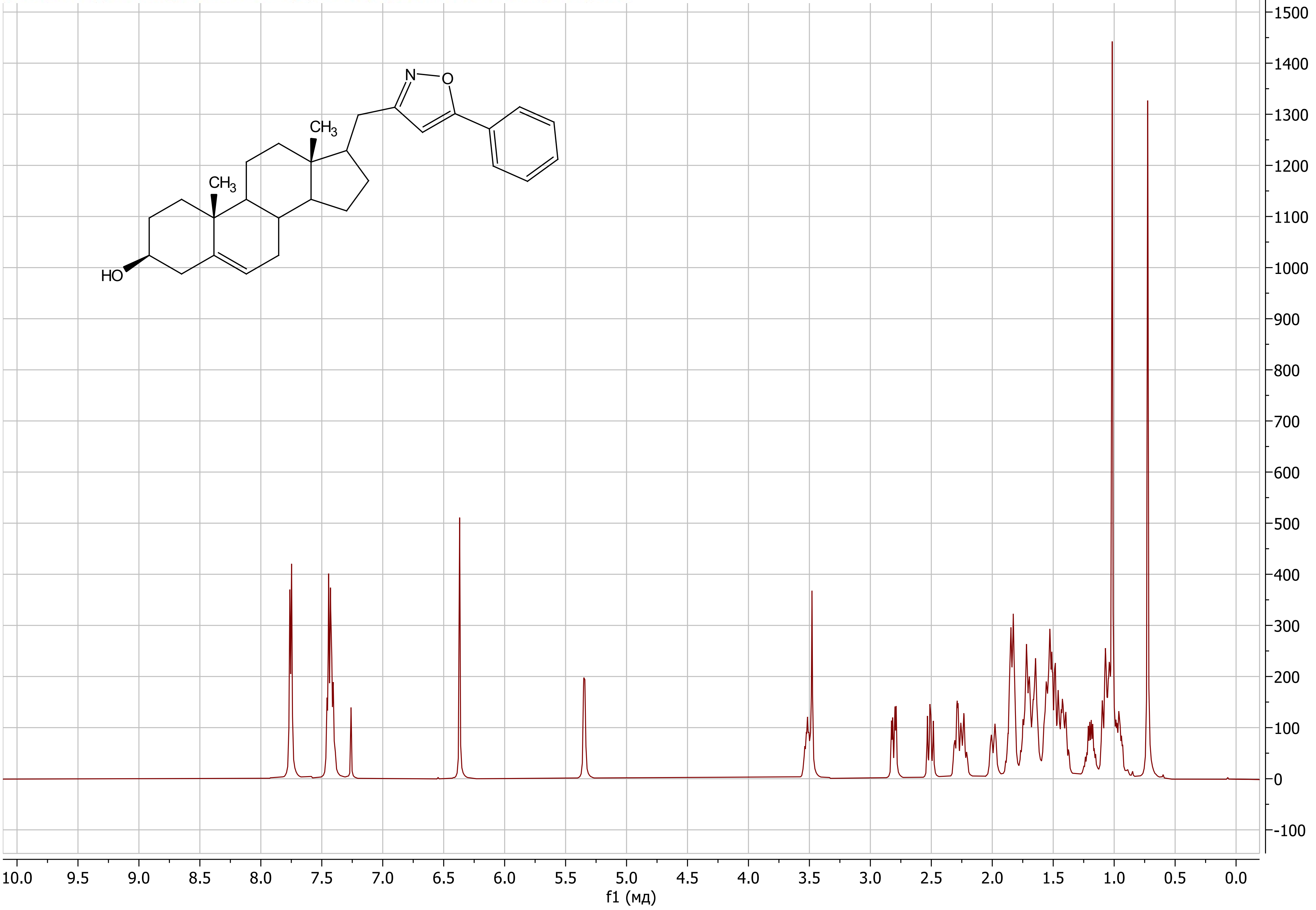

(17*R*)-17-((5-Phenylisoxazol-3-yl)methyl)-androst-5-en-3β-ol (**41e**)

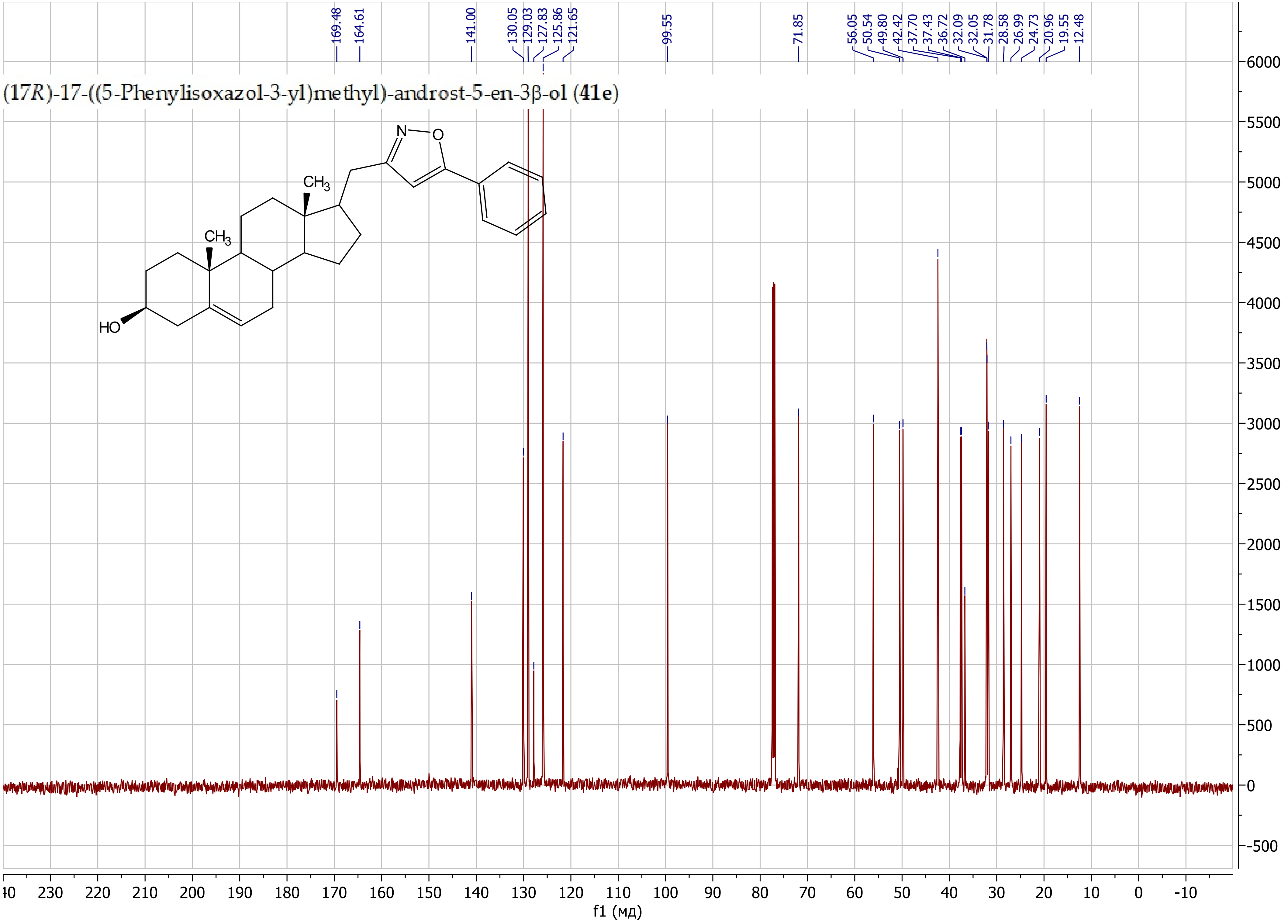

(17*R*)-17-((5-(Pyridin-3-yl)isoxazol-3-yl)methyl)-androst-5-en-3β-ol (**41f**)

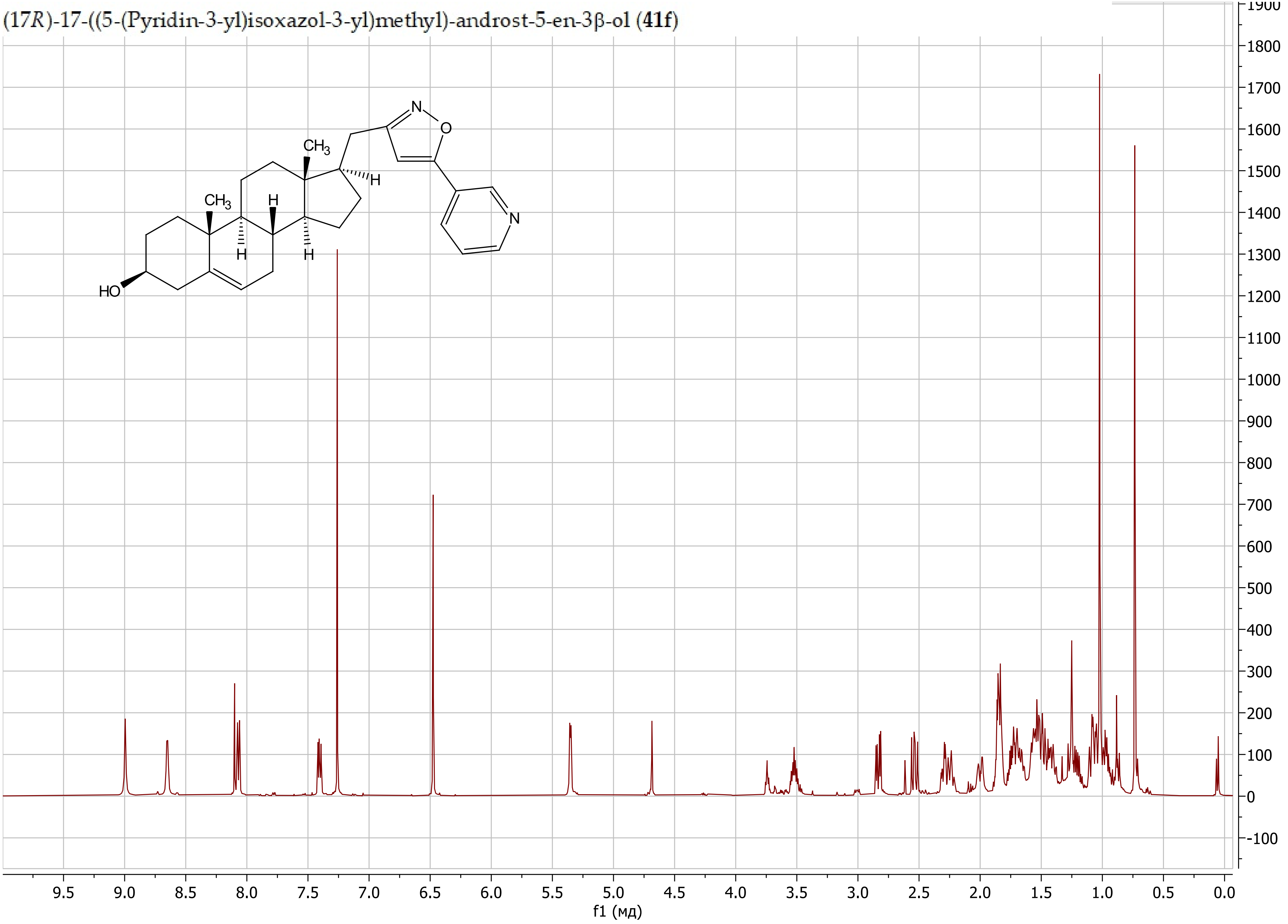

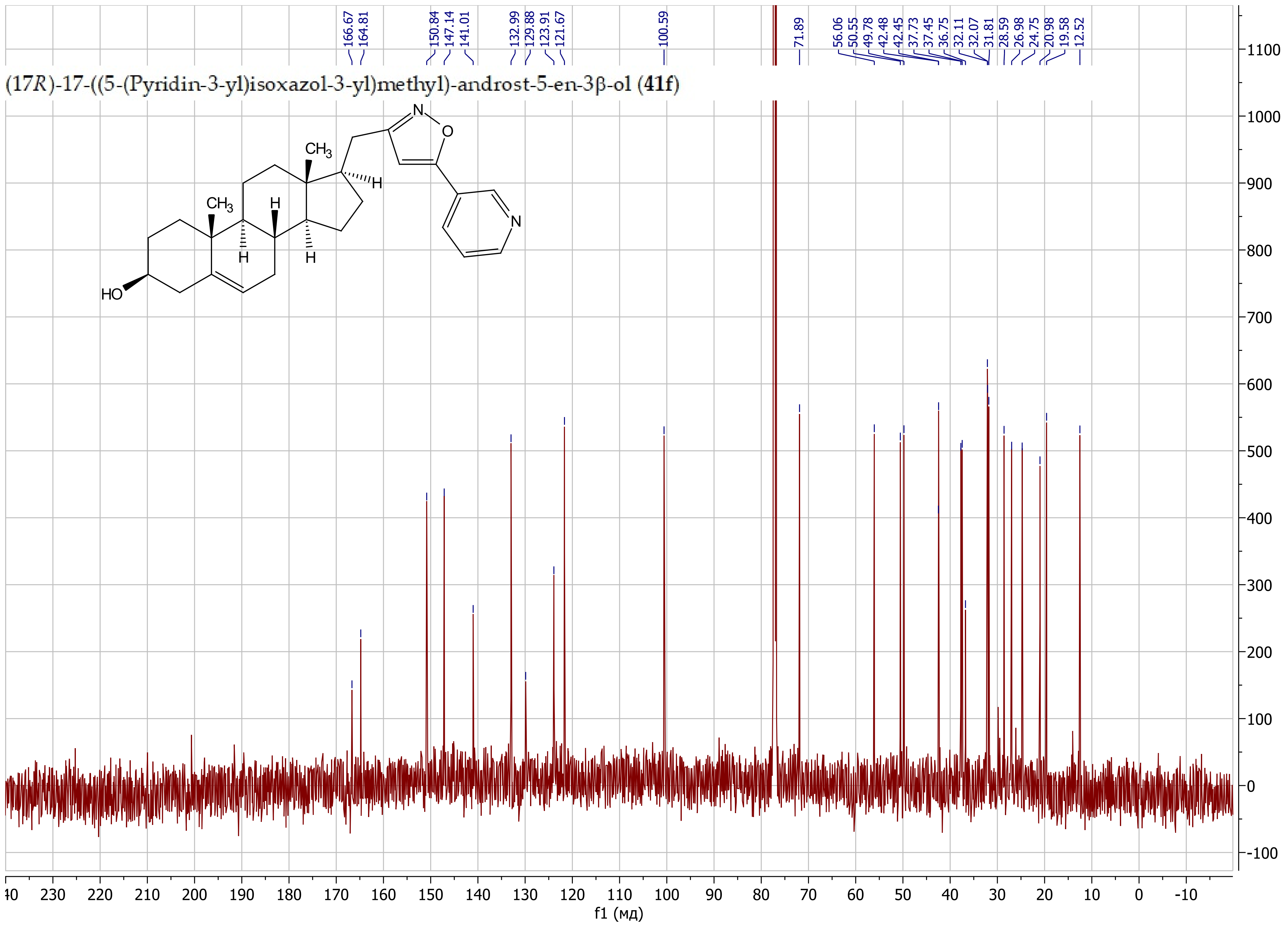

(17R)-17-((5-(Pyridin-3-yl)isoxazol-3-yl)methyl)-androst-5-en-3β-ol (41f)
166.67
164.81
150.84
147.14
141.01
132.99
129.88
123.91
121.67
100.59
71.89
56.06
50.55
49.78
42.48
42.45
37.73
37.45
36.75
32.11
32.07
31.81
28.59
26.98
24.75
20.98
19.58
12.52
CH3
CH3
H
H
H
H
HO
N
O
N
f1 (мд)

(17*R*)-17-((5-(2-Fluorophenyl)isoxazol-3-yl)methyl)-androst-5-en-3β-ol (**41g**)

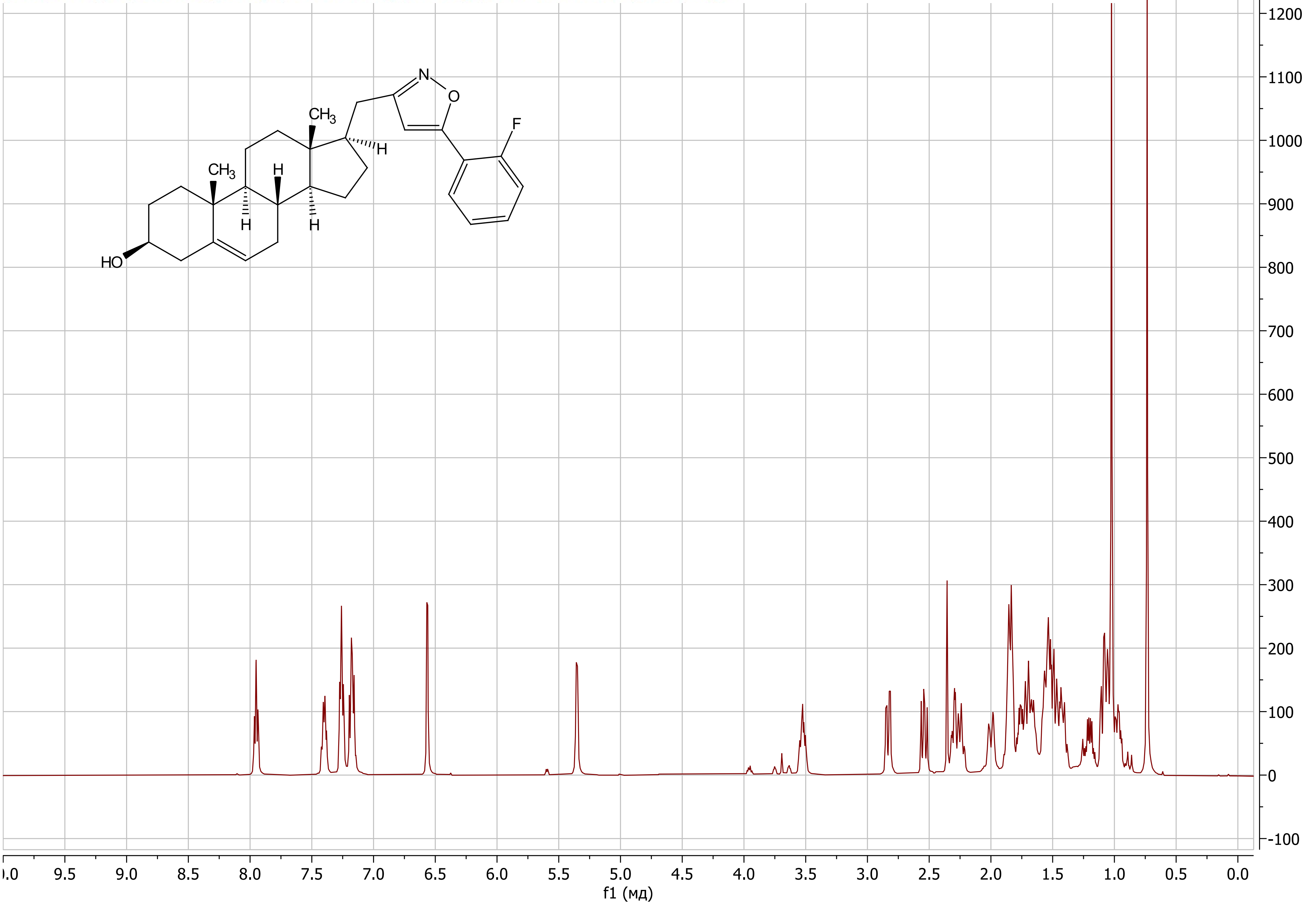

(17*R*)-17-((5-(2-Fluorophenyl)isoxazol-3-yl)methyl)-androst-5-en-3β-ol (41g)

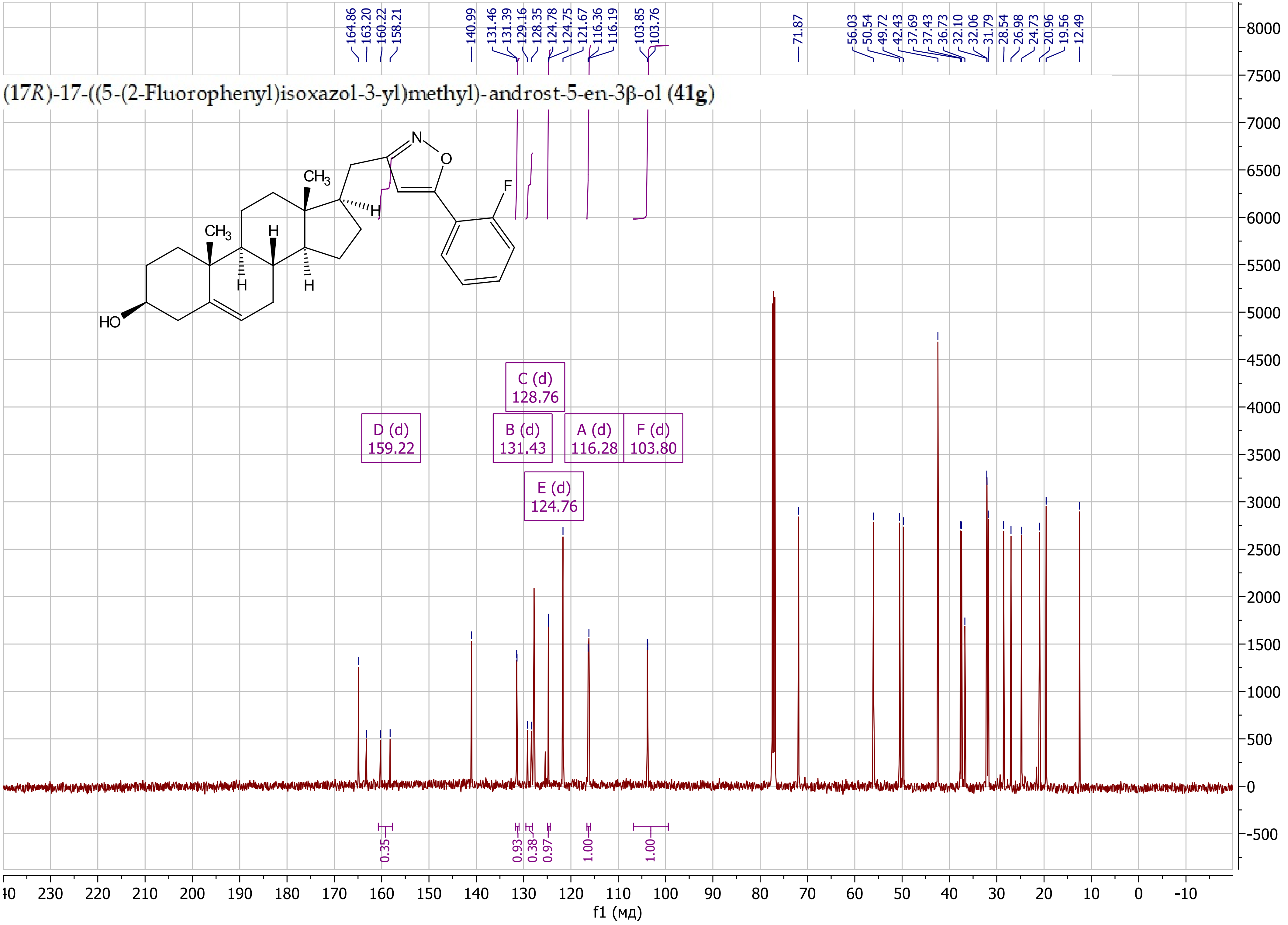

(17*R*)-17-((5-(2-Hydroxypropan-2-yl)isoxazol-3-yl)methyl)-androst-5-en-3β-ol (**41j**)

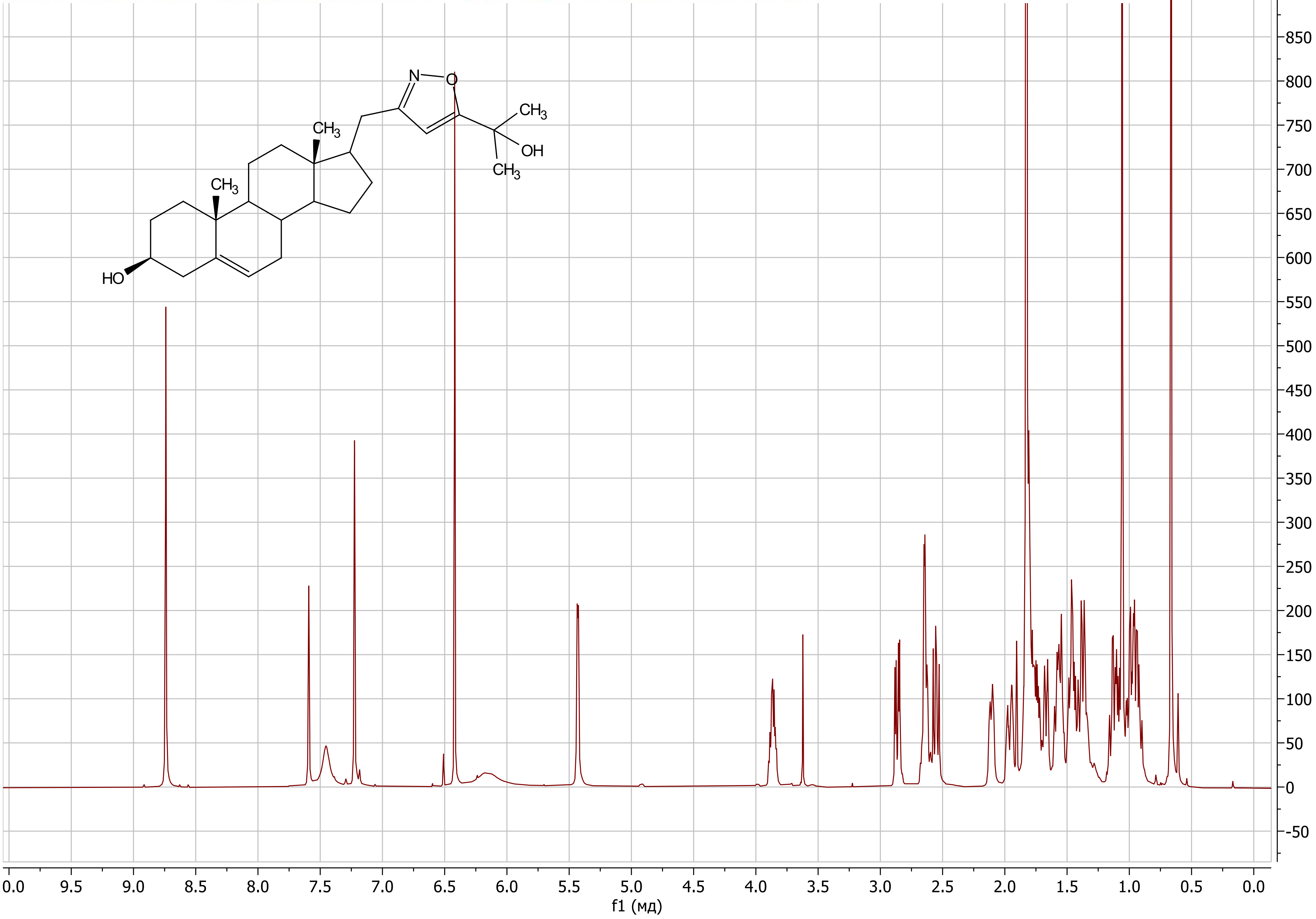

(17*R*)-17-((5-(2-Hydroxypropan-2-yl)isoxazol-3-yl)methyl)-androst-5-en-3β-ol (**41j**)

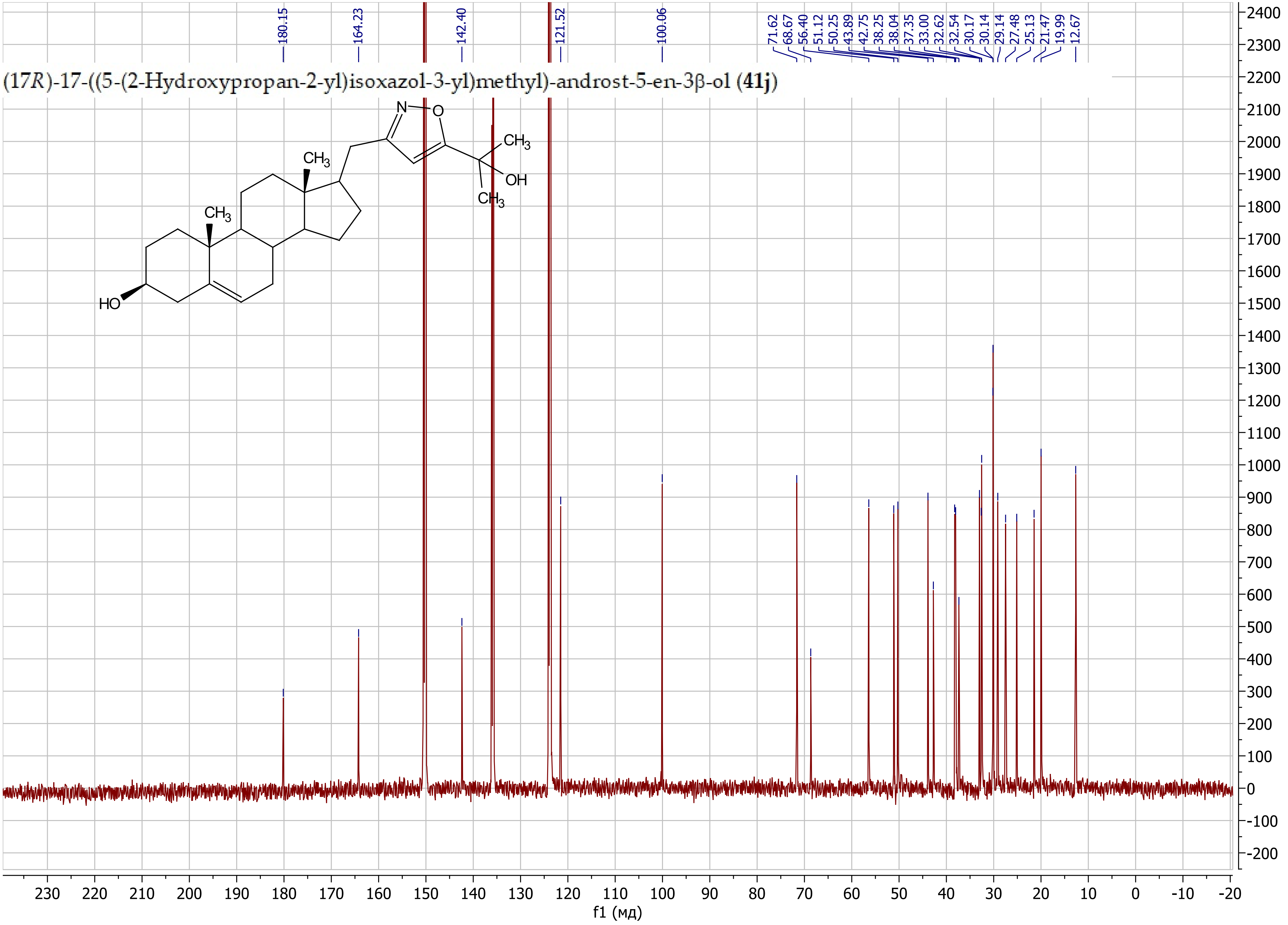

(17*R*)-17-((5-(Hydroxymethyl)isoxazol-3-yl)methyl)-androst-5-en-3β-ol (**41k**)

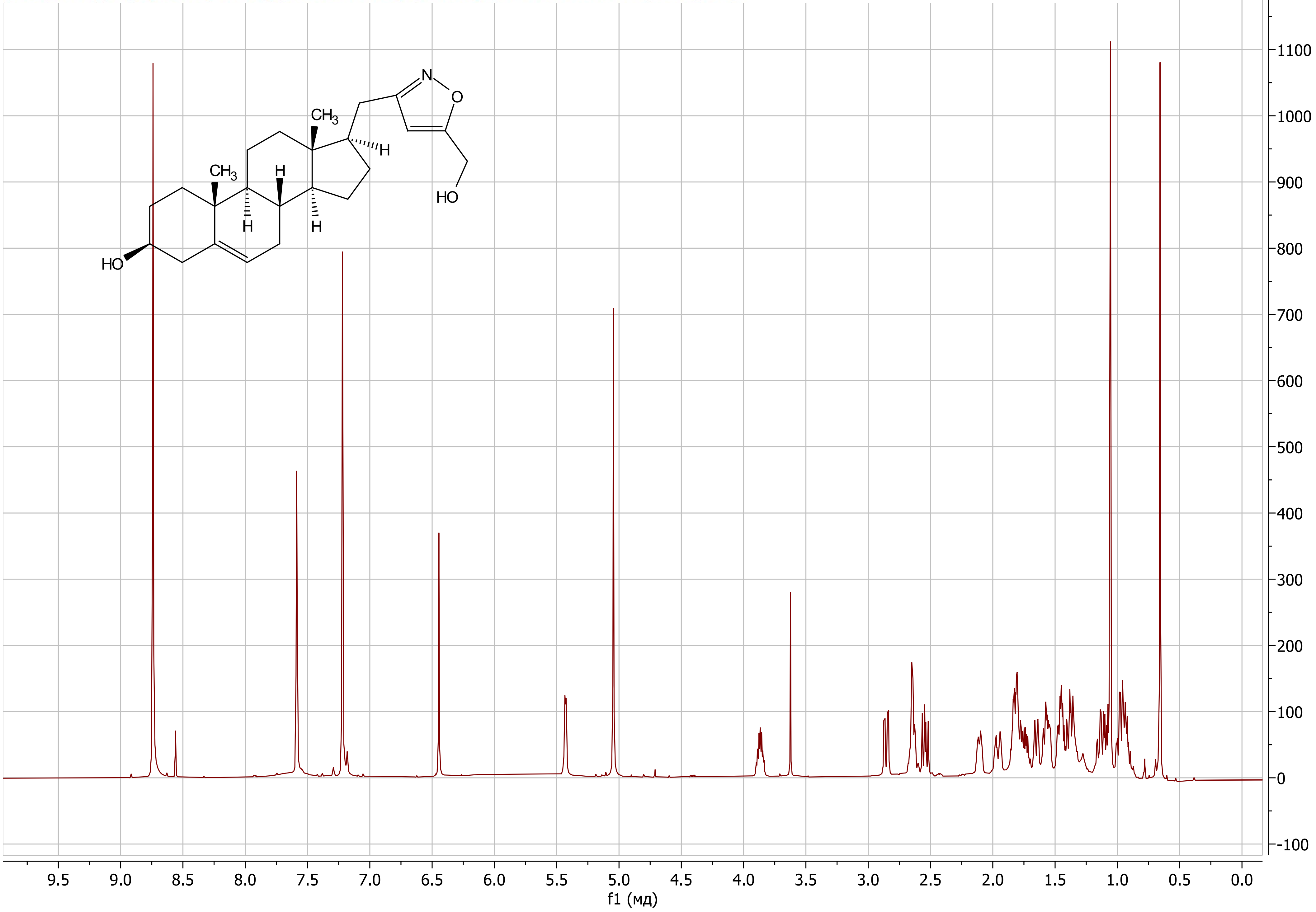

(17*R*)-17-((5-(Hydroxymethyl)isoxazol-3-yl)methyl)-androst-5-en-3β-ol (**41k**)

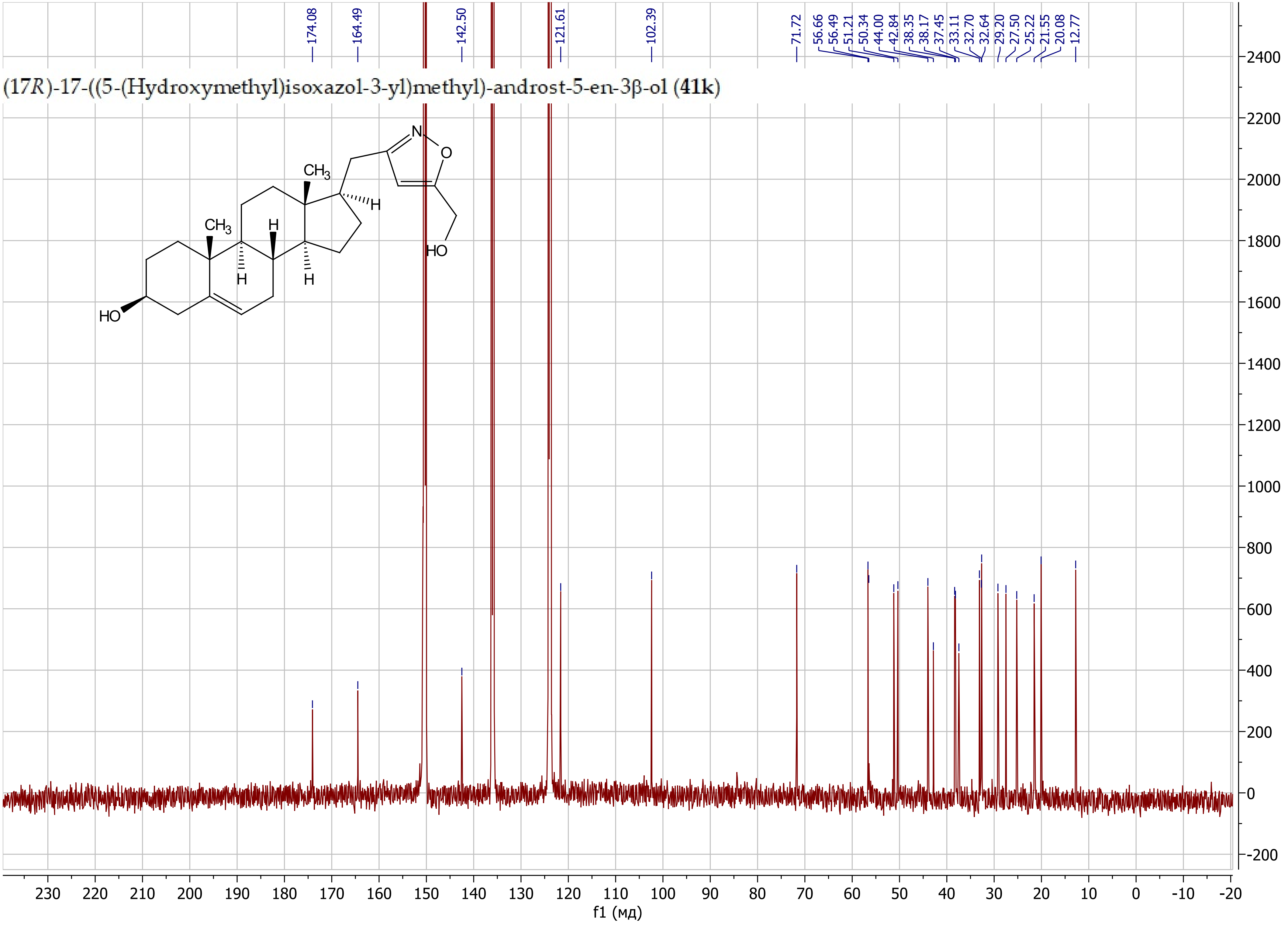

Supplement: Supplementary file 1 [file ijms-23-13534-s001.zip › ijms-2009886-supplementary.pdf]
